# Proteomic Analysis Provides Insights Into PPIP5K2 Function and Its Impact on Corneal Energy Metabolism

**DOI:** 10.1167/iovs.66.15.67

**Published:** 2025-12-23

**Authors:** Theresa Akoto, Caili Hao, Zhong Chen, Xiaowen Lu, Hongfang Yu, Chunfang Gu, Stephen B. Shears, Wenbo Zhi, Xingjun Fan, Mitchell A. Watsky, Yutao Liu

**Affiliations:** 1 Department of Cellular Biology and Anatomy, Medical College of Georgia, Augusta University, Augusta, Georgia, United States; 2 Inositol Signaling Group, Signal Transduction Laboratory, National Institute of Environmental Health Sciences, Research Triangle Park, North Carolina, United States; 3 Center for Biotechnology and Genomic Medicine, Medical College of Georgia, Augusta University, Augusta, Georgia, United States; 4 James & Jean Culver Vision Discovery Institute, Medical College of Georgia, Augusta University, Augusta, Georgia, United States

**Keywords:** keratoconus (KC), proteomics, PPIP5K2, corneal biomechanics, glycolysis

## Abstract

**Purpose:**

Variants in the Diphosphoinositol pentakisphosphate kinase 2 (*PPIP5K2*) gene may contribute to familial keratoconus (KC) pathogenesis, although the underlying molecular mechanisms remain elusive. We aimed to determine the proteomic effect of PPIP5K2 loss-of-function in response to two KC-related factors (cyclic mechanical stretch [CMS] and TGFβ1 treatment) in primary human corneal fibroblasts (HCFs).

**Methods:**

PPIP5K2 knockdown HCFs (*n* = 4) were cultured and treated with 0, 5, and 10 ng/mL of TGFβ1 with or without 15% CMS (1 cycle/second, 24 hours) using a Flexcell Tension system. Cellular proteins (50 µg) were digested with trypsin and analyzed through label-free liquid chromatography-mass spectrometry. Differentially expressed proteins (DEPs) were determined using 2-way ANOVA by adjusting the effect of TGFβ1 and CMS with false discovery rate (FDR)-adjusted *P* values ≤ 0.1. Gene ontology (GO) and pathway analyses were performed with the PANTHER classification system. Metabolic profiles of the PPIP5K2 knockdown in HCFs were evaluated with Seahorse assays.

**Results:**

A total of 1549 proteins were identified across 48 samples. With FDR ≤ 0.1, the effect of PPIP5K2, TGFβ1 treatment, and CMS status revealed 19 DEPs in the PPIP5K2 knockdown HCFs. GO and pathway analyses revealed over-representation of proteins involved in energy metabolism pathways. Furthermore, a reduction in ATP levels was observed, corroborated by diminished glycolytic function following the loss of PPIP5K2 in HCFs.

**Conclusions:**

This study identified many proteins and energy-related pathways following the loss of PPIP5K2 in HCFs, suggesting a potential role of PPIP5K2 in regulating glycolysis in the cornea.

Keratoconus (KC) results in progressive corneal thinning and steepening, forming a cone-like shaped cornea, which may lead to severe visual impairment.^1^ It is a leading indicator of corneal transplantation in the United States and worldwide.^2^ KC is often aggressive, with its onset in the teenage years, and progresses for approximately 10 to 20 years before stabilizing in the third to fourth decade of the individual's life.^3^^,^^4^

Although environmental factors, such as frequent eye rubbing and wearing of contact lenses, hormonal, and UV exposure have been reported as risk factors contributing to the pathogenesis of KC, there has been strong underlying evidence suggesting genetic contributions to the condition.^2^^,^^4^^–^^6^ Some reports suggest a recessive inheritance pattern for KC, such as in consanguineous marriages, but most reports indicate an autosomal dominant pattern of inheritance.^7^^,^^8^ Furthermore, many genes have been implicated as candidate genes that may contribute to the disease. However, these observations have been reported in less than 10% of KC cases.^1^^,^^8^^–^^15^ Despite the reports on genetic contributions, the disease etiology is still poorly understood, suggesting the urgent need for research into KC.

In previous work, using whole exome sequencing and whole genome sequencing, our group identified two pathogenic variants in the phosphatase domain of Diphosphoinositol pentakisphosphate kinase 2 (PPIP5K2) in two multiplex KC families.^7^ PPIP5K2 functions as cell signaling kinases and phosphatases involved in inositol pyrophosphate metabolism.^7^ Increased levels of 5-InsP7 and 1, 5-InsP8 are involved in apoptosis and modulate the production of ATP in the mitochondria, respectively.^16^^,^^17^ Studies have shown that CRISPR-based knockout of PPIP5K1/2 in HCT116 cells and HEK293 cells decreased levels of 1,5-InsP8, resulting in increased intracellular levels of ATP due to increased oxidative phosphorylation and enhanced glycolysis.^16^ KC corneas have an increased level of mitochondrial DNA damage.^18^ This damage may affect oxidative phosphorylation proteins, resulting in reduced ATP production and increased reactive oxygen species (ROS), which may lead to apoptosis of corneal stromal keratocytes in patients with KC.

Mass spectrometry (MS)-based proteomics is a powerful tool to answer many biological questions, especially regarding protein profiling and identification.^19^^–^^21^ Studies have been conducted to profile the proteome of corneal epithelial and stromal cells and biological fluids, including tears from patients with KC.^22^^–^^24^ However, to the best of our knowledge, no report exists on the proteome changes due to the contributions of multiple risk factors of KC, such as genetics,^1^^,^^2^^,^^7^^,^^25^ altered TGFβ signaling,^26^^,^^27^ and biomechanical factors.^28^^–^^31^

Therefore, we sought to determine the proteome changes in human corneal fibroblasts (HCFs) after the knockdown of PPIP5K2 in response to CMS and TGFβ1 treatment. In addition, we sought to determine if these proteins were involved in pathways related to energy metabolism. Functional studies were conducted to determine the metabolic profiles of HCFs following the knockdown of PPIP5K2.

## Methods

### Culture of Primary Human Stromal Fibroblasts

De-identified human donor corneal rim tissue was received from the Augusta University Eye Clinic or the Eye Surgery Center of Augusta in Augusta, Georgia, after corneal transplantation. HCFs (Table 1) were isolated and cultured according to the established protocols as previously described.^7^^,^^32^^,^^33^ Briefly, after removing the epithelium and endothelium layers, the dissected stroma tissue was cut into small pieces, digested with collagenase overnight, centrifuged to collect the HCFs. The isolated fibroblasts were validated with the expression of α-smooth muscle actin (αSMA), COL1A1, and COL3A1 from the four primary human corneal stromal cell donors using antibody-based Western blots with separate Western blots against each marker protein (Supplementary Fig. S1).^32^^,^^33^ We used rabbit anti-αSMA antibody (Abcam, catalog #ab32575, with 1:3000 dilution; Waltham, MA, USA), rabbit anti-COL1A1 antibody (Invitrogen, catalog #PA5-29569, with 1:1000 dilution; Carlsbad, CA, USA), and goat anti-COL3A1 antibody (SouthernBioTech, catalog #1330-01, with 1:500 dilution; Homewood, AL, USA). The secondary antibody was peroxidase-conjugated anti-rabbit IgG secondary antibody (ThermoFisher Scientific, Catalog #31460, Waltham, MA, USA). For αSMA and COL1A1, total protein loading was 5 µg per sample whereas 100 µg was used for COL3A1. Membranes were exposed to Clarity enhanced chemiluminescence (ECL) reagent (Bio-Rad) and visualized using a ChemiDoc MP (Bio-Rad). Detection and quantification of band intensities was conducted using Image Lab 5.2.1 software (Bio-Rad). We tried our best to match the donors’ clinical phenotypes with ages approximately 50 years. HCFs from four postmortem donors (*n* = 4) were cultured in high glucose Dulbecco's modified eagle medium (DMEM; Gibco), 10% fetal bovine serum, and 1% penicillin-streptomycin at 37°C with 5% CO_2_ in a humidified incubator with media changes every 2 days.

**Table 1.** Clinical Phenotypes of Primary Human Corneal Stromal Fibroblasts

Sample ID	Age, Y	OD/OS	Sex	Ethnicity	Cause of Death	PMI (h:min)
HCF-1	54	OS	N/A	N/A	N/A	4:30
HCF-2	N/A	OD	N/A	N/A	N/A	14:54
HCF-3	51	OS	N/A	N/A	N/A	7:27
HCF-4	54	OD	Female	Caucasian	Cardiac arrest	6.25

N/A, not available; OD, oculus dexter (right eyes); OS, oculus sinister (left eyes); PMI, postmortem interval.

### shRNA-based PPIP5K2 Knockdown

The shRNA was produced through an miR30-based system using the MSCV-miR30-eGFP vector^34^ for stable knockdown cells following a previous report.^35^ Three sets of independent shRNA targeting *PPIP5K2* KD1: TTTGTGAAAGGTTTATACAG, KD2: CGGTTCAAAATAGCATAACG, and KD3: GTGAAAAGTGCAAATATGAA, and one set of scrambled shRNA: CTCCCGTGAATTGGAATCC were used for HCFs. The shPPIP5K2 #1 was selected for subsequent downstream studies to knockdown PPIP5K2 protein expression in HCFs from four different donors (*n* = 4). All the HCFs used in the study were between passages 7 and 10, mostly due to the lentiviral transduction and selection of the positive cells. The morphology, including cell shapes and behaviors, was closely monitored during the experimental process.

As previously described, the miR30 shRNA lentiviral particles were produced from the HEK293T cells.^36^ HCFs were infected with mir30 shRNA lentivirus for 48 hours, and the stable RNAi cells were grown out of the optimal puromycin selection (2 µg/mL) selection. PPIP5K2 knockdown was validated by immunoblot analysis, as described below. Briefly, cultured HCF cells with PPIP5K2 knockdown (*n* = 4) or scrambled controls (*n* = 4) were processed using the Pierce RIPA Lysis and Extraction Buffer supplemented with 1% of Halt Protease and Phosphatase Inhibitor (100X) to collect cell lysates for protein quantification using the Pierce BCA protein assay kit. Then, 20 µg protein lysates were used for Western blotting using primary antibody-rabbit anti-PPIP5K2 (Abcam, Waltham, MA, USA; ab204374, 1:500), secondary antibody-peroxidase-conjugated secondary antibody (Invitrogen, Franklin, MA, USA; goat anti-rabbit, 1:3000), horseradish peroxidase substrate, and rabbit anti-GAPDH primary antibody (Cell Signaling Technology, Danvers, MA, USA; #2118, 1:300,000).

### Sample Preparation for Mass Spectrometry Analysis

HCFs (*n* = 4) transduced with the shControl or shPPIP5K2 #1 lentiviral vectors were cultured in flexible-bottom collagen-coated 6-well rubber Bioflex plates at an initial density of 1.4 × 10^5^/well treated with 0, 5, and 10 ng/mL of TGFβ1 in the presence or absence of 15% CMS (1 cycle/second, half-Sine mode, 24 hours) using a computer-controlled Flexcell FX-6000T Tension system. Cells plated on Bioflex plates under the same conditions but not subjected to stretch served as non-stretched controls. Cell lysates were collected after lysis with RIPA buffer and total protein concentrations were determined using the Pierce BCA Protein Assay Kit.

### Proteomic Profiling by Liquid Chromatography– Mass Spectrometry 

The extracted proteins (50 µg) from the 48 cell lysates of HCFs were used to determine the proteomic profiles using a label-free spectral counting method with MS, as previously described.^37^^–^^40^ Briefly, the precipitated protein lysate was re-dissolved in 40 µL 8M urea for overnight digestion using MS grade trypsin. Digested peptides were cleaned, lyophilized, and analyzed on the Orbitrap Fusion Tribrid MS coupled with an Ultimate 3000 nano-UPLC system. Raw MS data were processed via the Proteome Discoverer software (version 1.4) and submitted for SequestHT search against the SwissProt human database. A protein report containing the identities and number of PSM for each protein group was generated, which were further utilized for spectral counting-based semi-quantitative analysis. Proteins with low abundance and detections were filtered out from analysis (proteins detected in at least 12 samples with 1 PSM threshold were used for downstream analyses). Data were normalized with median ratios. Negative binomial models were fitted with covariates, including PPIP5K2, TGFβ1, and stretch, using the DESeq2 package. The FDR was controlled using the Benjamini-Hochberg method, and *P* values were adjusted accordingly. The global PPIP5K2 effect was determined by adjusting the impact of TGFβ1 and stretching conditions. Due to the discovery nature of this exploratory study, FDR-adjusted *P* values ≤ 0.1 were considered significant.^41^ To determine the over-represented biological processes, cellular components, molecular functions, and significant pathways, we uploaded the complete list of differentially expressed proteins (DEPs) to the PANTHER (Protein ANalysis THrough Evolutionary Relationships) classification system (FDR ≤ 0.05) for further analysis.^42^^,^^43^ The proteomics analysis was done with four biological replicates.

### Seahorse Real-Time ATP Rate Assay in Primary Human Stromal Fibroblasts

HCFs with PPIP5K2 knockdown (*n* = 4) or scrambled controls (*n* = 4) maintained in DMEM media were seeded at a density of 20,000 cells/well into Seahorse XFe96 cell culture plates (Agilent Technologies, Santa Clara, CA, USA; *n* = 8 wells per group for technical replicates) and incubated at 37°C with 5% CO_2_ and allowed 2 days to adhere. Before the assay, the culture media was replaced with XF DMEM Medium, pH 7.4 (Agilent #103575-100) supplemented with 200 mM L-glutamine (Agilent #103579-100), 1 M glucose (Agilent #103577-100), and 100 mM pyruvate (Agilent #103578-100) for 1 hour at 37°C, without CO_2_ to allow for pH and temperature calibration. Oxygen consumption rate (OCR) and extracellular acidification rate (ECAR) were analyzed using the Seahorse XF Real-Time ATP Rate Assay Kit (Agilent #103592-100) according to the manufacturer's protocol. In brief, cells were treated with a final concentration of 1.5 µM oligomycin (an inhibitor of mitochondrial ATP production) and 0.5 µM Rotenone + Antimycin A (a combination of complex I inhibitor and complex III inhibitor, respectively). Raw values for OCR and ECAR were normalized to total biomass by staining cellular proteins using CytoScan SRB Cytotoxicity Assay (G-Biosciences, St. Louis, MO, USA) according to the manufacturer's protocol to determine the percentage of ATP production from oxidative phosphorylation and glycolysis. Briefly, cells were fixed, washed, incubated in a 50°C incubator, stained with SRB Dye Solution, washed, incubated in a 50°C incubator, dissolved with SRB Solubilization Buffer, and absorbance measured at 565 nm using an Infinite M200 Pro plate reader (Tecan, Männedorf, Switzerland). The experiment was done with four biological replicates and eight technical replicates for each condition with each cell. For each cell, the mean ATP production rate for glycolysis and oxidative phosphorylation were derived from the technical replicates. We calculated the percentage of glycolysis and oxidative phosphorylation among the combined ATP production rate and compared the difference between HCFs with PPIP5K2 knockdown and those with scrambled controls. To analyze the impact of PPIP5K2 knockdown on ATP production rate via glycolysis or oxidative phosphorylation, we normalized the ATP production rates in HCFs with PPIP5K2 knockdown against those in HCFs without any transduction as a percentage change.

### Seahorse XF Cell Mito Stress Test in Primary Human Stromal Fibroblasts

HCF cells with PPIP5K2 knockdown (*n* = 4) or scramble controls (*n* = 4) were seeded and maintained as described in ATP Rate Assay in DMEM media and subsequent media change to XF DMEM with supplements. Afterward, OCR was analyzed using the Seahorse XF Cell Mito Stress Test kit (Agilent #103015-100). Briefly, cells were treated with a final concentration of 1.5 µM oligomycin, 2 µM Carbonyl cyanide p-trifluoro-methoxyphenyl hydrazone (FCCP), a mitochondrial membrane depolarizing agent, and 0.5 µM Rotenone + Antimycin A. Raw values for OCR were normalized to total biomass by staining cellular proteins with CytoScan SRB Cytotoxicity Assay to determine Basal Respiration, ATP-linked OCR, Maximal respiration, Spare Respiratory Capacity, and Proton Leak. The experiment was done with four biological replicates and eight technical replicates for each condition with each cell. To reduce the variations among different biological donor HCFs, we normalized the measurements in HCFs with PPIP5K2 knockdown to those HCFs with scrambled controls within each donor HCF cell.

### Seahorse XF Cell Glycolysis Stress Test in Primary Human Stromal Fibroblasts

HCF cells with PPIP5K2-knockdown (*n* = 4) or scrambled controls (*n* = 4) were seeded and maintained as protocol in ATP Rate Assay in DMEM media. Before the assay, the culture media was replaced with XF DMEM Medium, pH 7.4, supplemented with 200 mM L-glutamine for 1 hour at 37°C, without CO_2_, to allow for pH and temperature calibration. Afterward, ECAR was analyzed using the Seahorse XF Glycolysis Stress test kit (Agilent #103020-100) according to the manufacturer's protocol. Cells were treated with a final concentration of 10 mM glucose, 1 µM oligomycin, and 50 mM 2-Deoxy-D-glucose (2-DG). Raw values for ECAR were normalized to total biomass by staining cellular proteins using CytoScan SRB Cytotoxicity Assay, according to the manufacturer's protocol to determine glycolysis, glycolytic capacity, and glycolytic reserve. The experiment was done with four biological replicates and eight technical replicates for each condition with each cell. To reduce the variations among different biological donor HCFs, we normalized the measurements in HCFs with PPIP5K2 knockdown to those HCFs with scrambled controls within each donor HCF cell.

### Statistical Analysis

Statistical analyses were calculated using the Student's *t*-test and 1-way ANOVA with GraphPad Prism (GraphPad Software, San Diego, CA, USA) by comparing HCF-shPPIP5K2 #1 versus HCF-shControl or HCF-shPPIP5K2 #1 versus HCF-shControl versus HCF only. A *P* value ≤ 0.05 was considered statistically significant.

## Results

### shRNA-based PPIP5K2 Knockdown

**Figure 1.**
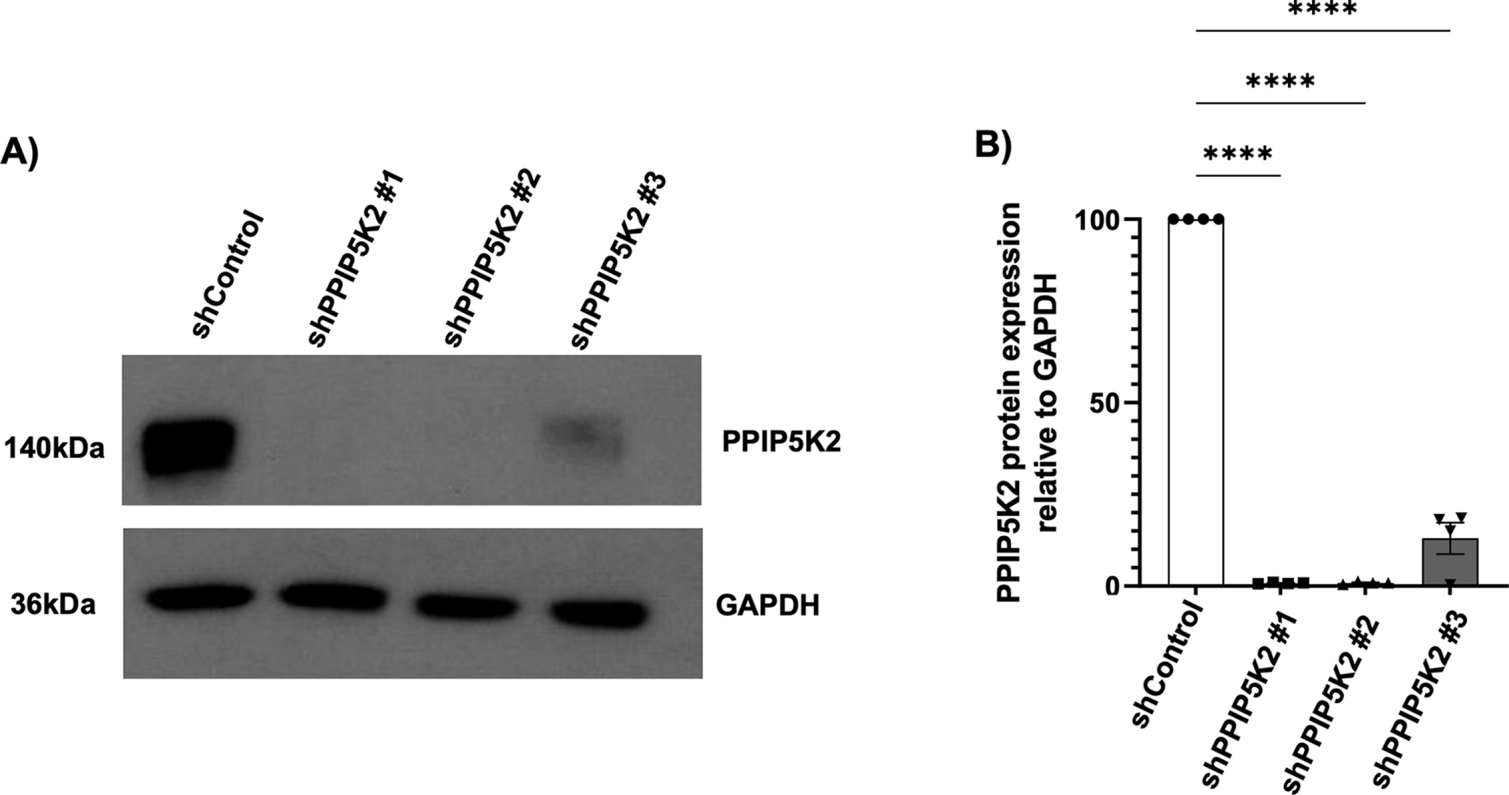
**PPIP5K2 knockdown in HCFs using three (3) different lentiviral particles containing three specific shRNAs against PPIP5K2.** (**A**) Western blot analyses of total protein lysates from HCFs expressing three independent shRNA for PPIP5K2 (shPPIP5K2 #1, shPPIP5K2 #2, and shPPIP5K2 #3) or the control (shControl). (**B**) Quantification of PPIP5K2 levels relative to the level of GAPDH. Note that subsequent data in all figures show shPPIP5K2 #1. All data represents the mean ± SEM of four independent experiments. *P* values were determined using 1-way ANOVA. **** *P* < 0.0001.

A previous study described how loss-of-function of PPIP5K2 alters energy metabolism in cancer cell lines.^16^ To pursue this finding, we analyzed the impact of stable knockdown of endogenous *PPIP5K2* in primary HCFs. We confirmed with immunoblotting analysis approximately 90% to 100% knockdown of PPIP5K2 with all shRNAs compared with scrambled controls (Figs. 1A, 1B). These transduced HCFs with PPIP5K2 knockdown or scrambled controls were included in the downstream proteomics experiment.

### Identification of Differentially Expressed Proteins

Our study included transduced healthy non-KC HCFs with shControl or shPPIP5K2 #1 (*n* = 4) treated with three different concentrations of TGFβ1 (0, 5, and 10 ng/mL) with or without CMS, leading to a total of 48 samples. A total of 1549 proteins were identified in all 48 samples (Supplementary Table S1A). With FDR ≤ 0.1, our DE multi-factorial analysis, with PPIP5K2 effect, TGFβ1 treatment, and CMS status as co-factors, identified 19 DEPs in HCFs lacking PPIP5K2 with TGFβ1 treatment and CMS status as cofactors compared with HCFs with shControl (Table 2; Supplementary Table S1B). It is noted that 15 proteins were down-regulated and only 4 proteins were upregulated upon PPIP5K2 knockdown compared with the shControl group. To increase the number of DEPs identified, using a *P* value < 0.05, we identified 110 DEPs in PPIP5K2 knockdown HCF with TGFβ1 treatment and CMS status as cofactors (Supplementary Table S1C), of which 74 were downregulated and 36 were upregulated upon PPIP5K2 knockdown.

**Table 2.** Nineteen Significant Differentially Expressed Proteins in PPIP5K2 Knockdown HCFs With TGFβ1 Treatment and CMS Status as Cofactors Compared With HCFs With shControl

Accession	Description	Gene Name	Base Mean	Fold Change	*P* Value	FDR-Adjusted *P* Value
P04179	Superoxide dismutase [Mn], mitochondrial	SOD2	3.8	−3.7	1.2E-09	1.2E-06
Q9Y6N5	Sulfide: quinone oxidoreductase, mitochondrial	SQOR	5.6	−2.5	2.2E-09	1.2E-06
P04406	Glyceraldehyde-3-phosphate dehydrogenase	GAPDH	294.8	−1.1	4.5E-09	1.7E-06
O00469	Procollagen-lysine, 2-oxoglutarate 5-dioxygenase 2	PLOD2	13.9	−1.6	8.6E-08	2.4E-05
P46821	Microtubule-associated protein 1B	MAP1B	69.5	−1.3	1.2E-07	2.6E-05
P04075	Fructose-bisphosphate aldolase A	ALDOA	88.6	−1.1	6.9E-06	1.3E-03
P17301	Integrin alpha-2	ITGA2	26.4	−1.4	4.6E-05	7.4E-03
P21980	Protein-glutamine gamma-glutamyltransferase 2	TGM2	2.2	−2.6	1.1E-04	1.6E-02
Q9NR30	Nucleolar RNA helicase 2	DDX21	10.7	1.5	1.4E-04	1.7E-02
P07237	Protein disulfide-isomerase	P4HB	91.0	−1.1	2.4E-04	2.7E-02
P00558	Phosphoglycerate Kinase 1	PGK1	85.3	−1.1	4.6E-04	4.7E-02
P00338	L-lactate dehydrogenase A chain	LDHA	68.7	−1.1	6.9E-04	6.1E-02
P14618	Pyruvate kinase PKM	PKM	287.6	−1.1	7.1E-04	6.1E-02
Q9HB71	Calcyclin-binding protein	CACYBP	3.0	1.9	8.3E-04	6.3E-02
P06899	Histone H2B type 1-J	H2BC11	15.0	1.4	8.3E-04	6.3E-02
Q14108	Lysosome membrane protein 2	SCARB2	6.0	−1.5	1.1E-03	7.8E-02
Q14914	Prostaglandin reductase 1	PTGR1	4.8	−1.9	1.3E-03	8.7E-02
Q06210	Glutamine-fructose-6-phosphate aminotransferase [isomerizing] 1	GFPT1	19.5	−1.2	1.6E-03	9.7E-02
P22626	Heterogeneous nuclear ribonucleoproteins A2/B1	HNRNPA2B1	60.0	1.1	1.7E-03	9.9E-02

FDR-adjusted *P* value ≤ 0.1.

**Figure 2.**
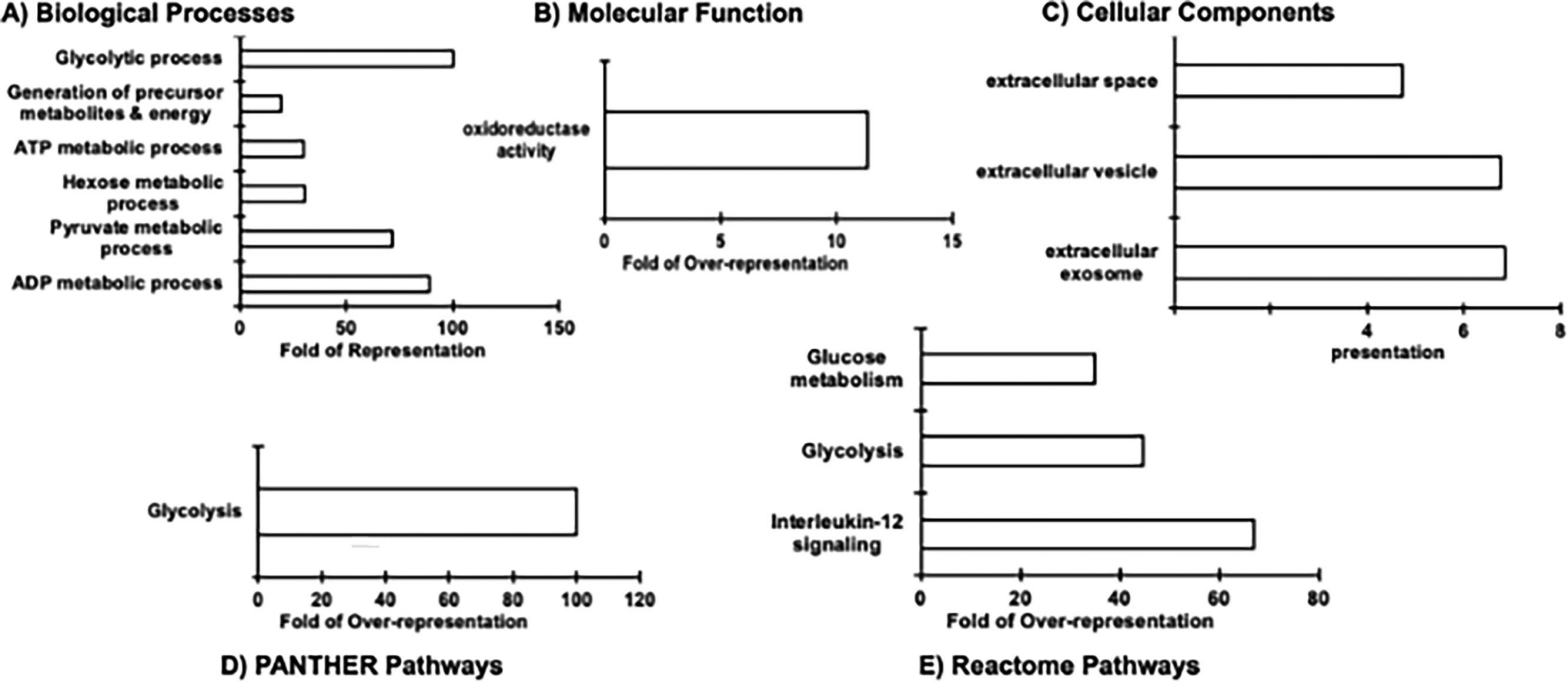
**Gene ontology terms.** Showing (**A**) biological processes, (**B**) molecular functions, and (**C**) cellular components, (**D**) PANTHER KEGG, and (**E**) Reactome pathway analyses of 19 significant differentially expressed proteins in PPIP5K2 knockdown HCF with TGFβ1 treatment and CMS status as cofactors (FDR ≤ 0.05).

We uploaded these 19 DEPs to the PANTHER tool online to identify the over-represented functional categories. Gene ontology analysis revealed that these proteins were over-represented in biological processes such as the glycolytic process, hexose metabolic process, pyruvate metabolic process, and ATP metabolic process (Fig. 2A; Supplementary Table S2A). In addition, these proteins were enriched in molecular functions such as oxidoreductase activity (Fig. 2B; Supplementary Table S2B). Furthermore, the proteins were localized in the extracellular exosomes and the extracellular space (Fig. 2C; Supplementary Table S2C). PANTHER Pathway and Reactome analyses also indicated the over-representation of proteins involved in glycolysis, glucose metabolism, and IL-12 signaling (Figs. 2D, 2E). All the identified proteins in glycolysis and glucose metabolism were downregulated, suggesting potential downregulation of glycolysis upon PPIP5K2 knockdown. Interestingly, all these processes overlapped with those of the enriched gene ontology categories using the 110 DEPs as the input proteins (Supplementary Figs. S2, S3). PANTHER Pathway and Reactome analyses using only the 74 downregulated proteins suggested the over-representation of proteins related to glycolysis, glucose metabolism, and cellular response to stress. PANTHER Gene Ontology analyses using the downregulated proteins indicated the over-representation of proteins related to NAD(P)H metabolism and activity, actin filament binding, cell adhesion, and actin cytoskeleton organization. PANTHER Pathway and Reactome analyses using only the 36 upregulated proteins indicated the over-representation of proteins involved in collagen formation/crosslinking and apoptosis. PANTHER Gene Ontology analyses using these upregulated 36 proteins revealed the over-representation of proteins involved in RNA binding, pyrophosphatase activity, and B-WICH complex, which is a chromatin remodeling complex to regulate histone H3 acetylation (H3K9).

Consistent with the role of PPIP5K2 in energy metabolism in the HCT116 cells and HEK293 cells,^16^ our proteomics study revealed that PPIP5K2 knockdown in HCFs modified the expression of proteins altered in glycolytic functions. We further determined whether these proteins were upregulated or downregulated in glycolysis after PPIP5K2 knockdown with TGFβ1 treatment and CMS status as cofactors. Interestingly, we observed a downregulation of several glycolytic proteins (see Table 2), namely Glyceraldehyde-3-phosphate dehydrogenase (GAPDH), Fructose-bisphosphate aldolase A (ALDOA), Phosphoglycerate kinase 1 (PGK1), L-lactate dehydrogenase A chain (LDHA), and Pyruvate kinase (PKM; Figs. 2A, 2D, 2E). Because glycolysis is one of the primary energy-producing pathways alongside oxidative phosphorylation, we focused on determining the energy profiles of HCFs after the knockdown of PPIP5K2 using seahorse assays.

### ATP Profiles in PPIP5K2 Knockdown HCFs

**Figure 3.**
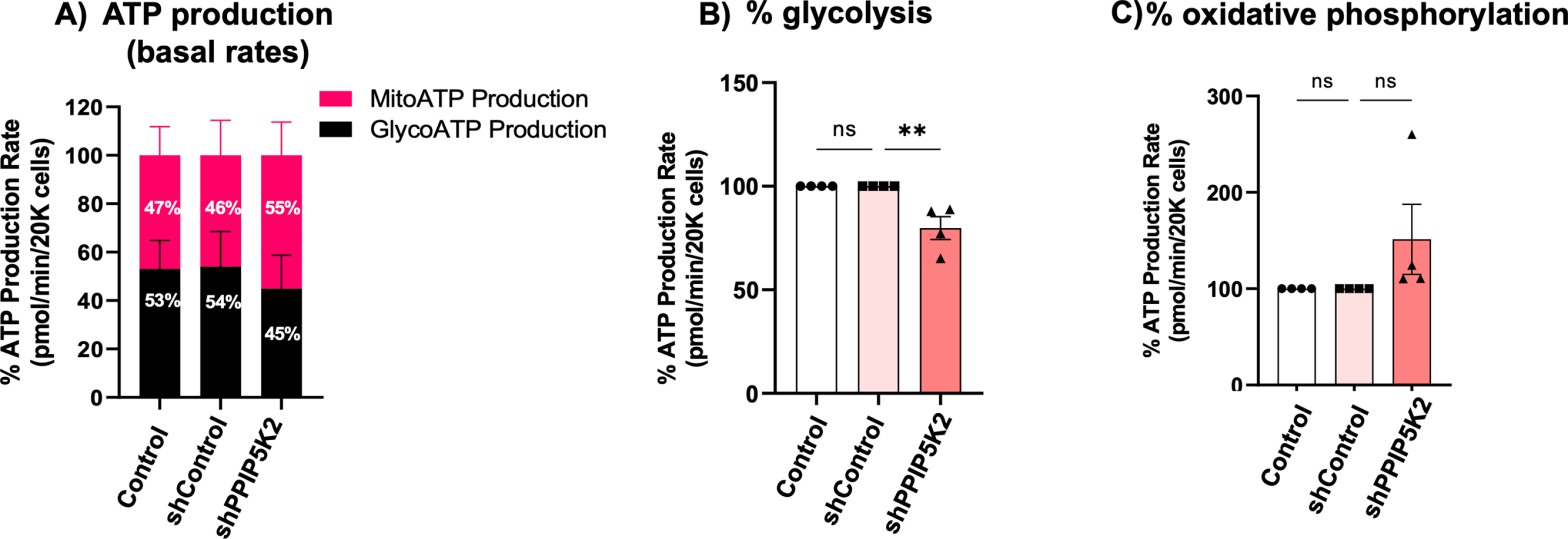
**PPIP5K2 knockdown in HCFs decreases the percentage of ATP production from glycolysis.** (**A**) Real-time ATP rate assay in primary human corneal stromal fibroblasts (*n* = 4 donors) with control (no transduction), shControl, and shPPIP5K2 #1. Approximately 54% and 46% of ATP were generated from glycolysis and oxidative phosphorylation, respectively, in the corneal stromal fibroblasts. A knockdown of PPIP5K2 slightly reduced ATP production from glycolysis to favor oxidative phosphorylation. (**B**) Quantification of ATP from glycolysis was normalized to the measurement in the group of controls, (**C**) quantification of ATP production from oxidative phosphorylation was normalized to the measurements in the group of controls. All values are mean ± SEM. ** *P* value < 0.003, ns: *P* > 0.05 using 1-way ANOVA.

To determine the metabolic profiles of HCFs following PPIP5K2 knockdown, first, ATP production was quantified through real-time measurements of the activity of two primary energy-producing pathways, glycolysis and mitochondrial oxidative phosphorylation. Approximately 93%, 52%, 48%, and 23% of ATP were generated from glycolysis from 4 donors, respectively (Supplementary Fig. S4). The ATP rate assay showed that approximately 54% of ATP was generated from glycolysis. All four donors showed different metabolic profiles. In comparison, approximately 46% of total ATP was produced by oxidative phosphorylation in stromal fibroblasts, suggesting that corneal stromal fibroblasts slightly favored the glycolytic pathway for energy generation (Figs. 3A–C). PPIP5K2 knockdown showed a trend of reduced glycolysis (see Fig. 3A–C) in the HCFs (matched Student’s *t*-test, *P* value < 0.003) while increasing ATP generation from oxidative phosphorylation (matched Student’s *t*-test, *P* value > 0.05; see Figs. 3A–C). We also observed variations in ATP production from glycolysis in the four donors after PPIP5K2 knockdown (82%, 40%, 43%, and 15%, respectively; see Supplementary Fig. S4). Despite the variations, the impact of knockdown was consistent as a percentage in all four donors.

### Mitochondrial Function in PPIP5K2 Knockdown HCFs

**Figure 4.**
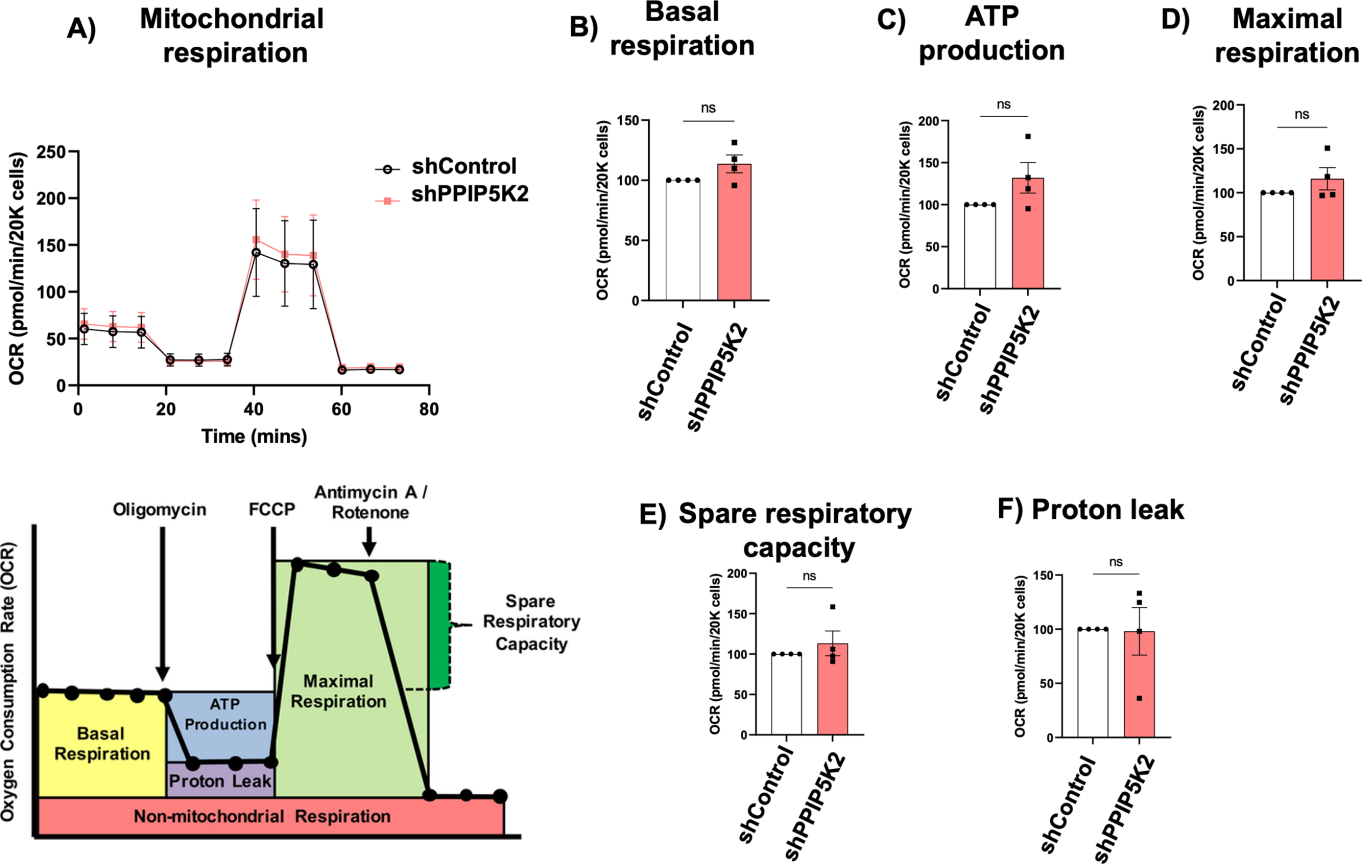
**Mito Stress Test to determine mitochondrial function shows increased mitochondrial bioenergetic capacity after PPIP5K2 knockdown in HCFs.** (**A**) OCR in primary human corneal stromal fibroblasts before and after PPIP5K2 knockdown (*upper panel*). Schematic of mitochondrial stress test (*lower panel*), under basal conditions followed by the sequential addition of oligomycin (1.5 µM), FCCP (2 µM), as well as rotenone and antimycin A (1 µM). Each data point represents an OCR measurement (**B**) individual parameter for basal respiration, (**C**) ATP Production, (**D**) Maximal Respiration, (**E**) Spare Respiratory Capacity, and (**F**) Proton Leak in the PPIP5K2 knockdown group in reference to the control groups (100%) in each biological donor HCF cells. Data are expressed as means ± SEM, *n* = 4 independent experiments with 8 technical replicates. Ns: *P* value < 0.05.

To determine functional changes in corneal cell mitochondria, we measured the OCR using PPIP5K2 silenced HCF cells. We observed that PPIP5K2 knockdown showed a trend of increased mitochondrial bioenergetic capacity in the stromal cells (*P* value > 0.05; Figs. 4A–F). We also recorded variabilities in the four donors (Supplementary Fig. S5). Two donor HCFs showed a trend of increased mitochondrial bioenergetic capacity, whereas the other two HCFs showed no changes (see Supplementary Fig. S5).

### Glycolytic Function in PPIP5K2 Knockdown HCFs

**Figure 5.**
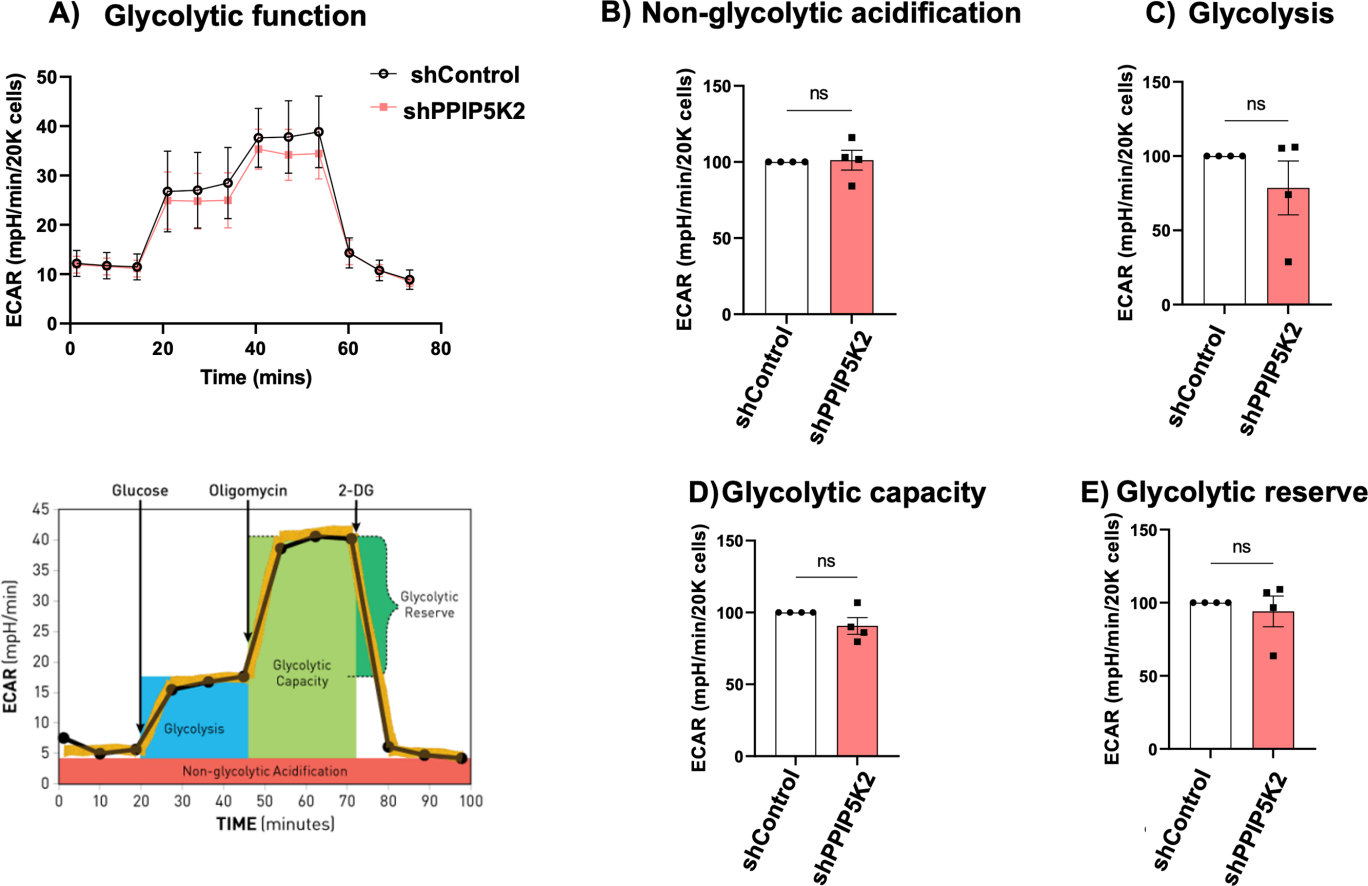
**Glycolysis Stress Test to determine glycolytic function shows decreased glycolytic profiles after PPIP5K2 knockdown in HCFs.** (**A**) ECAR in primary human corneal stromal fibroblasts before and after PPIP5K2 knockdown (*upper panel*). Schematic of glycolysis stress test (*lower panel*), under basal conditions followed by the sequential addition of glucose (10 mM), oligomycin (1 µM), as well as 2-Deoxy-D-glucose (2-DG; 50 mM). Each data point represents an ECAR measurement (**B**) individual parameters for non-glycolytic acidification, (**C**) Glycolysis, (**D**) Glycolytic Capacity, and (**E**) Glycolytic Reserve in the PPIP5K2 knockdown group in reference to the shControl group (100%) in each biological donor HCF cell. Data are expressed as means ± SEM, *n* = 4 independent experiments with 8 technical replicates. ns: *P* value < 0.05.

To determine glycolytic function changes in the corneal cells, we measured the ECAR using HCFs with PPIP5K2 knockdown or scrambled controls. We observed a trend of decreased glycolytic function in the HCFs after PPIP5K2 knockdown (*P* value > 0.05; Figs. 5A–E). As in the previous measurements, we observed similar variations in the four biological replicates where only two out of the four donors exhibited a trend of decreased glycolytic function (Supplementary Fig. S6).

## Discussion

In this study, we hypothesized that the lack of PPIP5K2 in HCFs may alter the expression of proteins involved in energy metabolism. Our analysis revealed DEPs over-represented in energy metabolism pathways, such as glycolysis, after the knockdown of PPIP5K2. It is necessary to integrate multiple risk factors of KC, including genetic, biomechanical (CMS), and molecular factors (TGFβ1 treatment), to model KC as a multifactorial disease. In addition, our findings revealed that HCFs slightly preferred the glycolytic pathway for energy generation, and the knockdown of PPIP5K2 decreased ATP generation from glycolysis and changed the metabolic profile of HCFs to favor the oxidative phosphorylation pathway for energy generation.

Inositol pyrophosphates (PP-InsPs) have been implicated in many human diseases, including cancer, hearing loss, and keratoconus.^44^^–^^46^ However, how PP-InPs alter proteome changes and regulate metabolic homeostasis in human corneas has not been elucidated. In our study, we identified two downregulated proteins that are of interest to the etiology of KC: superoxide dismutase 2 (SOD2) and procollagen-lysine, 2-oxoglutarate 5-dioxygenase 2 (PLOD2) after PPIP5K2 knockdown that may affect functional roles in the cornea or related to KC.^47^^–^^51^

In addition, our proteomic study also revealed the downregulation of some glycolytic proteins, such as GAPDH, ALDOA, PGK1, LDHA, and PKM, following the knockdown of PPIP5K2 in HCFs. All these proteins function as necessary enzymes of glycolysis.^52^^–^^55^ Interestingly, PPIP5K1 and PPIP5K2 have been shown to play roles in mitochondrial and glycolytic functions in other cell types but not in the cornea.^16^ Hence, we tested the impact of the knockdown of PPIP5K2 on energy dynamics in HCFs.

ATP is generated from two main energy pathways – glycolysis and mitochondrial respiration. Our findings suggest that HCFs mainly generate ATP through glycolysis. Interestingly, this observation confirms reports by Liang et al. after they compared the metabolic profiles of the human corneal epithelium and stroma.^56^ Because HCF utilizes ATP predominantly generated from glycolysis, loss of the PPIP5K2 phosphatase domain due to S419A and N843S mutations in KC may result in decreased ATP levels through decreased glycolysis in the KC cornea. Although we observed a trend of increase in oxidative phosphorylation, which could be a compensatory mechanism for generating ATP, it was not statistically significant.

To confirm the glycolytic/mitochondrial respiration imbalance, we used modulators of respiration into cell wells during the experiment to evaluate the OCR of our cells in real-time to identify the critical aspects of mitochondrial function. Mitochondrial spare respiratory capacity or reserve capacity is attained when cells have increased energy demand through high ATP demand to overcome stress, including oxidative stress.^57^^,^^58^ The KC cornea is susceptible to mitochondrial dysfunction and oxidative stress due to high oxygen tension and exposure to ultraviolet radiation.^47^^,^^59^^,^^60^ Cells with higher spare respiratory capacity may generate more ATP to withstand stress, such as oxidative stress.^57^ Our PPIP5K2 knockdown corneal fibroblasts showed a trend of increased spare respiratory capacity compared with the control cells. This suggests that these cells may have increased energy demand, contrary to what we expected. However, it is reflective of a study by Gu et al. where the authors observed a decrease in 1,5-InsP8 resulting in increased ATP levels owing to improved mitochondrial function after the knockout of PPIP5K1 and PPIP5K2 in the HCT116 and HEK293 cells.^16^

Extracellular acid is produced by cells because of the conversion of glucose to lactate in anaerobic conditions.^61^^,^^62^ Thus, we measured the ECAR to test glycolytic functions in the cells. Our results showed that the knockdown of PPIP5K2 in HCFs reduced glycolytic function, as evidenced by a trend of decrease in all the glycolytic parameters.

Many reasons could account for the non-significance in our mitochondrial and glycolytic studies. First, despite the similar donors’ ages, the HCFs from four donors exhibited variations in metabolic profiles. However, limited information on donor sex and ethnicity was only available from one of the four donors. In metabolic profiling studies, a significant issue is that factors other than genotypes, such as age, sex, ethnicity, lifestyle, environmental conditions, nutritional status, medication use, and other metabolites from symbiotic organisms (such as the gut microbiota) could contribute to changes in experimental studies.^52^^,^^53^^,^^63^^,^^64^ In the future, it will be necessary to design studies with more age/sex/ethnicity-matched donors while controlling other confounding factors.

Second, due to the lentiviral transduction and puromycin selection of our PPIP5K2 knockdown HCFs, we used cells between 7 and 10 passages, which could affect the cellular profile. To the best of our knowledge, many cell lines have been used to determine the involvement of PP-InPs in various processes that are specific to mammals.^16^^,^^65^^,^^66^ In primary cell culture, the behavior and morphology of cells could be affected by subsequent passages compared with cell lines.^54^^,^^55^ In addition, findings from cell lines may not be replicative in primary cells.^67^^,^^68^ Future studies with early passages of primary cells could mitigate these variations.

Third, PP-InPs in mammals have a rapid turnover and are generally in sub-micromolar to low micromolar ranges.^69^ In steady-state, total InsP7 (1-InsP7 and 5-InsP7) concentrations are between 1 and 2 µM, whereas the levels of 1,5-InsP8 are almost 10 times lower.^70^^–^^73^ Although different methods have been proposed to quantify these PP-InPs, it remains a technical challenge in the field.^69^^,^^74^ Currently, no studies have reported the levels of 5-Ins7 and 1,5-InsP8 in the corneas. From our results, it may be necessary to determine the actual changes of 1,5-InsP8 level after the knockdown of PPIP5K2 in our cells, which could help explain the effect of PPIP5K2 on the energy metabolic profiles we reported here.

In addition, the proteomics study identified GAPDH with significant reduction (−1.1-fold, 10% reduction) in HCFs with PPIP5K2 knockdown compared to those HCFs with scrambled controls. However, we used GAPDH as the reference in the Western blot to confirm the PPIP5K2 knockdown. Due to the limited sensitivity of Western blot, this relatively minor expression change was not noted. The validation of PPIP5K2 knockdown is still considered to be effective. In the future, other reference proteins and genes should be considered for KC studies.

## Conclusions

We determined the global protein expression in HCFs after the knockdown of PPIP5K2 underlying the treatment of TGFβ1 and CMS. We have shown that HCFs prefer the glycolytic pathway for energy generation, whereas the knockdown of PPIP5K2 decreased ATP generation from glycolysis to favor oxidative phosphorylation, although not statistically significant. This was supported by a test of glycolytic function, which showed a trend of decrease in glycolytic parameters, such as glycolysis, glycolytic capacity, and glycolytic reserve after the PPIP5K2 knockdown. Future studies could test the enzymatic activity of the three key enzymes of glycolysis: hexokinase, phosphofructokinase, and pyruvate kinase to buttress this claim. It is essential to determine the levels of 5-Ins7 and 1,5-InsP8 in HCFs with the knockdown of PPIP5K2 levels to understand this glycolytic/oxidative phosphorylation paradox.

## Supplementary Material

Supplement 1
