# Supplementary material for: Proteomic Analysis Provides Insights Into PPIP5K2 Function and Its Impact on Corneal Energy Metabolism
Source: Invest Ophthalmol Vis Sci. 2025 Dec 23;66(15):67. doi: 10.1167/iovs.66.15.67 (PMC12743493; doi:10.1167/iovs.66.15.67)
Supplement: Supplement 1 [file iovs-66-15-67_s001.docx]

**Proteomic Analysis Provides Insights into PPIP5K2 function and its Impact on Corneal Energy Metabolism**

Theresa Dansu^1^, Caili Hao^1^, Zhong Chen^1^, Xiaowen Lu^1^, Hongfang Yu^1^, Chunfang Gu^2^, Stephen B. Shears^2^, Wenbo Zhi^3^, Xingjun Fan^1,4^, Mitchell A. Watsky^1,4^, Yutao Liu^1,3,4*^

^1^ Department of Cellular Biology and Anatomy, Medical College of Georgia, Augusta University, Augusta, Georgia, United States

^2^ Inositol Signaling Group, Signal Transduction Laboratory, National Institute of Environmental Health Sciences, Research Triangle Park, North Carolina, United States

^3^ Center for Biotechnology and Genomic Medicine, Medical College of Georgia, Augusta University, Augusta, Georgia, United States

^4^ James & Jean Culver Vision Discovery Institute, Medical College of Georgia, Augusta University, Augusta, Georgia, United States

**Supplementary files**

**Supplementary Table S1A.** Total number of proteins identified in the forty-eight samples in the PPIP5K2-knockdown HCF cells with TGFβ1 treatment and CMS status as cofactors, compared to those HCF cells without PPIP5K2 knockdown.

| Accession | Description | Gene Name | Species | baseMean | log2 Fold Change | lfcSE | stat | p-value | FDR-adjusted p-value |
| --- | --- | --- | --- | --- | --- | --- | --- | --- | --- |
| P04179 | Superoxide dismutase [Mn], mitochondrial | SOD2 | Homo sapiens | 3.827286543 | -1.886837703 | 0.310384606 | -6.0790312 | 1.21E-09 | 1.23E-06 |
| Q9Y6N5 | Sulfide:quinone oxidoreductase, mitochondrial | SQOR | Homo sapiens | 5.648694408 | -1.319805421 | 0.220572322 | -5.983549568 | 2.18E-09 | 1.23E-06 |
| P04406 | Glyceraldehyde-3-phosphate dehydrogenase | GAPDH | Homo sapiens | 294.8185616 | -0.23065628 | 0.039331881 | -5.864359301 | 4.51E-09 | 1.70E-06 |
| O00469 | Procollagen-lysine,2-oxoglutarate 5-dioxygenase 2 | PLOD2 | Homo sapiens | 13.85104678 | -0.686098907 | 0.128123114 | -5.354997115 | 8.56E-08 | 2.41E-05 |
| P46821 | Microtubule-associated protein 1B | MAP1B | Homo sapiens | 69.45629736 | -0.371656664 | 0.070141646 | -5.298658984 | 1.17E-07 | 2.63E-05 |
| P04075 | Fructose-bisphosphate aldolase A | ALDOA | Homo sapiens | 88.55034928 | -0.231730659 | 0.051535287 | -4.496543446 | 6.91E-06 | 0.001298461 |
| P17301 | Integrin alpha-2 | ITGA2 | Homo sapiens | 26.41743162 | -0.542608163 | 0.133116958 | -4.076176115 | 4.58E-05 | 0.007377499 |
| P21980 | Protein-glutamine gamma-glutamyltransferase 2 | TGM2 | Homo sapiens | 2.18265185 | -1.380109681 | 0.357460667 | -3.860871438 | 0.000112983 | 0.015930651 |
| Q9NR30 | Nucleolar RNA helicase 2 | DDX21 | Homo sapiens | 10.70539072 | 0.550656977 | 0.144538983 | 3.809747139 | 0.000139109 | 0.017434994 |
| P07237 | Protein disulfide-isomerase | P4HB | Homo sapiens | 91.00120091 | -0.195123337 | 0.053177005 | -3.66931789 | 0.000243199 | 0.027432792 |
| P00558 | Phosphoglycerate kinase 1 | PGK1 | Homo sapiens | 85.30931736 | -0.172743628 | 0.049327774 | -3.501954664 | 0.000461858 | 0.047361459 |
| P00338 | L-lactate dehydrogenase A chain | LDHA | Homo sapiens | 68.73577007 | -0.186289287 | 0.054866995 | -3.395288688 | 0.000685563 | 0.061216691 |
| P14618 | Pyruvate kinase PKM | PKM | Homo sapiens | 287.6436456 | -0.104401959 | 0.030820419 | -3.387428317 | 0.000705512 | 0.061216691 |
| Q9HB71 | Calcyclin-binding protein | CACYBP | Homo sapiens | 2.968795966 | 0.939121994 | 0.281049726 | 3.341479844 | 0.000833331 | 0.062695408 |
| P06899 | Histone H2B type 1-J | H2BC11 | Homo sapiens | 14.98095805 | 0.506106703 | 0.151467656 | 3.341351661 | 0.000833716 | 0.062695408 |
| Q14108 | Lysosome membrane protein 2 | SCARB2 | Homo sapiens | 5.955731073 | -0.603194204 | 0.184949856 | -3.261393206 | 0.001108662 | 0.07816066 |
| Q14914 | Prostaglandin reductase 1 | PTGR1 | Homo sapiens | 4.84516069 | -0.895266713 | 0.278611765 | -3.213312663 | 0.001312134 | 0.087063927 |
| Q06210 | Glutamine--fructose-6-phosphate aminotransferase [isomerizing] 1 | GFPT1 | Homo sapiens | 19.53188985 | -0.330994726 | 0.104584611 | -3.164851134 | 0.001551623 | 0.097235051 |
| P22626 | Heterogeneous nuclear ribonucleoproteins A2/B1 | HNRNPA2B1 | Homo sapiens | 59.97775342 | 0.174551162 | 0.055517802 | 3.144057529 | 0.001666227 | 0.098921254 |
| Q8IUE6 | Histone H2A type 2-B | H2AC21 | Homo sapiens | 4.051539189 | 0.812759486 | 0.260170001 | 3.123955422 | 0.001784375 | 0.100638743 |
| P42166 | Lamina-associated polypeptide 2, isoform alpha | TMPO | Homo sapiens | 4.639938645 | 0.647277133 | 0.214313721 | 3.020231881 | 0.002525812 | 0.135672203 |
| P13010 | X-ray repair cross-complementing protein 5 | XRCC5 | Homo sapiens | 20.56463212 | 0.301585326 | 0.10092833 | 2.988113699 | 0.002807051 | 0.140328462 |
| P20700 | Lamin-B1 | LMNB1 | Homo sapiens | 7.832015185 | 0.472584144 | 0.158634462 | 2.979076163 | 0.002891189 | 0.140328462 |
| P35754 | Glutaredoxin-1 | GLRX | Homo sapiens | 2.696621426 | -1.168217097 | 0.393444386 | -2.969205146 | 0.002985712 | 0.140328462 |
| P43490 | Nicotinamide phosphoribosyltransferase | NAMPT | Homo sapiens | 3.902009212 | -0.671509156 | 0.229872563 | -2.921223606 | 0.003486595 | 0.151445869 |
| Q9Y2D5 | A-kinase anchor protein 2 | AKAP2 | Homo sapiens | 4.304103459 | -0.662266175 | 0.226827726 | -2.919687943 | 0.00350382 | 0.151445869 |
| Q8IVF2 | Protein AHNAK2 | AHNAK2 | Homo sapiens | 1.871858894 | 1.093729516 | 0.376514373 | 2.904881182 | 0.003673925 | 0.151445869 |
| Q15149 | Plectin | PLEC | Homo sapiens | 159.8969962 | 0.216292708 | 0.074890077 | 2.888135757 | 0.003875326 | 0.151445869 |
| P15144 | Aminopeptidase N | ANPEP | Homo sapiens | 10.29794596 | -0.464367516 | 0.160866752 | -2.886659362 | 0.003893555 | 0.151445869 |
| P48681 | Nestin | NES | Homo sapiens | 4.007349196 | 0.709975597 | 0.253020666 | 2.805998452 | 0.005016093 | 0.182552134 |
| Q15437 | Protein transport protein Sec23B | SEC23B | Homo sapiens | 6.973120505 | -0.461882962 | 0.165100729 | -2.79758281 | 0.005148657 | 0.182552134 |
| Q16222 | UDP-N-acetylhexosamine pyrophosphorylase | UAP1 | Homo sapiens | 3.446334954 | 0.704260576 | 0.251908714 | 2.795697556 | 0.005178784 | 0.182552134 |
| Q96D15 | Reticulocalbin-3 | RCN3 | Homo sapiens | 5.95830039 | -0.591400936 | 0.213208233 | -2.773818482 | 0.005540258 | 0.189359649 |
| Q96T76 | MMS19 nucleotide excision repair protein homolog | MMS19 | Homo sapiens | 1.997705387 | 0.939064621 | 0.339733628 | 2.764120312 | 0.005707649 | 0.189359649 |
| P09936 | Ubiquitin carboxyl-terminal hydrolase isozyme L1 | UCHL1 | Homo sapiens | 17.1484001 | -0.298739925 | 0.109036566 | -2.739814133 | 0.006147394 | 0.193079897 |
| A5A3E0 | POTE ankyrin domain family member F | POTEF | Homo sapiens | 81.36538604 | -0.151822882 | 0.055429487 | -2.739027368 | 0.006162124 | 0.193079897 |
| P39748 | Flap endonuclease 1 | FEN1 | Homo sapiens | 0.871322361 | 1.343890965 | 0.491867688 | 2.732220465 | 0.006290903 | NA |
| P01889 | HLA class I histocompatibility antigen, B alpha chain | HLA-B | Homo sapiens | 3.654785272 | -0.63398274 | 0.238066839 | -2.663045143 | 0.007743702 | 0.235626498 |
| P09493 | Tropomyosin alpha-1 chain | TPM1 | Homo sapiens | 5.222368099 | -0.656059642 | 0.247130874 | -2.654705302 | 0.007937772 | 0.235626498 |
| P11277 | Spectrin beta chain, erythrocytic | SPTB | Homo sapiens | 0.880603621 | -1.295630368 | 0.489089585 | -2.649065548 | 0.008071468 | NA |
| P53396 | ATP-citrate synthase | ACLY | Homo sapiens | 62.79748371 | -0.150855199 | 0.057985327 | -2.601609846 | 0.009278734 | 0.260618167 |
| P21796 | Voltage-dependent anion-selective channel protein 1 | VDAC1 | Homo sapiens | 23.03881725 | -0.348796934 | 0.134402078 | -2.595175164 | 0.009454272 | 0.260618167 |
| Q9BQG0 | Myb-binding protein 1A | MYBBP1A | Homo sapiens | 2.841275812 | 0.688510429 | 0.265689357 | 2.591411399 | 0.009558315 | 0.260618167 |
| Q14315 | Filamin-C | FLNC | Homo sapiens | 146.122025 | -0.168014843 | 0.065108438 | -2.580538707 | 0.00986463 | 0.260618167 |
| P52895 | Aldo-keto reductase family 1 member C2 | AKR1C2 | Homo sapiens | 5.105652422 | -0.740025842 | 0.287044613 | -2.578086503 | 0.009934912 | 0.260618167 |
| P08133 | Annexin A6 | ANXA6 | Homo sapiens | 19.9749562 | -0.373144763 | 0.148251551 | -2.51697038 | 0.011836878 | 0.303454508 |
| Q15436 | Protein transport protein Sec23A | SEC23A | Homo sapiens | 18.23814561 | -0.26742296 | 0.106777652 | -2.50448437 | 0.012263002 | 0.307392592 |
| P10599 | Thioredoxin | TXN | Homo sapiens | 5.294389051 | -0.517498896 | 0.207931528 | -2.488794749 | 0.012817694 | 0.308881399 |
| Q04828 | Aldo-keto reductase family 1 member C1 | AKR1C1 | Homo sapiens | 3.750335207 | -0.666499242 | 0.267956108 | -2.487344833 | 0.012870058 | 0.308881399 |
| Q9NY65 | Tubulin alpha-8 chain | TUBA8 | Homo sapiens | 49.4652756 | -0.217228701 | 0.087623861 | -2.47910442 | 0.013171274 | 0.309524939 |
| P18621 | 60S ribosomal protein L17 | RPL17 | Homo sapiens | 12.61527373 | 0.297990557 | 0.121201767 | 2.458632119 | 0.013946745 | 0.310417804 |
| P28300 | Protein-lysine 6-oxidase | LOX | Homo sapiens | 2.69737661 | 0.862817149 | 0.352375902 | 2.448570248 | 0.014342446 | 0.310417804 |
| P05121 | Plasminogen activator inhibitor 1 | SERPINE1 | Homo sapiens | 8.958393289 | -0.439881335 | 0.181142754 | -2.428368378 | 0.015166929 | 0.310417804 |
| Q96CX2 | BTB/POZ domain-containing protein KCTD12 | KCTD12 | Homo sapiens | 15.77576205 | -0.285381023 | 0.11775604 | -2.423493724 | 0.015372019 | 0.310417804 |
| Q969X5 | Endoplasmic reticulum-Golgi intermediate compartment protein 1 | ERGIC1 | Homo sapiens | 1.598268765 | -0.899228714 | 0.372053822 | -2.416931797 | 0.01565195 | NA |
| Q70UQ0 | Inhibitor of nuclear factor kappa-B kinase-interacting protein | IKBIP | Homo sapiens | 4.055260659 | -0.662059502 | 0.274041375 | -2.41591074 | 0.015695909 | 0.310417804 |
| Q6AWC2 | Protein WWC2 | WWC2 | Homo sapiens | 0.659881434 | 1.315819193 | 0.545278305 | 2.41311488 | 0.015816833 | NA |
| Q9UPN3 | Microtubule-actin cross-linking factor 1, isoforms 1/2/3/5 | MACF1 | Homo sapiens | 3.741430952 | -0.575892073 | 0.238968146 | -2.409911452 | 0.015956393 | 0.310417804 |
| P04083 | Annexin A1 | ANXA1 | Homo sapiens | 33.05532248 | -0.191715408 | 0.07972283 | -2.404774255 | 0.016182459 | 0.310417804 |
| P30041 | Peroxiredoxin-6 | PRDX6 | Homo sapiens | 13.09802476 | -0.324860253 | 0.135290498 | -2.401205244 | 0.016341168 | 0.310417804 |
| P16401 | Histone H1.5 | H1-5 | Homo sapiens | 14.47277004 | 0.279193463 | 0.116297146 | 2.400690577 | 0.016364167 | 0.310417804 |
| Q9HDC9 | Adipocyte plasma membrane-associated protein | APMAP | Homo sapiens | 5.839341822 | -0.456241866 | 0.190200289 | -2.398744329 | 0.016451397 | 0.310417804 |
| P12814 | Alpha-actinin-1 | ACTN1 | Homo sapiens | 108.9410106 | -0.131775582 | 0.05493823 | -2.398613537 | 0.016457274 | 0.310417804 |
| P14625 | Endoplasmin | HSP90B1 | Homo sapiens | 99.2792623 | -0.114662003 | 0.047827514 | -2.397406701 | 0.016511585 | 0.310417804 |
| O94808 | Glutamine--fructose-6-phosphate aminotransferase [isomerizing] 2 | GFPT2 | Homo sapiens | 7.5587698 | -0.480011686 | 0.201442305 | -2.38287427 | 0.01717806 | 0.31765331 |
| Q9BQ39 | ATP-dependent RNA helicase DDX50 | DDX50 | Homo sapiens | 0.751751344 | 1.21676203 | 0.512619892 | 2.373614542 | 0.017614929 | NA |
| P07602 | Prosaposin | PSAP | Homo sapiens | 8.095391184 | -0.41383002 | 0.17480517 | -2.367378612 | 0.017914596 | 0.325930064 |
| P60174 | Triosephosphate isomerase | TPI1 | Homo sapiens | 52.90565514 | -0.145678098 | 0.061956304 | -2.351303876 | 0.018707749 | 0.334957789 |
| Q14956 | Transmembrane glycoprotein NMB | GPNMB | Homo sapiens | 1.709939151 | -0.826863943 | 0.352582871 | -2.34516198 | 0.019018815 | NA |
| Q16881 | Thioredoxin reductase 1, cytoplasmic | TXNRD1 | Homo sapiens | 28.80939167 | -0.203379811 | 0.087553118 | -2.322930534 | 0.020182885 | 0.35572334 |
| Q03405 | Urokinase plasminogen activator surface receptor | PLAUR | Homo sapiens | 1.94364584 | -0.834969807 | 0.363324621 | -2.298137147 | 0.021553985 | 0.374044533 |
| P84996 | Protein ALEX | GNAS | Homo sapiens | 1.44870162 | 1.070660627 | 0.468339685 | 2.286077099 | 0.022249749 | NA |
| P15559 | NAD(P)H dehydrogenase [quinone] 1 | NQO1 | Homo sapiens | 14.17011403 | -0.289050923 | 0.126818599 | -2.279247093 | 0.022652381 | 0.387149787 |
| P18669 | Phosphoglycerate mutase 1 | PGAM1 | Homo sapiens | 13.96273978 | -0.448270508 | 0.197369593 | -2.271223752 | 0.023133436 | 0.388821474 |
| Q8WWM7 | Ataxin-2-like protein | ATXN2L | Homo sapiens | 1.983556751 | 0.719263178 | 0.317388369 | 2.266192619 | 0.023439592 | 0.388821474 |
| Q14683 | Structural maintenance of chromosomes protein 1A | SMC1A | Homo sapiens | 1.293707112 | 0.997141635 | 0.440033081 | 2.266060616 | 0.023447672 | NA |
| P42167 | Lamina-associated polypeptide 2, isoforms beta/gamma | TMPO | Homo sapiens | 6.396704429 | 0.394580259 | 0.175977817 | 2.242215901 | 0.024947422 | 0.407220105 |
| P27797 | Calreticulin | CALR | Homo sapiens | 56.35528988 | -0.1392784 | 0.062254603 | -2.237238578 | 0.025270751 | 0.407220105 |
| P33316 | Deoxyuridine 5'-triphosphate nucleotidohydrolase, mitochondrial | DUT | Homo sapiens | 0.832407744 | 1.110857038 | 0.49673685 | 2.2363089 | 0.025331544 | NA |
| O15355 | Protein phosphatase 1G | PPM1G | Homo sapiens | 0.983470092 | 1.152206393 | 0.518427409 | 2.222502849 | 0.026249342 | NA |
| P62495 | Eukaryotic peptide chain release factor subunit 1 | ETF1 | Homo sapiens | 8.271750928 | 0.335067545 | 0.150793531 | 2.222028643 | 0.02628137 | 0.416229462 |
| Q9HC07 | Transmembrane protein 165 | TMEM165 | Homo sapiens | 3.944478001 | -0.503918993 | 0.227214754 | -2.217809296 | 0.026567838 | 0.416229462 |
| Q8NEY1 | Neuron navigator 1 | NAV1 | Homo sapiens | 0.544595461 | 1.25272774 | 0.566623442 | 2.210864654 | 0.027045211 | NA |
| P17655 | Calpain-2 catalytic subunit | CAPN2 | Homo sapiens | 40.88738806 | -0.155019439 | 0.071187026 | -2.177636104 | 0.029433139 | 0.454802483 |
| Q6PIU2 | Neutral cholesterol ester hydrolase 1 | NCEH1 | Homo sapiens | 2.99170878 | -0.564445131 | 0.261286958 | -2.16024992 | 0.030753328 | 0.468780452 |
| P67936 | Tropomyosin alpha-4 chain | TPM4 | Homo sapiens | 12.39741629 | -0.340442636 | 0.158554072 | -2.147170554 | 0.031779706 | 0.474019574 |
| P08123 | Collagen alpha-2(I) chain | COL1A2 | Homo sapiens | 12.13820933 | 0.322103857 | 0.150151519 | 2.145192124 | 0.031937489 | 0.474019574 |
| O43660 | Pleiotropic regulator 1 | PLRG1 | Homo sapiens | 1.153430181 | 0.86229234 | 0.403404829 | 2.137535989 | 0.032554422 | NA |
| Q9H2G2 | STE20-like serine/threonine-protein kinase | SLK | Homo sapiens | 1.001954295 | 0.971739317 | 0.455015636 | 2.135617417 | 0.032710611 | NA |
| Q9Y3A5 | Ribosome maturation protein SBDS | SBDS | Homo sapiens | 0.760646687 | -1.148781215 | 0.542814682 | -2.116341458 | 0.034315777 | NA |
| P13797 | Plastin-3 | PLS3 | Homo sapiens | 21.07765008 | -0.212485429 | 0.100533728 | -2.11357357 | 0.034551705 | 0.506160046 |
| P13674 | Prolyl 4-hydroxylase subunit alpha-1 | P4HA1 | Homo sapiens | 11.83303278 | -0.323590127 | 0.154069079 | -2.100292474 | 0.035703121 | 0.514253409 |
| Q9BRF8 | Serine/threonine-protein phosphatase CPPED1 | CPPED1 | Homo sapiens | 0.641171218 | -1.293979273 | 0.616129651 | -2.100173675 | 0.035713566 | NA |
| Q53GQ0 | Very-long-chain 3-oxoacyl-CoA reductase | HSD17B12 | Homo sapiens | 1.835043027 | -0.703513998 | 0.336093084 | -2.093211764 | 0.036330255 | 0.514253409 |
| O75534 | Cold shock domain-containing protein E1 | CSDE1 | Homo sapiens | 4.236853426 | 0.444053825 | 0.21230065 | 2.091627249 | 0.036471873 | 0.514253409 |
| O15118 | NPC intracellular cholesterol transporter 1 | NPC1 | Homo sapiens | 1.499547945 | -0.805365565 | 0.386396305 | -2.084299345 | 0.037132947 | NA |
| Q9Y5S1 | Transient receptor potential cation channel subfamily V member 2 | TRPV2 | Homo sapiens | 1.122621661 | -0.946404991 | 0.456157059 | -2.074734944 | 0.038011107 | NA |
| P01033 | Metalloproteinase inhibitor 1 | TIMP1 | Homo sapiens | 1.151126581 | -0.915980241 | 0.443222546 | -2.066637291 | 0.038768344 | NA |
| P00505 | Aspartate aminotransferase, mitochondrial | GOT2 | Homo sapiens | 4.847586436 | -0.398946535 | 0.194477083 | -2.051380695 | 0.040229888 | 0.557204715 |
| P36578 | 60S ribosomal protein L4 | RPL4 | Homo sapiens | 38.83111079 | 0.142987001 | 0.069799084 | 2.048551242 | 0.040506017 | 0.557204715 |
| P0DME0 | Protein SETSIP | SETSIP | Homo sapiens | 1.367600944 | -0.847491573 | 0.415144525 | -2.041437434 | 0.041207367 | NA |
| P69905 | Hemoglobin subunit alpha | HBA1 | Homo sapiens | 47.59033187 | -0.15484732 | 0.076098159 | -2.034836629 | 0.041867314 | 0.568656347 |
| P09429 | High mobility group protein B1 | HMGB1 | Homo sapiens | 6.437889827 | 0.361753284 | 0.178223829 | 2.029769447 | 0.04237998 | 0.568656347 |
| P32119 | Peroxiredoxin-2 | PRDX2 | Homo sapiens | 13.85720049 | -0.239986301 | 0.118502356 | -2.025160588 | 0.042850877 | 0.568656347 |
| Q8NG11 | Tetraspanin-14 | TSPAN14 | Homo sapiens | 2.031371434 | -0.664244213 | 0.330855706 | -2.007655303 | 0.044679933 | 0.586034475 |
| P08727 | Keratin, type I cytoskeletal 19 | KRT19 | Homo sapiens | 3.044482107 | 0.565141589 | 0.283666498 | 1.992274704 | 0.046340926 | 0.60083407 |
| P30101 | Protein disulfide-isomerase A3 | PDIA3 | Homo sapiens | 56.53702673 | -0.116999055 | 0.058896449 | -1.986521364 | 0.046975463 | 0.602140026 |
| Q9NYU2 | UDP-glucose:glycoprotein glucosyltransferase 1 | UGGT1 | Homo sapiens | 5.28287694 | -0.412255674 | 0.208103089 | -1.981016598 | 0.047589412 | 0.603155697 |
| Q15417 | Calponin-3 | CNN3 | Homo sapiens | 21.15203789 | -0.195060459 | 0.09878538 | -1.97458833 | 0.048314884 | 0.605546552 |
| Q15113 | Procollagen C-endopeptidase enhancer 1 | PCOLCE | Homo sapiens | 1.023640762 | 0.884455903 | 0.450858043 | 1.961717032 | 0.049795438 | NA |
| P49411 | Elongation factor Tu, mitochondrial | TUFM | Homo sapiens | 6.661774849 | 0.343004404 | 0.177085398 | 1.936943462 | 0.052752255 | 0.637626878 |
| Q14166 | Tubulin--tyrosine ligase-like protein 12 | TTLL12 | Homo sapiens | 0.720375935 | 1.011778849 | 0.523530194 | 1.932608397 | 0.053284457 | NA |
| P47895 | Aldehyde dehydrogenase family 1 member A3 | ALDH1A3 | Homo sapiens | 3.262213205 | -0.567305024 | 0.293804054 | -1.930895834 | 0.053495936 | 0.637626878 |
| O43707 | Alpha-actinin-4 | ACTN4 | Homo sapiens | 86.53293306 | -0.117208793 | 0.060794766 | -1.927942178 | 0.053862319 | 0.637626878 |
| Q969H8 | Myeloid-derived growth factor | MYDGF | Homo sapiens | 4.104343883 | -0.430251382 | 0.223187058 | -1.927761342 | 0.053884818 | 0.637626878 |
| Q562R1 | Beta-actin-like protein 2 | ACTBL2 | Homo sapiens | 97.57743906 | -0.0871501 | 0.045468755 | -1.916703014 | 0.055275672 | 0.637626878 |
| P06744 | Glucose-6-phosphate isomerase | GPI | Homo sapiens | 26.30225408 | -0.173228212 | 0.09062837 | -1.911412646 | 0.055951573 | 0.637626878 |
| O75643 | U5 small nuclear ribonucleoprotein 200 kDa helicase | SNRNP200 | Homo sapiens | 15.22881366 | 0.214206577 | 0.112161867 | 1.909798607 | 0.056159149 | 0.637626878 |
| Q16643 | Drebrin | DBN1 | Homo sapiens | 17.52728889 | -0.211270003 | 0.110631543 | -1.909672392 | 0.056175408 | 0.637626878 |
| P40189 | Interleukin-6 receptor subunit beta | IL6ST | Homo sapiens | 1.837003203 | -0.743635906 | 0.390707311 | -1.903306863 | 0.057000516 | 0.637626878 |
| O60814 | Histone H2B type 1-K | H2BC12 | Homo sapiens | 76.69308562 | 0.145143441 | 0.076441928 | 1.898741227 | 0.057598508 | 0.637626878 |
| P63104 | 14-3-3 protein zeta/delta | YWHAZ | Homo sapiens | 42.91290757 | -0.124603038 | 0.065785205 | -1.894089063 | 0.058213189 | 0.637626878 |
| Q9UGI8 | Testin | TES | Homo sapiens | 1.293908218 | 0.726137381 | 0.38367808 | 1.892569364 | 0.058415161 | NA |
| Q96N66 | Lysophospholipid acyltransferase 7 | MBOAT7 | Homo sapiens | 2.971079654 | -0.489917173 | 0.258968766 | -1.891800236 | 0.058517602 | 0.637626878 |
| O43852 | Calumenin | CALU | Homo sapiens | 19.75257444 | -0.221217759 | 0.117024332 | -1.890356937 | 0.058710239 | 0.637626878 |
| Q13435 | Splicing factor 3B subunit 2 | SF3B2 | Homo sapiens | 4.552581981 | 0.383953677 | 0.203400455 | 1.887673637 | 0.059069778 | 0.637626878 |
| P23284 | Peptidyl-prolyl cis-trans isomerase B | PPIB | Homo sapiens | 26.69176639 | -0.171420233 | 0.090924801 | -1.88529676 | 0.059389783 | 0.637626878 |
| P12110 | Collagen alpha-2(VI) chain | COL6A2 | Homo sapiens | 6.374331025 | -0.336311793 | 0.178757052 | -1.881390355 | 0.059918838 | 0.637626878 |
| Q9Y5S9 | RNA-binding protein 8A | RBM8A | Homo sapiens | 1.210417026 | 0.76411412 | 0.409296306 | 1.866897179 | 0.061915948 | NA |
| P21399 | Cytoplasmic aconitate hydratase | ACO1 | Homo sapiens | 8.033342312 | -0.296799789 | 0.160064313 | -1.854253351 | 0.063702923 | 0.671559783 |
| P32322 | Pyrroline-5-carboxylate reductase 1, mitochondrial | PYCR1 | Homo sapiens | 1.282890877 | 0.801850506 | 0.432724557 | 1.853027505 | 0.063878416 | NA |
| P78559 | Microtubule-associated protein 1A | MAP1A | Homo sapiens | 20.69792671 | -0.199648352 | 0.108163831 | -1.845795861 | 0.064921852 | 0.671923318 |
| P78347 | General transcription factor II-I | GTF2I | Homo sapiens | 1.210076969 | 0.769146959 | 0.416838855 | 1.845190172 | 0.06500988 | NA |
| Q14839 | Chromodomain-helicase-DNA-binding protein 4 | CHD4 | Homo sapiens | 3.608405131 | 0.452105666 | 0.245101913 | 1.844561967 | 0.065101284 | 0.671923318 |
| Q9BS26 | Endoplasmic reticulum resident protein 44 | ERP44 | Homo sapiens | 2.796989385 | -0.516416818 | 0.280407937 | -1.841662629 | 0.065524514 | 0.671923318 |
| P07355 | Annexin A2 | ANXA2 | Homo sapiens | 77.27858251 | -0.112032004 | 0.061123439 | -1.832881227 | 0.066820236 | 0.671923318 |
| Q07065 | Cytoskeleton-associated protein 4 | CKAP4 | Homo sapiens | 64.39679807 | -0.117458051 | 0.064345921 | -1.825415659 | 0.067938328 | 0.671923318 |
| P22307 | Sterol carrier protein 2 | SCP2 | Homo sapiens | 6.380535112 | -0.323439127 | 0.177318706 | -1.824055305 | 0.068143712 | 0.671923318 |
| Q13740 | CD166 antigen | ALCAM | Homo sapiens | 8.810141978 | -0.277634082 | 0.152214688 | -1.823963804 | 0.068157545 | 0.671923318 |
| Q15181 | Inorganic pyrophosphatase | PPA1 | Homo sapiens | 17.10874734 | -0.203359514 | 0.111677351 | -1.820955743 | 0.068613585 | 0.671923318 |
| P30084 | Enoyl-CoA hydratase, mitochondrial | ECHS1 | Homo sapiens | 4.040401154 | -0.452452812 | 0.248904711 | -1.817775209 | 0.069098497 | 0.671923318 |
| Q96CW1 | AP-2 complex subunit mu | AP2M1 | Homo sapiens | 5.093636486 | -0.354752109 | 0.195828026 | -1.811549224 | 0.070055877 | 0.672713622 |
| Q06830 | Peroxiredoxin-1 | PRDX1 | Homo sapiens | 70.99296825 | -0.098762802 | 0.054579999 | -1.809505385 | 0.070372524 | 0.672713622 |
| P10809 | 60 kDa heat shock protein, mitochondrial | HSPD1 | Homo sapiens | 85.76146966 | 0.085684319 | 0.047797659 | 1.792646773 | 0.073029422 | 0.683629653 |
| P52209 | 6-phosphogluconate dehydrogenase, decarboxylating | PGD | Homo sapiens | 45.2220908 | -0.119228702 | 0.066623994 | -1.789576024 | 0.073522096 | 0.683629653 |
| Q5TEC6 | Histone HIST2H3PS2 | H3-2 | Homo sapiens | 8.431575742 | -0.314486214 | 0.17575392 | -1.789355332 | 0.073557608 | 0.683629653 |
| Q9Y450 | HBS1-like protein | HBS1L | Homo sapiens | 1.213735888 | 0.762614893 | 0.427387246 | 1.784365114 | 0.074364356 | NA |
| P24844 | Myosin regulatory light polypeptide 9 | MYL9 | Homo sapiens | 6.748199431 | -0.347060109 | 0.194880242 | -1.780889148 | 0.074930562 | 0.683629653 |
| P06493 | Cyclin-dependent kinase 1 | CDK1 | Homo sapiens | 7.578580647 | 0.287579762 | 0.161627595 | 1.779273902 | 0.075194868 | 0.683629653 |
| P17813 | Endoglin | ENG | Homo sapiens | 4.095090491 | -0.39437642 | 0.221675091 | -1.779074132 | 0.075227609 | 0.683629653 |
| P04004 | Vitronectin | VTN | Homo sapiens | 1.483424691 | -0.643449177 | 0.363468238 | -1.770303726 | 0.076676558 | NA |
| Q13162 | Peroxiredoxin-4 | PRDX4 | Homo sapiens | 35.67120093 | -0.12884122 | 0.072779851 | -1.770286947 | 0.076679351 | 0.683629653 |
| O76094 | Signal recognition particle subunit SRP72 | SRP72 | Homo sapiens | 3.015694262 | 0.458640306 | 0.259289586 | 1.76883427 | 0.076921536 | 0.683629653 |
| Q8TEX9 | Importin-4 | IPO4 | Homo sapiens | 2.194851996 | 0.541456173 | 0.306985399 | 1.763784773 | 0.077768222 | 0.683629653 |
| O75131 | Copine-3 | CPNE3 | Homo sapiens | 2.640332594 | 0.469072182 | 0.266006586 | 1.763385596 | 0.077835478 | 0.683629653 |
| Q92841 | Probable ATP-dependent RNA helicase DDX17 | DDX17 | Homo sapiens | 13.34423809 | -0.208997383 | 0.118658242 | -1.761338953 | 0.078181051 | 0.683629653 |
| P27695 | DNA-(apurinic or apyrimidinic site) endonuclease | APEX1 | Homo sapiens | 8.133669642 | -0.268853211 | 0.153173614 | -1.755218832 | 0.079221884 | 0.687402193 |
| P06576 | ATP synthase subunit beta, mitochondrial | ATP5F1B | Homo sapiens | 98.50742944 | -0.076066662 | 0.043784098 | -1.737312504 | 0.082332026 | 0.708935304 |
| P50281 | Matrix metalloproteinase-14 | MMP14 | Homo sapiens | 2.917542777 | -0.470697841 | 0.271968988 | -1.730704094 | 0.083504555 | 0.713584376 |
| Q9UG63 | ATP-binding cassette sub-family F member 2 | ABCF2 | Homo sapiens | 1.629192101 | 0.598439044 | 0.346654252 | 1.726328295 | 0.084288368 | NA |
| P80723 | Brain acid soluble protein 1 | BASP1 | Homo sapiens | 16.52612187 | -0.191063495 | 0.111051598 | -1.720492972 | 0.08534287 | 0.723810206 |
| O43592 | Exportin-T | XPOT | Homo sapiens | 1.245210226 | 0.698034616 | 0.406522139 | 1.717088811 | 0.085962946 | NA |
| Q9P2E9 | Ribosome-binding protein 1 | RRBP1 | Homo sapiens | 47.14578349 | -0.114254637 | 0.067262357 | -1.698641594 | 0.089386734 | 0.737168873 |
| P26038 | Moesin | MSN | Homo sapiens | 70.78982326 | -0.089932192 | 0.052944953 | -1.698598007 | 0.089394952 | 0.737168873 |
| O14617 | AP-3 complex subunit delta-1 | AP3D1 | Homo sapiens | 2.03545923 | 0.543272033 | 0.319914446 | 1.698179122 | 0.08947396 | 0.737168873 |
| Q71U36 | Tubulin alpha-1A chain | TUBA1A | Homo sapiens | 325.5366174 | -0.060799944 | 0.03580951 | -1.697871406 | 0.089532035 | 0.737168873 |
| Q14764 | Major vault protein | MVP | Homo sapiens | 41.68334112 | -0.119722803 | 0.07068956 | -1.693641927 | 0.090333346 | 0.737464107 |
| Q8NE71 | ATP-binding cassette sub-family F member 1 | ABCF1 | Homo sapiens | 4.265979743 | -0.35778492 | 0.211607176 | -1.690797667 | 0.090875453 | 0.737464107 |
| P02452 | Collagen alpha-1(I) chain | COL1A1 | Homo sapiens | 37.85607625 | 0.217676144 | 0.129410861 | 1.682054676 | 0.092558238 | 0.74572741 |
| Q86UP2 | Kinectin | KTN1 | Homo sapiens | 1.595278912 | -0.597713109 | 0.355455531 | -1.681541165 | 0.092657848 | NA |
| P52272 | Heterogeneous nuclear ribonucleoprotein M | HNRNPM | Homo sapiens | 16.00855997 | 0.179104549 | 0.106838291 | 1.676407843 | 0.093658334 | 0.74572741 |
| P26368 | Splicing factor U2AF 65 kDa subunit | U2AF2 | Homo sapiens | 15.13616918 | 0.182446174 | 0.10890413 | 1.675291603 | 0.093877032 | 0.74572741 |
| Q9Y6Y8 | SEC23-interacting protein | SEC23IP | Homo sapiens | 1.238674262 | -0.68424796 | 0.409011676 | -1.672930138 | 0.09434105 | NA |
| P40939 | Trifunctional enzyme subunit alpha, mitochondrial | HADHA | Homo sapiens | 18.56233223 | -0.176311625 | 0.105488904 | -1.671376029 | 0.094647428 | 0.746589498 |
| Q13263 | Transcription intermediary factor 1-beta | TRIM28 | Homo sapiens | 19.05719396 | 0.173928884 | 0.104803299 | 1.659574513 | 0.097000079 | 0.759833954 |
| Q9UN86 | Ras GTPase-activating protein-binding protein 2 | G3BP2 | Homo sapiens | 3.271391244 | -0.392050628 | 0.23907383 | -1.639872618 | 0.101031655 | 0.774210009 |
| Q92626 | Peroxidasin homolog | PXDN | Homo sapiens | 0.828615994 | -0.856879649 | 0.523787585 | -1.635929667 | 0.101854308 | NA |
| P04264 | Keratin, type II cytoskeletal 1 | KRT1 | Homo sapiens | 58.89434734 | -0.203935313 | 0.124850164 | -1.633440489 | 0.102376387 | 0.774210009 |
| P09972 | Fructose-bisphosphate aldolase C | ALDOC | Homo sapiens | 22.3144274 | -0.149350929 | 0.091438747 | -1.633344005 | 0.102396667 | 0.774210009 |
| P23921 | Ribonucleoside-diphosphate reductase large subunit | RRM1 | Homo sapiens | 2.064315556 | 0.514319914 | 0.315039373 | 1.632557571 | 0.102562081 | 0.774210009 |
| P68104 | Elongation factor 1-alpha 1 | EEF1A1 | Homo sapiens | 292.2767927 | 0.051399626 | 0.031651692 | 1.623913988 | 0.104394167 | 0.774210009 |
| P04040 | Catalase | CAT | Homo sapiens | 1.112922482 | -0.706669689 | 0.435500198 | -1.622662151 | 0.104661647 | NA |
| Q14498 | RNA-binding protein 39 | RBM39 | Homo sapiens | 3.234674094 | 0.388686526 | 0.239902014 | 1.620188671 | 0.105191754 | 0.774210009 |
| P27816 | Microtubule-associated protein 4 | MAP4 | Homo sapiens | 25.29289304 | -0.146608844 | 0.090602914 | -1.618147126 | 0.105630895 | 0.774210009 |
| Q96QK1 | Vacuolar protein sorting-associated protein 35 | VPS35 | Homo sapiens | 6.777904593 | -0.325466649 | 0.201593029 | -1.614473733 | 0.10642471 | 0.774210009 |
| Q3KQU3 | MAP7 domain-containing protein 1 | MAP7D1 | Homo sapiens | 0.846958798 | 0.821742567 | 0.509114657 | 1.614061892 | 0.106514002 | NA |
| Q9H4A4 | Aminopeptidase B | RNPEP | Homo sapiens | 2.348185068 | -0.456527706 | 0.283350147 | -1.611178648 | 0.10714079 | 0.774210009 |
| O43776 | Asparagine--tRNA ligase, cytoplasmic | NARS1 | Homo sapiens | 8.435602219 | -0.242311759 | 0.150427961 | -1.61081595 | 0.107219843 | 0.774210009 |
| P07195 | L-lactate dehydrogenase B chain | LDHB | Homo sapiens | 41.76773303 | -0.122783244 | 0.076391807 | -1.607282894 | 0.107992325 | 0.774210009 |
| P12956 | X-ray repair cross-complementing protein 6 | XRCC6 | Homo sapiens | 23.13517856 | 0.15965387 | 0.099519404 | 1.604248658 | 0.108659253 | 0.774210009 |
| Q9BWD1 | Acetyl-CoA acetyltransferase, cytosolic | ACAT2 | Homo sapiens | 6.014733375 | -0.284222811 | 0.177346372 | -1.60264237 | 0.109013634 | 0.774210009 |
| O60684 | Importin subunit alpha-7 | KPNA6 | Homo sapiens | 1.633905904 | -0.560096588 | 0.34966257 | -1.601820258 | 0.109195362 | NA |
| P49321 | Nuclear autoantigenic sperm protein | NASP | Homo sapiens | 1.750950166 | 0.540498215 | 0.337595729 | 1.601022077 | 0.109372029 | 0.774210009 |
| P98179 | RNA-binding protein 3 | RBM3 | Homo sapiens | 8.076167211 | 0.242511644 | 0.151901435 | 1.596506602 | 0.110375733 | 0.774210009 |
| P30086 | Phosphatidylethanolamine-binding protein 1 | PEBP1 | Homo sapiens | 14.00445806 | -0.189312696 | 0.118604651 | -1.596165874 | 0.110451765 | 0.774210009 |
| Q9H299 | SH3 domain-binding glutamic acid-rich-like protein 3 | SH3BGRL3 | Homo sapiens | 0.705023393 | 0.872000369 | 0.546326844 | 1.596114813 | 0.110463162 | NA |
| P49368 | T-complex protein 1 subunit gamma | CCT3 | Homo sapiens | 24.32165778 | 0.138902373 | 0.087035124 | 1.595934674 | 0.110503379 | 0.774210009 |
| Q03135 | Caveolin-1 | CAV1 | Homo sapiens | 7.250003415 | -0.260746305 | 0.164046851 | -1.589462419 | 0.111956032 | 0.774377179 |
| Q96QV6 | Histone H2A type 1-A | H2AC1 | Homo sapiens | 23.62784938 | -0.249861822 | 0.157675735 | -1.584656144 | 0.113044481 | 0.774377179 |
| Q8NBS9 | Thioredoxin domain-containing protein 5 | TXNDC5 | Homo sapiens | 14.01803436 | -0.185358789 | 0.11719304 | -1.581653557 | 0.113728681 | 0.774377179 |
| Q9Y4L1 | Hypoxia up-regulated protein 1 | HYOU1 | Homo sapiens | 27.1913746 | -0.136918356 | 0.086578923 | -1.581428263 | 0.113780149 | 0.774377179 |
| Q15084 | Protein disulfide-isomerase A6 | PDIA6 | Homo sapiens | 33.63745642 | -0.118051138 | 0.074780543 | -1.578634407 | 0.114419938 | 0.774377179 |
| P60953 | Cell division control protein 42 homolog | CDC42 | Homo sapiens | 15.14849894 | -0.172851053 | 0.1097248 | -1.575314355 | 0.115183902 | 0.774377179 |
| Q15427 | Splicing factor 3B subunit 4 | SF3B4 | Homo sapiens | 1.743589166 | 0.54231066 | 0.344793295 | 1.572857326 | 0.115751858 | 0.774377179 |
| O60568 | Multifunctional procollagen lysine hydroxylase and glycosyltransferase LH3 | PLOD3 | Homo sapiens | 1.820759385 | -0.524983677 | 0.335211899 | -1.566124825 | 0.117319397 | 0.774377179 |
| Q6UVK1 | Chondroitin sulfate proteoglycan 4 | CSPG4 | Homo sapiens | 2.998000116 | 0.560234532 | 0.35777584 | 1.565881396 | 0.117376386 | 0.774377179 |
| P26641 | Elongation factor 1-gamma | EEF1G | Homo sapiens | 68.94608561 | -0.08248761 | 0.052738113 | -1.564098614 | 0.117794411 | 0.774377179 |
| P45974 | Ubiquitin carboxyl-terminal hydrolase 5 | USP5 | Homo sapiens | 17.1936227 | 0.159771462 | 0.102228309 | 1.562888642 | 0.11807879 | 0.774377179 |
| Q9BVC6 | Transmembrane protein 109 | TMEM109 | Homo sapiens | 0.508354516 | 0.882656751 | 0.567053965 | 1.556565698 | 0.119573633 | NA |
| Q9BY44 | Eukaryotic translation initiation factor 2A | EIF2A | Homo sapiens | 2.45780482 | 0.451729264 | 0.290474664 | 1.555141708 | 0.119912324 | 0.781856076 |
| Q9Y5K6 | CD2-associated protein | CD2AP | Homo sapiens | 0.664269035 | 0.870923595 | 0.561121018 | 1.552113658 | 0.120635031 | NA |
| O60884 | DnaJ homolog subfamily A member 2 | DNAJA2 | Homo sapiens | 6.474705577 | 0.259345822 | 0.169024135 | 1.534371543 | 0.124938274 | 0.799712959 |
| P54577 | Tyrosine--tRNA ligase, cytoplasmic | YARS1 | Homo sapiens | 5.315683235 | 0.296102078 | 0.193239686 | 1.532304697 | 0.125447263 | 0.799712959 |
| Q9BUJ2 | Heterogeneous nuclear ribonucleoprotein U-like protein 1 | HNRNPUL1 | Homo sapiens | 3.269399991 | 0.39241063 | 0.256583809 | 1.529366296 | 0.126173663 | 0.799712959 |
| Q9BXP5 | Serrate RNA effector molecule homolog | SRRT | Homo sapiens | 1.844276137 | 0.509747209 | 0.333955028 | 1.526394771 | 0.126911579 | 0.799712959 |
| P60709 | Actin, cytoplasmic 1 | ACTB | Homo sapiens | 725.0621323 | -0.03088213 | 0.020237103 | -1.526015363 | 0.127006038 | 0.799712959 |
| O43747 | AP-1 complex subunit gamma-1 | AP1G1 | Homo sapiens | 3.230768848 | 0.386156548 | 0.254341442 | 1.518260431 | 0.128948759 | 0.799712959 |
| P56192 | Methionine--tRNA ligase, cytoplasmic | MARS1 | Homo sapiens | 5.33898526 | 0.291505449 | 0.192000699 | 1.518252022 | 0.128950878 | 0.799712959 |
| P17858 | ATP-dependent 6-phosphofructokinase, liver type | PFKL | Homo sapiens | 7.104520428 | -0.245230629 | 0.161634828 | -1.517189282 | 0.129218902 | 0.799712959 |
| P54886 | Delta-1-pyrroline-5-carboxylate synthase | ALDH18A1 | Homo sapiens | 9.866532225 | 0.223246996 | 0.147569089 | 1.512830352 | 0.130322757 | 0.799712959 |
| Q93008 | Probable ubiquitin carboxyl-terminal hydrolase FAF-X | USP9X | Homo sapiens | 2.706750598 | -0.459630693 | 0.304109149 | -1.511400413 | 0.130686464 | 0.799712959 |
| Q01813 | ATP-dependent 6-phosphofructokinase, platelet type | PFKP | Homo sapiens | 46.61575895 | 0.102697908 | 0.067972374 | 1.510877166 | 0.130819749 | 0.799712959 |
| P08779 | Keratin, type I cytoskeletal 16 | KRT16 | Homo sapiens | 0.880949721 | -0.826412838 | 0.547141457 | -1.510418974 | 0.13093655 | NA |
| Q99536 | Synaptic vesicle membrane protein VAT-1 homolog | VAT1 | Homo sapiens | 22.99148536 | -0.160193601 | 0.106165582 | -1.508903335 | 0.131323487 | 0.799712959 |
| Q8WZ42 | Titin | TTN | Homo sapiens | 2.208665234 | -0.488455744 | 0.324172329 | -1.506778032 | 0.131867562 | 0.799712959 |
| P49736 | DNA replication licensing factor MCM2 | MCM2 | Homo sapiens | 3.065741389 | 0.382415151 | 0.254583143 | 1.502122831 | 0.133065389 | 0.802136603 |
| Q96IZ0 | PRKC apoptosis WT1 regulator protein | PAWR | Homo sapiens | 0.820949882 | 0.744502761 | 0.49597122 | 1.501100731 | 0.13332951 | NA |
| O43684 | Mitotic checkpoint protein BUB3 | BUB3 | Homo sapiens | 2.727471441 | 0.399266125 | 0.266228816 | 1.499710406 | 0.133689434 | 0.802136603 |
| Q08379 | Golgin subfamily A member 2 | GOLGA2 | Homo sapiens | 0.657400087 | 0.835742109 | 0.558735639 | 1.495773764 | 0.134712621 | NA |
| Q9BQ61 | Telomerase RNA component interacting RNase | TRIR | Homo sapiens | 1.233855302 | 0.612898473 | 0.409818565 | 1.495536136 | 0.134774576 | NA |
| Q92747 | Actin-related protein 2/3 complex subunit 1A | ARPC1A | Homo sapiens | 3.473602268 | -0.34836836 | 0.234385377 | -1.486305861 | 0.137198237 | 0.810642071 |
| Q9Y2T7 | Y-box-binding protein 2 | YBX2 | Homo sapiens | 3.688923694 | 0.357179126 | 0.240618203 | 1.484422716 | 0.137696812 | 0.810642071 |
| P30044 | Peroxiredoxin-5, mitochondrial | PRDX5 | Homo sapiens | 6.843653537 | -0.28176282 | 0.190157798 | -1.481731604 | 0.138411724 | 0.810642071 |
| P08134 | Rho-related GTP-binding protein RhoC | RHOC | Homo sapiens | 7.496278853 | 0.240655865 | 0.162612102 | 1.479938221 | 0.138889735 | 0.810642071 |
| P04062 | Lysosomal acid glucosylceramidase | GBA | Homo sapiens | 1.331184137 | -0.604082285 | 0.408468631 | -1.478895168 | 0.139168335 | NA |
| P42330 | Aldo-keto reductase family 1 member C3 | AKR1C3 | Homo sapiens | 1.613932251 | -0.560988846 | 0.379331699 | -1.478887338 | 0.139170428 | NA |
| O14979 | Heterogeneous nuclear ribonucleoprotein D-like | HNRNPDL | Homo sapiens | 23.56850547 | 0.131844588 | 0.089152953 | 1.478858346 | 0.139178178 | 0.810642071 |
| P02786 | Transferrin receptor protein 1 | TFRC | Homo sapiens | 14.32787181 | -0.171448568 | 0.116003656 | -1.477958309 | 0.139418938 | 0.810642071 |
| Q15654 | Thyroid receptor-interacting protein 6 | TRIP6 | Homo sapiens | 2.700139405 | 0.412285621 | 0.281044914 | 1.46697414 | 0.14238309 | 0.813504886 |
| Q9UQE7 | Structural maintenance of chromosomes protein 3 | SMC3 | Homo sapiens | 1.745308135 | -0.563260183 | 0.384032152 | -1.466700588 | 0.142457523 | 0.813504886 |
| O00487 | 26S proteasome non-ATPase regulatory subunit 14 | PSMD14 | Homo sapiens | 2.703694838 | -0.417883946 | 0.285156055 | -1.465457034 | 0.142796267 | 0.813504886 |
| P31689 | DnaJ homolog subfamily A member 1 | DNAJA1 | Homo sapiens | 9.408060849 | 0.205242473 | 0.140332186 | 1.462547392 | 0.143591267 | 0.813504886 |
| P23526 | Adenosylhomocysteinase | AHCY | Homo sapiens | 2.422462755 | -0.41032167 | 0.28068123 | -1.461877839 | 0.143774689 | 0.813504886 |
| O75368 | SH3 domain-binding glutamic acid-rich-like protein | SH3BGRL | Homo sapiens | 1.824977307 | -0.571893157 | 0.391657251 | -1.460187845 | 0.144238455 | 0.813504886 |
| P08729 | Keratin, type II cytoskeletal 7 | KRT7 | Homo sapiens | 1.418592382 | 0.87863144 | 0.602226976 | 1.458970579 | 0.144573206 | NA |
| P00390 | Glutathione reductase, mitochondrial | GSR | Homo sapiens | 7.172068928 | -0.252045638 | 0.173066603 | -1.456350522 | 0.145295746 | 0.815391051 |
| P31947 | 14-3-3 protein sigma | SFN | Homo sapiens | 4.044325398 | -0.339714999 | 0.233776169 | -1.453163514 | 0.14617836 | 0.816283118 |
| P56537 | Eukaryotic translation initiation factor 6 | EIF6 | Homo sapiens | 3.365842288 | 0.348184947 | 0.240261627 | 1.449190832 | 0.147284297 | 0.818407327 |
| P09960 | Leukotriene A-4 hydrolase | LTA4H | Homo sapiens | 1.044265544 | -0.671280869 | 0.463983924 | -1.446776138 | 0.147959632 | NA |
| Q9BY77 | Polymerase delta-interacting protein 3 | POLDIP3 | Homo sapiens | 0.615389791 | 0.814816654 | 0.563310808 | 1.446477934 | 0.148043197 | NA |
| P49588 | Alanine--tRNA ligase, cytoplasmic | AARS1 | Homo sapiens | 21.62305042 | 0.132654369 | 0.092033539 | 1.441369868 | 0.149480218 | 0.819787747 |
| Q86UE4 | Protein LYRIC | MTDH | Homo sapiens | 3.288492458 | -0.359229519 | 0.24929528 | -1.440980025 | 0.149590326 | 0.819787747 |
| P12004 | Proliferating cell nuclear antigen | PCNA | Homo sapiens | 8.843689626 | 0.207036076 | 0.143720568 | 1.440545912 | 0.149713011 | 0.819787747 |
| Q5JTH9 | RRP12-like protein | RRP12 | Homo sapiens | 1.365301142 | 0.59561218 | 0.415033952 | 1.435092666 | 0.151260693 | NA |
| P13645 | Keratin, type I cytoskeletal 10 | KRT10 | Homo sapiens | 57.68993857 | -0.241239287 | 0.168415788 | -1.432403046 | 0.152028507 | 0.8284452 |
| P04844 | Dolichyl-diphosphooligosaccharide--protein glycosyltransferase subunit 2 | RPN2 | Homo sapiens | 21.45193765 | -0.136778463 | 0.096573666 | -1.416312213 | 0.156684114 | 0.849710001 |
| P18754 | Regulator of chromosome condensation | RCC1 | Homo sapiens | 0.867022694 | 0.718215885 | 0.508558861 | 1.412257144 | 0.15787426 | NA |
| P16403 | Histone H1.2 | H1-2 | Homo sapiens | 30.77544917 | 0.114698571 | 0.081283572 | 1.411091665 | 0.158217587 | 0.853920758 |
| Q09666 | Neuroblast differentiation-associated protein AHNAK | AHNAK | Homo sapiens | 297.8259202 | 0.067725893 | 0.048171296 | 1.405938776 | 0.159742306 | 0.855776654 |
| O75396 | Vesicle-trafficking protein SEC22b | SEC22B | Homo sapiens | 1.948995146 | -0.452381404 | 0.3221072 | -1.404443627 | 0.160186787 | 0.855776654 |
| Q92888 | Rho guanine nucleotide exchange factor 1 | ARHGEF1 | Homo sapiens | 0.621706821 | 0.779020325 | 0.555816101 | 1.40157927 | 0.161040921 | NA |
| Q14847 | LIM and SH3 domain protein 1 | LASP1 | Homo sapiens | 9.509808425 | -0.197435602 | 0.141161881 | -1.39864672 | 0.161918949 | 0.855776654 |
| P51659 | Peroxisomal multifunctional enzyme type 2 | HSD17B4 | Homo sapiens | 8.949220424 | -0.215576383 | 0.154216299 | -1.397883256 | 0.162148128 | 0.855776654 |
| P0CG39 | POTE ankyrin domain family member J | POTEJ | Homo sapiens | 32.96952994 | -0.113223825 | 0.081036494 | -1.397195496 | 0.162354791 | 0.855776654 |
| P33992 | DNA replication licensing factor MCM5 | MCM5 | Homo sapiens | 1.134043116 | 0.662835298 | 0.474941269 | 1.395615292 | 0.162830375 | NA |
| P19971 | Thymidine phosphorylase | TYMP | Homo sapiens | 1.830854323 | -0.498732245 | 0.359075168 | -1.388935492 | 0.164852367 | 0.86489986 |
| P30876 | DNA-directed RNA polymerase II subunit RPB2 | POLR2B | Homo sapiens | 0.871445238 | 0.639535752 | 0.460786014 | 1.387923534 | 0.165160331 | NA |
| Q99832 | T-complex protein 1 subunit eta | CCT7 | Homo sapiens | 31.34007651 | 0.10561264 | 0.07629255 | 1.384311301 | 0.166263155 | 0.868263143 |
| Q9UL25 | Ras-related protein Rab-21 | RAB21 | Homo sapiens | 4.076060506 | -0.295713121 | 0.214193034 | -1.380591681 | 0.167404543 | 0.870195042 |
| P35354 | Prostaglandin G/H synthase 2 | PTGS2 | Homo sapiens | 5.187581435 | -0.536394368 | 0.389450856 | -1.377309512 | 0.168416575 | 0.870745296 |
| Q93052 | Lipoma-preferred partner | LPP | Homo sapiens | 1.739750941 | -0.457474045 | 0.333190592 | -1.373010091 | 0.16974921 | 0.870745296 |
| Q08J23 | RNA cytosine C(5)-methyltransferase NSUN2 | NSUN2 | Homo sapiens | 2.566918284 | 0.374041709 | 0.272706163 | 1.371592432 | 0.170190351 | 0.870745296 |
| Q14011 | Cold-inducible RNA-binding protein | CIRBP | Homo sapiens | 2.436259951 | 0.392489494 | 0.286429226 | 1.370284379 | 0.170598147 | 0.870745296 |
| P09619 | Platelet-derived growth factor receptor beta | PDGFRB | Homo sapiens | 1.075292956 | -0.776182285 | 0.569653539 | -1.362551501 | 0.173023903 | NA |
| O60610 | Protein diaphanous homolog 1 | DIAPH1 | Homo sapiens | 4.03127938 | 0.307819933 | 0.226430188 | 1.359447415 | 0.174004855 | 0.884132776 |
| P60660 | Myosin light polypeptide 6 | MYL6 | Homo sapiens | 24.19163307 | 0.116822138 | 0.086427173 | 1.351682973 | 0.176476754 | 0.892671654 |
| P04632 | Calpain small subunit 1 | CAPNS1 | Homo sapiens | 14.10861864 | -0.158108148 | 0.117427325 | -1.346434047 | 0.178162577 | 0.894244428 |
| P84243 | Histone H3.3 | H3-3A | Homo sapiens | 11.66497481 | -0.190288768 | 0.141465532 | -1.345124607 | 0.178584998 | 0.894244428 |
| P29692 | Elongation factor 1-delta | EEF1D | Homo sapiens | 8.056062003 | -0.216665698 | 0.162085896 | -1.336733815 | 0.18130954 | 0.894244428 |
| P08243 | Asparagine synthetase [glutamine-hydrolyzing] | ASNS | Homo sapiens | 3.140634096 | 0.328733901 | 0.246010232 | 1.336261093 | 0.181463949 | 0.894244428 |
| P50990 | T-complex protein 1 subunit theta | CCT8 | Homo sapiens | 17.21210212 | -0.139947714 | 0.104734574 | -1.336213142 | 0.181479617 | 0.894244428 |
| Q5SSJ5 | Heterochromatin protein 1-binding protein 3 | HP1BP3 | Homo sapiens | 1.08826856 | 0.569121956 | 0.426212237 | 1.335301773 | 0.181777599 | NA |
| P49207 | 60S ribosomal protein L34 | RPL34 | Homo sapiens | 0.536419025 | 0.820016123 | 0.614472757 | 1.334503627 | 0.182038859 | NA |
| Q96F85 | CB1 cannabinoid receptor-interacting protein 1 | CNRIP1 | Homo sapiens | 0.751576247 | 0.648021619 | 0.485725404 | 1.334131616 | 0.182160726 | NA |
| Q10471 | Polypeptide N-acetylgalactosaminyltransferase 2 | GALNT2 | Homo sapiens | 1.901213048 | -0.435622446 | 0.326670357 | -1.333523036 | 0.182360221 | 0.894244428 |
| Q9UMS4 | Pre-mRNA-processing factor 19 | PRPF19 | Homo sapiens | 5.319257699 | 0.278160876 | 0.208825792 | 1.332023566 | 0.182852445 | 0.894244428 |
| Q14697 | Neutral alpha-glucosidase AB | GANAB | Homo sapiens | 34.20875073 | -0.102940904 | 0.077330576 | -1.331179841 | 0.183129843 | 0.894244428 |
| Q9Y266 | Nuclear migration protein nudC | NUDC | Homo sapiens | 1.275236789 | -0.546601716 | 0.413566071 | -1.321679303 | 0.186274964 | NA |
| Q13185 | Chromobox protein homolog 3 | CBX3 | Homo sapiens | 3.723610771 | 0.298771614 | 0.226714367 | 1.31783273 | 0.187559649 | 0.897894371 |
| Q14974 | Importin subunit beta-1 | KPNB1 | Homo sapiens | 50.87907715 | 0.079981542 | 0.060720519 | 1.31720781 | 0.187768977 | 0.897894371 |
| P36871 | Phosphoglucomutase-1 | PGM1 | Homo sapiens | 7.091648052 | -0.228247302 | 0.173302406 | -1.317046358 | 0.187823086 | 0.897894371 |
| P55010 | Eukaryotic translation initiation factor 5 | EIF5 | Homo sapiens | 2.129553468 | 0.388805726 | 0.295421665 | 1.316104311 | 0.188139034 | 0.897894371 |
| P23588 | Eukaryotic translation initiation factor 4B | EIF4B | Homo sapiens | 5.290681766 | 0.260195662 | 0.198010355 | 1.314050783 | 0.188829115 | 0.897894371 |
| P35611 | Alpha-adducin | ADD1 | Homo sapiens | 1.043031629 | -0.5660056 | 0.432837113 | -1.307664207 | 0.190987229 | NA |
| P40222 | Alpha-taxilin | TXLNA | Homo sapiens | 3.020049274 | 0.33576679 | 0.25701296 | 1.306419681 | 0.191409876 | 0.897894371 |
| P63010 | AP-2 complex subunit beta | AP2B1 | Homo sapiens | 21.36327156 | -0.121954409 | 0.093397174 | -1.305761233 | 0.191633767 | 0.897894371 |
| Q9HAV0 | Guanine nucleotide-binding protein subunit beta-4 | GNB4 | Homo sapiens | 2.478494245 | -0.351975198 | 0.269724048 | -1.304945556 | 0.191911387 | 0.897894371 |
| P52292 | Importin subunit alpha-1 | KPNA2 | Homo sapiens | 15.36306919 | 0.152004761 | 0.116500981 | 1.304750902 | 0.191977683 | 0.897894371 |
| P55060 | Exportin-2 | CSE1L | Homo sapiens | 17.95306885 | 0.136433503 | 0.104711968 | 1.302940873 | 0.192594948 | 0.897894371 |
| P40763 | Signal transducer and activator of transcription 3 | STAT3 | Homo sapiens | 3.075922266 | -0.330550149 | 0.253717342 | -1.302828358 | 0.192633367 | 0.897894371 |
| Q9Y230 | RuvB-like 2 | RUVBL2 | Homo sapiens | 0.787124295 | 0.664691289 | 0.511262675 | 1.300097429 | 0.193567579 | NA |
| P12268 | Inosine-5'-monophosphate dehydrogenase 2 | IMPDH2 | Homo sapiens | 8.897529423 | 0.198103893 | 0.152504589 | 1.299002825 | 0.19394296 | 0.900278429 |
| P30085 | UMP-CMP kinase | CMPK1 | Homo sapiens | 7.698467093 | -0.216996722 | 0.16759044 | -1.294803698 | 0.195387956 | 0.901432607 |
| P07858 | Cathepsin B | CTSB | Homo sapiens | 5.988325457 | -0.231029722 | 0.178890603 | -1.29145812 | 0.196544868 | 0.901432607 |
| Q8N3D4 | EH domain-binding protein 1-like protein 1 | EHBP1L1 | Homo sapiens | 1.041793294 | -0.535255941 | 0.414854476 | -1.290225782 | 0.196972277 | NA |
| P52789 | Hexokinase-2 | HK2 | Homo sapiens | 2.601354993 | -0.369740799 | 0.286579057 | -1.290187789 | 0.196985465 | 0.901432607 |
| Q7Z794 | Keratin, type II cytoskeletal 1b | KRT77 | Homo sapiens | 4.013684899 | -0.276583397 | 0.21456732 | -1.289028529 | 0.197388168 | 0.901432607 |
| Q04206 | Transcription factor p65 | RELA | Homo sapiens | 0.684613946 | 0.720694594 | 0.559291927 | 1.288583939 | 0.19754277 | NA |
| P49454 | Centromere protein F | CENPF | Homo sapiens | 0.706748131 | 0.663858982 | 0.515800526 | 1.287045958 | 0.19807827 | NA |
| P43034 | Platelet-activating factor acetylhydrolase IB subunit beta | PAFAH1B1 | Homo sapiens | 8.594615811 | -0.186741719 | 0.145138713 | -1.286643065 | 0.198218726 | 0.901575496 |
| P38117 | Electron transfer flavoprotein subunit beta | ETFB | Homo sapiens | 1.391932671 | -0.517366244 | 0.403226315 | -1.283066668 | 0.19946872 | NA |
| P51397 | Death-associated protein 1 | DAP | Homo sapiens | 0.486137774 | -0.992132642 | 0.774620482 | -1.280798359 | 0.200264501 | NA |
| P22102 | Trifunctional purine biosynthetic protein adenosine-3 | GART | Homo sapiens | 9.831470893 | 0.185554688 | 0.145226526 | 1.277691436 | 0.201358246 | 0.909113127 |
| Q14566 | DNA replication licensing factor MCM6 | MCM6 | Homo sapiens | 3.573639242 | 0.319492521 | 0.250691532 | 1.27444481 | 0.20250582 | 0.909113127 |
| Q6UN15 | Pre-mRNA 3'-end-processing factor FIP1 | FIP1L1 | Homo sapiens | 0.690408662 | 0.629850191 | 0.495260355 | 1.271755722 | 0.203459925 | NA |
| Q8N6T3 | ADP-ribosylation factor GTPase-activating protein 1 | ARFGAP1 | Homo sapiens | 1.741918628 | 0.427203411 | 0.336049206 | 1.271252552 | 0.203638816 | 0.909113127 |
| P22314 | Ubiquitin-like modifier-activating enzyme 1 | UBA1 | Homo sapiens | 81.10391454 | -0.060906263 | 0.04795935 | -1.269955954 | 0.20410032 | 0.909113127 |
| Q9Y295 | Developmentally-regulated GTP-binding protein 1 | DRG1 | Homo sapiens | 2.763391828 | 0.328574142 | 0.259061043 | 1.2683271 | 0.204681163 | 0.909113127 |
| P30040 | Endoplasmic reticulum resident protein 29 | ERP29 | Homo sapiens | 1.908428092 | 0.420999734 | 0.331955437 | 1.268241716 | 0.204711644 | 0.909113127 |
| P07339 | Cathepsin D | CTSD | Homo sapiens | 7.312257169 | -0.206539038 | 0.163557059 | -1.262795013 | 0.206662857 | 0.911390481 |
| Q9Y6G9 | Cytoplasmic dynein 1 light intermediate chain 1 | DYNC1LI1 | Homo sapiens | 5.119790896 | 0.256597858 | 0.203277823 | 1.262301289 | 0.206840393 | 0.911390481 |
| P68133 | Actin, alpha skeletal muscle | ACTA1 | Homo sapiens | 157.970691 | -0.057623243 | 0.045782003 | -1.258643973 | 0.208158957 | 0.913631529 |
| O00203 | AP-3 complex subunit beta-1 | AP3B1 | Homo sapiens | 1.658704029 | -0.43332004 | 0.345763463 | -1.253226806 | 0.210123177 | NA |
| Q96KR1 | Zinc finger RNA-binding protein | ZFR | Homo sapiens | 0.811203443 | 0.576160727 | 0.461829682 | 1.247561058 | 0.212191847 | NA |
| O00154 | Cytosolic acyl coenzyme A thioester hydrolase | ACOT7 | Homo sapiens | 4.113909595 | -0.262893387 | 0.211357497 | -1.243832797 | 0.213561107 | 0.93232858 |
| P31930 | Cytochrome b-c1 complex subunit 1, mitochondrial | UQCRC1 | Homo sapiens | 2.935028525 | -0.326880587 | 0.263240775 | -1.241755146 | 0.214326917 | 0.93232858 |
| Q9H3Z4 | DnaJ homolog subfamily C member 5 | DNAJC5 | Homo sapiens | 1.090375437 | -0.677438184 | 0.547007945 | -1.238443043 | 0.215551829 | NA |
| Q14696 | LRP chaperone MESD | MESD | Homo sapiens | 0.570140781 | 0.770347536 | 0.623046967 | 1.236419687 | 0.216302602 | NA |
| P04216 | Thy-1 membrane glycoprotein | THY1 | Homo sapiens | 8.498493108 | -0.188604667 | 0.152555306 | -1.236303557 | 0.216345749 | 0.93232858 |
| Q1KMD3 | Heterogeneous nuclear ribonucleoprotein U-like protein 2 | HNRNPUL2 | Homo sapiens | 8.220523293 | 0.185003292 | 0.14982843 | 1.234767604 | 0.216917008 | 0.93232858 |
| P62841 | 40S ribosomal protein S15 | RPS15 | Homo sapiens | 5.226080489 | 0.234289195 | 0.189848859 | 1.234082715 | 0.217172084 | 0.93232858 |
| P05556 | Integrin beta-1 | ITGB1 | Homo sapiens | 43.69932965 | -0.08557578 | 0.069564038 | -1.230172702 | 0.21863244 | 0.93232858 |
| P62266 | 40S ribosomal protein S23 | RPS23 | Homo sapiens | 19.0003886 | 0.121672978 | 0.099033984 | 1.228598234 | 0.219222478 | 0.93232858 |
| Q15165 | Serum paraoxonase/arylesterase 2 | PON2 | Homo sapiens | 0.734604903 | -0.627017224 | 0.510474465 | -1.228302819 | 0.219333313 | NA |
| Q9Y2Z9 | Ubiquinone biosynthesis monooxygenase COQ6, mitochondrial | COQ6 | Homo sapiens | 1.926532104 | -0.412545754 | 0.336170526 | -1.227191924 | 0.219750464 | 0.93232858 |
| Q9H1E3 | Nuclear ubiquitous casein and cyclin-dependent kinase substrate 1 | NUCKS1 | Homo sapiens | 1.719314877 | 0.48459405 | 0.39497218 | 1.22690679 | 0.219857626 | 0.93232858 |
| Q14767 | Latent-transforming growth factor beta-binding protein 2 | LTBP2 | Homo sapiens | 1.249582048 | 0.473875661 | 0.38980251 | 1.2156814 | 0.224106312 | NA |
| Q92599 | Septin-8 | SEPTIN8 | Homo sapiens | 7.904325468 | 0.18338707 | 0.150871547 | 1.215517927 | 0.224168616 | 0.944192524 |
| Q9UJ70 | N-acetyl-D-glucosamine kinase | NAGK | Homo sapiens | 2.568318895 | -0.349405953 | 0.287554165 | -1.21509613 | 0.22432943 | 0.944192524 |
| P23528 | Cofilin-1 | CFL1 | Homo sapiens | 55.7842252 | -0.072235308 | 0.059880213 | -1.206330171 | 0.227690209 | 0.954775299 |
| Q8N163 | Cell cycle and apoptosis regulator protein 2 | CCAR2 | Homo sapiens | 1.351357811 | 0.583548446 | 0.483825845 | 1.206112597 | 0.227774078 | NA |
| Q9BXK5 | Bcl-2-like protein 13 | BCL2L13 | Homo sapiens | 0.573555843 | -0.933044876 | 0.774334958 | -1.204962874 | 0.228217635 | NA |
| P28838 | Cytosol aminopeptidase | LAP3 | Homo sapiens | 11.94845479 | -0.168625095 | 0.140290895 | -1.201967489 | 0.229376125 | 0.958282476 |
| Q7Z7H5 | Transmembrane emp24 domain-containing protein 4 | TMED4 | Homo sapiens | 1.678113065 | -0.403807277 | 0.336491777 | -1.200050949 | 0.230119554 | NA |
| P26885 | Peptidyl-prolyl cis-trans isomerase FKBP2 | FKBP2 | Homo sapiens | 1.03912068 | 0.511949481 | 0.427040943 | 1.198829971 | 0.230594066 | NA |
| P52907 | F-actin-capping protein subunit alpha-1 | CAPZA1 | Homo sapiens | 22.59440504 | -0.110435185 | 0.092321086 | -1.196207609 | 0.231615552 | 0.96198605 |
| O00193 | Small acidic protein | SMAP | Homo sapiens | 0.659711582 | 0.606252595 | 0.507889652 | 1.193669908 | 0.232607116 | NA |
| P12081 | Histidine--tRNA ligase, cytoplasmic | HARS1 | Homo sapiens | 2.474031416 | -0.398717167 | 0.334897465 | -1.190564901 | 0.233824437 | 0.96198605 |
| Q14141 | Septin-6 | SEPTIN6 | Homo sapiens | 11.33531679 | 0.149883989 | 0.125897039 | 1.190528307 | 0.233838811 | 0.96198605 |
| P19105 | Myosin regulatory light chain 12A | MYL12A | Homo sapiens | 15.50096343 | -0.158405097 | 0.133173312 | -1.189465782 | 0.234256429 | 0.96198605 |
| Q13526 | Peptidyl-prolyl cis-trans isomerase NIMA-interacting 1 | PIN1 | Homo sapiens | 1.674218698 | -0.389190183 | 0.327614838 | -1.187950418 | 0.234852945 | NA |
| P62081 | 40S ribosomal protein S7 | RPS7 | Homo sapiens | 29.74689839 | 0.093324461 | 0.078577548 | 1.187673364 | 0.234962122 | 0.96198605 |
| P11166 | Solute carrier family 2, facilitated glucose transporter member 1 | SLC2A1 | Homo sapiens | 4.88305971 | -0.238195843 | 0.200778736 | -1.18635991 | 0.235480197 | 0.96198605 |
| Q13151 | Heterogeneous nuclear ribonucleoprotein A0 | HNRNPA0 | Homo sapiens | 3.671079509 | 0.268108994 | 0.226428447 | 1.184078224 | 0.236382099 | 0.96198605 |
| P78527 | DNA-dependent protein kinase catalytic subunit | PRKDC | Homo sapiens | 16.67015782 | 0.146225047 | 0.12397338 | 1.179487463 | 0.238204126 | 0.96198605 |
| P27708 | CAD protein | CAD | Homo sapiens | 7.632780272 | 0.181277786 | 0.154482167 | 1.173454442 | 0.240613616 | 0.96198605 |
| P31040 | Succinate dehydrogenase [ubiquinone] flavoprotein subunit, mitochondrial | SDHA | Homo sapiens | 8.231889848 | -0.175895563 | 0.150033633 | -1.172374223 | 0.241046844 | 0.96198605 |
| P40429 | 60S ribosomal protein L13a | RPL13A | Homo sapiens | 4.850198855 | -0.231316479 | 0.197320427 | -1.17228856 | 0.241081223 | 0.96198605 |
| Q9UBT2 | SUMO-activating enzyme subunit 2 | UBA2 | Homo sapiens | 5.422426168 | -0.218885451 | 0.186779633 | -1.171891429 | 0.241240649 | 0.96198605 |
| P16104 | Histone H2AX | H2AX | Homo sapiens | 8.430078039 | -0.293000249 | 0.250431293 | -1.169982576 | 0.242007981 | 0.96198605 |
| P28066 | Proteasome subunit alpha type-5 | PSMA5 | Homo sapiens | 0.767937509 | 0.602455901 | 0.516014678 | 1.167516986 | 0.243001653 | NA |
| P0DJD0 | RANBP2-like and GRIP domain-containing protein 1 | RGPD1 | Homo sapiens | 1.091824428 | 0.521530041 | 0.446748393 | 1.167390973 | 0.243052515 | NA |
| Q13126 | S-methyl-5'-thioadenosine phosphorylase | MTAP | Homo sapiens | 4.158872699 | -0.253675338 | 0.217618254 | -1.165689615 | 0.243739961 | 0.96198605 |
| Q15293 | Reticulocalbin-1 | RCN1 | Homo sapiens | 2.320626713 | -0.34749303 | 0.29909788 | -1.161803723 | 0.245315201 | 0.96198605 |
| P0CG38 | POTE ankyrin domain family member I | POTEI | Homo sapiens | 32.8811738 | -0.09938205 | 0.085627911 | -1.160626819 | 0.245793694 | 0.96198605 |
| P49748 | Very long-chain specific acyl-CoA dehydrogenase, mitochondrial | ACADVL | Homo sapiens | 4.577618227 | 0.24114193 | 0.207990772 | 1.159387636 | 0.246298214 | 0.96198605 |
| Q7Z434 | Mitochondrial antiviral-signaling protein | MAVS | Homo sapiens | 1.434121629 | -0.438500289 | 0.379142161 | -1.156559027 | 0.24745257 | NA |
| Q6S8J3 | POTE ankyrin domain family member E | POTEE | Homo sapiens | 83.04900902 | -0.071588146 | 0.06195054 | -1.155569364 | 0.247857345 | 0.96198605 |
| P50579 | Methionine aminopeptidase 2 | METAP2 | Homo sapiens | 1.838866479 | 0.437599248 | 0.378687991 | 1.155566743 | 0.247858418 | 0.96198605 |
| Q16891 | MICOS complex subunit MIC60 | IMMT | Homo sapiens | 2.075669779 | -0.359487186 | 0.312025294 | -1.152109116 | 0.249276237 | 0.96198605 |
| P62917 | 60S ribosomal protein L8 | RPL8 | Homo sapiens | 6.462483536 | 0.198864666 | 0.17266105 | 1.151763329 | 0.249418341 | 0.96198605 |
| P13929 | Beta-enolase | ENO3 | Homo sapiens | 14.22840032 | 0.165769674 | 0.144027158 | 1.150961218 | 0.249748191 | 0.96198605 |
| P0DPB5 | Protein POLR1D, isoform 2 | POLR1D | Homo sapiens | 1.722559519 | -0.380339678 | 0.332182936 | -1.144970547 | 0.252221358 | 0.96198605 |
| P24534 | Elongation factor 1-beta | EEF1B2 | Homo sapiens | 10.04761589 | -0.167380659 | 0.146266644 | -1.144352907 | 0.25247731 | 0.96198605 |
| P60866 | 40S ribosomal protein S20 | RPS20 | Homo sapiens | 14.21489663 | 0.129500124 | 0.113611773 | 1.13984775 | 0.254349736 | 0.96198605 |
| Q8NBJ5 | Procollagen galactosyltransferase 1 | COLGALT1 | Homo sapiens | 1.966198879 | 0.39752942 | 0.348866406 | 1.139488963 | 0.254499269 | 0.96198605 |
| P30419 | Glycylpeptide N-tetradecanoyltransferase 1 | NMT1 | Homo sapiens | 3.200037561 | 0.271708258 | 0.238840098 | 1.137615754 | 0.255280965 | 0.96198605 |
| O15460 | Prolyl 4-hydroxylase subunit alpha-2 | P4HA2 | Homo sapiens | 6.292708291 | -0.228291958 | 0.200754273 | -1.137171105 | 0.255466764 | 0.96198605 |
| Q8WXI7 | Mucin-16 | MUC16 | Homo sapiens | 1.509396961 | 0.438142548 | 0.385638702 | 1.136147763 | 0.255894728 | NA |
| Q96EP5 | DAZ-associated protein 1 | DAZAP1 | Homo sapiens | 2.746848494 | 0.297347561 | 0.261867198 | 1.13548991 | 0.256170107 | 0.96198605 |
| O94973 | AP-2 complex subunit alpha-2 | AP2A2 | Homo sapiens | 5.32823417 | -0.210850399 | 0.185874568 | -1.13436927 | 0.256639684 | 0.96198605 |
| Q96P70 | Importin-9 | IPO9 | Homo sapiens | 6.816975751 | 0.187603288 | 0.165516363 | 1.133442544 | 0.257028457 | 0.96198605 |
| Q8N684 | Cleavage and polyadenylation specificity factor subunit 7 | CPSF7 | Homo sapiens | 1.887318892 | 0.380519646 | 0.336090584 | 1.132193713 | 0.257553003 | 0.96198605 |
| O96005 | Cleft lip and palate transmembrane protein 1 | CLPTM1 | Homo sapiens | 1.486095774 | 0.460406455 | 0.407479358 | 1.129889027 | 0.258522988 | NA |
| P21964 | Catechol O-methyltransferase | COMT | Homo sapiens | 0.884428903 | 0.527963081 | 0.467273784 | 1.129879523 | 0.258526993 | NA |
| P19022 | Cadherin-2 | CDH2 | Homo sapiens | 2.0411853 | 0.487858879 | 0.432284803 | 1.128558941 | 0.25908394 | 0.964510508 |
| Q92734 | Protein TFG | TFG | Homo sapiens | 4.657555536 | -0.237411704 | 0.211412876 | -1.12297656 | 0.26144745 | 0.967754341 |
| P56134 | ATP synthase subunit f, mitochondrial | ATP5MF | Homo sapiens | 0.672556158 | -0.583879786 | 0.5200801 | -1.122672808 | 0.261576481 | NA |
| P09110 | 3-ketoacyl-CoA thiolase, peroxisomal | ACAA1 | Homo sapiens | 2.510872818 | -0.31424521 | 0.280038241 | -1.122151064 | 0.261798215 | 0.967754341 |
| P17302 | Gap junction alpha-1 protein | GJA1 | Homo sapiens | 1.254528176 | -0.617784825 | 0.551131893 | -1.12093826 | 0.262314144 | NA |
| P78417 | Glutathione S-transferase omega-1 | GSTO1 | Homo sapiens | 6.805889061 | -0.266050071 | 0.237452813 | -1.120433436 | 0.262529103 | 0.967754341 |
| Q5ZPR3 | CD276 antigen | CD276 | Homo sapiens | 1.2690248 | -0.437540535 | 0.393115205 | -1.113008425 | 0.265704811 | NA |
| Q68CZ2 | Tensin-3 | TNS3 | Homo sapiens | 1.383264877 | -0.41753831 | 0.375999914 | -1.110474482 | 0.266794618 | NA |
| Q9H930 | Nuclear body protein SP140-like protein | SP140L | Homo sapiens | 1.799099066 | 0.380124717 | 0.342984307 | 1.108286035 | 0.267738305 | 0.97909037 |
| P84095 | Rho-related GTP-binding protein RhoG | RHOG | Homo sapiens | 2.52658636 | 0.31856424 | 0.288276161 | 1.105066192 | 0.269130909 | 0.97909037 |
| Q9UHX1 | Poly(U)-binding-splicing factor PUF60 | PUF60 | Homo sapiens | 7.645482455 | 0.170274711 | 0.154218246 | 1.104115206 | 0.269543166 | 0.97909037 |
| P61981 | 14-3-3 protein gamma | YWHAG | Homo sapiens | 27.69771278 | -0.089115687 | 0.080742436 | -1.103703219 | 0.269721899 | 0.97909037 |
| Q9NP72 | Ras-related protein Rab-18 | RAB18 | Homo sapiens | 3.294182371 | 0.26054029 | 0.237133539 | 1.098707048 | 0.271895867 | 0.97909037 |
| Q03252 | Lamin-B2 | LMNB2 | Homo sapiens | 1.497534614 | 0.420998903 | 0.383663451 | 1.097313028 | 0.272504578 | NA |
| Q9Y4G6 | Talin-2 | TLN2 | Homo sapiens | 2.831125991 | -0.306398321 | 0.279659956 | -1.095610277 | 0.273249362 | 0.97909037 |
| Q96CS3 | FAS-associated factor 2 | FAF2 | Homo sapiens | 2.564548403 | 0.300605078 | 0.27475055 | 1.094101828 | 0.273910321 | 0.97909037 |
| O75312 | Zinc finger protein ZPR1 | ZPR1 | Homo sapiens | 1.035634322 | -0.459668389 | 0.420604685 | -1.092875104 | 0.27444864 | NA |
| Q9NTK5 | Obg-like ATPase 1 | OLA1 | Homo sapiens | 2.837059317 | 0.303370438 | 0.277688485 | 1.092484763 | 0.274620084 | 0.97909037 |
| Q04446 | 1,4-alpha-glucan-branching enzyme | GBE1 | Homo sapiens | 1.537791596 | -0.449937198 | 0.412168652 | -1.091633718 | 0.27499413 | NA |
| Q14568 | Heat shock protein HSP 90-alpha A2 | HSP90AA2P | Homo sapiens | 13.69292192 | -0.151779957 | 0.139509807 | -1.087951879 | 0.276616352 | 0.97909037 |
| O95433 | Activator of 90 kDa heat shock protein ATPase homolog 1 | AHSA1 | Homo sapiens | 2.156568364 | 0.324113949 | 0.298140278 | 1.08711896 | 0.27698424 | 0.97909037 |
| P31939 | Bifunctional purine biosynthesis protein ATIC | ATIC | Homo sapiens | 21.91784072 | -0.101323341 | 0.093255181 | -1.086517015 | 0.277250317 | 0.97909037 |
| Q9H9B4 | Sideroflexin-1 | SFXN1 | Homo sapiens | 1.281372618 | 0.435930617 | 0.402904446 | 1.081970235 | 0.279265754 | NA |
| P16152 | Carbonyl reductase [NADPH] 1 | CBR1 | Homo sapiens | 15.41179377 | -0.124478217 | 0.115265892 | -1.07992239 | 0.280176742 | 0.97909037 |
| Q14240 | Eukaryotic initiation factor 4A-II | EIF4A2 | Homo sapiens | 8.975961175 | 0.186153019 | 0.173210918 | 1.074718738 | 0.282500672 | 0.97909037 |
| O14976 | Cyclin-G-associated kinase | GAK | Homo sapiens | 1.326903913 | -0.444707439 | 0.414425446 | -1.07306982 | 0.283239791 | NA |
| P35080 | Profilin-2 | PFN2 | Homo sapiens | 0.942346558 | 0.491447649 | 0.458475571 | 1.071916761 | 0.283757421 | NA |
| Q7L2H7 | Eukaryotic translation initiation factor 3 subunit M | EIF3M | Homo sapiens | 1.182297176 | 0.460021136 | 0.429558572 | 1.070915972 | 0.284207214 | NA |
| Q9NR12 | PDZ and LIM domain protein 7 | PDLIM7 | Homo sapiens | 3.744003093 | 0.25358206 | 0.2368069 | 1.070838985 | 0.284241834 | 0.97909037 |
| P29144 | Tripeptidyl-peptidase 2 | TPP2 | Homo sapiens | 3.146952205 | -0.274523044 | 0.256711925 | -1.069381738 | 0.284897693 | 0.97909037 |
| P15311 | Ezrin | EZR | Homo sapiens | 37.47024148 | 0.075142894 | 0.070347719 | 1.068163905 | 0.285446583 | 0.97909037 |
| Q06323 | Proteasome activator complex subunit 1 | PSME1 | Homo sapiens | 2.506365185 | -0.309595928 | 0.289879456 | -1.068016108 | 0.285513246 | 0.97909037 |
| P05386 | 60S acidic ribosomal protein P1 | RPLP1 | Homo sapiens | 2.132478532 | 0.321437639 | 0.301646018 | 1.065612077 | 0.286599038 | 0.97909037 |
| P62277 | 40S ribosomal protein S13 | RPS13 | Homo sapiens | 9.833205175 | -0.14568159 | 0.136749416 | -1.065317816 | 0.286732133 | 0.97909037 |
| P18206 | Vinculin | VCL | Homo sapiens | 63.7977701 | -0.101685236 | 0.095511259 | -1.064641355 | 0.287038258 | 0.97909037 |
| Q9NZB2 | Constitutive coactivator of PPAR-gamma-like protein 1 | FAM120A | Homo sapiens | 0.895976994 | -0.574608026 | 0.540269511 | -1.063558122 | 0.287528922 | NA |
| P06733 | Alpha-enolase | ENO1 | Homo sapiens | 164.8465809 | -0.07062155 | 0.066450775 | -1.062764879 | 0.287888591 | 0.97909037 |
| P08237 | ATP-dependent 6-phosphofructokinase, muscle type | PFKM | Homo sapiens | 1.928695173 | -0.354235502 | 0.333821657 | -1.061151949 | 0.288620852 | 0.97909037 |
| P68366 | Tubulin alpha-4A chain | TUBA4A | Homo sapiens | 228.8866799 | -0.038164949 | 0.035976724 | -1.060823368 | 0.28877018 | 0.97909037 |
| Q9HCJ6 | Synaptic vesicle membrane protein VAT-1 homolog-like | VAT1L | Homo sapiens | 3.11706753 | 0.299066668 | 0.282635748 | 1.058134615 | 0.289994075 | 0.97909037 |
| P04439 | HLA class I histocompatibility antigen, A alpha chain | HLA-A | Homo sapiens | 6.417075213 | -0.185122585 | 0.175019065 | -1.057728111 | 0.290179415 | 0.97909037 |
| P62140 | Serine/threonine-protein phosphatase PP1-beta catalytic subunit | PPP1CB | Homo sapiens | 2.878023068 | -0.39224599 | 0.371214579 | -1.056655673 | 0.290668762 | 0.97909037 |
| Q9Y2J2 | Band 4.1-like protein 3 | EPB41L3 | Homo sapiens | 1.219034221 | 0.430849913 | 0.408153927 | 1.055606438 | 0.291148058 | NA |
| Q96HC4 | PDZ and LIM domain protein 5 | PDLIM5 | Homo sapiens | 8.884450966 | -0.156762826 | 0.148709495 | -1.054154786 | 0.291812057 | 0.97909037 |
| P12270 | Nucleoprotein TPR | TPR | Homo sapiens | 4.132229965 | 0.235621982 | 0.224085648 | 1.05148181 | 0.293037359 | 0.97909037 |
| P09382 | Galectin-1 | LGALS1 | Homo sapiens | 120.4314102 | -0.044889493 | 0.042801253 | -1.048789224 | 0.294275138 | 0.97909037 |
| P43243 | Matrin-3 | MATR3 | Homo sapiens | 20.25107852 | 0.101215096 | 0.09652118 | 1.04863095 | 0.294348006 | 0.97909037 |
| Q16822 | Phosphoenolpyruvate carboxykinase [GTP], mitochondrial | PCK2 | Homo sapiens | 0.924650163 | 0.498548165 | 0.475452636 | 1.048575878 | 0.294373363 | NA |
| P09651 | Heterogeneous nuclear ribonucleoprotein A1 | HNRNPA1 | Homo sapiens | 43.36738979 | 0.069415617 | 0.066251812 | 1.047754238 | 0.294751852 | 0.97909037 |
| Q00839 | Heterogeneous nuclear ribonucleoprotein U | HNRNPU | Homo sapiens | 44.10220697 | 0.068680975 | 0.065595635 | 1.047035764 | 0.295083084 | 0.97909037 |
| P39656 | Dolichyl-diphosphooligosaccharide--protein glycosyltransferase 48 kDa subunit | DDOST | Homo sapiens | 11.97312845 | -0.1382226 | 0.132132628 | -1.046089844 | 0.295519554 | 0.97909037 |
| P27824 | Calnexin | CANX | Homo sapiens | 22.49893821 | -0.105498058 | 0.100903662 | -1.045532499 | 0.295776928 | 0.97909037 |
| O60701 | UDP-glucose 6-dehydrogenase | UGDH | Homo sapiens | 46.34222258 | 0.069343072 | 0.066351636 | 1.045084581 | 0.29598388 | 0.97909037 |
| Q9P2J5 | Leucine--tRNA ligase, cytoplasmic | LARS1 | Homo sapiens | 13.55677403 | 0.122341034 | 0.117497307 | 1.041224155 | 0.297771527 | 0.979255119 |
| Q96HE7 | ERO1-like protein alpha | ERO1A | Homo sapiens | 3.23195127 | -0.262079565 | 0.252878437 | -1.036385577 | 0.300022296 | 0.979255119 |
| O60711 | Leupaxin | LPXN | Homo sapiens | 0.758414366 | -0.640234364 | 0.618193261 | -1.035654067 | 0.300363559 | NA |
| Q71UM5 | 40S ribosomal protein S27-like | RPS27L | Homo sapiens | 1.40464671 | -0.3804247 | 0.367473971 | -1.035242574 | 0.300555641 | NA |
| Q08945 | FACT complex subunit SSRP1 | SSRP1 | Homo sapiens | 5.159378773 | 0.201062713 | 0.194390398 | 1.034324305 | 0.300984578 | 0.979255119 |
| Q9H0A0 | RNA cytidine acetyltransferase | NAT10 | Homo sapiens | 0.817666824 | 0.510631698 | 0.493807956 | 1.034069403 | 0.301103719 | NA |
| P23142 | Fibulin-1 | FBLN1 | Homo sapiens | 10.88765845 | -0.134846885 | 0.13069456 | -1.031771218 | 0.302179305 | 0.979255119 |
| Q92901 | 60S ribosomal protein L3-like | RPL3L | Homo sapiens | 1.32258074 | -0.376054891 | 0.364534867 | -1.031601981 | 0.302258612 | NA |
| P17812 | CTP synthase 1 | CTPS1 | Homo sapiens | 11.96737511 | -0.133789615 | 0.130110486 | -1.028276957 | 0.303819567 | 0.979255119 |
| O15144 | Actin-related protein 2/3 complex subunit 2 | ARPC2 | Homo sapiens | 3.831335067 | -0.245493917 | 0.238785361 | -1.028094501 | 0.303905377 | 0.979255119 |
| Q63ZY3 | KN motif and ankyrin repeat domain-containing protein 2 | KANK2 | Homo sapiens | 2.300567367 | 0.310770805 | 0.302797927 | 1.026330689 | 0.304735736 | 0.979255119 |
| P07384 | Calpain-1 catalytic subunit | CAPN1 | Homo sapiens | 11.96893068 | -0.133357302 | 0.129938152 | -1.026313669 | 0.304743756 | 0.979255119 |
| P62873 | Guanine nucleotide-binding protein G(I)/G(S)/G(T) subunit beta-1 | GNB1 | Homo sapiens | 9.721961402 | -0.144225436 | 0.140796048 | -1.024357134 | 0.305666621 | 0.979255119 |
| P30566 | Adenylosuccinate lyase | ADSL | Homo sapiens | 1.345282488 | 0.382868416 | 0.373801271 | 1.024256592 | 0.305714095 | NA |
| Q16531 | DNA damage-binding protein 1 | DDB1 | Homo sapiens | 11.67999424 | 0.129750068 | 0.126681709 | 1.024221012 | 0.305730897 | 0.979255119 |
| Q9UKV3 | Apoptotic chromatin condensation inducer in the nucleus | ACIN1 | Homo sapiens | 0.955450895 | 0.487881188 | 0.476503432 | 1.023877597 | 0.305893093 | NA |
| P16989 | Y-box-binding protein 3 | YBX3 | Homo sapiens | 12.27706202 | 0.126779117 | 0.123860508 | 1.023563674 | 0.30604141 | 0.979255119 |
| Q8IY81 | pre-rRNA 2'-O-ribose RNA methyltransferase FTSJ3 | FTSJ3 | Homo sapiens | 0.667841091 | 0.568535918 | 0.555940672 | 1.022655738 | 0.306470646 | NA |
| Q14651 | Plastin-1 | PLS1 | Homo sapiens | 2.161185671 | -0.309991788 | 0.303242755 | -1.022256203 | 0.306659656 | 0.979255119 |
| Q92544 | Transmembrane 9 superfamily member 4 | TM9SF4 | Homo sapiens | 0.985004927 | 0.438146711 | 0.428851154 | 1.021675486 | 0.306934517 | NA |
| P19525 | Interferon-induced, double-stranded RNA-activated protein kinase | EIF2AK2 | Homo sapiens | 3.409698412 | 0.258572302 | 0.253497801 | 1.02001793 | 0.307719958 | 0.979255119 |
| Q13642 | Four and a half LIM domains protein 1 | FHL1 | Homo sapiens | 3.040372279 | -0.253453493 | 0.248719746 | -1.019032454 | 0.30818756 | 0.979255119 |
| Q08752 | Peptidyl-prolyl cis-trans isomerase D | PPID | Homo sapiens | 1.573422788 | 0.41782386 | 0.412605457 | 1.012647441 | 0.311228591 | NA |
| P21333 | Filamin-A | FLNA | Homo sapiens | 409.7426403 | -0.036695319 | 0.036300008 | -1.010890092 | 0.312069037 | 0.980182922 |
| O43670 | BUB3-interacting and GLEBS motif-containing protein ZNF207 | ZNF207 | Homo sapiens | 1.191191077 | 0.402666688 | 0.398908222 | 1.009421883 | 0.312772348 | NA |
| Q8IV08 | 5'-3' exonuclease PLD3 | PLD3 | Homo sapiens | 1.407591789 | -0.37545641 | 0.372907627 | -1.006834891 | 0.314014125 | NA |
| P11142 | Heat shock cognate 71 kDa protein | HSPA8 | Homo sapiens | 209.8819549 | 0.032231862 | 0.032061402 | 1.005316671 | 0.31474439 | 0.980182922 |
| O15143 | Actin-related protein 2/3 complex subunit 1B | ARPC1B | Homo sapiens | 16.38536969 | -0.108506492 | 0.107963461 | -1.005029769 | 0.314882515 | 0.980182922 |
| Q04760 | Lactoylglutathione lyase | GLO1 | Homo sapiens | 1.358104359 | -0.415278029 | 0.413372373 | -1.004610021 | 0.31508467 | NA |
| P25789 | Proteasome subunit alpha type-4 | PSMA4 | Homo sapiens | 5.813207839 | 0.192318983 | 0.191666815 | 1.003402612 | 0.315666644 | 0.980182922 |
| P27105 | Stomatin | STOM | Homo sapiens | 4.134433513 | -0.233357168 | 0.232858074 | -1.002143338 | 0.316274369 | 0.980182922 |
| Q15046 | Lysine--tRNA ligase | KARS1 | Homo sapiens | 3.994862422 | 0.224911325 | 0.224556391 | 1.001580598 | 0.316546196 | 0.980182922 |
| P46778 | 60S ribosomal protein L21 | RPL21 | Homo sapiens | 6.373551512 | 0.172656101 | 0.172508295 | 1.000856807 | 0.316896041 | 0.980182922 |
| P25685 | DnaJ homolog subfamily B member 1 | DNAJB1 | Homo sapiens | 4.184748777 | 0.216065491 | 0.215980267 | 1.000394591 | 0.317119586 | 0.980182922 |
| P46108 | Adapter molecule crk | CRK | Homo sapiens | 3.941337832 | -0.218550787 | 0.218671811 | -0.99944655 | 0.317578419 | 0.980182922 |
| P50995 | Annexin A11 | ANXA11 | Homo sapiens | 5.502429831 | -0.208930597 | 0.209212405 | -0.998653008 | 0.317962812 | 0.980182922 |
| Q9NQC3 | Reticulon-4 | RTN4 | Homo sapiens | 11.98466379 | -0.133612977 | 0.134000642 | -0.997106994 | 0.318712578 | 0.980182922 |
| A2RTX5 | Threonine--tRNA ligase 2, cytoplasmic | TARS3 | Homo sapiens | 0.829906174 | -0.493719659 | 0.49563469 | -0.996136205 | 0.319183971 | NA |
| P20618 | Proteasome subunit beta type-1 | PSMB1 | Homo sapiens | 2.112648807 | 0.307430692 | 0.308931084 | 0.99514328 | 0.319666584 | 0.980182922 |
| P14678 | Small nuclear ribonucleoprotein-associated proteins B and B' | SNRPB | Homo sapiens | 2.491750458 | 0.279703068 | 0.28160329 | 0.99325213 | 0.3205871 | 0.980182922 |
| A0AVT1 | Ubiquitin-like modifier-activating enzyme 6 | UBA6 | Homo sapiens | 3.80007492 | -0.230036091 | 0.231626576 | -0.993133408 | 0.320644945 | 0.980182922 |
| P16949 | Stathmin | STMN1 | Homo sapiens | 1.14067116 | 0.398885331 | 0.40354173 | 0.98846117 | 0.322926842 | NA |
| Q02952 | A-kinase anchor protein 12 | AKAP12 | Homo sapiens | 10.35397796 | 0.168719539 | 0.170705094 | 0.988368506 | 0.322972206 | 0.984628779 |
| Q9Y4E8 | Ubiquitin carboxyl-terminal hydrolase 15 | USP15 | Homo sapiens | 0.774742 | 0.467536629 | 0.476414577 | 0.981365079 | 0.326412741 | NA |
| Q99733 | Nucleosome assembly protein 1-like 4 | NAP1L4 | Homo sapiens | 3.086766682 | 0.246699688 | 0.25184269 | 0.979578515 | 0.327294215 | 0.988988648 |
| Q8WXF1 | Paraspeckle component 1 | PSPC1 | Homo sapiens | 1.311126368 | 0.380404792 | 0.389148312 | 0.977531651 | 0.328306016 | NA |
| P01023 | Alpha-2-macroglobulin | A2M | Homo sapiens | 10.55544524 | -0.130801824 | 0.134083044 | -0.975528446 | 0.329298198 | 0.988988648 |
| P25398 | 40S ribosomal protein S12 | RPS12 | Homo sapiens | 12.06660083 | 0.122654134 | 0.125873698 | 0.974422263 | 0.32984692 | 0.988988648 |
| P25787 | Proteasome subunit alpha type-2 | PSMA2 | Homo sapiens | 11.22586458 | -0.125301327 | 0.12891011 | -0.972005426 | 0.331047849 | 0.988988648 |
| P35221 | Catenin alpha-1 | CTNNA1 | Homo sapiens | 14.19279305 | -0.123787505 | 0.12739646 | -0.971671463 | 0.331214018 | 0.988988648 |
| P08758 | Annexin A5 | ANXA5 | Homo sapiens | 23.55529065 | -0.093657527 | 0.096474501 | -0.970800847 | 0.331647461 | 0.988988648 |
| P23258 | Tubulin gamma-1 chain | TUBG1 | Homo sapiens | 1.113473039 | 0.412388962 | 0.426604433 | 0.966677629 | 0.333705214 | NA |
| Q96AY3 | Peptidyl-prolyl cis-trans isomerase FKBP10 | FKBP10 | Homo sapiens | 17.02000559 | -0.104605325 | 0.108918179 | -0.960402813 | 0.336852523 | 0.988988648 |
| Q99426 | Tubulin-folding cofactor B | TBCB | Homo sapiens | 1.440928017 | 0.347607266 | 0.362315124 | 0.959405896 | 0.337354306 | NA |
| P18085 | ADP-ribosylation factor 4 | ARF4 | Homo sapiens | 28.4556575 | -0.078492535 | 0.081886821 | -0.95854906 | 0.337785965 | 0.988988648 |
| Q13045 | Protein flightless-1 homolog | FLII | Homo sapiens | 5.107087403 | 0.184346747 | 0.192444501 | 0.957921615 | 0.338102286 | 0.988988648 |
| Q9NZM1 | Myoferlin | MYOF | Homo sapiens | 63.34601294 | 0.060299712 | 0.063075986 | 0.955985255 | 0.339079684 | 0.988988648 |
| P07864 | L-lactate dehydrogenase C chain | LDHC | Homo sapiens | 1.993397676 | -0.306138479 | 0.320328867 | -0.955700566 | 0.339223536 | 0.988988648 |
| P13473 | Lysosome-associated membrane glycoprotein 2 | LAMP2 | Homo sapiens | 10.83906376 | -0.129318867 | 0.135409759 | -0.955018807 | 0.339568186 | 0.988988648 |
| P33993 | DNA replication licensing factor MCM7 | MCM7 | Homo sapiens | 5.741894391 | 0.173575277 | 0.181941163 | 0.954018731 | 0.340074161 | 0.988988648 |
| P52594 | Arf-GAP domain and FG repeat-containing protein 1 | AGFG1 | Homo sapiens | 1.074460853 | 0.396626785 | 0.416025657 | 0.953370973 | 0.340402143 | NA |
| P52943 | Cysteine-rich protein 2 | CRIP2 | Homo sapiens | 2.561524426 | 0.273192071 | 0.286616593 | 0.953162088 | 0.340507952 | 0.988988648 |
| O95292 | Vesicle-associated membrane protein-associated protein B/C | VAPB | Homo sapiens | 1.457360277 | 0.33390949 | 0.350568252 | 0.952480689 | 0.340853255 | NA |
| O15067 | Phosphoribosylformylglycinamidine synthase | PFAS | Homo sapiens | 1.303469853 | 0.390888358 | 0.412619711 | 0.947333215 | 0.34346901 | NA |
| P34897 | Serine hydroxymethyltransferase, mitochondrial | SHMT2 | Homo sapiens | 4.262220577 | 0.222952035 | 0.236034325 | 0.944574629 | 0.344876085 | 0.988988648 |
| P62258 | 14-3-3 protein epsilon | YWHAE | Homo sapiens | 23.72697812 | -0.082735458 | 0.087677778 | -0.943630872 | 0.34535831 | 0.988988648 |
| P08754 | Guanine nucleotide-binding protein G(i) subunit alpha-3 | GNAI3 | Homo sapiens | 1.042169 | 0.434813871 | 0.461476461 | 0.942223294 | 0.34607833 | NA |
| Q9HD67 | Unconventional myosin-X | MYO10 | Homo sapiens | 0.553346294 | -0.61421109 | 0.654490497 | -0.938456849 | 0.348009682 | NA |
| Q7KZF4 | Staphylococcal nuclease domain-containing protein 1 | SND1 | Homo sapiens | 37.75322901 | -0.067011785 | 0.071431027 | -0.93813274 | 0.348176198 | 0.988988648 |
| Q09028 | Histone-binding protein RBBP4 | RBBP4 | Homo sapiens | 5.768200931 | 0.173157509 | 0.184712334 | 0.937444213 | 0.348530107 | 0.988988648 |
| Q9Y277 | Voltage-dependent anion-selective channel protein 3 | VDAC3 | Homo sapiens | 6.471623292 | -0.15826099 | 0.168827641 | -0.937411605 | 0.348546874 | 0.988988648 |
| P52597 | Heterogeneous nuclear ribonucleoprotein F | HNRNPF | Homo sapiens | 22.18202526 | 0.085026561 | 0.090785896 | 0.936561343 | 0.348984244 | 0.988988648 |
| P63172 | Dynein light chain Tctex-type 1 | DYNLT1 | Homo sapiens | 1.187792843 | 0.384836648 | 0.411027371 | 0.936279858 | 0.349129116 | NA |
| P53618 | Coatomer subunit beta | COPB1 | Homo sapiens | 24.63099986 | 0.082305553 | 0.087981165 | 0.935490603 | 0.349535524 | 0.988988648 |
| Q12907 | Vesicular integral-membrane protein VIP36 | LMAN2 | Homo sapiens | 3.061797796 | -0.257454497 | 0.275654953 | -0.933973775 | 0.350317421 | 0.988988648 |
| Q14019 | Coactosin-like protein | COTL1 | Homo sapiens | 9.586048012 | -0.133561286 | 0.143074007 | -0.933511885 | 0.350555737 | 0.988988648 |
| Q96S97 | Myeloid-associated differentiation marker | MYADM | Homo sapiens | 6.166692101 | -0.166147788 | 0.178033234 | -0.933240296 | 0.350695914 | 0.988988648 |
| Q12905 | Interleukin enhancer-binding factor 2 | ILF2 | Homo sapiens | 16.64162864 | 0.102246343 | 0.10963633 | 0.93259546 | 0.351028878 | 0.988988648 |
| P11217 | Glycogen phosphorylase, muscle form | PYGM | Homo sapiens | 1.794798273 | 0.30904641 | 0.331635619 | 0.931885456 | 0.351395725 | 0.988988648 |
| P35241 | Radixin | RDX | Homo sapiens | 30.17015185 | -0.072887464 | 0.078218522 | -0.931844047 | 0.351417127 | 0.988988648 |
| O94925 | Glutaminase kidney isoform, mitochondrial | GLS | Homo sapiens | 15.54469675 | -0.103943642 | 0.111685191 | -0.930684197 | 0.352016946 | 0.988988648 |
| Q02750 | Dual specificity mitogen-activated protein kinase kinase 1 | MAP2K1 | Homo sapiens | 2.123099294 | 0.265038205 | 0.285507979 | 0.928304022 | 0.353249888 | 0.988988648 |
| Q15043 | Metal cation symporter ZIP14 | SLC39A14 | Homo sapiens | 2.947743136 | 0.237959624 | 0.256622053 | 0.927276594 | 0.353782943 | 0.988988648 |
| P62633 | Cellular nucleic acid-binding protein | CNBP | Homo sapiens | 2.902028891 | 0.230951543 | 0.249273473 | 0.926498678 | 0.354186884 | 0.988988648 |
| P26358 | DNA (cytosine-5)-methyltransferase 1 | DNMT1 | Homo sapiens | 2.185195456 | 0.290454867 | 0.314242598 | 0.92430138 | 0.355329425 | 0.988988648 |
| P43307 | Translocon-associated protein subunit alpha | SSR1 | Homo sapiens | 12.33230055 | -0.113867127 | 0.123291735 | -0.923558472 | 0.355716244 | 0.988988648 |
| P42224 | Signal transducer and activator of transcription 1-alpha/beta | STAT1 | Homo sapiens | 21.97635706 | -0.10824871 | 0.117322586 | -0.922658749 | 0.356185069 | 0.988988648 |
| Q9P258 | Protein RCC2 | RCC2 | Homo sapiens | 1.365830562 | -0.332611192 | 0.361589522 | -0.919858492 | 0.357646712 | NA |
| P35052 | Glypican-1 | GPC1 | Homo sapiens | 0.821722923 | 0.457295407 | 0.499592937 | 0.915336013 | 0.360015259 | NA |
| P49755 | Transmembrane emp24 domain-containing protein 10 | TMED10 | Homo sapiens | 2.027405267 | -0.285981237 | 0.313421556 | -0.912449164 | 0.361532319 | 0.988988648 |
| P50479 | PDZ and LIM domain protein 4 | PDLIM4 | Homo sapiens | 6.776616539 | -0.163097577 | 0.178788419 | -0.912237926 | 0.361643483 | 0.988988648 |
| P13861 | cAMP-dependent protein kinase type II-alpha regulatory subunit | PRKAR2A | Homo sapiens | 1.39361117 | 0.340724416 | 0.373759808 | 0.91161331 | 0.361972313 | NA |
| O00264 | Membrane-associated progesterone receptor component 1 | PGRMC1 | Homo sapiens | 1.468592862 | 0.365496068 | 0.401729555 | 0.90980627 | 0.362924687 | NA |
| P49327 | Fatty acid synthase | FASN | Homo sapiens | 81.12595933 | -0.046772086 | 0.051417773 | -0.90964823 | 0.363008054 | 0.988988648 |
| P18077 | 60S ribosomal protein L35a | RPL35A | Homo sapiens | 2.171859845 | -0.278348751 | 0.306650939 | -0.907705522 | 0.364033826 | 0.988988648 |
| Q9UBG0 | C-type mannose receptor 2 | MRC2 | Homo sapiens | 9.32624469 | -0.138990975 | 0.15336611 | -0.906269155 | 0.364793407 | 0.988988648 |
| P22492 | Histone H1t | H1-6 | Homo sapiens | 2.466552565 | 0.265272923 | 0.29366074 | 0.903331249 | 0.366350116 | 0.988988648 |
| P02751 | Fibronectin | FN1 | Homo sapiens | 89.90162441 | -0.091652909 | 0.101666728 | -0.901503476 | 0.367320686 | 0.988988648 |
| Q14978 | Nucleolar and coiled-body phosphoprotein 1 | NOLC1 | Homo sapiens | 2.560768761 | 0.248825313 | 0.276534733 | 0.899797685 | 0.368227927 | 0.988988648 |
| Q9BZK3 | Putative nascent polypeptide-associated complex subunit alpha-like protein | NACA4P | Homo sapiens | 0.774401619 | 0.408780188 | 0.454961347 | 0.898494323 | 0.36892207 | NA |
| O75874 | Isocitrate dehydrogenase [NADP] cytoplasmic | IDH1 | Homo sapiens | 13.01363708 | -0.108649473 | 0.121191489 | -0.896510753 | 0.36998004 | 0.988988648 |
| O00410 | Importin-5 | IPO5 | Homo sapiens | 21.60832574 | -0.081664372 | 0.091265384 | -0.894801167 | 0.370893386 | 0.988988648 |
| P47756 | F-actin-capping protein subunit beta | CAPZB | Homo sapiens | 18.04891815 | 0.091565387 | 0.10242276 | 0.893994531 | 0.371324817 | 0.988988648 |
| Q13243 | Serine/arginine-rich splicing factor 5 | SRSF5 | Homo sapiens | 1.0735264 | -0.37870972 | 0.424036054 | -0.893107359 | 0.371799683 | NA |
| Q9UKE5 | TRAF2 and NCK-interacting protein kinase | TNIK | Homo sapiens | 1.41531882 | 0.334316532 | 0.375196168 | 0.891044633 | 0.372905226 | NA |
| O95757 | Heat shock 70 kDa protein 4L | HSPA4L | Homo sapiens | 4.582959533 | 0.190748621 | 0.214131952 | 0.890799432 | 0.37303678 | 0.988988648 |
| O94776 | Metastasis-associated protein MTA2 | MTA2 | Homo sapiens | 0.827714025 | 0.474100043 | 0.53486649 | 0.886389504 | 0.375407673 | NA |
| P51116 | Fragile X mental retardation syndrome-related protein 2 | FXR2 | Homo sapiens | 1.600105158 | 0.336319286 | 0.380147625 | 0.884707057 | 0.376314649 | NA |
| Q8IYB3 | Serine/arginine repetitive matrix protein 1 | SRRM1 | Homo sapiens | 1.10418431 | 0.398478782 | 0.451369117 | 0.882822432 | 0.377332219 | NA |
| Q12849 | G-rich sequence factor 1 | GRSF1 | Homo sapiens | 0.976747807 | 0.402398529 | 0.455856112 | 0.882731455 | 0.377381383 | NA |
| A1X283 | SH3 and PX domain-containing protein 2B | SH3PXD2B | Homo sapiens | 0.792140496 | -0.446803562 | 0.508810153 | -0.878134132 | 0.379870932 | NA |
| Q7L0X0 | TLR4 interactor with leucine rich repeats | TRIL | Homo sapiens | 0.715146111 | 0.534668413 | 0.609369726 | 0.877412169 | 0.380262806 | NA |
| Q00325 | Phosphate carrier protein, mitochondrial | SLC25A3 | Homo sapiens | 12.18639399 | -0.105987722 | 0.120914733 | -0.876549273 | 0.380731502 | 0.988988648 |
| Q8IWE2 | Protein NOXP20 | FAM114A1 | Homo sapiens | 4.784083155 | 0.177972153 | 0.203983453 | 0.872483285 | 0.382944783 | 0.988988648 |
| P31946 | 14-3-3 protein beta/alpha | YWHAB | Homo sapiens | 26.29001078 | -0.07237411 | 0.083228432 | -0.869583969 | 0.384527802 | 0.988988648 |
| O43823 | A-kinase anchor protein 8 | AKAP8 | Homo sapiens | 1.220772951 | 0.336848446 | 0.38746172 | 0.869372196 | 0.384643586 | NA |
| O14907 | Tax1-binding protein 3 | TAX1BP3 | Homo sapiens | 0.843245437 | -0.404255579 | 0.465224152 | -0.868947963 | 0.384875594 | NA |
| P00492 | Hypoxanthine-guanine phosphoribosyltransferase | HPRT1 | Homo sapiens | 2.737382968 | 0.230079472 | 0.26479324 | 0.868902364 | 0.384900537 | 0.988988648 |
| P83731 | 60S ribosomal protein L24 | RPL24 | Homo sapiens | 14.53221503 | 0.098823327 | 0.113788845 | 0.868479923 | 0.385131659 | 0.988988648 |
| O75475 | PC4 and SFRS1-interacting protein | PSIP1 | Homo sapiens | 0.836950567 | -0.383673203 | 0.442367811 | -0.867317182 | 0.385768245 | NA |
| Q9BQE3 | Tubulin alpha-1C chain | TUBA1C | Homo sapiens | 305.0935032 | -0.027626557 | 0.031857986 | -0.867178371 | 0.385844285 | 0.988988648 |
| Q13423 | NAD(P) transhydrogenase, mitochondrial | NNT | Homo sapiens | 1.729340798 | 0.312594828 | 0.360790567 | 0.8664163 | 0.386261908 | 0.988988648 |
| P62805 | Histone H4 | H4C1 | Homo sapiens | 14.05262161 | 0.108899947 | 0.12572526 | 0.866173968 | 0.386394767 | 0.988988648 |
| Q07866 | Kinesin light chain 1 | KLC1 | Homo sapiens | 6.252644202 | 0.150806645 | 0.174504332 | 0.864200008 | 0.387478028 | 0.988988648 |
| P08238 | Heat shock protein HSP 90-beta | HSP90AB1 | Homo sapiens | 212.5447269 | 0.028890817 | 0.033515744 | 0.862007317 | 0.388683492 | 0.988988648 |
| P02533 | Keratin, type I cytoskeletal 14 | KRT14 | Homo sapiens | 2.026726389 | -0.301469576 | 0.349837699 | -0.86174125 | 0.388829921 | 0.988988648 |
| P0DMV8 | Heat shock 70 kDa protein 1A | HSPA1A | Homo sapiens | 42.07296779 | 0.0796371 | 0.09248551 | 0.861076514 | 0.389195903 | 0.988988648 |
| P60981 | Destrin | DSTN | Homo sapiens | 7.704574909 | 0.133619597 | 0.155283784 | 0.86048648 | 0.389520933 | 0.988988648 |
| P35237 | Serpin B6 | SERPINB6 | Homo sapiens | 4.795010679 | -0.175028536 | 0.203689531 | -0.859290782 | 0.390180109 | 0.988988648 |
| P23381 | Tryptophan--tRNA ligase, cytoplasmic | WARS1 | Homo sapiens | 14.92396506 | -0.100445279 | 0.117188707 | -0.857124218 | 0.391376238 | 0.988988648 |
| P47755 | F-actin-capping protein subunit alpha-2 | CAPZA2 | Homo sapiens | 8.458763829 | -0.126095764 | 0.147192285 | -0.856673727 | 0.391625227 | 0.988988648 |
| Q9UBE0 | SUMO-activating enzyme subunit 1 | SAE1 | Homo sapiens | 3.665145445 | 0.197095771 | 0.230156791 | 0.856354358 | 0.391801802 | 0.988988648 |
| P35527 | Keratin, type I cytoskeletal 9 | KRT9 | Homo sapiens | 28.4687102 | -0.135820042 | 0.158625301 | -0.856231892 | 0.391869525 | 0.988988648 |
| Q01650 | Large neutral amino acids transporter small subunit 1 | SLC7A5 | Homo sapiens | 1.885816428 | 0.281406601 | 0.32890353 | 0.85559009 | 0.392224553 | 0.988988648 |
| Q92598 | Heat shock protein 105 kDa | HSPH1 | Homo sapiens | 30.18083714 | 0.073321895 | 0.085843322 | 0.854136267 | 0.393029491 | 0.988988648 |
| Q92572 | AP-3 complex subunit sigma-1 | AP3S1 | Homo sapiens | 1.197735671 | -0.329178409 | 0.388063775 | -0.848258535 | 0.396294004 | NA |
| P46776 | 60S ribosomal protein L27a | RPL27A | Homo sapiens | 6.492814077 | 0.144976871 | 0.17117121 | 0.846969948 | 0.397011872 | 0.988988648 |
| O14744 | Protein arginine N-methyltransferase 5 | PRMT5 | Homo sapiens | 0.78226877 | 0.445734327 | 0.527681913 | 0.844702681 | 0.398276862 | NA |
| P37802 | Transgelin-2 | TAGLN2 | Homo sapiens | 47.34633291 | -0.053304409 | 0.063107835 | -0.844655951 | 0.39830296 | 0.988988648 |
| Q9UKK9 | ADP-sugar pyrophosphatase | NUDT5 | Homo sapiens | 1.069593765 | -0.380466549 | 0.450553667 | -0.844442241 | 0.398422326 | NA |
| O95302 | Peptidyl-prolyl cis-trans isomerase FKBP9 | FKBP9 | Homo sapiens | 2.689127067 | 0.252389721 | 0.300030915 | 0.841212383 | 0.400228965 | 0.988988648 |
| Q13283 | Ras GTPase-activating protein-binding protein 1 | G3BP1 | Homo sapiens | 20.95184036 | 0.079514049 | 0.094803448 | 0.838725284 | 0.401623487 | 0.988988648 |
| P45880 | Voltage-dependent anion-selective channel protein 2 | VDAC2 | Homo sapiens | 20.01188748 | -0.081918684 | 0.097856416 | -0.837131454 | 0.402518682 | 0.988988648 |
| Q9UBT7 | Alpha-catulin | CTNNAL1 | Homo sapiens | 0.530554531 | -0.703531785 | 0.840886991 | -0.83665438 | 0.402786869 | NA |
| P62942 | Peptidyl-prolyl cis-trans isomerase FKBP1A | FKBP1A | Homo sapiens | 1.130820381 | 0.409100723 | 0.489126253 | 0.836390851 | 0.402935058 | NA |
| Q13418 | Integrin-linked protein kinase | ILK | Homo sapiens | 3.728642492 | -0.186989669 | 0.223599643 | -0.836269983 | 0.403003036 | 0.988988648 |
| P46977 | Dolichyl-diphosphooligosaccharide--protein glycosyltransferase subunit STT3A | STT3A | Homo sapiens | 4.29806596 | -0.172822436 | 0.207288528 | -0.833728897 | 0.404433771 | 0.988988648 |
| Q15036 | Sorting nexin-17 | SNX17 | Homo sapiens | 0.828024601 | -0.400631491 | 0.481066891 | -0.832797888 | 0.404958726 | NA |
| P35232 | Prohibitin | PHB | Homo sapiens | 9.274912089 | -0.118385328 | 0.142593091 | -0.830231863 | 0.406407704 | 0.988988648 |
| P50502 | Hsc70-interacting protein | ST13 | Homo sapiens | 5.342869181 | -0.180212801 | 0.217421052 | -0.828865462 | 0.407180541 | 0.988988648 |
| O60518 | Ran-binding protein 6 | RANBP6 | Homo sapiens | 2.587751486 | -0.221529773 | 0.26743852 | -0.828339063 | 0.407478506 | 0.988988648 |
| Q14677 | Clathrin interactor 1 | CLINT1 | Homo sapiens | 1.344865861 | 0.338934035 | 0.409397244 | 0.827885484 | 0.407735357 | NA |
| P42285 | Exosome RNA helicase MTR4 | MTREX | Homo sapiens | 2.037233355 | 0.257711471 | 0.311419539 | 0.827537896 | 0.407932252 | 0.988988648 |
| Q9UNZ2 | NSFL1 cofactor p47 | NSFL1C | Homo sapiens | 4.658229274 | 0.165689245 | 0.201254249 | 0.823283212 | 0.410346958 | 0.988988648 |
| P68032 | Actin, alpha cardiac muscle 1 | ACTC1 | Homo sapiens | 158.4133669 | -0.031904658 | 0.038801758 | -0.822247744 | 0.410935911 | 0.988988648 |
| P39019 | 40S ribosomal protein S19 | RPS19 | Homo sapiens | 5.371528856 | 0.156137668 | 0.190011871 | 0.821725858 | 0.411232939 | 0.988988648 |
| Q9UBS4 | DnaJ homolog subfamily B member 11 | DNAJB11 | Homo sapiens | 2.036009121 | 0.251204081 | 0.306900588 | 0.818519387 | 0.413060677 | 0.988988648 |
| Q5XKE5 | Keratin, type II cytoskeletal 79 | KRT79 | Homo sapiens | 1.06290925 | 0.347503978 | 0.424800586 | 0.818040251 | 0.413334204 | NA |
| Q96PK6 | RNA-binding protein 14 | RBM14 | Homo sapiens | 3.400521757 | -0.19127211 | 0.233886138 | -0.817800111 | 0.413471335 | 0.988988648 |
| Q92783 | Signal transducing adapter molecule 1 | STAM | Homo sapiens | 1.160702888 | 0.358067351 | 0.438034639 | 0.81744072 | 0.413676613 | NA |
| Q9NQW7 | Xaa-Pro aminopeptidase 1 | XPNPEP1 | Homo sapiens | 5.782786341 | -0.146103001 | 0.178764426 | -0.817293489 | 0.413760727 | 0.988988648 |
| Q9BTV4 | Transmembrane protein 43 | TMEM43 | Homo sapiens | 4.764091977 | -0.165049113 | 0.20225046 | -0.816062981 | 0.414464115 | 0.988988648 |
| Q6NUQ4 | Transmembrane protein 214 | TMEM214 | Homo sapiens | 0.68700645 | 0.452318637 | 0.555269681 | 0.814592714 | 0.415305483 | NA |
| Q92499 | ATP-dependent RNA helicase DDX1 | DDX1 | Homo sapiens | 21.64117711 | -0.076705807 | 0.094267599 | -0.813702776 | 0.415815244 | 0.988988648 |
| Q92890 | Ubiquitin recognition factor in ER-associated degradation protein 1 | UFD1 | Homo sapiens | 1.900017237 | 0.253734358 | 0.313280754 | 0.809926416 | 0.417982469 | 0.988988648 |
| P08195 | 4F2 cell-surface antigen heavy chain | SLC3A2 | Homo sapiens | 9.24002365 | -0.125705622 | 0.155267024 | -0.809609266 | 0.418164781 | 0.988988648 |
| P51665 | 26S proteasome non-ATPase regulatory subunit 7 | PSMD7 | Homo sapiens | 2.759512934 | -0.207567065 | 0.256921482 | -0.807900777 | 0.419147704 | 0.988988648 |
| Q01581 | Hydroxymethylglutaryl-CoA synthase, cytoplasmic | HMGCS1 | Homo sapiens | 1.762693115 | 0.273306353 | 0.339166415 | 0.805817856 | 0.420347881 | 0.988988648 |
| Q8TCT9 | Minor histocompatibility antigen H13 | HM13 | Homo sapiens | 2.577294116 | -0.233771148 | 0.290586869 | -0.804479395 | 0.421120165 | 0.988988648 |
| Q86V81 | THO complex subunit 4 | ALYREF | Homo sapiens | 5.313249057 | 0.149557294 | 0.186065578 | 0.80378808 | 0.421519375 | 0.988988648 |
| P06756 | Integrin alpha-V | ITGAV | Homo sapiens | 3.822514021 | -0.19515847 | 0.243140833 | -0.802656086 | 0.422173541 | 0.988988648 |
| Q16543 | Hsp90 co-chaperone Cdc37 | CDC37 | Homo sapiens | 4.880891404 | -0.156402348 | 0.195188402 | -0.801289145 | 0.422964273 | 0.988988648 |
| Q9BU23 | Lipase maturation factor 2 | LMF2 | Homo sapiens | 2.085160773 | -0.254061499 | 0.317095248 | -0.801215092 | 0.423007136 | 0.988988648 |
| Q58FF6 | Putative heat shock protein HSP 90-beta 4 | HSP90AB4P | Homo sapiens | 11.01437811 | -0.10404286 | 0.129860651 | -0.801188499 | 0.423022529 | 0.988988648 |
| P61221 | ATP-binding cassette sub-family E member 1 | ABCE1 | Homo sapiens | 2.546978537 | -0.217294458 | 0.271430406 | -0.800553117 | 0.423390401 | 0.988988648 |
| P61513 | 60S ribosomal protein L37a | RPL37A | Homo sapiens | 5.783321744 | -0.143027937 | 0.179123704 | -0.798486932 | 0.424587974 | 0.988988648 |
| P04080 | Cystatin-B | CSTB | Homo sapiens | 1.173452302 | -0.371626805 | 0.467603247 | -0.794748127 | 0.426760031 | NA |
| P61077 | Ubiquitin-conjugating enzyme E2 D3 | UBE2D3 | Homo sapiens | 6.71065635 | -0.134732331 | 0.170029936 | -0.792403588 | 0.428125388 | 0.988988648 |
| A6NIZ1 | Ras-related protein Rap-1b-like protein | Ras-related protein Rap-1b-like protein | Homo sapiens | 1.220299178 | -0.311266208 | 0.392860319 | -0.792307578 | 0.428181354 | NA |
| P11388 | DNA topoisomerase 2-alpha | TOP2A | Homo sapiens | 5.958423882 | 0.222288301 | 0.280911435 | 0.791310971 | 0.428762548 | 0.988988648 |
| P02774 | Vitamin D-binding protein | GC | Homo sapiens | 2.935055972 | 0.201239374 | 0.254625999 | 0.790333174 | 0.429333218 | 0.988988648 |
| P24752 | Acetyl-CoA acetyltransferase, mitochondrial | ACAT1 | Homo sapiens | 4.064339323 | -0.176784888 | 0.223927855 | -0.789472519 | 0.429835885 | 0.988988648 |
| P05023 | Sodium/potassium-transporting ATPase subunit alpha-1 | ATP1A1 | Homo sapiens | 23.61331514 | -0.070747238 | 0.089729055 | -0.788454055 | 0.430431163 | 0.988988648 |
| Q99666 | RANBP2-like and GRIP domain-containing protein 5/6 | RGPD5 | Homo sapiens | 1.000641561 | 0.345112709 | 0.438320222 | 0.787352924 | 0.431075296 | NA |
| Q9UHD1 | Cysteine and histidine-rich domain-containing protein 1 | CHORDC1 | Homo sapiens | 1.763053097 | 0.261510626 | 0.332738715 | 0.785933869 | 0.431906231 | 0.988988648 |
| P35580 | Myosin-10 | MYH10 | Homo sapiens | 75.75362728 | 0.039338123 | 0.050236631 | 0.783056549 | 0.433593905 | 0.988988648 |
| P11908 | Ribose-phosphate pyrophosphokinase 2 | PRPS2 | Homo sapiens | 2.730188856 | 0.197302929 | 0.252033397 | 0.782844381 | 0.433718502 | 0.988988648 |
| O75821 | Eukaryotic translation initiation factor 3 subunit G | EIF3G | Homo sapiens | 5.392112177 | 0.146103679 | 0.187074035 | 0.780993894 | 0.434806086 | 0.988988648 |
| Q9UBL6 | Copine-7 | CPNE7 | Homo sapiens | 1.543418635 | 0.271938273 | 0.348579309 | 0.780133147 | 0.435312507 | NA |
| P69849 | Nodal modulator 3 | NOMO3 | Homo sapiens | 2.47055382 | -0.249570668 | 0.32052279 | -0.778636266 | 0.43619401 | 0.988988648 |
| Q12906 | Interleukin enhancer-binding factor 3 | ILF3 | Homo sapiens | 16.66287549 | 0.087001732 | 0.112058752 | 0.776393903 | 0.437516443 | 0.988988648 |
| P42704 | Leucine-rich PPR motif-containing protein, mitochondrial | LRPPRC | Homo sapiens | 7.046162595 | 0.128599624 | 0.165839812 | 0.775444826 | 0.438076856 | 0.988988648 |
| O15371 | Eukaryotic translation initiation factor 3 subunit D | EIF3D | Homo sapiens | 7.144550659 | -0.12650535 | 0.163723652 | -0.772676081 | 0.439714106 | 0.988988648 |
| P62861 | 40S ribosomal protein S30 | FAU | Homo sapiens | 4.261094572 | 0.159675068 | 0.207247409 | 0.77045628 | 0.441029279 | 0.988988648 |
| O75578 | Integrin alpha-10 | ITGA10 | Homo sapiens | 3.186973095 | 0.193771825 | 0.252252122 | 0.768167271 | 0.442387815 | 0.988988648 |
| P17612 | cAMP-dependent protein kinase catalytic subunit alpha | PRKACA | Homo sapiens | 2.376989085 | -0.300753724 | 0.392400313 | -0.766446186 | 0.44341086 | 0.988988648 |
| P46109 | Crk-like protein | CRKL | Homo sapiens | 1.322040897 | 0.301844324 | 0.394955242 | 0.764249444 | 0.444718607 | NA |
| P20073 | Annexin A7 | ANXA7 | Homo sapiens | 0.776071826 | -0.409039397 | 0.536356322 | -0.762626225 | 0.44568634 | NA |
| O95232 | Luc7-like protein 3 | LUC7L3 | Homo sapiens | 1.604288902 | -0.287528054 | 0.377141187 | -0.762388368 | 0.445828247 | NA |
| P26639 | Threonine--tRNA ligase 1, cytoplasmic | TARS1 | Homo sapiens | 11.95711474 | -0.098862196 | 0.129823001 | -0.761515257 | 0.446349369 | 0.988988648 |
| P28072 | Proteasome subunit beta type-6 | PSMB6 | Homo sapiens | 4.041857959 | -0.165456896 | 0.217453132 | -0.760885317 | 0.446725569 | 0.988988648 |
| Q99497 | Parkinson disease protein 7 | PARK7 | Homo sapiens | 9.743420087 | 0.110125127 | 0.144801598 | 0.760524249 | 0.44694128 | 0.988988648 |
| Q9UQ35 | Serine/arginine repetitive matrix protein 2 | SRRM2 | Homo sapiens | 5.764948388 | 0.141400126 | 0.186066538 | 0.759943877 | 0.447288133 | 0.988988648 |
| P53814 | Smoothelin | SMTN | Homo sapiens | 3.607229941 | -0.183309626 | 0.241407429 | -0.759337136 | 0.447650908 | 0.988988648 |
| O94979 | Protein transport protein Sec31A | SEC31A | Homo sapiens | 18.27606284 | -0.081973451 | 0.108151663 | -0.757949056 | 0.448481481 | 0.988988648 |
| P09211 | Glutathione S-transferase P | GSTP1 | Homo sapiens | 5.86906845 | -0.179801661 | 0.237240982 | -0.757886178 | 0.448519125 | 0.988988648 |
| O00299 | Chloride intracellular channel protein 1 | CLIC1 | Homo sapiens | 30.38744263 | 0.060646738 | 0.080026682 | 0.75783147 | 0.44855188 | 0.988988648 |
| Q15019 | Septin-2 | SEPTIN2 | Homo sapiens | 15.99825023 | -0.081302887 | 0.10736498 | -0.757257042 | 0.448895882 | 0.988988648 |
| O00567 | Nucleolar protein 56 | NOP56 | Homo sapiens | 7.012248654 | -0.122142842 | 0.161426889 | -0.756644961 | 0.449262597 | 0.988988648 |
| Q00577 | Transcriptional activator protein Pur-alpha | PURA | Homo sapiens | 1.884970623 | -0.258597887 | 0.341987311 | -0.75616223 | 0.449551935 | 0.988988648 |
| Q02809 | Procollagen-lysine,2-oxoglutarate 5-dioxygenase 1 | PLOD1 | Homo sapiens | 4.337464401 | -0.159817281 | 0.212062986 | -0.753631191 | 0.45107071 | 0.988988648 |
| O15427 | Monocarboxylate transporter 4 | SLC16A3 | Homo sapiens | 4.769598813 | -0.152841397 | 0.203201265 | -0.752167549 | 0.451950306 | 0.988988648 |
| P07305 | Histone H1.0 | H1-0 | Homo sapiens | 2.970086616 | 0.240712399 | 0.320081477 | 0.752034768 | 0.452030151 | 0.988988648 |
| O43143 | Pre-mRNA-splicing factor ATP-dependent RNA helicase DHX15 | DHX15 | Homo sapiens | 13.36151957 | 0.088547614 | 0.117762461 | 0.751917144 | 0.452100887 | 0.988988648 |
| P51991 | Heterogeneous nuclear ribonucleoprotein A3 | HNRNPA3 | Homo sapiens | 27.61502845 | -0.061362732 | 0.081827601 | -0.74990261 | 0.453313362 | 0.988988648 |
| O14787 | Transportin-2 | TNPO2 | Homo sapiens | 0.748423992 | 0.360249237 | 0.48232979 | 0.746894023 | 0.455127534 | NA |
| O43252 | Bifunctional 3'-phosphoadenosine 5'-phosphosulfate synthase 1 | PAPSS1 | Homo sapiens | 0.530188088 | -0.474688549 | 0.637359802 | -0.744773278 | 0.456408791 | NA |
| Q6P2Q9 | Pre-mRNA-processing-splicing factor 8 | PRPF8 | Homo sapiens | 12.59955621 | 0.095280742 | 0.128179505 | 0.743338349 | 0.45727686 | 0.988988648 |
| P04350 | Tubulin beta-4A chain | TUBB4A | Homo sapiens | 50.31421017 | -0.059848479 | 0.080805984 | -0.740644143 | 0.458909235 | 0.988988648 |
| P24941 | Cyclin-dependent kinase 2 | CDK2 | Homo sapiens | 1.342061969 | 0.276413115 | 0.373789649 | 0.739488413 | 0.459610473 | NA |
| Q9Y6M1 | Insulin-like growth factor 2 mRNA-binding protein 2 | IGF2BP2 | Homo sapiens | 4.772968754 | 0.154192246 | 0.208679251 | 0.738895916 | 0.459970203 | 0.988988648 |
| P84077 | ADP-ribosylation factor 1 | ARF1 | Homo sapiens | 23.04330001 | 0.068939572 | 0.093659341 | 0.736067235 | 0.461689782 | 0.988988648 |
| O00425 | Insulin-like growth factor 2 mRNA-binding protein 3 | IGF2BP3 | Homo sapiens | 2.921412795 | 0.198885601 | 0.270472179 | 0.735327388 | 0.462140132 | 0.988988648 |
| P13798 | Acylamino-acid-releasing enzyme | APEH | Homo sapiens | 1.178406465 | 0.301410356 | 0.411411539 | 0.732624943 | 0.463787214 | NA |
| Q9UBB4 | Ataxin-10 | ATXN10 | Homo sapiens | 0.885473478 | 0.354745555 | 0.484909624 | 0.731570456 | 0.464430786 | NA |
| Q13409 | Cytoplasmic dynein 1 intermediate chain 2 | DYNC1I2 | Homo sapiens | 12.32322211 | -0.090411994 | 0.123612888 | -0.731412363 | 0.464527316 | 0.988988648 |
| Q9HCJ1 | Progressive ankylosis protein homolog | ANKH | Homo sapiens | 3.066708442 | 0.179776127 | 0.2459372 | 0.730983872 | 0.464789004 | 0.988988648 |
| P46934 | E3 ubiquitin-protein ligase NEDD4 | NEDD4 | Homo sapiens | 5.50712035 | -0.150241337 | 0.205572027 | -0.73084524 | 0.464873687 | 0.988988648 |
| Q15582 | Transforming growth factor-beta-induced protein ig-h3 | TGFBI | Homo sapiens | 3.351617521 | -0.244200752 | 0.334422223 | -0.730216878 | 0.465257627 | 0.988988648 |
| Q02218 | 2-oxoglutarate dehydrogenase, mitochondrial | OGDH | Homo sapiens | 6.228750077 | -0.124473883 | 0.170518271 | -0.729973876 | 0.465406153 | 0.988988648 |
| P04259 | Keratin, type II cytoskeletal 6B | KRT6B | Homo sapiens | 2.316705954 | 0.221739218 | 0.303965333 | 0.72948851 | 0.465702893 | 0.988988648 |
| P21802 | Fibroblast growth factor receptor 2 | FGFR2 | Homo sapiens | 0.834974165 | 0.33046475 | 0.453082432 | 0.729370037 | 0.465775341 | NA |
| Q13620 | Cullin-4B | CUL4B | Homo sapiens | 1.708022037 | -0.252896551 | 0.347013726 | -0.728779676 | 0.466136446 | NA |
| Q32P28 | Prolyl 3-hydroxylase 1 | P3H1 | Homo sapiens | 3.853189702 | -0.17498242 | 0.240816504 | -0.72662138 | 0.467457927 | 0.988988648 |
| P13804 | Electron transfer flavoprotein subunit alpha, mitochondrial | ETFA | Homo sapiens | 5.269898676 | -0.136359457 | 0.188202167 | -0.724537122 | 0.468736046 | 0.988988648 |
| Q12797 | Aspartyl/asparaginyl beta-hydroxylase | ASPH | Homo sapiens | 14.08304926 | -0.089512911 | 0.123547427 | -0.72452266 | 0.468744921 | 0.988988648 |
| Q96S21 | Ras-related protein Rab-40C | RAB40C | Homo sapiens | 2.449091916 | -0.200670401 | 0.277172774 | -0.723990305 | 0.469071687 | 0.988988648 |
| O60888 | Protein CutA | CUTA | Homo sapiens | 7.295767225 | -0.118526839 | 0.163766472 | -0.72375522 | 0.469216026 | 0.988988648 |
| Q14258 | E3 ubiquitin/ISG15 ligase TRIM25 | TRIM25 | Homo sapiens | 4.441477414 | 0.157979285 | 0.218515959 | 0.722964517 | 0.469701684 | 0.988988648 |
| P06396 | Gelsolin | GSN | Homo sapiens | 27.35527024 | -0.05900582 | 0.081908651 | -0.720385685 | 0.471287561 | 0.988988648 |
| Q96KG9 | N-terminal kinase-like protein | SCYL1 | Homo sapiens | 0.511602829 | 0.488846447 | 0.680250347 | 0.718627266 | 0.472370609 | NA |
| O15523 | ATP-dependent RNA helicase DDX3Y | DDX3Y | Homo sapiens | 3.537450783 | -0.17755382 | 0.247298904 | -0.717972531 | 0.472774224 | 0.988988648 |
| P30043 | Flavin reductase (NADPH) | BLVRB | Homo sapiens | 3.452015629 | -0.180493649 | 0.251797419 | -0.716820884 | 0.473484624 | 0.988988648 |
| Q9UHB6 | LIM domain and actin-binding protein 1 | LIMA1 | Homo sapiens | 7.700118902 | -0.115935804 | 0.162514647 | -0.713386799 | 0.47560644 | 0.988988648 |
| P0DP24 | Calmodulin-2 | CALM2 | Homo sapiens | 1.718157778 | 0.239834978 | 0.337319945 | 0.711001473 | 0.477083323 | 0.988988648 |
| Q9P266 | Junctional protein associated with coronary artery disease | JCAD | Homo sapiens | 1.23921544 | -0.339471118 | 0.478454605 | -0.709515835 | 0.478004429 | NA |
| O75828 | Carbonyl reductase [NADPH] 3 | CBR3 | Homo sapiens | 4.943237316 | 0.144500463 | 0.204508164 | 0.706575524 | 0.479830304 | 0.988988648 |
| Q15691 | Microtubule-associated protein RP/EB family member 1 | MAPRE1 | Homo sapiens | 4.557702067 | -0.140909836 | 0.199570353 | -0.706065976 | 0.480147109 | 0.988988648 |
| Q9BXJ9 | N-alpha-acetyltransferase 15, NatA auxiliary subunit | NAA15 | Homo sapiens | 0.703409626 | 0.392381048 | 0.55577546 | 0.70600643 | 0.480184139 | NA |
| P62491 | Ras-related protein Rab-11A | RAB11A | Homo sapiens | 0.621094149 | -0.368998809 | 0.522862282 | -0.705728491 | 0.480357 | NA |
| P63000 | Ras-related C3 botulinum toxin substrate 1 | RAC1 | Homo sapiens | 4.644999644 | -0.141095027 | 0.199966588 | -0.705593015 | 0.480441269 | 0.988988648 |
| Q00266 | S-adenosylmethionine synthase isoform type-1 | MAT1A | Homo sapiens | 0.790023951 | 0.325593289 | 0.461791683 | 0.705065294 | 0.480769604 | NA |
| P50991 | T-complex protein 1 subunit delta | CCT4 | Homo sapiens | 30.19372936 | 0.055665175 | 0.078952026 | 0.705050627 | 0.480778731 | 0.988988648 |
| P60903 | Protein S100-A10 | S100A10 | Homo sapiens | 5.307746839 | -0.131041073 | 0.186143184 | -0.703979968 | 0.481445249 | 0.988988648 |
| O00116 | Alkyldihydroxyacetonephosphate synthase, peroxisomal | AGPS | Homo sapiens | 1.136216539 | -0.30519593 | 0.43446279 | -0.702467364 | 0.482387748 | NA |
| P19338 | Nucleolin | NCL | Homo sapiens | 46.40125027 | 0.045497763 | 0.064840847 | 0.701683659 | 0.482876466 | 0.988988648 |
| Q9Y5L0 | Transportin-3 | TNPO3 | Homo sapiens | 0.789475223 | 0.344925843 | 0.492642959 | 0.700153806 | 0.483831257 | NA |
| Q15287 | RNA-binding protein with serine-rich domain 1 | RNPS1 | Homo sapiens | 1.787835402 | 0.217815074 | 0.311457245 | 0.699341814 | 0.484338442 | 0.988988648 |
| P28331 | NADH-ubiquinone oxidoreductase 75 kDa subunit, mitochondrial | NDUFS1 | Homo sapiens | 0.560763217 | -0.478835345 | 0.685002274 | -0.699027381 | 0.484534919 | NA |
| P63244 | Receptor of activated protein C kinase 1 | RACK1 | Homo sapiens | 61.10326676 | 0.039578587 | 0.056646139 | 0.698698759 | 0.484740309 | 0.988988648 |
| P13693 | Translationally-controlled tumor protein | TPT1 | Homo sapiens | 9.168729503 | 0.101103849 | 0.144806859 | 0.698197927 | 0.485053423 | 0.988988648 |
| Q15382 | GTP-binding protein Rheb | RHEB | Homo sapiens | 1.578331844 | -0.24181146 | 0.346644359 | -0.697577946 | 0.485441178 | NA |
| Q5T4S7 | E3 ubiquitin-protein ligase UBR4 | UBR4 | Homo sapiens | 5.138895453 | -0.136202802 | 0.196330016 | -0.69374416 | 0.487842667 | 0.988988648 |
| Q14194 | Dihydropyrimidinase-related protein 1 | CRMP1 | Homo sapiens | 8.488486847 | -0.101126039 | 0.146328626 | -0.691088554 | 0.489509893 | 0.988988648 |
| Q9NZN4 | EH domain-containing protein 2 | EHD2 | Homo sapiens | 17.33082783 | -0.075729857 | 0.109668167 | -0.690536362 | 0.489856951 | 0.988988648 |
| Q9Y5B9 | FACT complex subunit SPT16 | SUPT16H | Homo sapiens | 3.160515425 | 0.170120688 | 0.246441679 | 0.690308102 | 0.490000454 | 0.988988648 |
| Q9Y316 | Protein MEMO1 | MEMO1 | Homo sapiens | 3.144597284 | -0.168979925 | 0.245218872 | -0.689098372 | 0.490761365 | 0.988988648 |
| P12111 | Collagen alpha-3(VI) chain | COL6A3 | Homo sapiens | 51.78248591 | -0.055459776 | 0.0807588 | -0.68673354 | 0.492250657 | 0.988988648 |
| P07996 | Thrombospondin-1 | THBS1 | Homo sapiens | 29.20826188 | -0.124037709 | 0.180869319 | -0.68578634 | 0.492847851 | 0.988988648 |
| Q8WUM4 | Programmed cell death 6-interacting protein | PDCD6IP | Homo sapiens | 22.63924041 | 0.062165074 | 0.090702118 | 0.685376211 | 0.493106552 | 0.988988648 |
| O43432 | Eukaryotic translation initiation factor 4 gamma 3 | EIF4G3 | Homo sapiens | 2.622398758 | -0.182929088 | 0.267129676 | -0.684795082 | 0.493473239 | 0.988988648 |
| Q04637 | Eukaryotic translation initiation factor 4 gamma 1 | EIF4G1 | Homo sapiens | 18.07283253 | -0.069640542 | 0.101779084 | -0.684232352 | 0.493828456 | 0.988988648 |
| P61026 | Ras-related protein Rab-10 | RAB10 | Homo sapiens | 11.40997305 | -0.086082387 | 0.125812933 | -0.68420936 | 0.493842972 | 0.988988648 |
| P08631 | Tyrosine-protein kinase HCK | HCK | Homo sapiens | 1.489482726 | 0.243260172 | 0.35564893 | 0.683989606 | 0.493981729 | NA |
| O14818 | Proteasome subunit alpha type-7 | PSMA7 | Homo sapiens | 3.213851009 | 0.224374603 | 0.328313818 | 0.683415047 | 0.494344615 | 0.988988648 |
| A1L0T0 | 2-hydroxyacyl-CoA lyase 2 | ILVBL | Homo sapiens | 0.737776546 | 0.416089441 | 0.609881391 | 0.682246495 | 0.495083099 | NA |
| Q07960 | Rho GTPase-activating protein 1 | ARHGAP1 | Homo sapiens | 6.275290663 | 0.121057653 | 0.177771977 | 0.680971519 | 0.495889512 | 0.988988648 |
| Q15185 | Prostaglandin E synthase 3 | PTGES3 | Homo sapiens | 5.949189545 | 0.118062667 | 0.173884062 | 0.678973484 | 0.497154663 | 0.988988648 |
| P28070 | Proteasome subunit beta type-4 | PSMB4 | Homo sapiens | 1.917668486 | -0.216563346 | 0.319031465 | -0.678815008 | 0.497255083 | 0.988988648 |
| Q9UKM9 | RNA-binding protein Raly | RALY | Homo sapiens | 0.984441211 | 0.313999871 | 0.463264323 | 0.677798517 | 0.497899451 | NA |
| P55884 | Eukaryotic translation initiation factor 3 subunit B | EIF3B | Homo sapiens | 13.19339869 | 0.07966643 | 0.117681524 | 0.676966335 | 0.498427314 | 0.988988648 |
| Q01082 | Spectrin beta chain, non-erythrocytic 1 | SPTBN1 | Homo sapiens | 27.32609136 | -0.061113392 | 0.090462669 | -0.675564761 | 0.499317021 | 0.988988648 |
| Q92526 | T-complex protein 1 subunit zeta-2 | CCT6B | Homo sapiens | 4.288359578 | 0.137626664 | 0.204371346 | 0.673414678 | 0.500683513 | 0.988988648 |
| Q9UHB9 | Signal recognition particle subunit SRP68 | SRP68 | Homo sapiens | 0.892904352 | 0.316712841 | 0.470874473 | 0.67260567 | 0.501198194 | NA |
| P18124 | 60S ribosomal protein L7 | RPL7 | Homo sapiens | 15.74018386 | -0.072483546 | 0.107967494 | -0.67134601 | 0.502000131 | 0.988988648 |
| Q8N4C8 | Misshapen-like kinase 1 | MINK1 | Homo sapiens | 2.828061216 | 0.177168779 | 0.264189706 | 0.67061197 | 0.502467755 | 0.988988648 |
| O00303 | Eukaryotic translation initiation factor 3 subunit F | EIF3F | Homo sapiens | 11.24183333 | -0.08493254 | 0.126914028 | -0.66921318 | 0.5033595 | 0.988988648 |
| Q9H0B6 | Kinesin light chain 2 | KLC2 | Homo sapiens | 1.561494178 | 0.239985609 | 0.359578261 | 0.667408557 | 0.504511202 | NA |
| P55265 | Double-stranded RNA-specific adenosine deaminase | ADAR | Homo sapiens | 0.652603165 | 0.351787306 | 0.527536041 | 0.666849806 | 0.504868075 | NA |
| Q13619 | Cullin-4A | CUL4A | Homo sapiens | 1.927438581 | 0.216347331 | 0.32524047 | 0.665191917 | 0.50592775 | 0.988988648 |
| P12109 | Collagen alpha-1(VI) chain | COL6A1 | Homo sapiens | 29.43898774 | -0.056184908 | 0.084494139 | -0.664956277 | 0.506078459 | 0.988988648 |
| P31150 | Rab GDP dissociation inhibitor alpha | GDI1 | Homo sapiens | 30.80247983 | -0.052382039 | 0.07892652 | -0.663681092 | 0.506894444 | 0.988988648 |
| P62937 | Peptidyl-prolyl cis-trans isomerase A | PPIA | Homo sapiens | 66.66020827 | -0.035990755 | 0.054321221 | -0.662554235 | 0.507616089 | 0.988988648 |
| P62829 | 60S ribosomal protein L23 | RPL23 | Homo sapiens | 14.7394964 | -0.073396687 | 0.110784788 | -0.662515934 | 0.507640627 | 0.988988648 |
| P40261 | Nicotinamide N-methyltransferase | NNMT | Homo sapiens | 6.454601724 | 0.117417266 | 0.177389068 | 0.661919405 | 0.508022875 | 0.988988648 |
| P51149 | Ras-related protein Rab-7a | RAB7A | Homo sapiens | 11.14154883 | -0.087511098 | 0.132407108 | -0.660924475 | 0.508660749 | 0.988988648 |
| O00560 | Syntenin-1 | SDCBP | Homo sapiens | 4.253800695 | 0.144504635 | 0.218772998 | 0.660523174 | 0.508918152 | 0.988988648 |
| P17931 | Galectin-3 | LGALS3 | Homo sapiens | 3.431645089 | 0.181631657 | 0.275621708 | 0.658988941 | 0.50990287 | 0.988988648 |
| Q13501 | Sequestosome-1 | SQSTM1 | Homo sapiens | 20.0769414 | -0.06815119 | 0.104049367 | -0.65498899 | 0.512474843 | 0.988988648 |
| O43795 | Unconventional myosin-Ib | MYO1B | Homo sapiens | 0.920328685 | 0.354277245 | 0.541439656 | 0.654324525 | 0.512902749 | NA |
| P62826 | GTP-binding nuclear protein Ran | RAN | Homo sapiens | 26.34250692 | -0.054145654 | 0.083019374 | -0.652205039 | 0.51426891 | 0.988988648 |
| Q9UQ16 | Dynamin-3 | DNM3 | Homo sapiens | 0.971573027 | 0.280972364 | 0.430888486 | 0.652076751 | 0.514351661 | NA |
| P58107 | Epiplakin | EPPK1 | Homo sapiens | 3.306158888 | 0.15902643 | 0.244248659 | 0.651084148 | 0.514992169 | 0.988988648 |
| Q7Z2W4 | Zinc finger CCCH-type antiviral protein 1 | ZC3HAV1 | Homo sapiens | 1.582707099 | 0.233682632 | 0.359115192 | 0.650717756 | 0.515228699 | NA |
| P25205 | DNA replication licensing factor MCM3 | MCM3 | Homo sapiens | 5.546576949 | 0.120761756 | 0.185716623 | 0.650247426 | 0.515532411 | 0.988988648 |
| Q15813 | Tubulin-specific chaperone E | TBCE | Homo sapiens | 0.686847428 | -0.361488975 | 0.55656156 | -0.649504028 | 0.516012644 | NA |
| Q9Y6V0 | Protein piccolo | PCLO | Homo sapiens | 3.050322136 | -0.174312139 | 0.268575638 | -0.649024387 | 0.516322614 | 0.988988648 |
| P29992 | Guanine nucleotide-binding protein subunit alpha-11 | GNA11 | Homo sapiens | 2.609934473 | 0.176577759 | 0.272592654 | 0.647771524 | 0.517132736 | 0.988988648 |
| Q92974 | Rho guanine nucleotide exchange factor 2 | ARHGEF2 | Homo sapiens | 1.086875786 | 0.282289614 | 0.435811794 | 0.647732847 | 0.517157755 | NA |
| Q14152 | Eukaryotic translation initiation factor 3 subunit A | EIF3A | Homo sapiens | 17.99648586 | 0.065133906 | 0.100578141 | 0.647595048 | 0.517246901 | 0.988988648 |
| O76003 | Glutaredoxin-3 | GLRX3 | Homo sapiens | 5.010423187 | -0.142768825 | 0.221283215 | -0.645185965 | 0.518806681 | 0.988988648 |
| Q96CG8 | Collagen triple helix repeat-containing protein 1 | CTHRC1 | Homo sapiens | 1.898909749 | -0.209272115 | 0.325179091 | -0.643559567 | 0.519861076 | 0.988988648 |
| Q5T9A4 | ATPase family AAA domain-containing protein 3B | ATAD3B | Homo sapiens | 0.924695357 | 0.340518037 | 0.529592565 | 0.64298115 | 0.520236331 | NA |
| P00441 | Superoxide dismutase [Cu-Zn] | SOD1 | Homo sapiens | 17.49366366 | -0.066840679 | 0.103966044 | -0.642908745 | 0.520283315 | 0.988988648 |
| P62888 | 60S ribosomal protein L30 | RPL30 | Homo sapiens | 11.76674099 | 0.079071106 | 0.123544912 | 0.640019123 | 0.522160167 | 0.988988648 |
| O95394 | Phosphoacetylglucosamine mutase | PGM3 | Homo sapiens | 2.893647913 | 0.170682658 | 0.267224634 | 0.638723517 | 0.523002811 | 0.988988648 |
| Q3ZCM7 | Tubulin beta-8 chain | TUBB8 | Homo sapiens | 80.76902286 | 0.031950074 | 0.050036261 | 0.638538397 | 0.523123267 | 0.988988648 |
| P38606 | V-type proton ATPase catalytic subunit A | ATP6V1A | Homo sapiens | 10.27704654 | 0.085709124 | 0.134239587 | 0.638478748 | 0.523162084 | 0.988988648 |
| Q96IU4 | Protein ABHD14B | ABHD14B | Homo sapiens | 0.726420559 | -0.325155512 | 0.510236586 | -0.637264205 | 0.523952763 | NA |
| Q7Z4F1 | Low-density lipoprotein receptor-related protein 10 | LRP10 | Homo sapiens | 0.82891455 | -0.393034738 | 0.616937244 | -0.637074098 | 0.524076579 | NA |
| Q9HCH3 | Copine-5 | CPNE5 | Homo sapiens | 1.42202488 | 0.211950542 | 0.334304092 | 0.634005228 | 0.52607741 | NA |
| P21291 | Cysteine and glycine-rich protein 1 | CSRP1 | Homo sapiens | 14.39614992 | 0.071619166 | 0.112989882 | 0.63385468 | 0.526175664 | 0.988988648 |
| Q9UHD9 | Ubiquilin-2 | UBQLN2 | Homo sapiens | 9.776389724 | -0.090492602 | 0.143222456 | -0.6318325 | 0.527496334 | 0.988988648 |
| Q9Y265 | RuvB-like 1 | RUVBL1 | Homo sapiens | 5.006840167 | 0.121440851 | 0.192245547 | 0.63169656 | 0.527585176 | 0.988988648 |
| P61086 | Ubiquitin-conjugating enzyme E2 K | UBE2K | Homo sapiens | 6.013185268 | -0.110326432 | 0.174819428 | -0.631087936 | 0.527983029 | 0.988988648 |
| P05198 | Eukaryotic translation initiation factor 2 subunit 1 | EIF2S1 | Homo sapiens | 3.220057276 | 0.166860488 | 0.264879444 | 0.629948801 | 0.528728083 | 0.988988648 |
| O00151 | PDZ and LIM domain protein 1 | PDLIM1 | Homo sapiens | 8.284043627 | -0.095520927 | 0.151720896 | -0.6295832 | 0.528967318 | 0.988988648 |
| Q12931 | Heat shock protein 75 kDa, mitochondrial | TRAP1 | Homo sapiens | 10.16852734 | 0.083560646 | 0.132869714 | 0.628891591 | 0.529420032 | 0.988988648 |
| Q14980 | Nuclear mitotic apparatus protein 1 | NUMA1 | Homo sapiens | 1.997802324 | 0.212768419 | 0.338362441 | 0.628818076 | 0.529468165 | 0.988988648 |
| Q9BVA1 | Tubulin beta-2B chain | TUBB2B | Homo sapiens | 56.94662808 | 0.044403177 | 0.070694759 | 0.628097148 | 0.529940302 | 0.988988648 |
| Q15021 | Condensin complex subunit 1 | NCAPD2 | Homo sapiens | 1.15815584 | 0.265449855 | 0.422828838 | 0.627795057 | 0.530138205 | NA |
| Q5T1J5 | Putative coiled-coil-helix-coiled-coil-helix domain-containing protein CHCHD2P9, mitochondrial | CHCHD2P9 | Homo sapiens | 0.620829019 | 0.379865117 | 0.606085193 | 0.626752017 | 0.530821801 | NA |
| Q96QS3 | Homeobox protein ARX | ARX | Homo sapiens | 2.149716357 | 0.227711087 | 0.363587174 | 0.626290208 | 0.531124608 | 0.988988648 |
| P20794 | Serine/threonine-protein kinase MAK | MAK | Homo sapiens | 0.817933301 | 0.314035268 | 0.502718176 | 0.624674585 | 0.532184657 | NA |
| Q9H4M9 | EH domain-containing protein 1 | EHD1 | Homo sapiens | 6.667469822 | 0.104627031 | 0.168264407 | 0.621801322 | 0.534072517 | 0.988988648 |
| P62879 | Guanine nucleotide-binding protein G(I)/G(S)/G(T) subunit beta-2 | GNB2 | Homo sapiens | 16.19078495 | -0.066533335 | 0.107160812 | -0.62087375 | 0.534682694 | 0.988988648 |
| Q7Z3Y8 | Keratin, type I cytoskeletal 27 | KRT27 | Homo sapiens | 1.32735795 | -0.266112818 | 0.429702283 | -0.619295798 | 0.535721512 | NA |
| P62191 | 26S proteasome regulatory subunit 4 | PSMC1 | Homo sapiens | 6.558115599 | 0.106359156 | 0.171999813 | 0.618367856 | 0.536332881 | 0.988988648 |
| Q99729 | Heterogeneous nuclear ribonucleoprotein A/B | HNRNPAB | Homo sapiens | 26.99002345 | -0.051225998 | 0.083038063 | -0.616897795 | 0.537302141 | 0.988988648 |
| O15050 | TPR and ankyrin repeat-containing protein 1 | TRANK1 | Homo sapiens | 0.888853171 | 0.288617621 | 0.468158249 | 0.616495856 | 0.537567306 | NA |
| Q99623 | Prohibitin-2 | PHB2 | Homo sapiens | 7.514318482 | 0.095331682 | 0.154669224 | 0.616358443 | 0.537657974 | 0.988988648 |
| Q02543 | 60S ribosomal protein L18a | RPL18A | Homo sapiens | 9.1357271 | 0.086494219 | 0.140651863 | 0.614952533 | 0.538586068 | 0.988988648 |
| P33947 | ER lumen protein-retaining receptor 2 | KDELR2 | Homo sapiens | 2.996178515 | -0.152066906 | 0.247396446 | -0.61466892 | 0.538773389 | 0.988988648 |
| Q969X1 | Protein lifeguard 3 | TMBIM1 | Homo sapiens | 0.639099649 | 0.320980183 | 0.523050925 | 0.613669087 | 0.53943402 | NA |
| P29966 | Myristoylated alanine-rich C-kinase substrate | MARCKS | Homo sapiens | 19.99027245 | -0.064514236 | 0.105227415 | -0.613093417 | 0.539814573 | 0.988988648 |
| P04843 | Dolichyl-diphosphooligosaccharide--protein glycosyltransferase subunit 1 | RPN1 | Homo sapiens | 28.8371061 | -0.05139935 | 0.083845064 | -0.613027739 | 0.539857999 | 0.988988648 |
| Q9BY32 | Inosine triphosphate pyrophosphatase | ITPA | Homo sapiens | 0.960293715 | 0.253914793 | 0.414199476 | 0.613025383 | 0.539859556 | NA |
| P62136 | Serine/threonine-protein phosphatase PP1-alpha catalytic subunit | PPP1CA | Homo sapiens | 8.103794711 | -0.103892921 | 0.16957559 | -0.612664363 | 0.540098291 | 0.988988648 |
| O95486 | Protein transport protein Sec24A | SEC24A | Homo sapiens | 1.861105743 | 0.2179737 | 0.357156976 | 0.610302233 | 0.541661618 | 0.988988648 |
| Q9Y6E0 | Serine/threonine-protein kinase 24 | STK24 | Homo sapiens | 0.904867432 | -0.30955428 | 0.507776232 | -0.609627352 | 0.542108688 | NA |
| P12236 | ADP/ATP translocase 3 | SLC25A6 | Homo sapiens | 20.51480815 | 0.060530704 | 0.099386329 | 0.609044573 | 0.542494895 | 0.988988648 |
| O00629 | Importin subunit alpha-3 | KPNA4 | Homo sapiens | 10.46996675 | 0.081531981 | 0.133870592 | 0.60903578 | 0.542500723 | 0.988988648 |
| P60900 | Proteasome subunit alpha type-6 | PSMA6 | Homo sapiens | 9.293508183 | -0.08549685 | 0.140801579 | -0.607215141 | 0.543708146 | 0.988988648 |
| P31948 | Stress-induced-phosphoprotein 1 | STIP1 | Homo sapiens | 9.814227274 | 0.084883257 | 0.140011834 | 0.606257729 | 0.544343624 | 0.988988648 |
| Q9H3P7 | Golgi resident protein GCP60 | ACBD3 | Homo sapiens | 5.39720712 | 0.118370917 | 0.195414607 | 0.605742421 | 0.54468581 | 0.988988648 |
| P04181 | Ornithine aminotransferase, mitochondrial | OAT | Homo sapiens | 6.721705905 | -0.139541421 | 0.230401131 | -0.605645556 | 0.544750144 | 0.988988648 |
| P20908 | Collagen alpha-1(V) chain | COL5A1 | Homo sapiens | 1.207338406 | 0.357694183 | 0.59123845 | 0.60499141 | 0.545184704 | NA |
| Q9BXS5 | AP-1 complex subunit mu-1 | AP1M1 | Homo sapiens | 0.599718738 | 0.344720915 | 0.57022571 | 0.604534151 | 0.545488571 | NA |
| O95573 | Long-chain-fatty-acid--CoA ligase 3 | ACSL3 | Homo sapiens | 7.412301792 | -0.100817826 | 0.166825476 | -0.604331115 | 0.545623524 | 0.988988648 |
| Q9Y3U8 | 60S ribosomal protein L36 | RPL36 | Homo sapiens | 1.51785233 | 0.352624812 | 0.583634652 | 0.604187586 | 0.545718933 | NA |
| P10768 | S-formylglutathione hydrolase | ESD | Homo sapiens | 12.43065213 | -0.0749073 | 0.124331729 | -0.602479354 | 0.546855102 | 0.988988648 |
| Q9UK76 | Jupiter microtubule associated homolog 1 | JPT1 | Homo sapiens | 4.975164866 | -0.12061587 | 0.200738367 | -0.600861069 | 0.547932525 | 0.988988648 |
| Q15369 | Elongin-C | ELOC | Homo sapiens | 2.23027646 | -0.174772622 | 0.291125921 | -0.600333427 | 0.548284046 | 0.988988648 |
| P55072 | Transitional endoplasmic reticulum ATPase | VCP | Homo sapiens | 84.57461239 | -0.033654537 | 0.056134259 | -0.5995365 | 0.548815177 | 0.988988648 |
| Q99715 | Collagen alpha-1(XII) chain | COL12A1 | Homo sapiens | 1.07438929 | 0.347147755 | 0.582132934 | 0.596337597 | 0.55094972 | NA |
| P61604 | 10 kDa heat shock protein, mitochondrial | HSPE1 | Homo sapiens | 12.02234956 | 0.101312037 | 0.170143106 | 0.595451909 | 0.551541436 | 0.988988648 |
| P30048 | Thioredoxin-dependent peroxide reductase, mitochondrial | PRDX3 | Homo sapiens | 6.411533175 | -0.106828244 | 0.179649966 | -0.594646615 | 0.552079714 | 0.988988648 |
| P35908 | Keratin, type II cytoskeletal 2 epidermal | KRT2 | Homo sapiens | 28.90941125 | -0.097027317 | 0.163346703 | -0.59399618 | 0.552514668 | 0.988988648 |
| Q14103 | Heterogeneous nuclear ribonucleoprotein D0 | HNRNPD | Homo sapiens | 24.11141354 | 0.05164189 | 0.087045737 | 0.593273051 | 0.552998431 | 0.988988648 |
| Q9NR31 | GTP-binding protein SAR1a | SAR1A | Homo sapiens | 5.704340738 | -0.109170411 | 0.185047816 | -0.589957845 | 0.555218912 | 0.988988648 |
| P36507 | Dual specificity mitogen-activated protein kinase kinase 2 | MAP2K2 | Homo sapiens | 1.278193263 | 0.226343864 | 0.383984541 | 0.589460876 | 0.55555215 | NA |
| Q8NC51 | Plasminogen activator inhibitor 1 RNA-binding protein | SERBP1 | Homo sapiens | 8.792040859 | -0.084167402 | 0.143154843 | -0.587946591 | 0.556568142 | 0.988988648 |
| O14974 | Protein phosphatase 1 regulatory subunit 12A | PPP1R12A | Homo sapiens | 3.379605004 | -0.138439847 | 0.235474932 | -0.587917557 | 0.556587631 | 0.988988648 |
| Q96BM9 | ADP-ribosylation factor-like protein 8A | ARL8A | Homo sapiens | 0.52214131 | -0.342423853 | 0.583502748 | -0.586841886 | 0.557309903 | NA |
| P68371 | Tubulin beta-4B chain | TUBB4B | Homo sapiens | 234.3528269 | 0.017964632 | 0.030685875 | 0.58543652 | 0.558254239 | 0.988988648 |
| P21589 | 5'-nucleotidase | NT5E | Homo sapiens | 10.16187008 | -0.087917068 | 0.150256474 | -0.585113342 | 0.558471508 | 0.988988648 |
| P02545 | Prelamin-A/C | LMNA | Homo sapiens | 86.42743374 | 0.030952928 | 0.052907012 | 0.585043966 | 0.558518154 | 0.988988648 |
| Q15233 | Non-POU domain-containing octamer-binding protein | NONO | Homo sapiens | 13.77401084 | 0.067590041 | 0.115697987 | 0.584193754 | 0.559089963 | 0.988988648 |
| O43854 | EGF-like repeat and discoidin I-like domain-containing protein 3 | EDIL3 | Homo sapiens | 0.52751476 | 0.338122576 | 0.579727463 | 0.583244019 | 0.559729042 | NA |
| P61106 | Ras-related protein Rab-14 | RAB14 | Homo sapiens | 5.241072521 | -0.108940495 | 0.186877352 | -0.582951833 | 0.559925726 | 0.988988648 |
| Q8NI27 | THO complex subunit 2 | THOC2 | Homo sapiens | 0.812105378 | -0.256949935 | 0.442780106 | -0.580310478 | 0.561705263 | NA |
| Q9Y2Z0 | Protein SGT1 homolog | SUGT1 | Homo sapiens | 3.919404962 | -0.128740859 | 0.221861847 | -0.580274887 | 0.561729259 | 0.988988648 |
| Q96FW1 | Ubiquitin thioesterase OTUB1 | OTUB1 | Homo sapiens | 2.597447013 | -0.152440264 | 0.26290506 | -0.579830087 | 0.562029206 | 0.988988648 |
| P61254 | 60S ribosomal protein L26 | RPL26 | Homo sapiens | 4.119974897 | 0.121150639 | 0.209290531 | 0.578863449 | 0.562681315 | 0.988988648 |
| Q96A23 | Copine-4 | CPNE4 | Homo sapiens | 1.116265672 | 0.22573068 | 0.390375535 | 0.578239822 | 0.563102217 | NA |
| P46777 | 60S ribosomal protein L5 | RPL5 | Homo sapiens | 22.85171176 | 0.052904693 | 0.091532568 | 0.577987646 | 0.56327246 | 0.988988648 |
| Q99714 | 3-hydroxyacyl-CoA dehydrogenase type-2 | HSD17B10 | Homo sapiens | 5.769306241 | -0.106685811 | 0.184756459 | -0.577440225 | 0.563642108 | 0.988988648 |
| O95373 | Importin-7 | IPO7 | Homo sapiens | 20.17422576 | 0.055158858 | 0.09569258 | 0.576417298 | 0.564333155 | 0.988988648 |
| P27348 | 14-3-3 protein theta | YWHAQ | Homo sapiens | 34.90800848 | -0.041915329 | 0.073103264 | -0.573371518 | 0.566393171 | 0.988988648 |
| O95747 | Serine/threonine-protein kinase OSR1 | OXSR1 | Homo sapiens | 2.736971513 | 0.148120647 | 0.25835296 | 0.57332669 | 0.566423517 | 0.988988648 |
| Q6YHK3 | CD109 antigen | CD109 | Homo sapiens | 2.991281841 | 0.170261159 | 0.297475424 | 0.572353698 | 0.567082378 | 0.988988648 |
| Q99829 | Copine-1 | CPNE1 | Homo sapiens | 8.537340554 | -0.084095843 | 0.147175522 | -0.571398298 | 0.567729683 | 0.988988648 |
| Q99873 | Protein arginine N-methyltransferase 1 | PRMT1 | Homo sapiens | 4.846033171 | 0.111430944 | 0.195087411 | 0.571184696 | 0.567874451 | 0.988988648 |
| P62745 | Rho-related GTP-binding protein RhoB | RHOB | Homo sapiens | 0.810581729 | 0.268980376 | 0.471713619 | 0.570219652 | 0.568528729 | NA |
| P55285 | Cadherin-6 | CDH6 | Homo sapiens | 1.713808217 | 0.235534452 | 0.413737024 | 0.569285411 | 0.569162466 | 0.988988648 |
| P50993 | Sodium/potassium-transporting ATPase subunit alpha-2 | ATP1A2 | Homo sapiens | 8.999366145 | -0.081869318 | 0.144311602 | -0.567309331 | 0.570504038 | 0.988988648 |
| P50213 | Isocitrate dehydrogenase [NAD] subunit alpha, mitochondrial | IDH3A | Homo sapiens | 2.376037511 | 0.165609145 | 0.292398709 | 0.566381247 | 0.57113464 | 0.988988648 |
| Q12904 | Aminoacyl tRNA synthase complex-interacting multifunctional protein 1 | AIMP1 | Homo sapiens | 2.072546294 | -0.191610073 | 0.338404346 | -0.566216349 | 0.571246717 | 0.988988648 |
| Q06787 | Synaptic functional regulator FMR1 | FMR1 | Homo sapiens | 1.983062231 | -0.168583155 | 0.298055997 | -0.565609003 | 0.571659606 | 0.988988648 |
| O43399 | Tumor protein D54 | TPD52L2 | Homo sapiens | 3.081792386 | 0.145648802 | 0.257603931 | 0.565398214 | 0.571802939 | 0.988988648 |
| O75694 | Nuclear pore complex protein Nup155 | NUP155 | Homo sapiens | 1.273982446 | 0.223846641 | 0.396277102 | 0.564874023 | 0.572159454 | NA |
| P62820 | Ras-related protein Rab-1A | RAB1A | Homo sapiens | 14.53792454 | -0.063305211 | 0.112122391 | -0.564608103 | 0.572340353 | 0.988988648 |
| Q9NX58 | Cell growth-regulating nucleolar protein | LYAR | Homo sapiens | 1.838391368 | 0.191937783 | 0.340472518 | 0.563739429 | 0.572931479 | 0.988988648 |
| O60841 | Eukaryotic translation initiation factor 5B | EIF5B | Homo sapiens | 4.625411188 | -0.113068165 | 0.200656344 | -0.563491603 | 0.573100176 | 0.988988648 |
| Q04695 | Keratin, type I cytoskeletal 17 | KRT17 | Homo sapiens | 0.812733435 | 0.27604197 | 0.490120081 | 0.563212936 | 0.573289895 | NA |
| Q9Y6I3 | Epsin-1 | EPN1 | Homo sapiens | 0.79520773 | 0.276066253 | 0.491160524 | 0.562069302 | 0.574068803 | NA |
| P55036 | 26S proteasome non-ATPase regulatory subunit 4 | PSMD4 | Homo sapiens | 4.609571115 | -0.114131328 | 0.203083538 | -0.561992022 | 0.574121455 | 0.988988648 |
| Q05639 | Elongation factor 1-alpha 2 | EEF1A2 | Homo sapiens | 71.12134434 | 0.031999543 | 0.057061589 | 0.560789561 | 0.574941004 | 0.988988648 |
| P02768 | Albumin | ALB | Homo sapiens | 33.5240969 | -0.05937465 | 0.106018696 | -0.560039426 | 0.575452546 | 0.988988648 |
| P08697 | Alpha-2-antiplasmin | SERPINF2 | Homo sapiens | 0.656964241 | -0.313843516 | 0.560498844 | -0.559936063 | 0.575523049 | NA |
| P34932 | Heat shock 70 kDa protein 4 | HSPA4 | Homo sapiens | 41.519479 | 0.037378882 | 0.066874161 | 0.558943567 | 0.576200235 | 0.988988648 |
| Q05682 | Caldesmon | CALD1 | Homo sapiens | 31.01525242 | -0.045898284 | 0.082174411 | -0.558547163 | 0.576470809 | 0.988988648 |
| Q9BR76 | Coronin-1B | CORO1B | Homo sapiens | 4.459458382 | -0.11963221 | 0.215459519 | -0.555242166 | 0.578729031 | 0.988988648 |
| P53999 | Activated RNA polymerase II transcriptional coactivator p15 | SUB1 | Homo sapiens | 5.900435483 | 0.097737777 | 0.176501646 | 0.553749949 | 0.579749986 | 0.988988648 |
| O00764 | Pyridoxal kinase | PDXK | Homo sapiens | 1.963985281 | 0.178672436 | 0.322767557 | 0.553563801 | 0.579877406 | 0.988988648 |
| Q14318 | Peptidyl-prolyl cis-trans isomerase FKBP8 | FKBP8 | Homo sapiens | 0.565507925 | -0.37351084 | 0.676003509 | -0.552527961 | 0.580586683 | NA |
| Q14192 | Four and a half LIM domains protein 2 | FHL2 | Homo sapiens | 2.879935031 | -0.1457882 | 0.264311377 | -0.551577469 | 0.581237876 | 0.988988648 |
| Q9UH99 | SUN domain-containing protein 2 | SUN2 | Homo sapiens | 2.78359385 | 0.147067047 | 0.267526949 | 0.54972797 | 0.582505969 | 0.988988648 |
| P49419 | Alpha-aminoadipic semialdehyde dehydrogenase | ALDH7A1 | Homo sapiens | 1.331489337 | -0.209637793 | 0.381514717 | -0.549488089 | 0.582670537 | NA |
| P17987 | T-complex protein 1 subunit alpha | TCP1 | Homo sapiens | 28.80707644 | 0.044809524 | 0.081563365 | 0.549382975 | 0.582742655 | 0.988988648 |
| P04792 | Heat shock protein beta-1 | HSPB1 | Homo sapiens | 26.27151998 | -0.05549337 | 0.101044076 | -0.549199639 | 0.582868452 | 0.988988648 |
| Q14195 | Dihydropyrimidinase-related protein 3 | DPYSL3 | Homo sapiens | 45.29619814 | -0.037264979 | 0.067926133 | -0.548610339 | 0.583272889 | 0.988988648 |
| P41252 | Isoleucine--tRNA ligase, cytoplasmic | IARS1 | Homo sapiens | 12.96326819 | -0.068268246 | 0.124802568 | -0.547009941 | 0.584371903 | 0.988988648 |
| P51114 | Fragile X mental retardation syndrome-related protein 1 | FXR1 | Homo sapiens | 5.394975035 | -0.100692628 | 0.184180787 | -0.546705387 | 0.584581153 | 0.988988648 |
| P54578 | Ubiquitin carboxyl-terminal hydrolase 14 | USP14 | Homo sapiens | 12.33599571 | -0.066563684 | 0.121894512 | -0.546076132 | 0.585013605 | 0.988988648 |
| O60282 | Kinesin heavy chain isoform 5C | KIF5C | Homo sapiens | 1.711509361 | -0.202571208 | 0.372267869 | -0.544154425 | 0.586335211 | 0.988988648 |
| P68402 | Platelet-activating factor acetylhydrolase IB subunit alpha2 | PAFAH1B2 | Homo sapiens | 2.507778728 | 0.153954092 | 0.283249601 | 0.543528009 | 0.586766311 | 0.988988648 |
| Q53EP0 | Fibronectin type III domain-containing protein 3B | FNDC3B | Homo sapiens | 1.008785932 | 0.25014021 | 0.462019501 | 0.541406173 | 0.588227653 | NA |
| O00625 | Pirin | PIR | Homo sapiens | 0.708904757 | -0.286523124 | 0.529259463 | -0.541366086 | 0.588255278 | NA |
| P53582 | Methionine aminopeptidase 1 | METAP1 | Homo sapiens | 0.95914034 | 0.220726423 | 0.407772888 | 0.541297447 | 0.58830258 | NA |
| P40616 | ADP-ribosylation factor-like protein 1 | ARL1 | Homo sapiens | 3.519395877 | -0.124507805 | 0.230698395 | -0.539699483 | 0.589404296 | 0.988988648 |
| P52926 | High mobility group protein HMGI-C | HMGA2 | Homo sapiens | 2.79882375 | 0.142737126 | 0.2649892 | 0.538652618 | 0.590126573 | 0.988988648 |
| P08621 | U1 small nuclear ribonucleoprotein 70 kDa | SNRNP70 | Homo sapiens | 5.951609569 | 0.097420147 | 0.181201867 | 0.537633244 | 0.590830274 | 0.988988648 |
| P55209 | Nucleosome assembly protein 1-like 1 | NAP1L1 | Homo sapiens | 23.76465666 | 0.04681066 | 0.087267445 | 0.53640461 | 0.591678946 | 0.988988648 |
| Q6UB35 | Monofunctional C1-tetrahydrofolate synthase, mitochondrial | MTHFD1L | Homo sapiens | 2.009384561 | 0.173219938 | 0.322951416 | 0.536365314 | 0.591706099 | 0.988988648 |
| P11233 | Ras-related protein Ral-A | RALA | Homo sapiens | 1.127509189 | -0.272108201 | 0.508353776 | -0.535273295 | 0.59246089 | NA |
| P50570 | Dynamin-2 | DNM2 | Homo sapiens | 6.199855815 | 0.090330639 | 0.169918299 | 0.53161219 | 0.59499462 | 0.988988648 |
| Q15008 | 26S proteasome non-ATPase regulatory subunit 6 | PSMD6 | Homo sapiens | 0.644893803 | 0.280604839 | 0.529729589 | 0.529713357 | 0.596310686 | NA |
| P37837 | Transaldolase | TALDO1 | Homo sapiens | 1.705921103 | -0.199800855 | 0.378986738 | -0.527197485 | 0.598056455 | NA |
| P62906 | 60S ribosomal protein L10a | RPL10A | Homo sapiens | 7.583138621 | -0.083458889 | 0.158356435 | -0.527031876 | 0.598171453 | 0.988988648 |
| O43175 | D-3-phosphoglycerate dehydrogenase | PHGDH | Homo sapiens | 23.32416589 | 0.046810563 | 0.088855758 | 0.526815188 | 0.598321935 | 0.988988648 |
| P30154 | Serine/threonine-protein phosphatase 2A 65 kDa regulatory subunit A beta isoform | PPP2R1B | Homo sapiens | 6.636949715 | -0.084925303 | 0.161518203 | -0.525794008 | 0.599031338 | 0.988988648 |
| P48735 | Isocitrate dehydrogenase [NADP], mitochondrial | IDH2 | Homo sapiens | 4.268162679 | -0.110456192 | 0.210141988 | -0.525626472 | 0.59914776 | 0.988988648 |
| Q9Y3F4 | Serine-threonine kinase receptor-associated protein | STRAP | Homo sapiens | 8.199971132 | 0.080648708 | 0.153945024 | 0.523879931 | 0.600362054 | 0.988988648 |
| P27635 | 60S ribosomal protein L10 | RPL10 | Homo sapiens | 15.24082567 | 0.057054378 | 0.109347654 | 0.521770479 | 0.601830147 | 0.988988648 |
| P47712 | Cytosolic phospholipase A2 | PLA2G4A | Homo sapiens | 1.802001073 | -0.175613623 | 0.33697863 | -0.521141721 | 0.602268049 | 0.988988648 |
| P63092 | Guanine nucleotide-binding protein G(s) subunit alpha isoforms short | GNAS | Homo sapiens | 0.766527599 | 0.245553299 | 0.472177376 | 0.52004461 | 0.603032483 | NA |
| O95786 | Antiviral innate immune response receptor RIG-I | DDX58 | Homo sapiens | 0.484742028 | 0.336432217 | 0.647360436 | 0.519698454 | 0.603273765 | NA |
| O15173 | Membrane-associated progesterone receptor component 2 | PGRMC2 | Homo sapiens | 2.088287854 | -0.226965509 | 0.437146533 | -0.519197779 | 0.603622828 | 0.988988648 |
| P57764 | Gasdermin-D | GSDMD | Homo sapiens | 0.53050334 | 0.311317709 | 0.599721462 | 0.519103831 | 0.603688337 | NA |
| O75367 | Core histone macro-H2A.1 | MACROH2A1 | Homo sapiens | 8.756144499 | -0.079855129 | 0.154335955 | -0.517411056 | 0.604869243 | 0.988988648 |
| Q9Y4P3 | Transducin beta-like protein 2 | TBL2 | Homo sapiens | 0.623847706 | -0.278520579 | 0.538649222 | -0.517072275 | 0.605105706 | NA |
| P62333 | 26S proteasome regulatory subunit 10B | PSMC6 | Homo sapiens | 5.698296148 | 0.096372814 | 0.186660885 | 0.516298924 | 0.605645648 | 0.988988648 |
| P0C0S5 | Histone H2A.Z | H2AZ1 | Homo sapiens | 26.10992209 | -0.072884834 | 0.14128434 | -0.515873412 | 0.605942825 | 0.988988648 |
| P15121 | Aldo-keto reductase family 1 member B1 | AKR1B1 | Homo sapiens | 1.075367316 | -0.349753214 | 0.677982762 | -0.515873313 | 0.605942894 | NA |
| P14174 | Macrophage migration inhibitory factor | MIF | Homo sapiens | 3.273473454 | -0.123430804 | 0.240175774 | -0.513918625 | 0.607308885 | 0.988988648 |
| P41219 | Peripherin | PRPH | Homo sapiens | 7.874370049 | 0.082696707 | 0.161061693 | 0.513447398 | 0.607638397 | 0.988988648 |
| Q9H3U1 | Protein unc-45 homolog A | UNC45A | Homo sapiens | 2.123074108 | 0.158391204 | 0.308859152 | 0.51282665 | 0.608072586 | 0.988988648 |
| P20042 | Eukaryotic translation initiation factor 2 subunit 2 | EIF2S2 | Homo sapiens | 2.614663951 | 0.135640725 | 0.264725913 | 0.512381744 | 0.608383865 | 0.988988648 |
| P25705 | ATP synthase subunit alpha, mitochondrial | ATP5F1A | Homo sapiens | 60.23393407 | -0.028518599 | 0.055671018 | -0.512270113 | 0.608461979 | 0.988988648 |
| P46782 | 40S ribosomal protein S5 | RPS5 | Homo sapiens | 9.136841539 | -0.074217401 | 0.14553447 | -0.509964418 | 0.610076391 | 0.988988648 |
| O43150 | Arf-GAP with SH3 domain, ANK repeat and PH domain-containing protein 2 | ASAP2 | Homo sapiens | 3.333666105 | -0.134438912 | 0.263627993 | -0.509956894 | 0.610081662 | 0.988988648 |
| P07437 | Tubulin beta chain | TUBB | Homo sapiens | 272.9463598 | 0.014621322 | 0.028672017 | 0.509950949 | 0.610085827 | 0.988988648 |
| P62753 | 40S ribosomal protein S6 | RPS6 | Homo sapiens | 13.86253999 | 0.059384216 | 0.116754413 | 0.508625028 | 0.611015082 | 0.988988648 |
| O00442 | RNA 3'-terminal phosphate cyclase | RTCA | Homo sapiens | 1.581303242 | 0.177250368 | 0.348830182 | 0.508127959 | 0.611363609 | NA |
| Q9H2M9 | Rab3 GTPase-activating protein non-catalytic subunit | RAB3GAP2 | Homo sapiens | 0.860699348 | -0.232307337 | 0.459993368 | -0.505023231 | 0.61354252 | NA |
| Q16576 | Histone-binding protein RBBP7 | RBBP7 | Homo sapiens | 6.909206193 | 0.088649622 | 0.175826153 | 0.504189057 | 0.614128529 | 0.988988648 |
| P19823 | Inter-alpha-trypsin inhibitor heavy chain H2 | ITIH2 | Homo sapiens | 4.643277542 | -0.100448081 | 0.199388383 | -0.503781012 | 0.614415272 | 0.988988648 |
| P60842 | Eukaryotic initiation factor 4A-I | EIF4A1 | Homo sapiens | 85.75725209 | 0.023811778 | 0.047364017 | 0.50273982 | 0.615147209 | 0.988988648 |
| Q9Y613 | FH1/FH2 domain-containing protein 1 | FHOD1 | Homo sapiens | 0.680120632 | 0.291023008 | 0.579628017 | 0.502085819 | 0.615607155 | NA |
| Q01201 | Transcription factor RelB | RELB | Homo sapiens | 1.39694847 | 0.195410543 | 0.390980101 | 0.499796647 | 0.617218272 | NA |
| P61353 | 60S ribosomal protein L27 | RPL27 | Homo sapiens | 14.46154069 | 0.057698766 | 0.115623961 | 0.499020841 | 0.617764702 | 0.988988648 |
| P62249 | 40S ribosomal protein S16 | RPS16 | Homo sapiens | 20.34430438 | 0.047346563 | 0.094966303 | 0.498561715 | 0.618088182 | 0.988988648 |
| Q08211 | ATP-dependent RNA helicase A | DHX9 | Homo sapiens | 30.344801 | -0.039100661 | 0.078726997 | -0.496661401 | 0.619427846 | 0.988988648 |
| Q10567 | AP-1 complex subunit beta-1 | AP1B1 | Homo sapiens | 8.021141772 | -0.076483578 | 0.154179577 | -0.49606815 | 0.61984633 | 0.988988648 |
| Q9Y4X5 | E3 ubiquitin-protein ligase ARIH1 | ARIH1 | Homo sapiens | 0.771862622 | -0.234738849 | 0.473822233 | -0.495415437 | 0.6203069 | NA |
| P54727 | UV excision repair protein RAD23 homolog B | RAD23B | Homo sapiens | 11.23953543 | 0.0644762 | 0.130557018 | 0.493854727 | 0.62140878 | 0.988988648 |
| Q15155 | Nodal modulator 1 | NOMO1 | Homo sapiens | 1.241079218 | -0.229496877 | 0.465745165 | -0.492752034 | 0.622187807 | NA |
| P00367 | Glutamate dehydrogenase 1, mitochondrial | GLUD1 | Homo sapiens | 13.27877954 | -0.058536749 | 0.118832192 | -0.492600096 | 0.62229518 | 0.988988648 |
| P20290 | Transcription factor BTF3 | BTF3 | Homo sapiens | 12.14375982 | 0.059611301 | 0.121850291 | 0.489217548 | 0.624687688 | 0.988988648 |
| P98194 | Calcium-transporting ATPase type 2C member 1 | ATP2C1 | Homo sapiens | 1.71096638 | 0.171120765 | 0.349912198 | 0.489039155 | 0.624813977 | NA |
| P62424 | 60S ribosomal protein L7a | RPL7A | Homo sapiens | 29.29297556 | 0.038379385 | 0.078850469 | 0.4867363 | 0.626445217 | 0.988988648 |
| P01024 | Complement C3 | C3 | Homo sapiens | 1.999550056 | -0.154489038 | 0.318746827 | -0.484676315 | 0.62790597 | 0.988988648 |
| Q9UHV9 | Prefoldin subunit 2 | PFDN2 | Homo sapiens | 0.95254197 | 0.214191741 | 0.442154871 | 0.484426961 | 0.628082888 | NA |
| P33176 | Kinesin-1 heavy chain | KIF5B | Homo sapiens | 25.41409115 | 0.041968666 | 0.086816643 | 0.483417288 | 0.628799475 | 0.988988648 |
| Q13595 | Transformer-2 protein homolog alpha | TRA2A | Homo sapiens | 2.810288195 | 0.122072144 | 0.252619179 | 0.483225955 | 0.628935308 | 0.988988648 |
| Q08257 | Quinone oxidoreductase | CRYZ | Homo sapiens | 0.393683309 | -0.33193544 | 0.687058732 | -0.483125277 | 0.629006787 | NA |
| P20591 | Interferon-induced GTP-binding protein Mx1 | MX1 | Homo sapiens | 28.92116717 | -0.104198814 | 0.21569342 | -0.48308759 | 0.629033546 | 0.988988648 |
| Q9C0C2 | 182 kDa tankyrase-1-binding protein | TNKS1BP1 | Homo sapiens | 2.531186777 | 0.141212386 | 0.294054367 | 0.480225434 | 0.631067104 | 0.988988648 |
| Q6NZI2 | Caveolae-associated protein 1 | CAVIN1 | Homo sapiens | 29.22453614 | -0.038587781 | 0.080391745 | -0.479996804 | 0.631229665 | 0.988988648 |
| P09874 | Poly [ADP-ribose] polymerase 1 | PARP1 | Homo sapiens | 1.886060619 | 0.166754504 | 0.347456391 | 0.479929306 | 0.631277662 | 0.988988648 |
| Q6PKG0 | La-related protein 1 | LARP1 | Homo sapiens | 1.144154384 | -0.201150931 | 0.419528676 | -0.479468848 | 0.631605124 | NA |
| P01893 | Putative HLA class I histocompatibility antigen, alpha chain H | HLA-H | Homo sapiens | 0.954590113 | -0.21263697 | 0.444427559 | -0.47845136 | 0.632328986 | NA |
| P09543 | 2',3'-cyclic-nucleotide 3'-phosphodiesterase | CNP | Homo sapiens | 3.281024361 | -0.117651378 | 0.246288329 | -0.477697738 | 0.632865354 | 0.988988648 |
| P51858 | Hepatoma-derived growth factor | HDGF | Homo sapiens | 6.273548603 | 0.085115349 | 0.178529984 | 0.476756605 | 0.63353545 | 0.988988648 |
| P84098 | 60S ribosomal protein L19 | RPL19 | Homo sapiens | 2.9685051 | -0.117397095 | 0.246721165 | -0.475829041 | 0.634196178 | 0.988988648 |
| P11766 | Alcohol dehydrogenase class-3 | ADH5 | Homo sapiens | 13.521831 | -0.056924052 | 0.119771254 | -0.475273072 | 0.63459235 | 0.988988648 |
| P14649 | Myosin light chain 6B | MYL6B | Homo sapiens | 2.019089746 | 0.152776605 | 0.321744291 | 0.474838589 | 0.634902025 | 0.988988648 |
| P26640 | Valine--tRNA ligase | VARS1 | Homo sapiens | 14.06957681 | -0.054688575 | 0.115458624 | -0.473663841 | 0.635739643 | 0.988988648 |
| P51148 | Ras-related protein Rab-5C | RAB5C | Homo sapiens | 2.854088275 | -0.12906764 | 0.273010832 | -0.472756482 | 0.636386925 | 0.988988648 |
| Q99798 | Aconitate hydratase, mitochondrial | ACO2 | Homo sapiens | 8.298410925 | 0.07193914 | 0.152561557 | 0.47154173 | 0.637253927 | 0.988988648 |
| O00231 | 26S proteasome non-ATPase regulatory subunit 11 | PSMD11 | Homo sapiens | 4.775000586 | -0.093732056 | 0.198839574 | -0.471395376 | 0.637358417 | 0.988988648 |
| Q9NRX5 | Serine incorporator 1 | SERINC1 | Homo sapiens | 0.845135229 | 0.215433658 | 0.457844719 | 0.470538698 | 0.637970193 | NA |
| Q92538 | Golgi-specific brefeldin A-resistance guanine nucleotide exchange factor 1 | GBF1 | Homo sapiens | 3.943223418 | -0.109263467 | 0.235157651 | -0.46463922 | 0.642189841 | 0.988988648 |
| P08865 | 40S ribosomal protein SA | RPSA | Homo sapiens | 28.17218943 | -0.03747212 | 0.080683755 | -0.464432034 | 0.642338244 | 0.988988648 |
| P13489 | Ribonuclease inhibitor | RNH1 | Homo sapiens | 37.43143149 | -0.036557563 | 0.078728054 | -0.464352434 | 0.642395263 | 0.988988648 |
| O75521 | Enoyl-CoA delta isomerase 2 | ECI2 | Homo sapiens | 1.093919756 | 0.204511617 | 0.440537787 | 0.464231725 | 0.642481734 | NA |
| P41250 | Glycine--tRNA ligase | GARS1 | Homo sapiens | 28.38147939 | 0.037906217 | 0.082106483 | 0.461671421 | 0.644316969 | 0.988988648 |
| O43286 | Beta-1,4-galactosyltransferase 5 | B4GALT5 | Homo sapiens | 0.592763756 | 0.244580075 | 0.530840819 | 0.460740896 | 0.64498451 | NA |
| P62318 | Small nuclear ribonucleoprotein Sm D3 | SNRPD3 | Homo sapiens | 4.568362775 | 0.09838755 | 0.214125383 | 0.459485692 | 0.645885424 | 0.988988648 |
| Q92945 | Far upstream element-binding protein 2 | KHSRP | Homo sapiens | 20.49821684 | -0.0433153 | 0.094328983 | -0.459193966 | 0.646094883 | 0.988988648 |
| P08648 | Integrin alpha-5 | ITGA5 | Homo sapiens | 5.162914859 | -0.08998399 | 0.19607113 | -0.45893544 | 0.646280528 | 0.988988648 |
| Q01518 | Adenylyl cyclase-associated protein 1 | CAP1 | Homo sapiens | 47.54690827 | 0.029787719 | 0.06503516 | 0.458024838 | 0.646934597 | 0.988988648 |
| Q92804 | TATA-binding protein-associated factor 2N | TAF15 | Homo sapiens | 6.945115334 | 0.07711106 | 0.168376899 | 0.457966981 | 0.646976163 | 0.988988648 |
| P54687 | Branched-chain-amino-acid aminotransferase, cytosolic | BCAT1 | Homo sapiens | 3.195973736 | 0.115890118 | 0.253302316 | 0.45751701 | 0.647299479 | 0.988988648 |
| P12235 | ADP/ATP translocase 1 | SLC25A4 | Homo sapiens | 29.86491831 | -0.036963082 | 0.081016749 | -0.456240011 | 0.648217397 | 0.988988648 |
| Q96JB1 | Dynein axonemal heavy chain 8 | DNAH8 | Homo sapiens | 0.863657079 | -0.226664912 | 0.497844072 | -0.455292981 | 0.648898475 | NA |
| P26447 | Protein S100-A4 | S100A4 | Homo sapiens | 1.478231883 | -0.154391769 | 0.339457719 | -0.454818848 | 0.649239569 | NA |
| O43837 | Isocitrate dehydrogenase [NAD] subunit beta, mitochondrial | IDH3B | Homo sapiens | 0.888926973 | -0.214143958 | 0.470995224 | -0.454662695 | 0.649351923 | NA |
| O95336 | 6-phosphogluconolactonase | PGLS | Homo sapiens | 1.306174275 | 0.177786403 | 0.391569628 | 0.454035222 | 0.649803476 | NA |
| P62899 | 60S ribosomal protein L31 | RPL31 | Homo sapiens | 8.02361743 | 0.06847603 | 0.150887569 | 0.453821549 | 0.649957272 | 0.988988648 |
| Q9NY33 | Dipeptidyl peptidase 3 | DPP3 | Homo sapiens | 7.970150678 | -0.070602345 | 0.155924895 | -0.452797132 | 0.650694829 | 0.988988648 |
| Q96I24 | Far upstream element-binding protein 3 | FUBP3 | Homo sapiens | 0.995128833 | -0.2170249 | 0.47949599 | -0.45261046 | 0.650829266 | NA |
| P30520 | Adenylosuccinate synthetase isozyme 2 | ADSS2 | Homo sapiens | 8.522789501 | -0.06636976 | 0.146783023 | -0.452162375 | 0.651152011 | 0.988988648 |
| P11413 | Glucose-6-phosphate 1-dehydrogenase | G6PD | Homo sapiens | 35.04939854 | -0.033890267 | 0.074986986 | -0.451948648 | 0.651305977 | 0.988988648 |
| P53007 | Tricarboxylate transport protein, mitochondrial | SLC25A1 | Homo sapiens | 0.725651745 | -0.227958242 | 0.504684402 | -0.451684738 | 0.651496114 | NA |
| P61011 | Signal recognition particle 54 kDa protein | SRP54 | Homo sapiens | 1.019218309 | -0.199769561 | 0.442344574 | -0.451615264 | 0.651546172 | NA |
| Q15459 | Splicing factor 3A subunit 1 | SF3A1 | Homo sapiens | 6.989967596 | 0.073493281 | 0.162962863 | 0.450981774 | 0.652002685 | 0.988988648 |
| P49902 | Cytosolic purine 5'-nucleotidase | NT5C2 | Homo sapiens | 1.286766698 | 0.19366847 | 0.429600119 | 0.450811026 | 0.652125754 | NA |
| P63162 | Small nuclear ribonucleoprotein-associated protein N | SNRPN | Homo sapiens | 2.133108308 | 0.139041872 | 0.309683434 | 0.448980659 | 0.653445609 | 0.988988648 |
| P35998 | 26S proteasome regulatory subunit 7 | PSMC2 | Homo sapiens | 12.92580156 | -0.054893047 | 0.12234926 | -0.448658598 | 0.653677955 | 0.988988648 |
| P20810 | Calpastatin | CAST | Homo sapiens | 12.78328303 | 0.056127321 | 0.125346977 | 0.447775625 | 0.654315137 | 0.988988648 |
| Q9Y6K8 | Adenylate kinase isoenzyme 5 | AK5 | Homo sapiens | 0.923575378 | -0.212552777 | 0.474974049 | -0.44750398 | 0.654511215 | NA |
| P29279 | CCN family member 2 | CCN2 | Homo sapiens | 3.170189345 | -0.207685761 | 0.46483331 | -0.446796211 | 0.655022208 | 0.988988648 |
| Q13177 | Serine/threonine-protein kinase PAK 2 | PAK2 | Homo sapiens | 2.041996746 | 0.139038266 | 0.311282783 | 0.446662242 | 0.655118948 | 0.988988648 |
| A6NHL2 | Tubulin alpha chain-like 3 | TUBAL3 | Homo sapiens | 21.14489212 | 0.041760873 | 0.093669041 | 0.44583432 | 0.655716929 | 0.988988648 |
| P22392 | Nucleoside diphosphate kinase B | NME2 | Homo sapiens | 25.12773237 | 0.044765135 | 0.100449904 | 0.445646374 | 0.655852707 | 0.988988648 |
| P13639 | Elongation factor 2 | EEF2 | Homo sapiens | 278.3357058 | 0.014080153 | 0.031596699 | 0.445621007 | 0.655871033 | 0.988988648 |
| P14866 | Heterogeneous nuclear ribonucleoprotein L | HNRNPL | Homo sapiens | 13.66635777 | 0.05151231 | 0.115619475 | 0.445533163 | 0.6559345 | 0.988988648 |
| P61088 | Ubiquitin-conjugating enzyme E2 N | UBE2N | Homo sapiens | 6.306755682 | -0.080429148 | 0.18070839 | -0.445076998 | 0.656264111 | 0.988988648 |
| Q96TA1 | Protein Niban 2 | NIBAN2 | Homo sapiens | 13.37021544 | -0.052333022 | 0.117907448 | -0.443848314 | 0.657152255 | 0.988988648 |
| O76021 | Ribosomal L1 domain-containing protein 1 | RSL1D1 | Homo sapiens | 2.742554683 | -0.121031846 | 0.272997702 | -0.443343828 | 0.657517059 | 0.988988648 |
| P67809 | Y-box-binding protein 1 | YBX1 | Homo sapiens | 36.04266163 | 0.031964841 | 0.072172001 | 0.44289808 | 0.657839456 | 0.988988648 |
| P05388 | 60S acidic ribosomal protein P0 | RPLP0 | Homo sapiens | 36.18135623 | 0.031245646 | 0.071033328 | 0.439873041 | 0.660029062 | 0.988988648 |
| Q14004 | Cyclin-dependent kinase 13 | CDK13 | Homo sapiens | 1.805733286 | 0.142941551 | 0.325674066 | 0.438909837 | 0.660726869 | 0.988988648 |
| Q96CV9 | Optineurin | OPTN | Homo sapiens | 1.62668597 | 0.152867113 | 0.350737537 | 0.435844746 | 0.662949376 | NA |
| Q13557 | Calcium/calmodulin-dependent protein kinase type II subunit delta | CAMK2D | Homo sapiens | 0.906948843 | -0.195581421 | 0.448965532 | -0.435626806 | 0.663107519 | NA |
| P52306 | Rap1 GTPase-GDP dissociation stimulator 1 | RAP1GDS1 | Homo sapiens | 0.936431733 | 0.203913608 | 0.468587504 | 0.435166551 | 0.663441539 | NA |
| P49773 | Histidine triad nucleotide-binding protein 1 | HINT1 | Homo sapiens | 1.818950877 | 0.150405656 | 0.34616928 | 0.434485856 | 0.663935664 | 0.988988648 |
| P30405 | Peptidyl-prolyl cis-trans isomerase F, mitochondrial | PPIF | Homo sapiens | 0.61523591 | -0.222324695 | 0.514132551 | -0.432426802 | 0.665431242 | NA |
| Q96B97 | SH3 domain-containing kinase-binding protein 1 | SH3KBP1 | Homo sapiens | 3.996655217 | 0.091707483 | 0.213004325 | 0.430542822 | 0.666800824 | 0.988988648 |
| Q15758 | Neutral amino acid transporter B(0) | SLC1A5 | Homo sapiens | 10.58010168 | -0.06064519 | 0.140942417 | -0.43028345 | 0.666989464 | 0.988988648 |
| P22059 | Oxysterol-binding protein 1 | OSBP | Homo sapiens | 1.142287397 | 0.174561199 | 0.406351897 | 0.429581357 | 0.667500201 | NA |
| O95793 | Double-stranded RNA-binding protein Staufen homolog 1 | STAU1 | Homo sapiens | 0.931608802 | -0.194690851 | 0.455430996 | -0.427487045 | 0.669024619 | NA |
| P69892 | Hemoglobin subunit gamma-2 | HBG2 | Homo sapiens | 0.859920264 | 0.186747012 | 0.437922934 | 0.426438074 | 0.669788663 | NA |
| P26373 | 60S ribosomal protein L13 | RPL13 | Homo sapiens | 9.865887928 | 0.060837129 | 0.142759149 | 0.426152226 | 0.669996927 | 0.988988648 |
| E9PAV3 | Nascent polypeptide-associated complex subunit alpha, muscle-specific form | NACA | Homo sapiens | 21.23342254 | 0.039101632 | 0.091795675 | 0.42596377 | 0.670134246 | 0.988988648 |
| O60716 | Catenin delta-1 | CTNND1 | Homo sapiens | 2.738275607 | -0.117235362 | 0.275820389 | -0.425042408 | 0.670805761 | 0.988988648 |
| Q8NCA5 | Protein FAM98A | FAM98A | Homo sapiens | 2.522814634 | -0.118055219 | 0.279002684 | -0.423132916 | 0.672198288 | 0.988988648 |
| O43242 | 26S proteasome non-ATPase regulatory subunit 3 | PSMD3 | Homo sapiens | 7.137710446 | -0.068004544 | 0.160793059 | -0.422932087 | 0.672344811 | 0.988988648 |
| P61160 | Actin-related protein 2 | ACTR2 | Homo sapiens | 14.80505505 | -0.048190461 | 0.11409408 | -0.422374768 | 0.672751492 | 0.988988648 |
| P14735 | Insulin-degrading enzyme | IDE | Homo sapiens | 1.962527125 | 0.139708205 | 0.330813897 | 0.422316615 | 0.672793932 | 0.988988648 |
| Q32MZ4 | Leucine-rich repeat flightless-interacting protein 1 | LRRFIP1 | Homo sapiens | 1.672160709 | 0.141503309 | 0.336447632 | 0.420580486 | 0.674061446 | NA |
| Q12888 | TP53-binding protein 1 | TP53BP1 | Homo sapiens | 1.019634897 | 0.212624227 | 0.506172817 | 0.420062515 | 0.674439785 | NA |
| O75369 | Filamin-B | FLNB | Homo sapiens | 109.9718269 | -0.018712037 | 0.044661972 | -0.418970244 | 0.675237879 | 0.988988648 |
| Q9GZS3 | WD repeat-containing protein 61 | WDR61 | Homo sapiens | 1.507377208 | 0.152210091 | 0.363424522 | 0.418821742 | 0.675346414 | NA |
| Q99460 | 26S proteasome non-ATPase regulatory subunit 1 | PSMD1 | Homo sapiens | 11.31440196 | -0.053835497 | 0.128654706 | -0.418449495 | 0.675618506 | 0.988988648 |
| Q8TAT6 | Nuclear protein localization protein 4 homolog | NPLOC4 | Homo sapiens | 4.432702654 | 0.089054972 | 0.212839112 | 0.418414508 | 0.675644081 | 0.988988648 |
| O75436 | Vacuolar protein sorting-associated protein 26A | VPS26A | Homo sapiens | 2.846444652 | -0.105592148 | 0.252625107 | -0.417979625 | 0.675962014 | 0.988988648 |
| P52701 | DNA mismatch repair protein Msh6 | MSH6 | Homo sapiens | 0.756152761 | 0.188303331 | 0.451124776 | 0.417408532 | 0.676379615 | NA |
| O75477 | Erlin-1 | ERLIN1 | Homo sapiens | 3.91190079 | 0.093485885 | 0.224118053 | 0.417127867 | 0.676584883 | 0.988988648 |
| Q8WVV9 | Heterogeneous nuclear ribonucleoprotein L-like | HNRNPLL | Homo sapiens | 1.316571222 | -0.153833398 | 0.369262575 | -0.416596233 | 0.676973765 | NA |
| P15259 | Phosphoglycerate mutase 2 | PGAM2 | Homo sapiens | 1.244569059 | 0.168053371 | 0.40341539 | 0.4165765 | 0.676988201 | NA |
| P02538 | Keratin, type II cytoskeletal 6A | KRT6A | Homo sapiens | 5.699020243 | -0.080767937 | 0.194448753 | -0.415368757 | 0.677871975 | 0.988988648 |
| P00734 | Prothrombin | F2 | Homo sapiens | 1.545048172 | -0.14769828 | 0.356756117 | -0.414003497 | 0.678871545 | NA |
| P22695 | Cytochrome b-c1 complex subunit 2, mitochondrial | UQCRC2 | Homo sapiens | 6.356760186 | -0.073100453 | 0.176726754 | -0.413635466 | 0.679141096 | 0.988988648 |
| Q04917 | 14-3-3 protein eta | YWHAH | Homo sapiens | 23.39200531 | -0.036299916 | 0.088303239 | -0.411082505 | 0.681012038 | 0.988988648 |
| Q6P5R6 | 60S ribosomal protein L22-like 1 | RPL22L1 | Homo sapiens | 1.564208037 | 0.148649201 | 0.361617939 | 0.411067 | 0.681023406 | NA |
| P28074 | Proteasome subunit beta type-5 | PSMB5 | Homo sapiens | 2.055177599 | -0.132193275 | 0.322270154 | -0.410193973 | 0.681663662 | 0.988988648 |
| Q92882 | Osteoclast-stimulating factor 1 | OSTF1 | Homo sapiens | 0.74286607 | 0.189686755 | 0.462842295 | 0.409830211 | 0.681930503 | NA |
| Q5H9R7 | Serine/threonine-protein phosphatase 6 regulatory subunit 3 | PPP6R3 | Homo sapiens | 1.907163325 | 0.132859273 | 0.324546655 | 0.409368796 | 0.682269036 | 0.988988648 |
| O14983 | Sarcoplasmic/endoplasmic reticulum calcium ATPase 1 | ATP2A1 | Homo sapiens | 2.259762361 | -0.132502007 | 0.324383536 | -0.40847328 | 0.682926243 | 0.988988648 |
| P48047 | ATP synthase subunit O, mitochondrial | ATP5PO | Homo sapiens | 1.829370833 | 0.134022108 | 0.328564222 | 0.40790232 | 0.683345389 | 0.988988648 |
| P30613 | Pyruvate kinase PKLR | PKLR | Homo sapiens | 8.932674951 | 0.05870444 | 0.143934462 | 0.407855351 | 0.683379874 | 0.988988648 |
| P00568 | Adenylate kinase isoenzyme 1 | AK1 | Homo sapiens | 3.71499922 | -0.090822565 | 0.222858185 | -0.407535245 | 0.683614913 | 0.988988648 |
| Q9Y5X1 | Sorting nexin-9 | SNX9 | Homo sapiens | 2.321002865 | -0.117265016 | 0.287906325 | -0.407302673 | 0.6837857 | 0.988988648 |
| Q00169 | Phosphatidylinositol transfer protein alpha isoform | PITPNA | Homo sapiens | 0.906844147 | 0.180946553 | 0.444833694 | 0.406773489 | 0.68417436 | NA |
| Q9HD45 | Transmembrane 9 superfamily member 3 | TM9SF3 | Homo sapiens | 1.342986526 | 0.163058663 | 0.40131234 | 0.406313604 | 0.684512191 | NA |
| Q9Y639 | Neuroplastin | NPTN | Homo sapiens | 0.759048763 | -0.201771963 | 0.496885176 | -0.406073621 | 0.684688507 | NA |
| Q16851 | UTP--glucose-1-phosphate uridylyltransferase | UGP2 | Homo sapiens | 15.82190863 | -0.046298692 | 0.114063227 | -0.405903754 | 0.684813319 | 0.988988648 |
| P50914 | 60S ribosomal protein L14 | RPL14 | Homo sapiens | 13.40208442 | 0.047645633 | 0.117422853 | 0.40576116 | 0.684918099 | 0.988988648 |
| Q96JY6 | PDZ and LIM domain protein 2 | PDLIM2 | Homo sapiens | 0.735648048 | -0.188226106 | 0.464018049 | -0.405643933 | 0.685004243 | NA |
| Q9BRA2 | Thioredoxin domain-containing protein 17 | TXNDC17 | Homo sapiens | 1.437727239 | -0.149775095 | 0.369331369 | -0.405530393 | 0.685087682 | NA |
| Q63HN8 | E3 ubiquitin-protein ligase RNF213 | RNF213 | Homo sapiens | 2.863818548 | -0.14192318 | 0.350463108 | -0.404958973 | 0.685507669 | 0.988988648 |
| P02788 | Lactotransferrin | LTF | Homo sapiens | 9.656244819 | -0.055579856 | 0.137290481 | -0.404834008 | 0.685599529 | 0.988988648 |
| Q15819 | Ubiquitin-conjugating enzyme E2 variant 2 | UBE2V2 | Homo sapiens | 1.558144675 | 0.140242429 | 0.346581128 | 0.404645314 | 0.685738245 | NA |
| P13637 | Sodium/potassium-transporting ATPase subunit alpha-3 | ATP1A3 | Homo sapiens | 2.259235128 | -0.129747815 | 0.322296502 | -0.402572829 | 0.687262501 | 0.988988648 |
| P11021 | Endoplasmic reticulum chaperone BiP | HSPA5 | Homo sapiens | 125.0434617 | 0.01830617 | 0.045583475 | 0.401596633 | 0.687980908 | 0.988988648 |
| Q15056 | Eukaryotic translation initiation factor 4H | EIF4H | Homo sapiens | 3.117346193 | -0.110402394 | 0.274933727 | -0.401560024 | 0.688007855 | 0.988988648 |
| P07947 | Tyrosine-protein kinase Yes | YES1 | Homo sapiens | 1.473846251 | 0.140715045 | 0.350455672 | 0.401520239 | 0.68803714 | NA |
| P46379 | Large proline-rich protein BAG6 | BAG6 | Homo sapiens | 3.827325314 | -0.089872555 | 0.224022647 | -0.401176204 | 0.688290399 | 0.988988648 |
| P46940 | Ras GTPase-activating-like protein IQGAP1 | IQGAP1 | Homo sapiens | 70.80983696 | -0.021432386 | 0.053482706 | -0.400734888 | 0.688615322 | 0.988988648 |
| P46063 | ATP-dependent DNA helicase Q1 | RECQL | Homo sapiens | 2.888513174 | -0.104204449 | 0.260750371 | -0.399632986 | 0.689426858 | 0.988988648 |
| P53801 | Pituitary tumor-transforming gene 1 protein-interacting protein | PTTG1IP | Homo sapiens | 3.29329331 | -0.096215286 | 0.240960506 | -0.399298987 | 0.689672914 | 0.988988648 |
| Q9P0M6 | Core histone macro-H2A.2 | MACROH2A2 | Homo sapiens | 4.240312871 | -0.087464649 | 0.219928438 | -0.397695952 | 0.690854322 | 0.988988648 |
| Q7L576 | Cytoplasmic FMR1-interacting protein 1 | CYFIP1 | Homo sapiens | 4.126564991 | -0.082962056 | 0.208982636 | -0.396980619 | 0.691381753 | 0.988988648 |
| O43491 | Band 4.1-like protein 2 | EPB41L2 | Homo sapiens | 3.260378093 | 0.0945101 | 0.238126777 | 0.396889847 | 0.691448691 | 0.988988648 |
| Q96AG4 | Leucine-rich repeat-containing protein 59 | LRRC59 | Homo sapiens | 17.31864799 | -0.042159578 | 0.106352991 | -0.39641178 | 0.691801277 | 0.988988648 |
| Q9Y512 | Sorting and assembly machinery component 50 homolog | SAMM50 | Homo sapiens | 0.64488092 | -0.22471684 | 0.567643297 | -0.39587685 | 0.69219588 | NA |
| P05161 | Ubiquitin-like protein ISG15 | ISG15 | Homo sapiens | 3.639472874 | -0.133191413 | 0.336658466 | -0.395627695 | 0.692379703 | 0.988988648 |
| Q96PU5 | E3 ubiquitin-protein ligase NEDD4-like | NEDD4L | Homo sapiens | 2.04269636 | 0.124971991 | 0.31736874 | 0.393775363 | 0.693746895 | 0.988988648 |
| P07900 | Heat shock protein HSP 90-alpha | HSP90AA1 | Homo sapiens | 180.061392 | 0.017241677 | 0.043968244 | 0.392139313 | 0.694955281 | 0.988988648 |
| P38919 | Eukaryotic initiation factor 4A-III | EIF4A3 | Homo sapiens | 5.730221321 | 0.069254427 | 0.17726729 | 0.390677982 | 0.696035276 | 0.988988648 |
| Q9BWF3 | RNA-binding protein 4 | RBM4 | Homo sapiens | 1.296088573 | 0.147961396 | 0.379635658 | 0.389745782 | 0.696724539 | NA |
| P61619 | Protein transport protein Sec61 subunit alpha isoform 1 | SEC61A1 | Homo sapiens | 3.819507686 | 0.088737706 | 0.227993292 | 0.389211914 | 0.697119391 | 0.988988648 |
| Q96AC1 | Fermitin family homolog 2 | FERMT2 | Homo sapiens | 5.519463406 | 0.071667633 | 0.184283841 | 0.388898082 | 0.697351541 | 0.988988648 |
| P48147 | Prolyl endopeptidase | PREP | Homo sapiens | 8.522147023 | -0.057140039 | 0.147973664 | -0.386150055 | 0.699385541 | 0.988988648 |
| P07686 | Beta-hexosaminidase subunit beta | HEXB | Homo sapiens | 1.77347874 | -0.127522 | 0.330362397 | -0.386006402 | 0.699491928 | 0.988988648 |
| Q13561 | Dynactin subunit 2 | DCTN2 | Homo sapiens | 3.605375493 | 0.09462288 | 0.245674797 | 0.385155013 | 0.700122572 | 0.988988648 |
| O75170 | Serine/threonine-protein phosphatase 6 regulatory subunit 2 | PPP6R2 | Homo sapiens | 1.133433394 | -0.159153497 | 0.414218448 | -0.384225998 | 0.700810951 | NA |
| Q96KP4 | Cytosolic non-specific dipeptidase | CNDP2 | Homo sapiens | 6.890775765 | 0.065093038 | 0.169717702 | 0.383537118 | 0.701321555 | 0.988988648 |
| P19367 | Hexokinase-1 | HK1 | Homo sapiens | 12.17022569 | -0.050119096 | 0.130921058 | -0.382819213 | 0.701853815 | 0.988988648 |
| Q14157 | Ubiquitin-associated protein 2-like | UBAP2L | Homo sapiens | 9.54945751 | 0.055103421 | 0.144101742 | 0.382392473 | 0.702170273 | 0.988988648 |
| Q4KWH8 | 1-phosphatidylinositol 4,5-bisphosphate phosphodiesterase eta-1 | PLCH1 | Homo sapiens | 0.759202363 | 0.19574481 | 0.513070462 | 0.381516428 | 0.702820084 | NA |
| P63173 | 60S ribosomal protein L38 | RPL38 | Homo sapiens | 2.44482975 | -0.121830491 | 0.319403635 | -0.381431136 | 0.702883361 | 0.988988648 |
| Q12840 | Kinesin heavy chain isoform 5A | KIF5A | Homo sapiens | 3.424146087 | 0.094582121 | 0.247971216 | 0.381423791 | 0.702888811 | 0.988988648 |
| Q5JTV8 | Torsin-1A-interacting protein 1 | TOR1AIP1 | Homo sapiens | 0.738846936 | -0.18406334 | 0.483823987 | -0.380434507 | 0.703622904 | NA |
| P07196 | Neurofilament light polypeptide | NEFL | Homo sapiens | 2.028473519 | -0.112619282 | 0.296106209 | -0.380334079 | 0.703697441 | 0.988988648 |
| P56945 | Breast cancer anti-estrogen resistance protein 1 | BCAR1 | Homo sapiens | 0.613491925 | 0.209758956 | 0.551692267 | 0.380210072 | 0.703789484 | NA |
| Q14699 | Raftlin | RFTN1 | Homo sapiens | 1.011769532 | 0.168383537 | 0.443089522 | 0.380021483 | 0.703929468 | NA |
| P36957 | Dihydrolipoyllysine-residue succinyltransferase component of 2-oxoglutarate dehydrogenase complex, mitochondrial | DLST | Homo sapiens | 0.537246772 | -0.243619028 | 0.641688053 | -0.379653364 | 0.704202742 | NA |
| P61289 | Proteasome activator complex subunit 3 | PSME3 | Homo sapiens | 0.867984802 | -0.199463123 | 0.525476355 | -0.379585344 | 0.704253242 | NA |
| Q9Y2W2 | WW domain-binding protein 11 | WBP11 | Homo sapiens | 0.763960669 | 0.167837333 | 0.443761716 | 0.37821499 | 0.705270891 | NA |
| Q99613 | Eukaryotic translation initiation factor 3 subunit C | EIF3C | Homo sapiens | 13.89745415 | 0.043228728 | 0.11478483 | 0.376606633 | 0.706465958 | 0.988988648 |
| Q96A33 | PAT complex subunit CCDC47 | CCDC47 | Homo sapiens | 1.143403267 | -0.159980011 | 0.425276246 | -0.376179042 | 0.706783795 | NA |
| Q8NF91 | Nesprin-1 | SYNE1 | Homo sapiens | 3.249826726 | -0.092988577 | 0.247453805 | -0.375781563 | 0.707079296 | 0.988988648 |
| P62263 | 40S ribosomal protein S14 | RPS14 | Homo sapiens | 10.65520137 | 0.048710217 | 0.129678337 | 0.375623392 | 0.707196898 | 0.988988648 |
| P11387 | DNA topoisomerase 1 | TOP1 | Homo sapiens | 3.412158783 | 0.086282816 | 0.230940557 | 0.373614826 | 0.708690902 | 0.988988648 |
| P17980 | 26S proteasome regulatory subunit 6A | PSMC3 | Homo sapiens | 7.861160452 | 0.056553043 | 0.151457615 | 0.373391877 | 0.708856804 | 0.988988648 |
| Q92900 | Regulator of nonsense transcripts 1 | UPF1 | Homo sapiens | 4.91598563 | 0.078407203 | 0.210167239 | 0.373070529 | 0.709095952 | 0.988988648 |
| Q9H0C2 | ADP/ATP translocase 4 | SLC25A31 | Homo sapiens | 0.613943558 | -0.276293868 | 0.743758908 | -0.371483105 | 0.710277738 | NA |
| P62854 | 40S ribosomal protein S26 | RPS26 | Homo sapiens | 9.994512933 | -0.05166996 | 0.139101403 | -0.371455347 | 0.710298409 | 0.988988648 |
| Q13641 | Trophoblast glycoprotein | TPBG | Homo sapiens | 1.856079238 | -0.122803265 | 0.331605145 | -0.370329795 | 0.711136777 | 0.988988648 |
| Q9H0D6 | 5'-3' exoribonuclease 2 | XRN2 | Homo sapiens | 0.881278028 | 0.180693638 | 0.489708563 | 0.368981986 | 0.712141152 | NA |
| Q9UJU6 | Drebrin-like protein | DBNL | Homo sapiens | 7.030531441 | 0.061407954 | 0.166483097 | 0.368853984 | 0.712236564 | 0.988988648 |
| P55795 | Heterogeneous nuclear ribonucleoprotein H2 | HNRNPH2 | Homo sapiens | 16.49947814 | -0.03965551 | 0.107834849 | -0.367742992 | 0.713064881 | 0.988988648 |
| P40926 | Malate dehydrogenase, mitochondrial | MDH2 | Homo sapiens | 37.5857075 | 0.025618846 | 0.069703514 | 0.367540236 | 0.713216085 | 0.988988648 |
| Q8TDB4 | Protein MGARP | MGARP | Homo sapiens | 0.683913995 | -0.194109528 | 0.530296638 | -0.366039522 | 0.714335585 | NA |
| Q969V3 | Nicalin | NCLN | Homo sapiens | 0.683812259 | -0.183919937 | 0.503883782 | -0.365004676 | 0.715107916 | NA |
| P53004 | Biliverdin reductase A | BLVRA | Homo sapiens | 0.733330207 | -0.198538543 | 0.545014362 | -0.364281305 | 0.715647959 | NA |
| Q9BWM7 | Sideroflexin-3 | SFXN3 | Homo sapiens | 1.210175955 | -0.144021439 | 0.395905916 | -0.363776931 | 0.71602459 | NA |
| Q14204 | Cytoplasmic dynein 1 heavy chain 1 | DYNC1H1 | Homo sapiens | 104.0498283 | -0.01813976 | 0.049933625 | -0.363277457 | 0.716397632 | 0.988988648 |
| Q96G03 | Phosphoglucomutase-2 | PGM2 | Homo sapiens | 1.946272324 | 0.115691137 | 0.319120064 | 0.362531692 | 0.716954745 | 0.988988648 |
| Q9UBF2 | Coatomer subunit gamma-2 | COPG2 | Homo sapiens | 2.606860426 | 0.098991679 | 0.273778157 | 0.36157625 | 0.717668715 | 0.988988648 |
| Q96BY6 | Dedicator of cytokinesis protein 10 | DOCK10 | Homo sapiens | 0.705488933 | 0.20044816 | 0.55532393 | 0.360957181 | 0.718131457 | NA |
| P46459 | Vesicle-fusing ATPase | NSF | Homo sapiens | 1.873568962 | 0.120837894 | 0.335810885 | 0.359839122 | 0.718967445 | 0.988988648 |
| P48444 | Coatomer subunit delta | ARCN1 | Homo sapiens | 12.68488953 | 0.043492331 | 0.121047191 | 0.359300625 | 0.719370207 | 0.988988648 |
| P22090 | 40S ribosomal protein S4, Y isoform 1 | RPS4Y1 | Homo sapiens | 2.398775952 | 0.105848246 | 0.294748674 | 0.359113561 | 0.719510137 | 0.988988648 |
| Q9Y570 | Protein phosphatase methylesterase 1 | PPME1 | Homo sapiens | 1.422082692 | -0.137264899 | 0.383494169 | -0.357932167 | 0.720394077 | NA |
| P49589 | Cysteine--tRNA ligase, cytoplasmic | CARS1 | Homo sapiens | 3.617000607 | 0.087127301 | 0.243434259 | 0.357908952 | 0.720411451 | 0.988988648 |
| P48507 | Glutamate--cysteine ligase regulatory subunit | GCLM | Homo sapiens | 1.575719618 | 0.126528768 | 0.353605508 | 0.357824653 | 0.72047454 | NA |
| P19623 | Spermidine synthase | SRM | Homo sapiens | 8.167480333 | -0.054590727 | 0.153175403 | -0.356393558 | 0.721545853 | 0.988988648 |
| Q5JQF8 | Polyadenylate-binding protein 1-like 2 | PABPC1L2A | Homo sapiens | 0.944237058 | -0.153586076 | 0.431834154 | -0.355659863 | 0.722095307 | NA |
| Q15286 | Ras-related protein Rab-35 | RAB35 | Homo sapiens | 1.598542142 | 0.116566969 | 0.327773064 | 0.355633156 | 0.72211531 | NA |
| O00461 | Golgi integral membrane protein 4 | GOLIM4 | Homo sapiens | 0.508343829 | -0.254570139 | 0.716017344 | -0.355536275 | 0.722187874 | NA |
| Q9NSD9 | Phenylalanine--tRNA ligase beta subunit | FARSB | Homo sapiens | 5.026716757 | 0.069319887 | 0.195176069 | 0.355165917 | 0.722465297 | 0.988988648 |
| P40227 | T-complex protein 1 subunit zeta | CCT6A | Homo sapiens | 34.61039468 | 0.026094976 | 0.07374162 | 0.353870396 | 0.723436014 | 0.988988648 |
| Q01081 | Splicing factor U2AF 35 kDa subunit | U2AF1 | Homo sapiens | 0.969511062 | -0.153292433 | 0.433363162 | -0.353727419 | 0.723543172 | NA |
| O95817 | BAG family molecular chaperone regulator 3 | BAG3 | Homo sapiens | 4.275934271 | 0.074335522 | 0.210284685 | 0.353499456 | 0.723714036 | 0.988988648 |
| P02042 | Hemoglobin subunit delta | HBD | Homo sapiens | 3.372991788 | -0.082840436 | 0.234815313 | -0.35278975 | 0.724246069 | 0.988988648 |
| P49720 | Proteasome subunit beta type-3 | PSMB3 | Homo sapiens | 2.145466725 | -0.123395974 | 0.350652715 | -0.351903659 | 0.724910515 | 0.988988648 |
| Q07954 | Prolow-density lipoprotein receptor-related protein 1 | LRP1 | Homo sapiens | 14.71475511 | -0.042757636 | 0.121652025 | -0.351474925 | 0.725232081 | 0.988988648 |
| P13647 | Keratin, type II cytoskeletal 5 | KRT5 | Homo sapiens | 5.135281887 | -0.070007665 | 0.199382508 | -0.351122402 | 0.725496522 | 0.988988648 |
| Q6DKJ4 | Nucleoredoxin | NXN | Homo sapiens | 3.967834031 | 0.087023988 | 0.24800312 | 0.350898763 | 0.725664299 | 0.988988648 |
| P16615 | Sarcoplasmic/endoplasmic reticulum calcium ATPase 2 | ATP2A2 | Homo sapiens | 14.17752925 | -0.040389206 | 0.115821268 | -0.348720112 | 0.727299444 | 0.988988648 |
| P53621 | Coatomer subunit alpha | COPA | Homo sapiens | 24.00481597 | -0.031040544 | 0.089368224 | -0.347333122 | 0.72834107 | 0.988988648 |
| P30153 | Serine/threonine-protein phosphatase 2A 65 kDa regulatory subunit A alpha isoform | PPP2R1A | Homo sapiens | 23.17162168 | 0.03088136 | 0.089173877 | 0.346305001 | 0.729113511 | 0.988988648 |
| Q01814 | Plasma membrane calcium-transporting ATPase 2 | ATP2B2 | Homo sapiens | 0.608507638 | 0.18526318 | 0.536222509 | 0.345496835 | 0.72972089 | NA |
| P32969 | 60S ribosomal protein L9 | RPL9 | Homo sapiens | 17.83500002 | 0.035388029 | 0.102461271 | 0.345379559 | 0.729809043 | 0.988988648 |
| P61018 | Ras-related protein Rab-4B | RAB4B | Homo sapiens | 3.478234972 | -0.081073172 | 0.234933244 | -0.345090249 | 0.730026524 | 0.988988648 |
| Q99961 | Endophilin-A2 | SH3GL1 | Homo sapiens | 3.588813839 | 0.080675587 | 0.234498579 | 0.344034438 | 0.730820387 | 0.988988648 |
| Q16850 | Lanosterol 14-alpha demethylase | CYP51A1 | Homo sapiens | 1.747587082 | -0.117334852 | 0.341619201 | -0.343466793 | 0.731247318 | 0.988988648 |
| P61247 | 40S ribosomal protein S3a | RPS3A | Homo sapiens | 31.9295191 | 0.02603895 | 0.075822755 | 0.343418671 | 0.731283515 | 0.988988648 |
| Q02880 | DNA topoisomerase 2-beta | TOP2B | Homo sapiens | 1.511117936 | -0.138197393 | 0.402689439 | -0.343186038 | 0.731458507 | NA |
| Q86X55 | Histone-arginine methyltransferase CARM1 | CARM1 | Homo sapiens | 0.722222025 | -0.172837889 | 0.503653842 | -0.343168015 | 0.731472066 | NA |
| Q14203 | Dynactin subunit 1 | DCTN1 | Homo sapiens | 8.791636451 | 0.051557848 | 0.150480366 | 0.342621761 | 0.731883029 | 0.988988648 |
| P17844 | Probable ATP-dependent RNA helicase DDX5 | DDX5 | Homo sapiens | 31.22475787 | -0.027076628 | 0.079254038 | -0.341643517 | 0.732619183 | 0.988988648 |
| P36542 | ATP synthase subunit gamma, mitochondrial | ATP5F1C | Homo sapiens | 1.604695252 | 0.118418132 | 0.347063021 | 0.341200661 | 0.732952525 | NA |
| Q7Z6Z7 | E3 ubiquitin-protein ligase HUWE1 | HUWE1 | Homo sapiens | 6.027505555 | 0.063043066 | 0.185153232 | 0.34049131 | 0.733486566 | 0.988988648 |
| Q15637 | Splicing factor 1 | SF1 | Homo sapiens | 4.360698974 | 0.068669601 | 0.202071655 | 0.339827974 | 0.73398608 | 0.988988648 |
| P05455 | Lupus La protein | SSB | Homo sapiens | 10.27390718 | -0.047371497 | 0.139422083 | -0.339770398 | 0.734029442 | 0.988988648 |
| Q8N9H8 | Exonuclease mut-7 homolog | EXD3 | Homo sapiens | 0.838206344 | 0.188280913 | 0.555287232 | 0.339069409 | 0.734557443 | NA |
| O00148 | ATP-dependent RNA helicase DDX39A | DDX39A | Homo sapiens | 14.20641199 | -0.038040596 | 0.112832742 | -0.337141468 | 0.736010258 | 0.988988648 |
| Q9BZZ5 | Apoptosis inhibitor 5 | API5 | Homo sapiens | 3.271721075 | 0.086669717 | 0.258051512 | 0.335862077 | 0.736974876 | 0.988988648 |
| Q9UMY4 | Sorting nexin-12 | SNX12 | Homo sapiens | 0.651479414 | -0.174397972 | 0.519377419 | -0.335782739 | 0.737034707 | NA |
| Q9HBL0 | Tensin-1 | TNS1 | Homo sapiens | 1.879213613 | -0.116210885 | 0.346940725 | -0.334958904 | 0.737656087 | 0.988988648 |
| Q15942 | Zyxin | ZYX | Homo sapiens | 16.4813501 | -0.036121159 | 0.108044568 | -0.334317212 | 0.738140203 | 0.988988648 |
| P11216 | Glycogen phosphorylase, brain form | PYGB | Homo sapiens | 12.2514438 | 0.042464962 | 0.127127729 | 0.334033829 | 0.738354032 | 0.988988648 |
| O00505 | Importin subunit alpha-4 | KPNA3 | Homo sapiens | 3.148467639 | 0.080147532 | 0.240215856 | 0.333647967 | 0.738645218 | 0.988988648 |
| O43390 | Heterogeneous nuclear ribonucleoprotein R | HNRNPR | Homo sapiens | 22.45398093 | 0.030823756 | 0.093117163 | 0.331021216 | 0.74062846 | 0.988988648 |
| P62995 | Transformer-2 protein homolog beta | TRA2B | Homo sapiens | 3.99628858 | -0.071028047 | 0.216462932 | -0.328130299 | 0.742813146 | 0.988988648 |
| P11279 | Lysosome-associated membrane glycoprotein 1 | LAMP1 | Homo sapiens | 9.973038789 | -0.046024698 | 0.140287705 | -0.328073642 | 0.742855982 | 0.988988648 |
| O15260 | Surfeit locus protein 4 | SURF4 | Homo sapiens | 2.181391823 | -0.094699678 | 0.289068186 | -0.327603255 | 0.743211661 | 0.988988648 |
| P23634 | Plasma membrane calcium-transporting ATPase 4 | ATP2B4 | Homo sapiens | 5.872182196 | -0.060591075 | 0.18532178 | -0.326950643 | 0.743705217 | 0.988988648 |
| P09622 | Dihydrolipoyl dehydrogenase, mitochondrial | DLD | Homo sapiens | 7.356371944 | 0.051584367 | 0.157906788 | 0.326676055 | 0.743912913 | 0.988988648 |
| Q9Y2B0 | Protein canopy homolog 2 | CNPY2 | Homo sapiens | 1.793984956 | -0.142988485 | 0.439269584 | -0.325514196 | 0.744791941 | 0.988988648 |
| O14828 | Secretory carrier-associated membrane protein 3 | SCAMP3 | Homo sapiens | 1.858775916 | 0.107965373 | 0.331724968 | 0.325466525 | 0.744828014 | 0.988988648 |
| P11717 | Cation-independent mannose-6-phosphate receptor | IGF2R | Homo sapiens | 1.889511852 | -0.124977429 | 0.385414415 | -0.324267655 | 0.745735405 | 0.988988648 |
| P35659 | Protein DEK | DEK | Homo sapiens | 1.755427957 | 0.108913654 | 0.33641007 | 0.323752657 | 0.746125301 | 0.988988648 |
| O60271 | C-Jun-amino-terminal kinase-interacting protein 4 | SPAG9 | Homo sapiens | 3.500036789 | -0.078857518 | 0.244571562 | -0.322431266 | 0.747126001 | 0.989152734 |
| P34931 | Heat shock 70 kDa protein 1-like | HSPA1L | Homo sapiens | 32.82844842 | 0.027678554 | 0.086285962 | 0.320777018 | 0.748379377 | 0.989306015 |
| P35579 | Myosin-9 | MYH9 | Homo sapiens | 358.4034487 | 0.007851227 | 0.024619693 | 0.318900279 | 0.749802133 | 0.989306015 |
| Q8N1G4 | Leucine-rich repeat-containing protein 47 | LRRC47 | Homo sapiens | 1.459326068 | -0.12885409 | 0.406445124 | -0.317027029 | 0.751223093 | NA |
| Q9Y3I0 | RNA-splicing ligase RtcB homolog | RTCB | Homo sapiens | 7.023511936 | -0.05184788 | 0.163974508 | -0.31619476 | 0.751854685 | 0.989306015 |
| Q13148 | TAR DNA-binding protein 43 | TARDBP | Homo sapiens | 10.95916852 | 0.040776996 | 0.128975012 | 0.316161987 | 0.751879559 | 0.989306015 |
| P31153 | S-adenosylmethionine synthase isoform type-2 | MAT2A | Homo sapiens | 9.986744835 | 0.043579911 | 0.138377495 | 0.314934963 | 0.752811035 | 0.989306015 |
| P49915 | GMP synthase [glutamine-hydrolyzing] | GMPS | Homo sapiens | 2.684040359 | 0.086006135 | 0.274179514 | 0.313685489 | 0.753759923 | 0.989306015 |
| Q14247 | Src substrate cortactin | CTTN | Homo sapiens | 14.37383529 | -0.037601748 | 0.119949874 | -0.313478842 | 0.753916893 | 0.989306015 |
| Q14117 | Dihydropyrimidinase | DPYS | Homo sapiens | 1.194687356 | -0.12826375 | 0.409214203 | -0.313439145 | 0.753947048 | NA |
| P46060 | Ran GTPase-activating protein 1 | RANGAP1 | Homo sapiens | 5.135799766 | 0.060832144 | 0.194333486 | 0.313029655 | 0.754258132 | 0.989306015 |
| P62280 | 40S ribosomal protein S11 | RPS11 | Homo sapiens | 7.212299395 | -0.051115815 | 0.164005306 | -0.311671714 | 0.755290027 | 0.989307126 |
| P23396 | 40S ribosomal protein S3 | RPS3 | Homo sapiens | 36.21054272 | 0.022149086 | 0.071282978 | 0.310720555 | 0.75601307 | 0.989307126 |
| Q13404 | Ubiquitin-conjugating enzyme E2 variant 1 | UBE2V1 | Homo sapiens | 1.188028413 | -0.138689684 | 0.446645432 | -0.310514053 | 0.756170074 | NA |
| P63167 | Dynein light chain 1, cytoplasmic | DYNLL1 | Homo sapiens | 1.21631058 | -0.122259032 | 0.396688788 | -0.30819886 | 0.75793102 | NA |
| Q5JRX3 | Presequence protease, mitochondrial | PITRM1 | Homo sapiens | 3.844339738 | 0.072186824 | 0.234800155 | 0.307439421 | 0.758508927 | 0.990328141 |
| Q92973 | Transportin-1 | TNPO1 | Homo sapiens | 7.27932359 | 0.054746147 | 0.178101997 | 0.307386484 | 0.758549215 | 0.990328141 |
| P02795 | Metallothionein-2 | MT2A | Homo sapiens | 0.689999232 | 0.202379424 | 0.662348845 | 0.305548089 | 0.75994875 | NA |
| P49591 | Serine--tRNA ligase, cytoplasmic | SARS1 | Homo sapiens | 7.035223236 | 0.050882721 | 0.166771719 | 0.305104014 | 0.760286934 | 0.9912737 |
| P14923 | Junction plakoglobin | JUP | Homo sapiens | 1.565246571 | -0.120444833 | 0.39577538 | -0.304326239 | 0.760879357 | NA |
| O75083 | WD repeat-containing protein 1 | WDR1 | Homo sapiens | 52.04131346 | -0.018859918 | 0.062013273 | -0.304127115 | 0.76103105 | 0.9912737 |
| P52294 | Importin subunit alpha-5 | KPNA1 | Homo sapiens | 0.679531989 | 0.155619222 | 0.515792441 | 0.301709 | 0.762873907 | NA |
| P11177 | Pyruvate dehydrogenase E1 component subunit beta, mitochondrial | PDHB | Homo sapiens | 1.147703093 | -0.151476205 | 0.504651587 | -0.300159969 | 0.764055138 | NA |
| P42677 | 40S ribosomal protein S27 | RPS27 | Homo sapiens | 3.525920869 | 0.069789956 | 0.233101195 | 0.299397676 | 0.764636635 | 0.992995366 |
| P10644 | cAMP-dependent protein kinase type I-alpha regulatory subunit | PRKAR1A | Homo sapiens | 3.715644603 | -0.069848118 | 0.234692847 | -0.297615026 | 0.765997005 | 0.992995366 |
| P15531 | Nucleoside diphosphate kinase A | NME1 | Homo sapiens | 19.18411231 | 0.033011499 | 0.111170606 | 0.296944491 | 0.766508888 | 0.992995366 |
| P48163 | NADP-dependent malic enzyme | ME1 | Homo sapiens | 0.922196826 | 0.136642998 | 0.461678151 | 0.29597025 | 0.767252801 | NA |
| P04156 | Major prion protein | PRNP | Homo sapiens | 2.542182593 | -0.082266249 | 0.278436746 | -0.295457586 | 0.767644348 | 0.992995366 |
| P21810 | Biglycan | BGN | Homo sapiens | 2.402088926 | 0.081974638 | 0.280217536 | 0.292539288 | 0.769874326 | 0.992995366 |
| P13987 | CD59 glycoprotein | CD59 | Homo sapiens | 4.025309127 | 0.065124699 | 0.223110537 | 0.291894321 | 0.770367426 | 0.992995366 |
| Q15404 | Ras suppressor protein 1 | RSU1 | Homo sapiens | 4.902992911 | -0.060813231 | 0.209596176 | -0.290144753 | 0.7717055 | 0.992995366 |
| O60664 | Perilipin-3 | PLIN3 | Homo sapiens | 24.78617016 | -0.026403564 | 0.091184323 | -0.289562532 | 0.772150935 | 0.992995366 |
| Q27J81 | Inverted formin-2 | INF2 | Homo sapiens | 10.23790854 | -0.039872019 | 0.138111812 | -0.288693764 | 0.772815735 | 0.992995366 |
| P20339 | Ras-related protein Rab-5A | RAB5A | Homo sapiens | 0.947276289 | 0.139123049 | 0.485004844 | 0.286848783 | 0.774228108 | NA |
| Q7Z589 | BRCA2-interacting transcriptional repressor EMSY | EMSY | Homo sapiens | 0.943132562 | -0.149935085 | 0.522973076 | -0.286697522 | 0.774343935 | NA |
| P35749 | Myosin-11 | MYH11 | Homo sapiens | 47.81485414 | -0.017675952 | 0.061723069 | -0.286375135 | 0.774590817 | 0.992995366 |
| Q13838 | Spliceosome RNA helicase DDX39B | DDX39B | Homo sapiens | 17.16924069 | -0.029320129 | 0.102688578 | -0.285524734 | 0.775242157 | 0.992995366 |
| Q9UL46 | Proteasome activator complex subunit 2 | PSME2 | Homo sapiens | 3.743185361 | -0.068933901 | 0.241647046 | -0.28526689 | 0.775439677 | 0.992995366 |
| P11047 | Laminin subunit gamma-1 | LAMC1 | Homo sapiens | 3.523528225 | -0.067784632 | 0.238924383 | -0.283707468 | 0.776634571 | 0.992995366 |
| P62857 | 40S ribosomal protein S28 | RPS28 | Homo sapiens | 4.017691119 | 0.059302736 | 0.209237678 | 0.283422836 | 0.776852725 | 0.992995366 |
| O00507 | Probable ubiquitin carboxyl-terminal hydrolase FAF-Y | USP9Y | Homo sapiens | 1.3028558 | -0.111004988 | 0.391915984 | -0.283236696 | 0.7769954 | NA |
| Q12792 | Twinfilin-1 | TWF1 | Homo sapiens | 2.584662764 | 0.07543231 | 0.266456518 | 0.283094258 | 0.777104583 | 0.992995366 |
| P17174 | Aspartate aminotransferase, cytoplasmic | GOT1 | Homo sapiens | 3.541713503 | 0.070301173 | 0.248605043 | 0.28278257 | 0.777343516 | 0.992995366 |
| P54652 | Heat shock-related 70 kDa protein 2 | HSPA2 | Homo sapiens | 19.07968837 | 0.029154662 | 0.103418749 | 0.28190886 | 0.778013396 | 0.992995366 |
| Q9Y6B6 | GTP-binding protein SAR1b | SAR1B | Homo sapiens | 1.689161381 | 0.091165843 | 0.323514511 | 0.281798313 | 0.778098165 | NA |
| P13591 | Neural cell adhesion molecule 1 | NCAM1 | Homo sapiens | 1.897024656 | -0.092777754 | 0.329387531 | -0.281667475 | 0.778198496 | 0.992995366 |
| P49585 | Choline-phosphate cytidylyltransferase A | PCYT1A | Homo sapiens | 0.825682566 | 0.145013558 | 0.522995052 | 0.2772752 | 0.781568801 | NA |
| Q8NB16 | Mixed lineage kinase domain-like protein | MLKL | Homo sapiens | 1.062573764 | -0.131198512 | 0.476272053 | -0.275469683 | 0.782955414 | NA |
| O43396 | Thioredoxin-like protein 1 | TXNL1 | Homo sapiens | 7.984557057 | -0.041328716 | 0.150123704 | -0.275297734 | 0.783087506 | 0.997430437 |
| Q9Y305 | Acyl-coenzyme A thioesterase 9, mitochondrial | ACOT9 | Homo sapiens | 2.600422387 | 0.074306061 | 0.270365685 | 0.274835398 | 0.783442701 | 0.997430437 |
| Q10713 | Mitochondrial-processing peptidase subunit alpha | PMPCA | Homo sapiens | 0.645357346 | -0.145570266 | 0.534578147 | -0.272308673 | 0.785384688 | NA |
| Q9Y520 | Protein PRRC2C | PRRC2C | Homo sapiens | 0.997859718 | 0.123357047 | 0.454329299 | 0.271514621 | 0.785995256 | NA |
| P00533 | Epidermal growth factor receptor | EGFR | Homo sapiens | 3.314582153 | 0.073064397 | 0.269526734 | 0.271084043 | 0.786326394 | 0.998182941 |
| Q02388 | Collagen alpha-1(VII) chain | COL7A1 | Homo sapiens | 0.737591353 | -0.147176411 | 0.544315487 | -0.270388065 | 0.786861721 | NA |
| Q14444 | Caprin-1 | CAPRIN1 | Homo sapiens | 8.447831471 | -0.040214933 | 0.148892837 | -0.270093134 | 0.787088604 | 0.998182941 |
| Q13813 | Spectrin alpha chain, non-erythrocytic 1 | SPTAN1 | Homo sapiens | 24.91092809 | -0.025915361 | 0.096347044 | -0.268979304 | 0.787945609 | 0.998182941 |
| Q86UU1 | Pleckstrin homology-like domain family B member 1 | PHLDB1 | Homo sapiens | 0.981137851 | 0.118365146 | 0.440248951 | 0.268859576 | 0.788037745 | NA |
| O75844 | CAAX prenyl protease 1 homolog | ZMPSTE24 | Homo sapiens | 1.092171665 | 0.119795012 | 0.446452747 | 0.268326296 | 0.788448167 | NA |
| P62714 | Serine/threonine-protein phosphatase 2A catalytic subunit beta isoform | PPP2CB | Homo sapiens | 2.351516029 | 0.08633712 | 0.323134589 | 0.267186253 | 0.78932576 | 0.998182941 |
| P40121 | Macrophage-capping protein | CAPG | Homo sapiens | 2.30762579 | 0.07842821 | 0.293549922 | 0.267171626 | 0.789337022 | 0.998182941 |
| P20592 | Interferon-induced GTP-binding protein Mx2 | MX2 | Homo sapiens | 8.637909277 | -0.051146249 | 0.192847496 | -0.265216039 | 0.79084304 | 0.998182941 |
| Q9Y2A7 | Nck-associated protein 1 | NCKAP1 | Homo sapiens | 0.613183684 | -0.175418086 | 0.6632489 | -0.26448304 | 0.791407731 | NA |
| Q9Y5P6 | Mannose-1-phosphate guanyltransferase beta | GMPPB | Homo sapiens | 1.082943018 | 0.108144904 | 0.409372365 | 0.264172458 | 0.791647033 | NA |
| P20336 | Ras-related protein Rab-3A | RAB3A | Homo sapiens | 1.435141373 | 0.099923214 | 0.378582322 | 0.263940517 | 0.791825754 | NA |
| Q9UBQ5 | Eukaryotic translation initiation factor 3 subunit K | EIF3K | Homo sapiens | 1.532602866 | 0.098525102 | 0.373411362 | 0.263851377 | 0.791894444 | NA |
| Q58FF8 | Putative heat shock protein HSP 90-beta 2 | HSP90AB2P | Homo sapiens | 4.524913942 | 0.062093587 | 0.235772351 | 0.263362461 | 0.792271221 | 0.998182941 |
| Q16555 | Dihydropyrimidinase-related protein 2 | DPYSL2 | Homo sapiens | 60.78387397 | -0.015197866 | 0.05782322 | -0.262833265 | 0.792679095 | 0.998182941 |
| Q01995 | Transgelin | TAGLN | Homo sapiens | 15.53169495 | 0.034818992 | 0.13336952 | 0.261071585 | 0.794037303 | 0.998182941 |
| Q9UHY1 | Nuclear receptor-binding protein | NRBP1 | Homo sapiens | 2.858858868 | -0.06749588 | 0.25934667 | -0.260253504 | 0.794668236 | 0.998182941 |
| P61204 | ADP-ribosylation factor 3 | ARF3 | Homo sapiens | 7.204607109 | -0.044347728 | 0.171382183 | -0.258765099 | 0.795816488 | 0.998182941 |
| O96008 | Mitochondrial import receptor subunit TOM40 homolog | TOMM40 | Homo sapiens | 2.151217852 | 0.079101994 | 0.307040191 | 0.25762749 | 0.796694411 | 0.998182941 |
| P30050 | 60S ribosomal protein L12 | RPL12 | Homo sapiens | 28.54761662 | -0.020540953 | 0.079858608 | -0.257216515 | 0.797011636 | 0.998182941 |
| O75352 | Mannose-P-dolichol utilization defect 1 protein | MPDU1 | Homo sapiens | 0.863470612 | 0.116338437 | 0.452478086 | 0.25711397 | 0.797090793 | NA |
| O43313 | ATM interactor | ATMIN | Homo sapiens | 1.272536989 | -0.105710566 | 0.411461671 | -0.256914735 | 0.797244595 | NA |
| P52565 | Rho GDP-dissociation inhibitor 1 | ARHGDIA | Homo sapiens | 13.66701965 | 0.029625735 | 0.117428154 | 0.252288175 | 0.800818327 | 0.998182941 |
| P10321 | HLA class I histocompatibility antigen, C alpha chain | HLA-C | Homo sapiens | 4.971667728 | -0.049879681 | 0.198075793 | -0.251821184 | 0.801179282 | 0.998182941 |
| P08670 | Vimentin | VIM | Homo sapiens | 370.1449285 | 0.018774398 | 0.074761329 | 0.251124448 | 0.801717894 | 0.998182941 |
| Q02930 | Cyclic AMP-responsive element-binding protein 5 | CREB5 | Homo sapiens | 3.083502622 | -0.062351779 | 0.250702085 | -0.248708658 | 0.803586151 | 0.998182941 |
| P20648 | Potassium-transporting ATPase alpha chain 1 | ATP4A | Homo sapiens | 1.460760335 | -0.09339919 | 0.375556674 | -0.248695327 | 0.803596464 | NA |
| Q92597 | Protein NDRG1 | NDRG1 | Homo sapiens | 2.26261663 | -0.07496163 | 0.302287995 | -0.247980837 | 0.804149234 | 0.998182941 |
| P84085 | ADP-ribosylation factor 5 | ARF5 | Homo sapiens | 24.05600368 | -0.021516286 | 0.087923615 | -0.244715666 | 0.806676597 | 0.998182941 |
| Q99439 | Calponin-2 | CNN2 | Homo sapiens | 16.383695 | -0.02602018 | 0.106377499 | -0.244602292 | 0.806764389 | 0.998182941 |
| P62750 | 60S ribosomal protein L23a | RPL23A | Homo sapiens | 7.818365213 | 0.038584469 | 0.158047127 | 0.244132685 | 0.80712806 | 0.998182941 |
| P61916 | NPC intracellular cholesterol transporter 2 | NPC2 | Homo sapiens | 0.872998123 | -0.112132809 | 0.460685368 | -0.243404322 | 0.807692196 | NA |
| Q9H0U4 | Ras-related protein Rab-1B | RAB1B | Homo sapiens | 14.41951823 | -0.02751465 | 0.113222162 | -0.243014702 | 0.807994008 | 0.998182941 |
| Q06033 | Inter-alpha-trypsin inhibitor heavy chain H3 | ITIH3 | Homo sapiens | 2.984906259 | -0.061712261 | 0.25433301 | -0.242643536 | 0.808281552 | 0.998182941 |
| P35222 | Catenin beta-1 | CTNNB1 | Homo sapiens | 3.596524902 | -0.061170196 | 0.253015871 | -0.241764264 | 0.808962831 | 0.998182941 |
| P54709 | Sodium/potassium-transporting ATPase subunit beta-3 | ATP1B3 | Homo sapiens | 2.173113426 | -0.071202569 | 0.297015598 | -0.239726698 | 0.810542136 | 0.998182941 |
| Q5JNZ5 | Putative 40S ribosomal protein S26-like 1 | RPS26P11 | Homo sapiens | 1.779832709 | -0.086831075 | 0.365106549 | -0.237823931 | 0.812017657 | 0.998182941 |
| Q9Y5G6 | Protocadherin gamma-A7 | PCDHGA7 | Homo sapiens | 1.883997037 | -0.080657626 | 0.341276646 | -0.23634089 | 0.81316816 | 0.998182941 |
| Q5VYK3 | Proteasome adapter and scaffold protein ECM29 | ECPAS | Homo sapiens | 1.842714993 | -0.079591554 | 0.339866896 | -0.234184484 | 0.81484176 | 0.998182941 |
| P61586 | Transforming protein RhoA | RHOA | Homo sapiens | 7.536728301 | 0.039174907 | 0.167821476 | 0.233432024 | 0.815425948 | 0.998182941 |
| P13796 | Plastin-2 | LCP1 | Homo sapiens | 1.179220167 | -0.0917444 | 0.39452001 | -0.232546888 | 0.816113274 | NA |
| P07099 | Epoxide hydrolase 1 | EPHX1 | Homo sapiens | 1.570507763 | -0.083966181 | 0.362187181 | -0.231830904 | 0.816669353 | NA |
| P07197 | Neurofilament medium polypeptide | NEFM | Homo sapiens | 7.851854222 | -0.034792548 | 0.150383852 | -0.231358272 | 0.81703648 | 0.998182941 |
| Q9Y262 | Eukaryotic translation initiation factor 3 subunit L | EIF3L | Homo sapiens | 7.570508117 | 0.036340031 | 0.157597279 | 0.230587931 | 0.817634944 | 0.998182941 |
| P20020 | Plasma membrane calcium-transporting ATPase 1 | ATP2B1 | Homo sapiens | 2.828980282 | -0.062815836 | 0.272808622 | -0.23025605 | 0.81789281 | 0.998182941 |
| Q14669 | E3 ubiquitin-protein ligase TRIP12 | TRIP12 | Homo sapiens | 0.894045281 | 0.131165731 | 0.570434695 | 0.22993996 | 0.818138425 | NA |
| P41091 | Eukaryotic translation initiation factor 2 subunit 3 | EIF2S3 | Homo sapiens | 14.91636363 | 0.025046447 | 0.10962876 | 0.228466025 | 0.819283966 | 0.998182941 |
| P22087 | rRNA 2'-O-methyltransferase fibrillarin | FBL | Homo sapiens | 3.321078979 | -0.054132699 | 0.238832018 | -0.226655956 | 0.820691278 | 0.998182941 |
| P46781 | 40S ribosomal protein S9 | RPS9 | Homo sapiens | 14.7154962 | 0.024576414 | 0.110083369 | 0.223252746 | 0.823338802 | 0.998182941 |
| P28482 | Mitogen-activated protein kinase 1 | MAPK1 | Homo sapiens | 0.657902635 | -0.13155428 | 0.593590131 | -0.221624777 | 0.824605993 | NA |
| Q7Z460 | CLIP-associating protein 1 | CLASP1 | Homo sapiens | 0.906219081 | 0.116499354 | 0.525850473 | 0.22154464 | 0.824668382 | NA |
| Q9Y2V2 | Calcium-regulated heat-stable protein 1 | CARHSP1 | Homo sapiens | 1.667171529 | 0.069944299 | 0.315772489 | 0.221502195 | 0.824701428 | NA |
| O75947 | ATP synthase subunit d, mitochondrial | ATP5PD | Homo sapiens | 0.753259943 | -0.115426435 | 0.521235616 | -0.221447713 | 0.824743845 | NA |
| P21281 | V-type proton ATPase subunit B, brain isoform | ATP6V1B2 | Homo sapiens | 6.95741492 | -0.036383406 | 0.164397673 | -0.221313387 | 0.824848427 | 0.998182941 |
| Q13347 | Eukaryotic translation initiation factor 3 subunit I | EIF3I | Homo sapiens | 4.866354376 | -0.045223347 | 0.204935058 | -0.2206716 | 0.825348146 | 0.998182941 |
| P22234 | Multifunctional protein ADE2 | PAICS | Homo sapiens | 11.8266673 | -0.027355457 | 0.124021402 | -0.220570454 | 0.825426909 | 0.998182941 |
| P25786 | Proteasome subunit alpha type-1 | PSMA1 | Homo sapiens | 1.346184518 | -0.122608694 | 0.560893068 | -0.218595488 | 0.826965168 | NA |
| P07942 | Laminin subunit beta-1 | LAMB1 | Homo sapiens | 2.318245676 | 0.064975417 | 0.297982888 | 0.218050833 | 0.827389505 | 0.998182941 |
| P06748 | Nucleophosmin | NPM1 | Homo sapiens | 61.59551516 | 0.012007005 | 0.055128235 | 0.217801374 | 0.827583874 | 0.998182941 |
| P35606 | Coatomer subunit beta' | COPB2 | Homo sapiens | 24.72546071 | -0.018986394 | 0.087232876 | -0.217651821 | 0.827700405 | 0.998182941 |
| Q9UMR2 | ATP-dependent RNA helicase DDX19B | DDX19B | Homo sapiens | 0.633975438 | -0.107496737 | 0.495301242 | -0.217033046 | 0.828182592 | NA |
| P23246 | Splicing factor, proline- and glutamine-rich | SFPQ | Homo sapiens | 18.49082271 | 0.021235641 | 0.099571727 | 0.213269788 | 0.831116537 | 0.998182941 |
| P53992 | Protein transport protein Sec24C | SEC24C | Homo sapiens | 9.529219152 | 0.030517012 | 0.144690744 | 0.210911987 | 0.832955951 | 0.998182941 |
| Q16401 | 26S proteasome non-ATPase regulatory subunit 5 | PSMD5 | Homo sapiens | 2.151930262 | 0.06415389 | 0.305003573 | 0.210338158 | 0.833403755 | 0.998182941 |
| Q96AE4 | Far upstream element-binding protein 1 | FUBP1 | Homo sapiens | 11.60899418 | -0.026856639 | 0.128109043 | -0.2096389 | 0.833949516 | 0.998182941 |
| Q16186 | Proteasomal ubiquitin receptor ADRM1 | ADRM1 | Homo sapiens | 0.978813239 | 0.097900294 | 0.468265356 | 0.20907012 | 0.8343935 | NA |
| P35268 | 60S ribosomal protein L22 | RPL22 | Homo sapiens | 16.10889682 | 0.022061592 | 0.106499146 | 0.20715276 | 0.835890559 | 0.998182941 |
| P20742 | Pregnancy zone protein | PZP | Homo sapiens | 4.460042914 | -0.041957358 | 0.203553573 | -0.2061244 | 0.836693739 | 0.998182941 |
| P07814 | Bifunctional glutamate/proline--tRNA ligase | EPRS1 | Homo sapiens | 19.42608452 | -0.020793541 | 0.101980338 | -0.203897548 | 0.838433561 | 0.998182941 |
| P67870 | Casein kinase II subunit beta | CSNK2B | Homo sapiens | 0.65428785 | 0.108241071 | 0.53302771 | 0.203068375 | 0.83908159 | NA |
| Q9UGP8 | Translocation protein SEC63 homolog | SEC63 | Homo sapiens | 2.305870262 | -0.056668868 | 0.27918451 | -0.202979985 | 0.839150676 | 0.998182941 |
| P62269 | 40S ribosomal protein S18 | RPS18 | Homo sapiens | 7.401809626 | 0.031748411 | 0.15662892 | 0.202698267 | 0.839370878 | 0.998182941 |
| Q9BRR6 | ADP-dependent glucokinase | ADPGK | Homo sapiens | 0.575794536 | -0.128404097 | 0.634162997 | -0.202478067 | 0.839543004 | NA |
| P09486 | SPARC | SPARC | Homo sapiens | 6.000089649 | 0.042014277 | 0.208241571 | 0.201757396 | 0.84010639 | 0.998182941 |
| P68036 | Ubiquitin-conjugating enzyme E2 L3 | UBE2L3 | Homo sapiens | 12.18653838 | 0.024701617 | 0.12304377 | 0.200754715 | 0.840890374 | 0.998182941 |
| Q13155 | Aminoacyl tRNA synthase complex-interacting multifunctional protein 2 | AIMP2 | Homo sapiens | 1.5260552 | 0.073782476 | 0.36990403 | 0.199463835 | 0.841899931 | NA |
| P62244 | 40S ribosomal protein S15a | RPS15A | Homo sapiens | 5.938863956 | 0.034481055 | 0.17408318 | 0.198072293 | 0.842988502 | 0.998182941 |
| P63241 | Eukaryotic translation initiation factor 5A-1 | EIF5A | Homo sapiens | 52.4399564 | -0.01210236 | 0.061128254 | -0.197983084 | 0.843058299 | 0.998182941 |
| P55786 | Puromycin-sensitive aminopeptidase | NPEPPS | Homo sapiens | 11.22961825 | 0.025814228 | 0.13132869 | 0.196561987 | 0.84417032 | 0.998182941 |
| O14786 | Neuropilin-1 | NRP1 | Homo sapiens | 2.583333282 | -0.056112871 | 0.288398504 | -0.194567136 | 0.845731833 | 0.998182941 |
| Q15075 | Early endosome antigen 1 | EEA1 | Homo sapiens | 1.691645953 | 0.067618813 | 0.35262891 | 0.191756294 | 0.847933108 | NA |
| Q96QD8 | Sodium-coupled neutral amino acid transporter 2 | SLC38A2 | Homo sapiens | 9.22649325 | 0.028565427 | 0.148987617 | 0.191730208 | 0.847953543 | 0.998182941 |
| Q15477 | Helicase SKI2W | SKIV2L | Homo sapiens | 0.910532291 | 0.098317781 | 0.516599019 | 0.190317398 | 0.849060421 | NA |
| Q01130 | Serine/arginine-rich splicing factor 2 | SRSF2 | Homo sapiens | 2.817617063 | 0.047047123 | 0.251536027 | 0.187039304 | 0.851629817 | 0.998182941 |
| P60891 | Ribose-phosphate pyrophosphokinase 1 | PRPS1 | Homo sapiens | 3.726838689 | 0.041246905 | 0.220841345 | 0.186771663 | 0.851839666 | 0.998182941 |
| O95741 | Copine-6 | CPNE6 | Homo sapiens | 0.404496781 | 0.135909947 | 0.734573421 | 0.185018873 | 0.853214232 | NA |
| P12955 | Xaa-Pro dipeptidase | PEPD | Homo sapiens | 0.769102042 | -0.089817798 | 0.486182893 | -0.184740762 | 0.853432373 | NA |
| P54136 | Arginine--tRNA ligase, cytoplasmic | RARS1 | Homo sapiens | 19.05968965 | 0.018248142 | 0.099139341 | 0.184065599 | 0.853961994 | 0.998182941 |
| P07741 | Adenine phosphoribosyltransferase | APRT | Homo sapiens | 1.561038289 | -0.065269176 | 0.354966341 | -0.183874269 | 0.854112091 | NA |
| P38646 | Stress-70 protein, mitochondrial | HSPA9 | Homo sapiens | 61.78549403 | 0.01005451 | 0.054766837 | 0.183587564 | 0.85433702 | 0.998182941 |
| P11586 | C-1-tetrahydrofolate synthase, cytoplasmic | MTHFD1 | Homo sapiens | 8.900391625 | -0.026923381 | 0.147192336 | -0.18291293 | 0.854866337 | 0.998182941 |
| Q86VP6 | Cullin-associated NEDD8-dissociated protein 1 | CAND1 | Homo sapiens | 22.66872867 | 0.016228917 | 0.08986428 | 0.180593629 | 0.856686557 | 0.998182941 |
| P61163 | Alpha-centractin | ACTR1A | Homo sapiens | 8.040344242 | -0.031850826 | 0.176605869 | -0.18034976 | 0.856877993 | 0.998182941 |
| P53675 | Clathrin heavy chain 2 | CLTCL1 | Homo sapiens | 35.75598 | 0.013214302 | 0.073283283 | 0.180318098 | 0.856902849 | 0.998182941 |
| P47897 | Glutamine--tRNA ligase | QARS1 | Homo sapiens | 8.662781111 | -0.027418375 | 0.152070417 | -0.180300515 | 0.856916651 | 0.998182941 |
| P20337 | Ras-related protein Rab-3B | RAB3B | Homo sapiens | 6.258267016 | -0.031968218 | 0.178304332 | -0.179290191 | 0.857709849 | 0.998182941 |
| P10515 | Dihydrolipoyllysine-residue acetyltransferase component of pyruvate dehydrogenase complex, mitochondrial | DLAT | Homo sapiens | 1.187052031 | -0.076247872 | 0.42535996 | -0.179254935 | 0.85773753 | NA |
| Q13492 | Phosphatidylinositol-binding clathrin assembly protein | PICALM | Homo sapiens | 6.598962679 | -0.031573881 | 0.176268736 | -0.179123548 | 0.857840692 | 0.998182941 |
| Q02978 | Mitochondrial 2-oxoglutarate/malate carrier protein | SLC25A11 | Homo sapiens | 0.711391402 | -0.08657125 | 0.484557549 | -0.178660408 | 0.858204358 | NA |
| P42765 | 3-ketoacyl-CoA thiolase, mitochondrial | ACAA2 | Homo sapiens | 4.030253862 | 0.039893904 | 0.227030107 | 0.175720763 | 0.860513323 | 0.998182941 |
| Q02878 | 60S ribosomal protein L6 | RPL6 | Homo sapiens | 32.18484083 | 0.013751014 | 0.078782426 | 0.174544177 | 0.861437815 | 0.998182941 |
| Q9UHD8 | Septin-9 | SEPTIN9 | Homo sapiens | 16.01168078 | -0.0194736 | 0.111887377 | -0.174046445 | 0.861828961 | 0.998182941 |
| Q15365 | Poly(rC)-binding protein 1 | PCBP1 | Homo sapiens | 34.2719547 | -0.012805997 | 0.073796926 | -0.173530217 | 0.862234679 | 0.998182941 |
| O95782 | AP-2 complex subunit alpha-1 | AP2A1 | Homo sapiens | 7.31779065 | -0.02746047 | 0.159587001 | -0.172072096 | 0.863380849 | 0.998182941 |
| Q53GS9 | U4/U6.U5 tri-snRNP-associated protein 2 | USP39 | Homo sapiens | 0.987312482 | -0.075769544 | 0.441272025 | -0.17170711 | 0.863667796 | NA |
| P50454 | Serpin H1 | SERPINH1 | Homo sapiens | 69.78248733 | 0.009332484 | 0.054803001 | 0.170291478 | 0.864780913 | 0.998182941 |
| Q6DD88 | Atlastin-3 | ATL3 | Homo sapiens | 9.3643462 | 0.02413506 | 0.141871832 | 0.170118761 | 0.86491674 | 0.998182941 |
| P39023 | 60S ribosomal protein L3 | RPL3 | Homo sapiens | 26.43701218 | 0.014215021 | 0.083691788 | 0.16984965 | 0.865128379 | 0.998182941 |
| P36776 | Lon protease homolog, mitochondrial | LONP1 | Homo sapiens | 1.358411892 | 0.066718269 | 0.394963752 | 0.168922513 | 0.865857591 | NA |
| P26232 | Catenin alpha-2 | CTNNA2 | Homo sapiens | 4.630046837 | 0.03436852 | 0.203687905 | 0.168731277 | 0.866008016 | 0.998182941 |
| P10155 | 60 kDa SS-A/Ro ribonucleoprotein | RO60 | Homo sapiens | 1.610353581 | -0.058009821 | 0.344841074 | -0.1682219 | 0.866408713 | NA |
| Q9Y2X3 | Nucleolar protein 58 | NOP58 | Homo sapiens | 1.857911991 | 0.052903033 | 0.318675528 | 0.166009085 | 0.868149801 | 0.998182941 |
| Q15032 | R3H domain-containing protein 1 | R3HDM1 | Homo sapiens | 1.08868692 | 0.068836136 | 0.414789897 | 0.165954225 | 0.868192974 | NA |
| P54707 | Potassium-transporting ATPase alpha chain 2 | ATP12A | Homo sapiens | 0.974531796 | 0.077018575 | 0.466182639 | 0.165211162 | 0.86877778 | NA |
| Q96QR8 | Transcriptional activator protein Pur-beta | PURB | Homo sapiens | 0.998340195 | -0.071632964 | 0.437708683 | -0.163654428 | 0.870003195 | NA |
| Q9BVK6 | Transmembrane emp24 domain-containing protein 9 | TMED9 | Homo sapiens | 4.479740183 | -0.033600022 | 0.206204576 | -0.162945086 | 0.870561672 | 0.998182941 |
| P25325 | 3-mercaptopyruvate sulfurtransferase | MPST | Homo sapiens | 1.551251147 | 0.057160473 | 0.351301038 | 0.162710801 | 0.870746142 | NA |
| Q4VXU2 | Polyadenylate-binding protein 1-like | PABPC1L | Homo sapiens | 1.445068302 | 0.061328754 | 0.38181278 | 0.1606252 | 0.872388606 | NA |
| Q96IJ6 | Mannose-1-phosphate guanyltransferase alpha | GMPPA | Homo sapiens | 1.805802115 | -0.052117124 | 0.329634076 | -0.158105995 | 0.874373277 | 0.998182941 |
| Q07021 | Complement component 1 Q subcomponent-binding protein, mitochondrial | C1QBP | Homo sapiens | 9.270255051 | 0.022932848 | 0.146477998 | 0.156561725 | 0.87559027 | 0.998182941 |
| P50402 | Emerin | EMD | Homo sapiens | 0.617929541 | 0.080187445 | 0.515863542 | 0.155443133 | 0.876471982 | NA |
| P38159 | RNA-binding motif protein, X chromosome | RBMX | Homo sapiens | 4.094428579 | -0.03364368 | 0.217667648 | -0.15456445 | 0.877164698 | 0.998182941 |
| Q8IYJ1 | Copine-9 | CPNE9 | Homo sapiens | 0.405018129 | 0.116324992 | 0.757697764 | 0.153524264 | 0.877984857 | NA |
| P14314 | Glucosidase 2 subunit beta | PRKCSH | Homo sapiens | 20.02938763 | 0.015287385 | 0.100406312 | 0.152255218 | 0.878985644 | 0.998182941 |
| P84103 | Serine/arginine-rich splicing factor 3 | SRSF3 | Homo sapiens | 8.869644532 | -0.021629532 | 0.142611752 | -0.15166725 | 0.879449389 | 0.998182941 |
| P31942 | Heterogeneous nuclear ribonucleoprotein H3 | HNRNPH3 | Homo sapiens | 8.006694235 | 0.023718883 | 0.156918429 | 0.151154223 | 0.87985406 | 0.998182941 |
| P13164 | Interferon-induced transmembrane protein 1 | IFITM1 | Homo sapiens | 0.787600947 | 0.075113831 | 0.497055771 | 0.151117511 | 0.87988302 | NA |
| O75494 | Serine/arginine-rich splicing factor 10 | SRSF10 | Homo sapiens | 1.619252423 | -0.051892439 | 0.346897291 | -0.149590212 | 0.881087931 | NA |
| Q6IBS0 | Twinfilin-2 | TWF2 | Homo sapiens | 3.6011016 | -0.03533931 | 0.23675684 | -0.149264159 | 0.881345196 | 0.998182941 |
| P62913 | 60S ribosomal protein L11 | RPL11 | Homo sapiens | 8.651632498 | 0.021499695 | 0.145262669 | 0.148005643 | 0.882338315 | 0.998182941 |
| Q92896 | Golgi apparatus protein 1 | GLG1 | Homo sapiens | 1.774507229 | -0.05316682 | 0.363051923 | -0.146444121 | 0.883570799 | 0.998182941 |
| Q05193 | Dynamin-1 | DNM1 | Homo sapiens | 3.2137502 | 0.034804414 | 0.240831355 | 0.144517783 | 0.885091615 | 0.998182941 |
| P17096 | High mobility group protein HMG-I/HMG-Y | HMGA1 | Homo sapiens | 9.4424109 | -0.020354052 | 0.141150982 | -0.14420057 | 0.885342091 | 0.998182941 |
| Q13247 | Serine/arginine-rich splicing factor 6 | SRSF6 | Homo sapiens | 2.13097247 | 0.049397488 | 0.345372137 | 0.143026849 | 0.886268977 | 0.998182941 |
| O94826 | Mitochondrial import receptor subunit TOM70 | TOMM70 | Homo sapiens | 2.492758357 | -0.040187935 | 0.281280163 | -0.142875111 | 0.886388816 | 0.998182941 |
| P07737 | Profilin-1 | PFN1 | Homo sapiens | 61.45524835 | 0.00810422 | 0.057422549 | 0.141133051 | 0.887764834 | 0.998182941 |
| O60488 | Long-chain-fatty-acid--CoA ligase 4 | ACSL4 | Homo sapiens | 3.700833219 | 0.032916214 | 0.233461904 | 0.1409918 | 0.88787642 | 0.998182941 |
| Q9Y5K5 | Ubiquitin carboxyl-terminal hydrolase isozyme L5 | UCHL5 | Homo sapiens | 1.02661869 | -0.05944195 | 0.421788214 | -0.140928428 | 0.887926484 | NA |
| Q02790 | Peptidyl-prolyl cis-trans isomerase FKBP4 | FKBP4 | Homo sapiens | 4.796794764 | 0.030002994 | 0.214214776 | 0.140060338 | 0.888612317 | 0.998182941 |
| O94905 | Erlin-2 | ERLIN2 | Homo sapiens | 5.728998493 | -0.026462971 | 0.189220148 | -0.139852818 | 0.88877628 | 0.998182941 |
| Q9Y263 | Phospholipase A-2-activating protein | PLAA | Homo sapiens | 0.892197261 | 0.065600981 | 0.469095492 | 0.139845687 | 0.888781915 | NA |
| P11940 | Polyadenylate-binding protein 1 | PABPC1 | Homo sapiens | 40.1391994 | 0.009317575 | 0.066665214 | 0.139766671 | 0.888844347 | 0.998182941 |
| P22455 | Fibroblast growth factor receptor 4 | FGFR4 | Homo sapiens | 0.857080316 | 0.066071367 | 0.472771752 | 0.139753203 | 0.888854989 | NA |
| P78371 | T-complex protein 1 subunit beta | CCT2 | Homo sapiens | 30.45041069 | -0.010839574 | 0.078201214 | -0.138611328 | 0.889757291 | 0.998182941 |
| P62979 | Ubiquitin-40S ribosomal protein S27a | RPS27A | Homo sapiens | 11.92438401 | 0.018298585 | 0.132014643 | 0.138610267 | 0.88975813 | 0.998182941 |
| P26599 | Polypyrimidine tract-binding protein 1 | PTBP1 | Homo sapiens | 33.85213206 | -0.010113226 | 0.073379975 | -0.137819965 | 0.890382705 | 0.998182941 |
| O75390 | Citrate synthase, mitochondrial | CS | Homo sapiens | 6.122123434 | -0.023450733 | 0.172141992 | -0.136229011 | 0.891640241 | 0.998182941 |
| P00491 | Purine nucleoside phosphorylase | PNP | Homo sapiens | 6.509271541 | -0.026110054 | 0.193091581 | -0.135221087 | 0.892437075 | 0.998182941 |
| O00571 | ATP-dependent RNA helicase DDX3X | DDX3X | Homo sapiens | 25.04083423 | 0.01140681 | 0.08566585 | 0.133154692 | 0.894071042 | 0.998182941 |
| P13667 | Protein disulfide-isomerase A4 | PDIA4 | Homo sapiens | 27.32787486 | -0.012165714 | 0.091774861 | -0.132560419 | 0.894541037 | 0.998182941 |
| Q9Y5M8 | Signal recognition particle receptor subunit beta | SRPRB | Homo sapiens | 2.945751455 | 0.032878437 | 0.250724622 | 0.131133657 | 0.895669577 | 0.998182941 |
| Q9BQE5 | Apolipoprotein L2 | APOL2 | Homo sapiens | 0.522857789 | -0.092487639 | 0.716900848 | -0.129010363 | 0.897349449 | NA |
| O75533 | Splicing factor 3B subunit 1 | SF3B1 | Homo sapiens | 8.085520542 | 0.020115555 | 0.156475547 | 0.128553987 | 0.897710577 | 0.998182941 |
| P40394 | All-trans-retinol dehydrogenase [NAD(+)] ADH7 | ADH7 | Homo sapiens | 0.949619498 | -0.071027468 | 0.561153648 | -0.126574011 | 0.899277567 | NA |
| P52788 | Spermine synthase | SMS | Homo sapiens | 1.336679282 | 0.049689183 | 0.39527662 | 0.125707367 | 0.89996357 | NA |
| Q00341 | Vigilin | HDLBP | Homo sapiens | 32.1844096 | -0.00970183 | 0.077963982 | -0.124439905 | 0.900966979 | 0.998182941 |
| Q15121 | Astrocytic phosphoprotein PEA-15 | PEA15 | Homo sapiens | 8.644958443 | 0.018599523 | 0.150257858 | 0.123784028 | 0.901486278 | 0.998182941 |
| Q9UNF1 | Melanoma-associated antigen D2 | MAGED2 | Homo sapiens | 1.94127273 | 0.040294884 | 0.325717003 | 0.123711331 | 0.90154384 | 0.998182941 |
| Q9NVI7 | ATPase family AAA domain-containing protein 3A | ATAD3A | Homo sapiens | 2.89128615 | 0.03356441 | 0.271661402 | 0.12355237 | 0.901669707 | 0.998182941 |
| P68431 | Histone H3.1 | H3C1 | Homo sapiens | 12.10164482 | -0.022767669 | 0.184741187 | -0.123240893 | 0.901916345 | 0.998182941 |
| Q96M27 | Protein PRRC1 | PRRC1 | Homo sapiens | 0.614813956 | -0.068801255 | 0.561206817 | -0.122595187 | 0.902427666 | NA |
| Q9NZL9 | Methionine adenosyltransferase 2 subunit beta | MAT2B | Homo sapiens | 0.640895297 | 0.060566388 | 0.497290143 | 0.121792858 | 0.90306307 | NA |
| P47914 | 60S ribosomal protein L29 | RPL29 | Homo sapiens | 5.571012806 | -0.022687815 | 0.186591604 | -0.121590759 | 0.903223133 | 0.998182941 |
| Q9P2R3 | Rabankyrin-5 | ANKFY1 | Homo sapiens | 0.743748004 | 0.061093436 | 0.502743791 | 0.121520021 | 0.903279158 | NA |
| P40925 | Malate dehydrogenase, cytoplasmic | MDH1 | Homo sapiens | 6.173220157 | -0.020958122 | 0.173019809 | -0.121131342 | 0.903587004 | 0.998182941 |
| Q14008 | Cytoskeleton-associated protein 5 | CKAP5 | Homo sapiens | 1.674076432 | -0.042737565 | 0.356191489 | -0.1199848 | 0.904495189 | NA |
| Q9H8Y8 | Golgi reassembly-stacking protein 2 | GORASP2 | Homo sapiens | 2.603345366 | -0.034181791 | 0.285799909 | -0.119600427 | 0.904799681 | 0.998182941 |
| Q12765 | Secernin-1 | SCRN1 | Homo sapiens | 6.254844765 | 0.020986981 | 0.177776892 | 0.118052357 | 0.906026173 | 0.998182941 |
| P49792 | E3 SUMO-protein ligase RanBP2 | RANBP2 | Homo sapiens | 2.700837158 | -0.033036745 | 0.281819207 | -0.117226733 | 0.906680383 | 0.998182941 |
| Q5SW79 | Centrosomal protein of 170 kDa | CEP170 | Homo sapiens | 3.217986272 | 0.032049888 | 0.275067854 | 0.116516298 | 0.90724337 | 0.998182941 |
| P17661 | Desmin | DES | Homo sapiens | 4.086594376 | -0.028123212 | 0.243998301 | -0.115259868 | 0.908239147 | 0.998182941 |
| Q9Y678 | Coatomer subunit gamma-1 | COPG1 | Homo sapiens | 38.13281443 | 0.008375746 | 0.07295522 | 0.11480667 | 0.908598362 | 0.998182941 |
| Q9BSJ8 | Extended synaptotagmin-1 | ESYT1 | Homo sapiens | 16.79196468 | -0.012289955 | 0.108045391 | -0.113748069 | 0.909437506 | 0.998182941 |
| Q16563 | Synaptophysin-like protein 1 | SYPL1 | Homo sapiens | 0.623917796 | 0.06646281 | 0.592566099 | 0.112161006 | 0.910695746 | NA |
| Q8WWI1 | LIM domain only protein 7 | LMO7 | Homo sapiens | 2.969315732 | 0.030983994 | 0.276279316 | 0.112147354 | 0.910706571 | 0.998182941 |
| Q9UNL2 | Translocon-associated protein subunit gamma | SSR3 | Homo sapiens | 1.932992326 | -0.035034503 | 0.313735074 | -0.111669068 | 0.911085806 | 0.998182941 |
| O43294 | Transforming growth factor beta-1-induced transcript 1 protein | TGFB1I1 | Homo sapiens | 5.442622128 | -0.020669538 | 0.186234753 | -0.110986472 | 0.911627074 | 0.998182941 |
| P25788 | Proteasome subunit alpha type-3 | PSMA3 | Homo sapiens | 0.884890046 | -0.048965329 | 0.446491217 | -0.109666949 | 0.912673513 | NA |
| P61158 | Actin-related protein 3 | ACTR3 | Homo sapiens | 23.92012635 | 0.010047156 | 0.092679246 | 0.108407831 | 0.913672189 | 0.998182941 |
| O60506 | Heterogeneous nuclear ribonucleoprotein Q | SYNCRIP | Homo sapiens | 45.07350508 | -0.00687564 | 0.064702266 | -0.106265838 | 0.915371435 | 0.998182941 |
| P60033 | CD81 antigen | CD81 | Homo sapiens | 1.947276309 | -0.034864521 | 0.328572828 | -0.10610896 | 0.915495902 | 0.998182941 |
| Q9NVA2 | Septin-11 | SEPTIN11 | Homo sapiens | 20.61538216 | -0.009963306 | 0.093919727 | -0.106083212 | 0.915516331 | 0.998182941 |
| P21926 | CD9 antigen | CD9 | Homo sapiens | 0.674004686 | 0.065298195 | 0.615590673 | 0.106074048 | 0.915523602 | NA |
| Q86YS6 | Ras-related protein Rab-43 | RAB43 | Homo sapiens | 1.159035122 | -0.042513465 | 0.400886003 | -0.106048765 | 0.915543661 | NA |
| P15880 | 40S ribosomal protein S2 | RPS2 | Homo sapiens | 29.98323211 | 0.008240699 | 0.078025629 | 0.10561528 | 0.915887601 | 0.998182941 |
| P62241 | 40S ribosomal protein S8 | RPS8 | Homo sapiens | 31.89700005 | 0.007920958 | 0.075063523 | 0.105523404 | 0.9159605 | 0.998182941 |
| Q15029 | 116 kDa U5 small nuclear ribonucleoprotein component | EFTUD2 | Homo sapiens | 8.431201121 | 0.015624565 | 0.148852745 | 0.104966588 | 0.916402321 | 0.998182941 |
| Q92616 | eIF-2-alpha kinase activator GCN1 | GCN1 | Homo sapiens | 24.13283347 | -0.009441389 | 0.090929724 | -0.10383171 | 0.917302902 | 0.998182941 |
| Q08043 | Alpha-actinin-3 | ACTN3 | Homo sapiens | 4.307462177 | -0.023476978 | 0.226447602 | -0.103675101 | 0.917427187 | 0.998182941 |
| P26196 | Probable ATP-dependent RNA helicase DDX6 | DDX6 | Homo sapiens | 3.679351714 | 0.023646714 | 0.228725317 | 0.103384769 | 0.9176576 | 0.998182941 |
| Q8IVL6 | Prolyl 3-hydroxylase 3 | P3H3 | Homo sapiens | 1.728136308 | -0.042086815 | 0.41029183 | -0.102577755 | 0.918298098 | 0.998182941 |
| P46783 | 40S ribosomal protein S10 | RPS10 | Homo sapiens | 23.50765274 | -0.008987957 | 0.08766143 | -0.102530349 | 0.918335725 | 0.998182941 |
| Q9Y383 | Putative RNA-binding protein Luc7-like 2 | LUC7L2 | Homo sapiens | 1.951230364 | -0.03220528 | 0.320049929 | -0.100625799 | 0.919847516 | 0.998182941 |
| P06737 | Glycogen phosphorylase, liver form | PYGL | Homo sapiens | 2.246076243 | 0.029083447 | 0.29081285 | 0.100007434 | 0.920338424 | 0.998182941 |
| P62701 | 40S ribosomal protein S4, X isoform | RPS4X | Homo sapiens | 21.94902559 | 0.009113887 | 0.091434291 | 0.099676909 | 0.920600833 | 0.998182941 |
| P29558 | RNA-binding motif, single-stranded-interacting protein 1 | RBMS1 | Homo sapiens | 1.427601203 | -0.035735245 | 0.365391571 | -0.09779986 | 0.922091218 | NA |
| Q9UNM6 | 26S proteasome non-ATPase regulatory subunit 13 | PSMD13 | Homo sapiens | 2.83380525 | 0.024872735 | 0.254721938 | 0.097646615 | 0.922212908 | 0.998182941 |
| P07951 | Tropomyosin beta chain | TPM2 | Homo sapiens | 1.440721652 | -0.040927682 | 0.427800724 | -0.09566997 | 0.923782692 | NA |
| Q92575 | UBX domain-containing protein 4 | UBXN4 | Homo sapiens | 1.407467071 | -0.034484214 | 0.360500387 | -0.095656524 | 0.923793371 | NA |
| Q93084 | Sarcoplasmic/endoplasmic reticulum calcium ATPase 3 | ATP2A3 | Homo sapiens | 3.07204931 | 0.02539082 | 0.265814593 | 0.09552079 | 0.923901178 | 0.998182941 |
| Q04323 | UBX domain-containing protein 1 | UBXN1 | Homo sapiens | 0.653393453 | 0.051759917 | 0.542535112 | 0.09540381 | 0.92399409 | NA |
| P46087 | Probable 28S rRNA (cytosine(4447)-C(5))-methyltransferase | NOP2 | Homo sapiens | 1.19139299 | 0.039489999 | 0.418639258 | 0.094329423 | 0.924847478 | NA |
| P55084 | Trifunctional enzyme subunit beta, mitochondrial | HADHB | Homo sapiens | 2.752034422 | -0.02538114 | 0.271486403 | -0.093489544 | 0.925514656 | 0.998182941 |
| Q9UJZ1 | Stomatin-like protein 2, mitochondrial | STOML2 | Homo sapiens | 1.444411675 | 0.035208168 | 0.377322826 | 0.093310464 | 0.92565692 | NA |
| P49756 | RNA-binding protein 25 | RBM25 | Homo sapiens | 2.271619164 | 0.026264289 | 0.283287495 | 0.092712491 | 0.926131973 | 0.998182941 |
| P48643 | T-complex protein 1 subunit epsilon | CCT5 | Homo sapiens | 14.25096216 | -0.010246125 | 0.112849507 | -0.090794592 | 0.927655807 | 0.998182941 |
| Q8WX93 | Palladin | PALLD | Homo sapiens | 7.918642719 | -0.015529562 | 0.172410924 | -0.090072957 | 0.928229239 | 0.998182941 |
| Q6EMK4 | Vasorin | VASN | Homo sapiens | 1.963650666 | 0.028599129 | 0.31755891 | 0.090059286 | 0.928240103 | 0.998182941 |
| Q03001 | Dystonin | DST | Homo sapiens | 0.892476199 | 0.043965083 | 0.488534679 | 0.089993782 | 0.928292156 | NA |
| P60228 | Eukaryotic translation initiation factor 3 subunit E | EIF3E | Homo sapiens | 2.449352831 | -0.025698142 | 0.292857974 | -0.087749503 | 0.930075774 | 0.998182941 |
| P99999 | Cytochrome c | CYCS | Homo sapiens | 0.999982265 | -0.035627575 | 0.407461766 | -0.087437837 | 0.930323495 | NA |
| Q00610 | Clathrin heavy chain 1 | CLTC | Homo sapiens | 173.8168354 | 0.003375727 | 0.038748686 | 0.087118487 | 0.930577331 | 0.998182941 |
| Q96RQ1 | Endoplasmic reticulum-Golgi intermediate compartment protein 2 | ERGIC2 | Homo sapiens | 0.520736208 | 0.048729464 | 0.563671698 | 0.086450082 | 0.931108636 | NA |
| Q12841 | Follistatin-related protein 1 | FSTL1 | Homo sapiens | 1.407258569 | -0.035380633 | 0.416808761 | -0.084884571 | 0.932353158 | NA |
| P09104 | Gamma-enolase | ENO2 | Homo sapiens | 47.2253976 | -0.006046744 | 0.071357209 | -0.084739081 | 0.932468826 | 0.998182941 |
| P42892 | Endothelin-converting enzyme 1 | ECE1 | Homo sapiens | 1.4562136 | 0.030586703 | 0.361423031 | 0.08462854 | 0.932556709 | NA |
| Q01469 | Fatty acid-binding protein 5 | FABP5 | Homo sapiens | 3.919759338 | 0.018952888 | 0.230806137 | 0.08211605 | 0.93455443 | 0.998182941 |
| P50552 | Vasodilator-stimulated phosphoprotein | VASP | Homo sapiens | 3.419116487 | 0.021440408 | 0.262577852 | 0.081653529 | 0.934922234 | 0.998182941 |
| P61019 | Ras-related protein Rab-2A | RAB2A | Homo sapiens | 9.001721641 | -0.011487905 | 0.142397513 | -0.080674898 | 0.9357005 | 0.998182941 |
| O00429 | Dynamin-1-like protein | DNM1L | Homo sapiens | 2.007822838 | -0.026586702 | 0.329896362 | -0.080591073 | 0.935767166 | 0.998182941 |
| O94855 | Protein transport protein Sec24D | SEC24D | Homo sapiens | 3.976430916 | -0.019105135 | 0.237118288 | -0.080572169 | 0.9357822 | 0.998182941 |
| P14868 | Aspartate--tRNA ligase, cytoplasmic | DARS1 | Homo sapiens | 8.295512807 | -0.011952158 | 0.150080294 | -0.079638422 | 0.936524836 | 0.998182941 |
| Q9NQ88 | Fructose-2,6-bisphosphatase TIGAR | TIGAR | Homo sapiens | 0.741008673 | 0.050877988 | 0.654390548 | 0.07774866 | 0.938027986 | NA |
| P09914 | Interferon-induced protein with tetratricopeptide repeats 1 | IFIT1 | Homo sapiens | 1.866456683 | 0.084614207 | 1.091636306 | 0.077511353 | 0.93821676 | 0.998182941 |
| O15145 | Actin-related protein 2/3 complex subunit 3 | ARPC3 | Homo sapiens | 0.845611683 | -0.035082232 | 0.458555823 | -0.076505914 | 0.939016609 | NA |
| Q13885 | Tubulin beta-2A chain | TUBB2A | Homo sapiens | 202.3161733 | -0.002581465 | 0.033833054 | -0.076300088 | 0.939180356 | 0.998182941 |
| Q8N8S7 | Protein enabled homolog | ENAH | Homo sapiens | 0.61484187 | 0.041287655 | 0.547659827 | 0.075389235 | 0.939905024 | NA |
| Q71DI3 | Histone H3.2 | H3C15 | Homo sapiens | 15.62018322 | -0.009012243 | 0.119737946 | -0.075266393 | 0.94000276 | 0.998182941 |
| P35637 | RNA-binding protein FUS | FUS | Homo sapiens | 16.16313864 | 0.007899725 | 0.105497965 | 0.074880357 | 0.940309906 | 0.998182941 |
| P47985 | Cytochrome b-c1 complex subunit Rieske, mitochondrial | UQCRFS1 | Homo sapiens | 1.273140303 | -0.032911731 | 0.439761355 | -0.07483998 | 0.940342031 | NA |
| P49189 | 4-trimethylaminobutyraldehyde dehydrogenase | ALDH9A1 | Homo sapiens | 1.422677751 | 0.02773187 | 0.370919205 | 0.074765259 | 0.940401484 | NA |
| P62314 | Small nuclear ribonucleoprotein Sm D1 | SNRPD1 | Homo sapiens | 6.009062815 | -0.013326806 | 0.178750906 | -0.07455518 | 0.940568636 | 0.998182941 |
| Q9NVD7 | Alpha-parvin | PARVA | Homo sapiens | 5.196948544 | -0.013945816 | 0.187089331 | -0.074540945 | 0.940579962 | 0.998182941 |
| Q9Y696 | Chloride intracellular channel protein 4 | CLIC4 | Homo sapiens | 12.34027918 | 0.009534686 | 0.128308326 | 0.074310733 | 0.940763137 | 0.998182941 |
| P02647 | Apolipoprotein A-I | APOA1 | Homo sapiens | 1.100855801 | -0.028762908 | 0.391790119 | -0.073414073 | 0.941476619 | NA |
| Q8N766 | ER membrane protein complex subunit 1 | EMC1 | Homo sapiens | 2.029941438 | -0.022606649 | 0.309043122 | -0.073150469 | 0.941686381 | 0.998182941 |
| Q9UQ80 | Proliferation-associated protein 2G4 | PA2G4 | Homo sapiens | 11.67671226 | -0.009301172 | 0.128765837 | -0.072233229 | 0.942416301 | 0.998182941 |
| P45877 | Peptidyl-prolyl cis-trans isomerase C | PPIC | Homo sapiens | 0.879853778 | 0.032669206 | 0.45982673 | 0.071046774 | 0.943360529 | NA |
| O14879 | Interferon-induced protein with tetratricopeptide repeats 3 | IFIT3 | Homo sapiens | 1.761568448 | 0.044718643 | 0.649138391 | 0.068889228 | 0.945077793 | 0.998182941 |
| P35613 | Basigin | BSG | Homo sapiens | 2.628026159 | -0.018670319 | 0.271528883 | -0.068759975 | 0.945180678 | 0.998182941 |
| P43686 | 26S proteasome regulatory subunit 6B | PSMC4 | Homo sapiens | 13.78594486 | 0.007615461 | 0.115578785 | 0.065889782 | 0.947465576 | 0.998182941 |
| Q13085 | Acetyl-CoA carboxylase 1 | ACACA | Homo sapiens | 1.430736973 | 0.025808765 | 0.399016082 | 0.064681014 | 0.94842798 | NA |
| Q9UJW0 | Dynactin subunit 4 | DCTN4 | Homo sapiens | 0.525967624 | -0.03971402 | 0.618326028 | -0.064228285 | 0.948788456 | NA |
| P00387 | NADH-cytochrome b5 reductase 3 | CYB5R3 | Homo sapiens | 8.54364804 | -0.009550041 | 0.14928971 | -0.063969852 | 0.948994232 | 0.998182941 |
| P29401 | Transketolase | TKT | Homo sapiens | 49.68548296 | -0.004114907 | 0.064655775 | -0.0636433 | 0.949254253 | 0.998182941 |
| Q13442 | 28 kDa heat- and acid-stable phosphoprotein | PDAP1 | Homo sapiens | 1.657134132 | -0.024045947 | 0.37902023 | -0.063442383 | 0.949414238 | NA |
| Q16629 | Serine/arginine-rich splicing factor 7 | SRSF7 | Homo sapiens | 1.946631079 | -0.019268223 | 0.309208383 | -0.062314684 | 0.950312235 | 0.998182941 |
| Q7Z406 | Myosin-14 | MYH14 | Homo sapiens | 23.35185727 | -0.005520038 | 0.08866441 | -0.062257651 | 0.950357653 | 0.998182941 |
| Q16527 | Cysteine and glycine-rich protein 2 | CSRP2 | Homo sapiens | 3.355735044 | -0.014862482 | 0.245031366 | -0.060655426 | 0.951633631 | 0.998182941 |
| P78344 | Eukaryotic translation initiation factor 4 gamma 2 | EIF4G2 | Homo sapiens | 3.505067172 | 0.014074345 | 0.237163245 | 0.059344545 | 0.952677682 | 0.998182941 |
| P50395 | Rab GDP dissociation inhibitor beta | GDI2 | Homo sapiens | 56.68803788 | -0.003471843 | 0.05924753 | -0.058598952 | 0.953271545 | 0.998182941 |
| Q13724 | Mannosyl-oligosaccharide glucosidase | MOGS | Homo sapiens | 5.642462272 | 0.011218186 | 0.191617767 | 0.058544602 | 0.953314836 | 0.998182941 |
| Q9H223 | EH domain-containing protein 4 | EHD4 | Homo sapiens | 3.678600276 | 0.013876035 | 0.237268252 | 0.058482477 | 0.95336432 | 0.998182941 |
| Q92542 | Nicastrin | NCSTN | Homo sapiens | 1.276766726 | -0.022948211 | 0.393675253 | -0.058292237 | 0.953515851 | NA |
| Q9BQ67 | Glutamate-rich WD repeat-containing protein 1 | GRWD1 | Homo sapiens | 0.882195158 | -0.027578083 | 0.476509376 | -0.057875216 | 0.953848024 | NA |
| Q9P0L0 | Vesicle-associated membrane protein-associated protein A | VAPA | Homo sapiens | 4.750654908 | -0.011411513 | 0.197201738 | -0.057867203 | 0.953854407 | 0.998182941 |
| P61224 | Ras-related protein Rap-1b | RAP1B | Homo sapiens | 6.890402474 | -0.009519729 | 0.164680677 | -0.0578072 | 0.953902203 | 0.998182941 |
| P05141 | ADP/ATP translocase 2 | SLC25A5 | Homo sapiens | 29.20069987 | -0.004420942 | 0.079738384 | -0.055443081 | 0.955785475 | 0.998182941 |
| P15173 | Myogenin | MYOG | Homo sapiens | 1.177430073 | -0.021612167 | 0.407798033 | -0.052997232 | 0.957734113 | NA |
| Q16666 | Gamma-interferon-inducible protein 16 | IFI16 | Homo sapiens | 1.188749631 | -0.026658423 | 0.507680363 | -0.05251025 | 0.958122129 | NA |
| Q8NHW5 | 60S acidic ribosomal protein P0-like | RPLP0P6 | Homo sapiens | 17.51339491 | -0.005389411 | 0.106308341 | -0.05069603 | 0.959567741 | 0.998182941 |
| P57721 | Poly(rC)-binding protein 3 | PCBP3 | Homo sapiens | 12.07456651 | -0.006179664 | 0.122496624 | -0.050447628 | 0.959765683 | 0.998182941 |
| P07910 | Heterogeneous nuclear ribonucleoproteins C1/C2 | HNRNPC | Homo sapiens | 15.91396084 | -0.005645737 | 0.111969719 | -0.050422001 | 0.959786104 | 0.998182941 |
| Q9Y3E5 | Peptidyl-tRNA hydrolase 2, mitochondrial | PTRH2 | Homo sapiens | 0.636611403 | -0.028001699 | 0.557017688 | -0.050270754 | 0.959906629 | NA |
| P08962 | CD63 antigen | CD63 | Homo sapiens | 2.689055615 | 0.013278115 | 0.266761049 | 0.049775316 | 0.960301437 | 0.998182941 |
| Q16777 | Histone H2A type 2-C | H2AC20 | Homo sapiens | 42.09581541 | 0.005775564 | 0.117471434 | 0.049165692 | 0.960787252 | 0.998182941 |
| Q9UMX0 | Ubiquilin-1 | UBQLN1 | Homo sapiens | 8.930521482 | 0.00704329 | 0.145163674 | 0.048519642 | 0.96130211 | 0.998182941 |
| Q8IXB1 | DnaJ homolog subfamily C member 10 | DNAJC10 | Homo sapiens | 0.67026445 | 0.02783107 | 0.576832614 | 0.048248087 | 0.961518527 | NA |
| Q7Z4V5 | Hepatoma-derived growth factor-related protein 2 | HDGFL2 | Homo sapiens | 1.032522317 | 0.020389399 | 0.424540009 | 0.048027037 | 0.961694695 | NA |
| P33991 | DNA replication licensing factor MCM4 | MCM4 | Homo sapiens | 3.747849674 | -0.010795702 | 0.229070794 | -0.047128236 | 0.962411023 | 0.998182941 |
| P05387 | 60S acidic ribosomal protein P2 | RPLP2 | Homo sapiens | 13.59079146 | -0.006012637 | 0.127769152 | -0.047058594 | 0.962466528 | 0.998182941 |
| P61978 | Heterogeneous nuclear ribonucleoprotein K | HNRNPK | Homo sapiens | 67.42146528 | 0.002377194 | 0.052132892 | 0.045598738 | 0.963630075 | 0.998182941 |
| Q9BUF5 | Tubulin beta-6 chain | TUBB6 | Homo sapiens | 118.069187 | 0.001869941 | 0.041614284 | 0.044935076 | 0.964159059 | 0.998182941 |
| O60551 | Glycylpeptide N-tetradecanoyltransferase 2 | NMT2 | Homo sapiens | 1.152060155 | 0.016620151 | 0.371742309 | 0.044708797 | 0.964339422 | NA |
| Q01844 | RNA-binding protein EWS | EWSR1 | Homo sapiens | 6.1057644 | -0.00752188 | 0.171379116 | -0.043890296 | 0.96499185 | 0.998182941 |
| P15170 | Eukaryotic peptide chain release factor GTP-binding subunit ERF3A | GSPT1 | Homo sapiens | 2.338146855 | -0.013212222 | 0.311126501 | -0.042465756 | 0.96612741 | 0.998182941 |
| O43681 | ATPase GET3 | GET3 | Homo sapiens | 2.125224397 | -0.013134391 | 0.32215371 | -0.04077057 | 0.967478801 | 0.998182941 |
| Q16658 | Fascin | FSCN1 | Homo sapiens | 51.71757966 | -0.002461397 | 0.061372807 | -0.040105668 | 0.968008883 | 0.998182941 |
| P62910 | 60S ribosomal protein L32 | RPL32 | Homo sapiens | 7.962221597 | 0.005973955 | 0.152882075 | 0.039075572 | 0.968830136 | 0.998182941 |
| P08708 | 40S ribosomal protein S17 | RPS17 | Homo sapiens | 11.40304156 | 0.005102522 | 0.131299148 | 0.038861803 | 0.96900057 | 0.998182941 |
| O95340 | Bifunctional 3'-phosphoadenosine 5'-phosphosulfate synthase 2 | PAPSS2 | Homo sapiens | 15.55701318 | 0.00452996 | 0.116973541 | 0.038726369 | 0.96910855 | 0.998182941 |
| O95831 | Apoptosis-inducing factor 1, mitochondrial | AIFM1 | Homo sapiens | 2.014833969 | -0.011158147 | 0.3020511 | -0.036941257 | 0.970531844 | 0.998182941 |
| P07954 | Fumarate hydratase, mitochondrial | FH | Homo sapiens | 5.650560106 | -0.006470755 | 0.182163283 | -0.03552173 | 0.971663719 | 0.998182941 |
| O15372 | Eukaryotic translation initiation factor 3 subunit H | EIF3H | Homo sapiens | 1.126703847 | -0.014399978 | 0.407004386 | -0.035380401 | 0.971776413 | NA |
| Q00535 | Cyclin-dependent-like kinase 5 | CDK5 | Homo sapiens | 1.103073628 | -0.014213024 | 0.415900415 | -0.034174105 | 0.972738316 | NA |
| Q16630 | Cleavage and polyadenylation specificity factor subunit 6 | CPSF6 | Homo sapiens | 3.39914695 | 0.007667104 | 0.229820023 | 0.03336134 | 0.973386439 | 0.998182941 |
| P82979 | SAP domain-containing ribonucleoprotein | SARNP | Homo sapiens | 2.472240981 | 0.008941976 | 0.270725271 | 0.033029706 | 0.973650899 | 0.998182941 |
| Q9H4B7 | Tubulin beta-1 chain | TUBB1 | Homo sapiens | 15.91934401 | 0.003787656 | 0.11575712 | 0.032720721 | 0.9738973 | 0.998182941 |
| Q9Y617 | Phosphoserine aminotransferase | PSAT1 | Homo sapiens | 3.911645882 | -0.008010293 | 0.246935919 | -0.03243875 | 0.974122161 | 0.998182941 |
| Q9HB07 | MYG1 exonuclease | MYG1 | Homo sapiens | 1.412973349 | 0.011198788 | 0.345945428 | 0.032371546 | 0.974175754 | NA |
| P31943 | Heterogeneous nuclear ribonucleoprotein H | HNRNPH1 | Homo sapiens | 30.21914434 | -0.002549603 | 0.078781608 | -0.032362915 | 0.974182636 | 0.998182941 |
| P31949 | Protein S100-A11 | S100A11 | Homo sapiens | 3.404148074 | -0.007809099 | 0.243252607 | -0.032102839 | 0.974390039 | 0.998182941 |
| P49257 | Protein ERGIC-53 | LMAN1 | Homo sapiens | 3.576815419 | 0.007884438 | 0.248495499 | 0.031728697 | 0.974688409 | 0.998182941 |
| P16070 | CD44 antigen | CD44 | Homo sapiens | 24.52772529 | 0.002858193 | 0.091548343 | 0.031220592 | 0.975093618 | 0.998182941 |
| O60264 | SWI/SNF-related matrix-associated actin-dependent regulator of chromatin subfamily A member 5 | SMARCA5 | Homo sapiens | 1.68895167 | -0.010870283 | 0.34961114 | -0.031092496 | 0.975195774 | NA |
| P61313 | 60S ribosomal protein L15 | RPL15 | Homo sapiens | 22.85530343 | -0.00277401 | 0.089403577 | -0.031027956 | 0.975247244 | 0.998182941 |
| P04899 | Guanine nucleotide-binding protein G(i) subunit alpha-2 | GNAI2 | Homo sapiens | 4.566153805 | -0.006138677 | 0.200722807 | -0.030582856 | 0.975602215 | 0.998182941 |
| O00159 | Unconventional myosin-Ic | MYO1C | Homo sapiens | 22.14222972 | -0.002815925 | 0.093502047 | -0.030116183 | 0.975974394 | 0.998182941 |
| O95819 | Mitogen-activated protein kinase kinase kinase kinase 4 | MAP4K4 | Homo sapiens | 1.786291405 | 0.009426542 | 0.316507633 | 0.029782986 | 0.976240128 | 0.998182941 |
| P62847 | 40S ribosomal protein S24 | RPS24 | Homo sapiens | 8.512425461 | 0.004310996 | 0.146291964 | 0.029468442 | 0.976490988 | 0.998182941 |
| P11802 | Cyclin-dependent kinase 4 | CDK4 | Homo sapiens | 1.354728068 | 0.010492591 | 0.359413725 | 0.029193628 | 0.976710163 | NA |
| Q13310 | Polyadenylate-binding protein 4 | PABPC4 | Homo sapiens | 15.33468103 | -0.003041361 | 0.107665562 | -0.028248226 | 0.977464173 | 0.998182941 |
| P20936 | Ras GTPase-activating protein 1 | RASA1 | Homo sapiens | 1.950088038 | -0.008065472 | 0.306708429 | -0.026296872 | 0.97902055 | 0.998182941 |
| Q9HAU5 | Regulator of nonsense transcripts 2 | UPF2 | Homo sapiens | 2.026203872 | 0.008456619 | 0.324956988 | 0.026023811 | 0.979238346 | 0.998182941 |
| P49023 | Paxillin | PXN | Homo sapiens | 6.330206599 | 0.004507994 | 0.177110343 | 0.025453022 | 0.979693619 | 0.998182941 |
| Q07020 | 60S ribosomal protein L18 | RPL18 | Homo sapiens | 6.207460875 | 0.004451025 | 0.176946117 | 0.02515469 | 0.979931578 | 0.998182941 |
| O14980 | Exportin-1 | XPO1 | Homo sapiens | 10.56283424 | -0.003361245 | 0.134618276 | -0.024968715 | 0.980079918 | 0.998182941 |
| Q08AM6 | Protein VAC14 homolog | VAC14 | Homo sapiens | 0.820617887 | -0.010996076 | 0.450038395 | -0.02443364 | 0.980506716 | NA |
| Q9ULV4 | Coronin-1C | CORO1C | Homo sapiens | 14.28097856 | -0.002908087 | 0.119308219 | -0.024374577 | 0.980553827 | 0.998182941 |
| Q9UDY4 | DnaJ homolog subfamily B member 4 | DNAJB4 | Homo sapiens | 3.873398315 | 0.005460753 | 0.232152747 | 0.02352224 | 0.981233699 | 0.998182941 |
| P00403 | Cytochrome c oxidase subunit 2 | MT-CO2 | Homo sapiens | 0.603370794 | 0.012035119 | 0.52617216 | 0.022872969 | 0.981751602 | NA |
| P98082 | Disabled homolog 2 | DAB2 | Homo sapiens | 4.059179674 | 0.005149932 | 0.231135278 | 0.022281028 | 0.982223783 | 0.998182941 |
| O75822 | Eukaryotic translation initiation factor 3 subunit J | EIF3J | Homo sapiens | 0.481865992 | -0.012284309 | 0.584132778 | -0.021029994 | 0.983221729 | NA |
| Q9BZK7 | F-box-like/WD repeat-containing protein TBL1XR1 | TBL1XR1 | Homo sapiens | 0.990885536 | 0.009257533 | 0.449516002 | 0.020594445 | 0.983569172 | NA |
| Q9H254 | Spectrin beta chain, non-erythrocytic 4 | SPTBN4 | Homo sapiens | 3.342668726 | -0.004719914 | 0.234764277 | -0.020104906 | 0.983959687 | 0.998182941 |
| Q07955 | Serine/arginine-rich splicing factor 1 | SRSF1 | Homo sapiens | 18.93353259 | -0.001927587 | 0.098630105 | -0.019543599 | 0.984407457 | 0.998182941 |
| Q16181 | Septin-7 | SEPTIN7 | Homo sapiens | 19.31280739 | 0.001873574 | 0.097261065 | 0.019263349 | 0.984631022 | 0.998182941 |
| P55735 | Protein SEC13 homolog | SEC13 | Homo sapiens | 5.079014363 | -0.00360157 | 0.190137687 | -0.018941903 | 0.984887452 | 0.998182941 |
| Q5JWF2 | Guanine nucleotide-binding protein G(s) subunit alpha isoforms XLas | GNAS | Homo sapiens | 1.60471097 | -0.006433802 | 0.353609081 | -0.018194673 | 0.985483552 | NA |
| Q6L8Q7 | 2',5'-phosphodiesterase 12 | PDE12 | Homo sapiens | 0.528260665 | 0.011277736 | 0.63043711 | 0.017888756 | 0.985727599 | NA |
| P62195 | 26S proteasome regulatory subunit 8 | PSMC5 | Homo sapiens | 6.144055442 | 0.003095052 | 0.173132176 | 0.017876817 | 0.985737124 | 0.998182941 |
| Q13200 | 26S proteasome non-ATPase regulatory subunit 2 | PSMD2 | Homo sapiens | 29.39889482 | -0.001458004 | 0.083850588 | -0.017388123 | 0.986126984 | 0.998182941 |
| Q15393 | Splicing factor 3B subunit 3 | SF3B3 | Homo sapiens | 11.88877975 | -0.002134067 | 0.127668211 | -0.016715726 | 0.986663401 | 0.998182941 |
| P63151 | Serine/threonine-protein phosphatase 2A 55 kDa regulatory subunit B alpha isoform | PPP2R2A | Homo sapiens | 4.093684926 | 0.003408349 | 0.211638005 | 0.016104619 | 0.987150929 | 0.998182941 |
| Q9BPW5 | Ras-like protein family member 11B | RASL11B | Homo sapiens | 1.09841311 | -0.008265808 | 0.561417506 | -0.014723104 | 0.988253087 | NA |
| P62851 | 40S ribosomal protein S25 | RPS25 | Homo sapiens | 13.25006166 | -0.001755485 | 0.124009268 | -0.014156081 | 0.988705459 | 0.998182941 |
| O60763 | General vesicular transport factor p115 | USO1 | Homo sapiens | 7.144360802 | -0.002024606 | 0.163713133 | -0.012366792 | 0.990132979 | 0.998182941 |
| Q9Y281 | Cofilin-2 | CFL2 | Homo sapiens | 23.90427606 | -0.001082293 | 0.087702475 | -0.01234051 | 0.990153948 | 0.998182941 |
| Q07666 | KH domain-containing, RNA-binding, signal transduction-associated protein 1 | KHDRBS1 | Homo sapiens | 6.365005171 | 0.001838979 | 0.174951355 | 0.010511375 | 0.99161329 | 0.998182941 |
| Q15366 | Poly(rC)-binding protein 2 | PCBP2 | Homo sapiens | 26.14689995 | -0.000848224 | 0.083506649 | -0.010157562 | 0.991895577 | 0.998182941 |
| Q13509 | Tubulin beta-3 chain | TUBB3 | Homo sapiens | 140.3666382 | -0.000376102 | 0.038287306 | -0.00982314 | 0.992162395 | 0.998182941 |
| P58546 | Myotrophin | MTPN | Homo sapiens | 1.515052717 | -0.003218285 | 0.346332361 | -0.009292475 | 0.992585784 | NA |
| Q9Y4K0 | Lysyl oxidase homolog 2 | LOXL2 | Homo sapiens | 0.84333515 | 0.004276112 | 0.492863686 | 0.008676053 | 0.993077598 | NA |
| Q9UKY7 | Protein CDV3 homolog | CDV3 | Homo sapiens | 4.478200943 | 0.002003824 | 0.231954422 | 0.008638871 | 0.993107264 | 0.998182941 |
| P05091 | Aldehyde dehydrogenase, mitochondrial | ALDH2 | Homo sapiens | 3.920981923 | 0.001960699 | 0.236267014 | 0.008298659 | 0.993378704 | 0.998182941 |
| P51571 | Translocon-associated protein subunit delta | SSR4 | Homo sapiens | 4.110797149 | -0.001823554 | 0.221381149 | -0.008237171 | 0.993427763 | 0.998182941 |
| Q14137 | Ribosome biogenesis protein BOP1 | BOP1 | Homo sapiens | 0.590695566 | 0.004231453 | 0.541328394 | 0.007816795 | 0.993763163 | NA |
| Q96JJ7 | Protein disulfide-isomerase TMX3 | TMX3 | Homo sapiens | 1.390235039 | 0.00286215 | 0.387863651 | 0.007379268 | 0.994112249 | NA |
| P20340 | Ras-related protein Rab-6A | RAB6A | Homo sapiens | 1.90002832 | -0.002044334 | 0.298561742 | -0.006847273 | 0.994536709 | 0.998182941 |
| Q9Y6C9 | Mitochondrial carrier homolog 2 | MTCH2 | Homo sapiens | 2.89965815 | 0.001773354 | 0.26413971 | 0.006713697 | 0.994643285 | 0.998182941 |
| Q8N1F7 | Nuclear pore complex protein Nup93 | NUP93 | Homo sapiens | 1.687690793 | -0.001939011 | 0.334435047 | -0.005797872 | 0.995373994 | NA |
| Q9Y490 | Talin-1 | TLN1 | Homo sapiens | 159.0426073 | 0.0002019 | 0.036550677 | 0.005523836 | 0.995592639 | 0.998247553 |
| Q6WCQ1 | Myosin phosphatase Rho-interacting protein | MPRIP | Homo sapiens | 1.463837362 | 0.001530565 | 0.372118901 | 0.004113106 | 0.996718225 | NA |
| Q9Y285 | Phenylalanine--tRNA ligase alpha subunit | FARSA | Homo sapiens | 1.648560099 | -0.001237511 | 0.33591603 | -0.003683991 | 0.997060608 | NA |
| Q6P597 | Kinesin light chain 3 | KLC3 | Homo sapiens | 0.693067058 | -0.001845348 | 0.518334421 | -0.00356015 | 0.997159417 | NA |
| Q01105 | Protein SET | SET | Homo sapiens | 11.90457435 | -0.000414223 | 0.147174347 | -0.002814504 | 0.997754354 | 0.998740635 |
| Q9Y536 | Peptidyl-prolyl cis-trans isomerase A-like 4A | PPIAL4A | Homo sapiens | 2.019303738 | -0.000855195 | 0.323190434 | -0.002646101 | 0.997888719 | 0.998740635 |
| Q6NYC8 | Phostensin | PPP1R18 | Homo sapiens | 4.395936674 | -0.000339678 | 0.21520666 | -0.001578381 | 0.998740635 | 0.998740635 |
| Q7L1Q6 | Basic leucine zipper and W2 domain-containing protein 1 | BZW1 | Homo sapiens | 1.628073803 | -0.000404332 | 0.33718234 | -0.001199149 | 0.999043218 | NA |
| Q9NRP0 | Oligosaccharyltransferase complex subunit OSTC | OSTC | Homo sapiens | 0.857475003 | 8.76E-05 | 0.453275693 | 0.000193272 | 0.999845791 | NA |

**Supplementary Table S1B.** Full details of the 19 significant proteins identified in PPIP5K2-knockdown HCF cells with TGFβ1 treatment and CMS status as cofactors, compared to those HCFs without PPIP5K2 knockdown, using FDR-adjusted p-value ≤0.10.

| Accession | Description | Gene Name | baseMean | log2  Fold Change | lfcSE | stat | p-value | FDR-adjusted p-value |
| --- | --- | --- | --- | --- | --- | --- | --- | --- |
| P04179 | Superoxide dismutase [Mn], mitochondrial | SOD2 | 3.83 | -1.89 | 0.31 | -6.08 | 1.2091E-09 | 1.23E-06 |
| Q9Y6N5 | Sulfide:quinone oxidoreductase, mitochondrial | SQOR | 5.65 | -1.32 | 0.22 | -5.98 | 2.1833E-09 | 1.23E-06 |
| P04406 | Glyceraldehyde-3-phosphate dehydrogenase | GAPDH | 294.82 | -0.23 | 0.04 | -5.86 | 4.5087E-09 | 1.70E-06 |
| O00469 | Procollagen-lysine,2-oxoglutarate 5-dioxygenase 2 | PLOD2 | 13.85 | -0.69 | 0.13 | -5.35 | 8.5558E-08 | 2.41E-05 |
| P46821 | Microtubule-associated protein 1B | MAP1B | 69.46 | -0.37 | 0.07 | -5.30 | 1.1666E-07 | 2.63E-05 |
| P04075 | Fructose-bisphosphate aldolase A | ALDOA | 88.55 | -0.23 | 0.05 | -4.50 | 6.9067E-06 | 1.30E-03 |
| P17301 | Integrin alpha-2 | ITGA2 | 26.42 | -0.54 | 0.13 | -4.08 | 4.5782E-05 | 7.38E-03 |
| P21980 | Protein-glutamine gamma-glutamyltransferase 2 | TGM2 | 2.18 | -1.38 | 0.36 | -3.86 | 1.1298E-04 | 1.59E-02 |
| Q9NR30 | Nucleolar RNA helicase 2 | DDX21 | 10.71 | 0.55 | 0.14 | 3.81 | 1.3911E-04 | 1.74E-02 |
| P07237 | Protein disulfide-isomerase | P4HB | 91.00 | -0.20 | 0.05 | -3.67 | 2.4320E-04 | 2.74E-02 |
| P00558 | Phosphoglycerate kinase 1 | PGK1 | 85.31 | -0.17 | 0.05 | -3.50 | 4.6186E-04 | 4.74E-02 |
| P00338 | L-lactate dehydrogenase A chain | LDHA | 68.74 | -0.19 | 0.05 | -3.40 | 6.8556E-04 | 6.12E-02 |
| P14618 | Pyruvate kinase PKM | PKM | 287.64 | -0.10 | 0.03 | -3.39 | 7.0551E-04 | 6.12E-02 |
| Q9HB71 | Calcyclin-binding protein | CACYBP | 2.97 | 0.94 | 0.28 | 3.34 | 8.3333E-04 | 6.27E-02 |
| P06899 | Histone H2B type 1-J | H2BC11 | 14.98 | 0.51 | 0.15 | 3.34 | 8.3372E-04 | 6.27E-02 |
| Q14108 | Lysosome membrane protein 2 | SCARB2 | 5.96 | -0.60 | 0.18 | -3.26 | 1.1087E-03 | 7.82E-02 |
| Q14914 | Prostaglandin reductase 1 | PTGR1 | 4.85 | -0.90 | 0.28 | -3.21 | 1.3121E-03 | 8.71E-02 |
| Q06210 | Glutamine--fructose-6-phosphate aminotransferase [isomerizing] 1 | GFPT1 | 19.53 | -0.33 | 0.10 | -3.16 | 1.5516E-03 | 9.72E-02 |
| P22626 | Heterogeneous nuclear ribonucleoproteins A2/B1 | HNRNPA2B1 | 59.98 | 0.17 | 0.06 | 3.14 | 1.6662E-03 | 9.89E-02 |

**Supplementary Table S1C**. Significant proteins identified in the PPIP5K2-knockdown HCF cells with TGFβ1 treatment and CMS status as cofactors, compared to those HCFs without PPIP5K2 knockdown, using the cutoff of p-value ≤0.05.

| Accession | Description | Gene Name | baseMean | log2 Fold Change | lfcSE | stat | p-value | FDR-adjusted p-value |
| --- | --- | --- | --- | --- | --- | --- | --- | --- |
| P04179 | Superoxide dismutase [Mn], mitochondrial | SOD2 | 3.827286543 | -1.886837703 | 0.310384606 | -6.0790312 | 1.21E-09 | 1.23E-06 |
| Q9Y6N5 | Sulfide:quinone oxidoreductase, mitochondrial | SQOR | 5.648694408 | -1.319805421 | 0.220572322 | -5.983549568 | 2.18E-09 | 1.23E-06 |
| P04406 | Glyceraldehyde-3-phosphate dehydrogenase | GAPDH | 294.8185616 | -0.23065628 | 0.039331881 | -5.864359301 | 4.51E-09 | 1.70E-06 |
| O00469 | Procollagen-lysine,2-oxoglutarate 5-dioxygenase 2 | PLOD2 | 13.85104678 | -0.686098907 | 0.128123114 | -5.354997115 | 8.56E-08 | 2.41E-05 |
| P46821 | Microtubule-associated protein 1B | MAP1B | 69.45629736 | -0.371656664 | 0.070141646 | -5.298658984 | 1.17E-07 | 2.63E-05 |
| P04075 | Fructose-bisphosphate aldolase A | ALDOA | 88.55034928 | -0.231730659 | 0.051535287 | -4.496543446 | 6.91E-06 | 0.001298461 |
| P17301 | Integrin alpha-2 | ITGA2 | 26.41743162 | -0.542608163 | 0.133116958 | -4.076176115 | 4.58E-05 | 0.007377499 |
| P21980 | Protein-glutamine gamma-glutamyltransferase 2 | TGM2 | 2.18265185 | -1.380109681 | 0.357460667 | -3.860871438 | 0.000112983 | 0.015930651 |
| Q9NR30 | Nucleolar RNA helicase 2 | DDX21 | 10.70539072 | 0.550656977 | 0.144538983 | 3.809747139 | 0.000139109 | 0.017434994 |
| P07237 | Protein disulfide-isomerase | P4HB | 91.00120091 | -0.195123337 | 0.053177005 | -3.66931789 | 0.000243199 | 0.027432792 |
| P00558 | Phosphoglycerate kinase 1 | PGK1 | 85.30931736 | -0.172743628 | 0.049327774 | -3.501954664 | 0.000461858 | 0.047361459 |
| P00338 | L-lactate dehydrogenase A chain | LDHA | 68.73577007 | -0.186289287 | 0.054866995 | -3.395288688 | 0.000685563 | 0.061216691 |
| P14618 | Pyruvate kinase PKM | PKM | 287.6436456 | -0.104401959 | 0.030820419 | -3.387428317 | 0.000705512 | 0.061216691 |
| Q9HB71 | Calcyclin-binding protein | CACYBP | 2.968795966 | 0.939121994 | 0.281049726 | 3.341479844 | 0.000833331 | 0.062695408 |
| P06899 | Histone H2B type 1-J | H2BC11 | 14.98095805 | 0.506106703 | 0.151467656 | 3.341351661 | 0.000833716 | 0.062695408 |
| Q14108 | Lysosome membrane protein 2 | SCARB2 | 5.955731073 | -0.603194204 | 0.184949856 | -3.261393206 | 0.001108662 | 0.07816066 |
| Q14914 | Prostaglandin reductase 1 | PTGR1 | 4.84516069 | -0.895266713 | 0.278611765 | -3.213312663 | 0.001312134 | 0.087063927 |
| Q06210 | Glutamine--fructose-6-phosphate aminotransferase [isomerizing] 1 | GFPT1 | 19.53188985 | -0.330994726 | 0.104584611 | -3.164851134 | 0.001551623 | 0.097235051 |
| P22626 | Heterogeneous nuclear ribonucleoproteins A2/B1 | HNRNPA2B1 | 59.97775342 | 0.174551162 | 0.055517802 | 3.144057529 | 0.001666227 | 0.098921254 |
| Q8IUE6 | Histone H2A type 2-B | H2AC21 | 4.051539189 | 0.812759486 | 0.260170001 | 3.123955422 | 0.001784375 | 0.100638743 |
| P42166 | Lamina-associated polypeptide 2, isoform alpha | TMPO | 4.639938645 | 0.647277133 | 0.214313721 | 3.020231881 | 0.002525812 | 0.135672203 |
| P13010 | X-ray repair cross-complementing protein 5 | XRCC5 | 20.56463212 | 0.301585326 | 0.10092833 | 2.988113699 | 0.002807051 | 0.140328462 |
| P20700 | Lamin-B1 | LMNB1 | 7.832015185 | 0.472584144 | 0.158634462 | 2.979076163 | 0.002891189 | 0.140328462 |
| P35754 | Glutaredoxin-1 | GLRX | 2.696621426 | -1.168217097 | 0.393444386 | -2.969205146 | 0.002985712 | 0.140328462 |
| P43490 | Nicotinamide phosphoribosyltransferase | NAMPT | 3.902009212 | -0.671509156 | 0.229872563 | -2.921223606 | 0.003486595 | 0.151445869 |
| Q9Y2D5 | A-kinase anchor protein 2 | AKAP2 | 4.304103459 | -0.662266175 | 0.226827726 | -2.919687943 | 0.00350382 | 0.151445869 |
| Q8IVF2 | Protein AHNAK2 | AHNAK2 | 1.871858894 | 1.093729516 | 0.376514373 | 2.904881182 | 0.003673925 | 0.151445869 |
| Q15149 | Plectin | PLEC | 159.8969962 | 0.216292708 | 0.074890077 | 2.888135757 | 0.003875326 | 0.151445869 |
| P15144 | Aminopeptidase N | ANPEP | 10.29794596 | -0.464367516 | 0.160866752 | -2.886659362 | 0.003893555 | 0.151445869 |
| P48681 | Nestin | NES | 4.007349196 | 0.709975597 | 0.253020666 | 2.805998452 | 0.005016093 | 0.182552134 |
| Q15437 | Protein transport protein Sec23B | SEC23B | 6.973120505 | -0.461882962 | 0.165100729 | -2.79758281 | 0.005148657 | 0.182552134 |
| Q16222 | UDP-N-acetylhexosamine pyrophosphorylase | UAP1 | 3.446334954 | 0.704260576 | 0.251908714 | 2.795697556 | 0.005178784 | 0.182552134 |
| Q96D15 | Reticulocalbin-3 | RCN3 | 5.95830039 | -0.591400936 | 0.213208233 | -2.773818482 | 0.005540258 | 0.189359649 |
| Q96T76 | MMS19 nucleotide excision repair protein homolog | MMS19 | 1.997705387 | 0.939064621 | 0.339733628 | 2.764120312 | 0.005707649 | 0.189359649 |
| P09936 | Ubiquitin carboxyl-terminal hydrolase isozyme L1 | UCHL1 | 17.1484001 | -0.298739925 | 0.109036566 | -2.739814133 | 0.006147394 | 0.193079897 |
| A5A3E0 | POTE ankyrin domain family member F | POTEF | 81.36538604 | -0.151822882 | 0.055429487 | -2.739027368 | 0.006162124 | 0.193079897 |
| P39748 | Flap endonuclease 1 | FEN1 | 0.871322361 | 1.343890965 | 0.491867688 | 2.732220465 | 0.006290903 | NA |
| P01889 | HLA class I histocompatibility antigen, B alpha chain | HLA-B | 3.654785272 | -0.63398274 | 0.238066839 | -2.663045143 | 0.007743702 | 0.235626498 |
| P09493 | Tropomyosin alpha-1 chain | TPM1 | 5.222368099 | -0.656059642 | 0.247130874 | -2.654705302 | 0.007937772 | 0.235626498 |
| P11277 | Spectrin beta chain, erythrocytic | SPTB | 0.880603621 | -1.295630368 | 0.489089585 | -2.649065548 | 0.008071468 | NA |
| P53396 | ATP-citrate synthase | ACLY | 62.79748371 | -0.150855199 | 0.057985327 | -2.601609846 | 0.009278734 | 0.260618167 |
| P21796 | Voltage-dependent anion-selective channel protein 1 | VDAC1 | 23.03881725 | -0.348796934 | 0.134402078 | -2.595175164 | 0.009454272 | 0.260618167 |
| Q9BQG0 | Myb-binding protein 1A | MYBBP1A | 2.841275812 | 0.688510429 | 0.265689357 | 2.591411399 | 0.009558315 | 0.260618167 |
| Q14315 | Filamin-C | FLNC | 146.122025 | -0.168014843 | 0.065108438 | -2.580538707 | 0.00986463 | 0.260618167 |
| P52895 | Aldo-keto reductase family 1 member C2 | AKR1C2 | 5.105652422 | -0.740025842 | 0.287044613 | -2.578086503 | 0.009934912 | 0.260618167 |
| P08133 | Annexin A6 | ANXA6 | 19.9749562 | -0.373144763 | 0.148251551 | -2.51697038 | 0.011836878 | 0.303454508 |
| Q15436 | Protein transport protein Sec23A | SEC23A | 18.23814561 | -0.26742296 | 0.106777652 | -2.50448437 | 0.012263002 | 0.307392592 |
| P10599 | Thioredoxin | TXN | 5.294389051 | -0.517498896 | 0.207931528 | -2.488794749 | 0.012817694 | 0.308881399 |
| Q04828 | Aldo-keto reductase family 1 member C1 | AKR1C1 | 3.750335207 | -0.666499242 | 0.267956108 | -2.487344833 | 0.012870058 | 0.308881399 |
|  |  |  |  |  |  |  |  |  |
| Q9NY65 | Tubulin alpha-8 chain | TUBA8 | 49.4652756 | -0.217228701 | 0.087623861 | -2.47910442 | 0.013171274 | 0.309524939 |
| P18621 | 60S ribosomal protein L17 | RPL17 | 12.61527373 | 0.297990557 | 0.121201767 | 2.458632119 | 0.013946745 | 0.310417804 |
| P28300 | Protein-lysine 6-oxidase | LOX | 2.69737661 | 0.862817149 | 0.352375902 | 2.448570248 | 0.014342446 | 0.310417804 |
| P05121 | Plasminogen activator inhibitor 1 | SERPINE1 | 8.958393289 | -0.439881335 | 0.181142754 | -2.428368378 | 0.015166929 | 0.310417804 |
| Q96CX2 | BTB/POZ domain-containing protein KCTD12 | KCTD12 | 15.77576205 | -0.285381023 | 0.11775604 | -2.423493724 | 0.015372019 | 0.310417804 |
| Q969X5 | Endoplasmic reticulum-Golgi intermediate compartment protein 1 | ERGIC1 | 1.598268765 | -0.899228714 | 0.372053822 | -2.416931797 | 0.01565195 | NA |
| Q70UQ0 | Inhibitor of nuclear factor kappa-B kinase-interacting protein | IKBIP | 4.055260659 | -0.662059502 | 0.274041375 | -2.41591074 | 0.015695909 | 0.310417804 |
| Q6AWC2 | Protein WWC2 | WWC2 | 0.659881434 | 1.315819193 | 0.545278305 | 2.41311488 | 0.015816833 | NA |
| Q9UPN3 | Microtubule-actin cross-linking factor 1, isoforms 1/2/3/5 | MACF1 | 3.741430952 | -0.575892073 | 0.238968146 | -2.409911452 | 0.015956393 | 0.310417804 |
| P04083 | Annexin A1 | ANXA1 | 33.05532248 | -0.191715408 | 0.07972283 | -2.404774255 | 0.016182459 | 0.310417804 |
| P30041 | Peroxiredoxin-6 | PRDX6 | 13.09802476 | -0.324860253 | 0.135290498 | -2.401205244 | 0.016341168 | 0.310417804 |
| P16401 | Histone H1.5 | H1-5 | 14.47277004 | 0.279193463 | 0.116297146 | 2.400690577 | 0.016364167 | 0.310417804 |
| Q9HDC9 | Adipocyte plasma membrane-associated protein | APMAP | 5.839341822 | -0.456241866 | 0.190200289 | -2.398744329 | 0.016451397 | 0.310417804 |
| P12814 | Alpha-actinin-1 | ACTN1 | 108.9410106 | -0.131775582 | 0.05493823 | -2.398613537 | 0.016457274 | 0.310417804 |
| P14625 | Endoplasmin | HSP90B1 | 99.2792623 | -0.114662003 | 0.047827514 | -2.397406701 | 0.016511585 | 0.310417804 |
| O94808 | Glutamine--fructose-6-phosphate aminotransferase [isomerizing] 2 | GFPT2 | 7.5587698 | -0.480011686 | 0.201442305 | -2.38287427 | 0.01717806 | 0.31765331 |
| Q9BQ39 | ATP-dependent RNA helicase DDX50 | DDX50 | 0.751751344 | 1.21676203 | 0.512619892 | 2.373614542 | 0.017614929 | NA |
| P07602 | Prosaposin | PSAP | 8.095391184 | -0.41383002 | 0.17480517 | -2.367378612 | 0.017914596 | 0.325930064 |
| P60174 | Triosephosphate isomerase | TPI1 | 52.90565514 | -0.145678098 | 0.061956304 | -2.351303876 | 0.018707749 | 0.334957789 |
| Q14956 | Transmembrane glycoprotein NMB | GPNMB | 1.709939151 | -0.826863943 | 0.352582871 | -2.34516198 | 0.019018815 | NA |
| Q16881 | Thioredoxin reductase 1, cytoplasmic | TXNRD1 | 28.80939167 | -0.203379811 | 0.087553118 | -2.322930534 | 0.020182885 | 0.35572334 |
| Q03405 | Urokinase plasminogen activator surface receptor | PLAUR | 1.94364584 | -0.834969807 | 0.363324621 | -2.298137147 | 0.021553985 | 0.374044533 |
| P84996 | Protein ALEX | GNAS | 1.44870162 | 1.070660627 | 0.468339685 | 2.286077099 | 0.022249749 | NA |
| P15559 | NAD(P)H dehydrogenase [quinone] 1 | NQO1 | 14.17011403 | -0.289050923 | 0.126818599 | -2.279247093 | 0.022652381 | 0.387149787 |
| P18669 | Phosphoglycerate mutase 1 | PGAM1 | 13.96273978 | -0.448270508 | 0.197369593 | -2.271223752 | 0.023133436 | 0.388821474 |
| Q8WWM7 | Ataxin-2-like protein | ATXN2L | 1.983556751 | 0.719263178 | 0.317388369 | 2.266192619 | 0.023439592 | 0.388821474 |
| Q14683 | Structural maintenance of chromosomes protein 1A | SMC1A | 1.293707112 | 0.997141635 | 0.440033081 | 2.266060616 | 0.023447672 | NA |
| P42167 | Lamina-associated polypeptide 2, isoforms beta/gamma | TMPO | 6.396704429 | 0.394580259 | 0.175977817 | 2.242215901 | 0.024947422 | 0.407220105 |
| P27797 | Calreticulin | CALR | 56.35528988 | -0.1392784 | 0.062254603 | -2.237238578 | 0.025270751 | 0.407220105 |
| P33316 | Deoxyuridine 5'-triphosphate nucleotidohydrolase, mitochondrial | DUT | 0.832407744 | 1.110857038 | 0.49673685 | 2.2363089 | 0.025331544 | NA |
| O15355 | Protein phosphatase 1G | PPM1G | 0.983470092 | 1.152206393 | 0.518427409 | 2.222502849 | 0.026249342 | NA |
| P62495 | Eukaryotic peptide chain release factor subunit 1 | ETF1 | 8.271750928 | 0.335067545 | 0.150793531 | 2.222028643 | 0.02628137 | 0.416229462 |
| Q9HC07 | Transmembrane protein 165 | TMEM165 | 3.944478001 | -0.503918993 | 0.227214754 | -2.217809296 | 0.026567838 | 0.416229462 |
| Q8NEY1 | Neuron navigator 1 | NAV1 | 0.544595461 | 1.25272774 | 0.566623442 | 2.210864654 | 0.027045211 | NA |
| P17655 | Calpain-2 catalytic subunit | CAPN2 | 40.88738806 | -0.155019439 | 0.071187026 | -2.177636104 | 0.029433139 | 0.454802483 |
| Q6PIU2 | Neutral cholesterol ester hydrolase 1 | NCEH1 | 2.99170878 | -0.564445131 | 0.261286958 | -2.16024992 | 0.030753328 | 0.468780452 |
| P67936 | Tropomyosin alpha-4 chain | TPM4 | 12.39741629 | -0.340442636 | 0.158554072 | -2.147170554 | 0.031779706 | 0.474019574 |
| P08123 | Collagen alpha-2(I) chain | COL1A2 | 12.13820933 | 0.322103857 | 0.150151519 | 2.145192124 | 0.031937489 | 0.474019574 |
| O43660 | Pleiotropic regulator 1 | PLRG1 | 1.153430181 | 0.86229234 | 0.403404829 | 2.137535989 | 0.032554422 | NA |
| Q9H2G2 | STE20-like serine/threonine-protein kinase | SLK | 1.001954295 | 0.971739317 | 0.455015636 | 2.135617417 | 0.032710611 | NA |
| Q9Y3A5 | Ribosome maturation protein SBDS | SBDS | 0.760646687 | -1.148781215 | 0.542814682 | -2.116341458 | 0.034315777 | NA |
| P13797 | Plastin-3 | PLS3 | 21.07765008 | -0.212485429 | 0.100533728 | -2.11357357 | 0.034551705 | 0.506160046 |
| P13674 | Prolyl 4-hydroxylase subunit alpha-1 | P4HA1 | 11.83303278 | -0.323590127 | 0.154069079 | -2.100292474 | 0.035703121 | 0.514253409 |
| Q9BRF8 | Serine/threonine-protein phosphatase CPPED1 | CPPED1 | 0.641171218 | -1.293979273 | 0.616129651 | -2.100173675 | 0.035713566 | NA |
| Q53GQ0 | Very-long-chain 3-oxoacyl-CoA reductase | HSD17B12 | 1.835043027 | -0.703513998 | 0.336093084 | -2.093211764 | 0.036330255 | 0.514253409 |
| O75534 | Cold shock domain-containing protein E1 | CSDE1 | 4.236853426 | 0.444053825 | 0.21230065 | 2.091627249 | 0.036471873 | 0.514253409 |
| O15118 | NPC intracellular cholesterol transporter 1 | NPC1 | 1.499547945 | -0.805365565 | 0.386396305 | -2.084299345 | 0.037132947 | NA |
| Q9Y5S1 | Transient receptor potential cation channel subfamily V member 2 | TRPV2 | 1.122621661 | -0.946404991 | 0.456157059 | -2.074734944 | 0.038011107 | NA |
| P01033 | Metalloproteinase inhibitor 1 | TIMP1 | 1.151126581 | -0.915980241 | 0.443222546 | -2.066637291 | 0.038768344 | NA |
| P00505 | Aspartate aminotransferase, mitochondrial | GOT2 | 4.847586436 | -0.398946535 | 0.194477083 | -2.051380695 | 0.040229888 | 0.557204715 |
| P36578 | 60S ribosomal protein L4 | RPL4 | 38.83111079 | 0.142987001 | 0.069799084 | 2.048551242 | 0.040506017 | 0.557204715 |
| P0DME0 | Protein SETSIP | SETSIP | 1.367600944 | -0.847491573 | 0.415144525 | -2.041437434 | 0.041207367 | NA |
| P69905 | Hemoglobin subunit alpha | HBA1 | 47.59033187 | -0.15484732 | 0.076098159 | -2.034836629 | 0.041867314 | 0.568656347 |
| P09429 | High mobility group protein B1 | HMGB1 | 6.437889827 | 0.361753284 | 0.178223829 | 2.029769447 | 0.04237998 | 0.568656347 |
| P32119 | Peroxiredoxin-2 | PRDX2 | 13.85720049 | -0.239986301 | 0.118502356 | -2.025160588 | 0.042850877 | 0.568656347 |
| Q8NG11 | Tetraspanin-14 | TSPAN14 | 2.031371434 | -0.664244213 | 0.330855706 | -2.007655303 | 0.044679933 | 0.586034475 |
| P08727 | Keratin, type I cytoskeletal 19 | KRT19 | 3.044482107 | 0.565141589 | 0.283666498 | 1.992274704 | 0.046340926 | 0.60083407 |
| P30101 | Protein disulfide-isomerase A3 | PDIA3 | 56.53702673 | -0.116999055 | 0.058896449 | -1.986521364 | 0.046975463 | 0.602140026 |
| Q9NYU2 | UDP-glucose:glycoprotein glucosyltransferase 1 | UGGT1 | 5.28287694 | -0.412255674 | 0.208103089 | -1.981016598 | 0.047589412 | 0.603155697 |
| Q15417 | Calponin-3 | CNN3 | 21.15203789 | -0.195060459 | 0.09878538 | -1.97458833 | 0.048314884 | 0.605546552 |
| Q15113 | Procollagen C-endopeptidase enhancer 1 | PCOLCE | 1.023640762 | 0.884455903 | 0.450858043 | 1.961717032 | 0.049795438 | NA |

**Supplementary Table S2A**. Biological process gene ontology terms for the 19 DEPs in PPIP5K2-knockdown HCF cells with TGFβ1 treatment and CMS status as cofactors (FDR ≤0.05).

| [GO biological process complete](http://www.pantherdb.org/tools/compareToRefList.jsp?sortOrder=1&sortList=categories) | Reference [#](http://www.pantherdb.org/tools/compareToRefList.jsp?sortOrder=2&sortList=Homo%20sapiens) | Observed [#](http://www.pantherdb.org/tools/compareToRefList.jsp?sortOrder=2&sortList=Client%20Text%20Box%20Input&sortField=num) | [expected](http://www.pantherdb.org/tools/compareToRefList.jsp?sortOrder=2&sortList=Client%20Text%20Box%20Input&sortField=exp) # | [Fold Enrichment](http://www.pantherdb.org/tools/compareToRefList.jsp?sortOrder=2&sortList=Client%20Text%20Box%20Input&sortField=foldEnrich) | [+/-](http://www.pantherdb.org/tools/compareToRefList.jsp?sortOrder=1&sortList=Client%20Text%20Box%20Input&sortField=rep) | [raw P value](http://www.pantherdb.org/tools/compareToRefList.jsp?sortOrder=1&sortList=Client%20Text%20Box%20Input&sortField=pval) | | [FDR](http://www.pantherdb.org/tools/compareToRefList.jsp?sortOrder=2&sortList=Client%20Text%20Box%20Input&sortField=fdr) | |  |
| --- | --- | --- | --- | --- | --- | --- | --- | --- | --- | --- |
| [response to L-ascorbic acid](http://amigo.geneontology.org/amigo/term/GO:0033591) | [6](http://www.pantherdb.org/tools/gxIdsList.do?acc=GO:0033591&reflist=1) | [2](http://www.pantherdb.org/tools/gxIdsList.do?acc=GO:0033591&list=Client%20Text%20Box%20Input&organism=Homo%20sapiens) | .01 | > 100 | + | | 2.76E-05 | | 3.57E-02 | |
| [glycolytic process](http://amigo.geneontology.org/amigo/term/GO:0006096) | [45](http://www.pantherdb.org/tools/gxIdsList.do?acc=GO:0006096&reflist=1) | [5](http://www.pantherdb.org/tools/gxIdsList.do?acc=GO:0006096&list=Client%20Text%20Box%20Input&organism=Homo%20sapiens) | .05 | > 100 | + | | 1.35E-09 | | 2.10E-05 | |
| 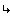[carbohydrate catabolic process](http://amigo.geneontology.org/amigo/term/GO:0016052) | [112](http://www.pantherdb.org/tools/gxIdsList.do?acc=GO:0016052&reflist=1) | [5](http://www.pantherdb.org/tools/gxIdsList.do?acc=GO:0016052&list=Client%20Text%20Box%20Input&organism=Homo%20sapiens) | .11 | 43.77 | + | | 1.02E-07 | | 5.30E-04 | |
| 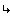[pyruvate metabolic process](http://amigo.geneontology.org/amigo/term/GO:0006090) | [71](http://www.pantherdb.org/tools/gxIdsList.do?acc=GO:0006090&reflist=1) | [5](http://www.pantherdb.org/tools/gxIdsList.do?acc=GO:0006090&list=Client%20Text%20Box%20Input&organism=Homo%20sapiens) | .07 | 69.04 | + | | 1.16E-08 | | 9.00E-05 | |
| 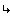[monocarboxylic acid metabolic process](http://amigo.geneontology.org/amigo/term/GO:0032787) | [523](http://www.pantherdb.org/tools/gxIdsList.do?acc=GO:0032787&reflist=1) | [6](http://www.pantherdb.org/tools/gxIdsList.do?acc=GO:0032787&list=Client%20Text%20Box%20Input&organism=Homo%20sapiens) | .53 | 11.25 | + | | 1.09E-05 | | 1.87E-02 | |
| 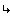[carboxylic acid metabolic process](http://amigo.geneontology.org/amigo/term/GO:0019752) | [841](http://www.pantherdb.org/tools/gxIdsList.do?acc=GO:0019752&reflist=1) | [8](http://www.pantherdb.org/tools/gxIdsList.do?acc=GO:0019752&list=Client%20Text%20Box%20Input&organism=Homo%20sapiens) | .86 | 9.33 | + | | 1.01E-06 | | 3.93E-03 | |
| 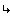[oxoacid metabolic process](http://amigo.geneontology.org/amigo/term/GO:0043436) | [863](http://www.pantherdb.org/tools/gxIdsList.do?acc=GO:0043436&reflist=1) | [8](http://www.pantherdb.org/tools/gxIdsList.do?acc=GO:0043436&list=Client%20Text%20Box%20Input&organism=Homo%20sapiens) | .88 | 9.09 | + | | 1.23E-06 | | 3.81E-03 | |
| 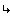[organic acid metabolic process](http://amigo.geneontology.org/amigo/term/GO:0006082) | [870](http://www.pantherdb.org/tools/gxIdsList.do?acc=GO:0006082&reflist=1) | [8](http://www.pantherdb.org/tools/gxIdsList.do?acc=GO:0006082&list=Client%20Text%20Box%20Input&organism=Homo%20sapiens) | .89 | 9.02 | + | | 1.30E-06 | | 3.37E-03 | |
| [hexose metabolic process](http://amigo.geneontology.org/amigo/term/GO:0019318) | [162](http://www.pantherdb.org/tools/gxIdsList.do?acc=GO:0019318&reflist=1) | [4](http://www.pantherdb.org/tools/gxIdsList.do?acc=GO:0019318&list=Client%20Text%20Box%20Input&organism=Homo%20sapiens) | .17 | 24.21 | + | | 2.18E-05 | | 3.08E-02 | |
| 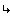[monosaccharide metabolic process](http://amigo.geneontology.org/amigo/term/GO:0005996) | [192](http://www.pantherdb.org/tools/gxIdsList.do?acc=GO:0005996&reflist=1) | [4](http://www.pantherdb.org/tools/gxIdsList.do?acc=GO:0005996&list=Client%20Text%20Box%20Input&organism=Homo%20sapiens) | .20 | 20.43 | + | | 4.18E-05 | | 5.00E-02 | |
| [response to hypoxia](http://amigo.geneontology.org/amigo/term/GO:0001666) | [272](http://www.pantherdb.org/tools/gxIdsList.do?acc=GO:0001666&reflist=1) | [5](http://www.pantherdb.org/tools/gxIdsList.do?acc=GO:0001666&list=Client%20Text%20Box%20Input&organism=Homo%20sapiens) | .28 | 18.02 | + | | 7.22E-06 | | 1.60E-02 | |
| 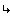[response to decreased oxygen levels](http://amigo.geneontology.org/amigo/term/GO:0036293) | [286](http://www.pantherdb.org/tools/gxIdsList.do?acc=GO:0036293&reflist=1) | [5](http://www.pantherdb.org/tools/gxIdsList.do?acc=GO:0036293&list=Client%20Text%20Box%20Input&organism=Homo%20sapiens) | .29 | 17.14 | + | | 9.17E-06 | | 1.78E-02 | |
| 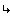[response to oxygen levels](http://amigo.geneontology.org/amigo/term/GO:0070482) | [314](http://www.pantherdb.org/tools/gxIdsList.do?acc=GO:0070482&reflist=1) | [5](http://www.pantherdb.org/tools/gxIdsList.do?acc=GO:0070482&list=Client%20Text%20Box%20Input&organism=Homo%20sapiens) | .32 | 15.61 | + | | 1.43E-05 | | 2.22E-02 | |

**Supplementary Table S2B**. Molecular function gene ontology terms for the 19 DEPs in PPIP5K2-knockdown HCF cells with TGFβ1 treatment and CMS status as cofactors (FDR ≤0.05).

| [GO molecular function complete](http://www.pantherdb.org/tools/compareToRefList.jsp?sortOrder=1&sortList=categories) | Reference [#](http://www.pantherdb.org/tools/compareToRefList.jsp?sortOrder=2&sortList=Homo%20sapiens) | Observed [#](http://www.pantherdb.org/tools/compareToRefList.jsp?sortOrder=2&sortList=Client%20Text%20Box%20Input&sortField=num) | [Expected](http://www.pantherdb.org/tools/compareToRefList.jsp?sortOrder=2&sortList=Client%20Text%20Box%20Input&sortField=exp) # | [Fold Enrichment](http://www.pantherdb.org/tools/compareToRefList.jsp?sortOrder=2&sortList=Client%20Text%20Box%20Input&sortField=foldEnrich) | [+/-](http://www.pantherdb.org/tools/compareToRefList.jsp?sortOrder=1&sortList=Client%20Text%20Box%20Input&sortField=rep) | [raw P value](http://www.pantherdb.org/tools/compareToRefList.jsp?sortOrder=1&sortList=Client%20Text%20Box%20Input&sortField=pval) | [FDR](http://www.pantherdb.org/tools/compareToRefList.jsp?sortOrder=2&sortList=Client%20Text%20Box%20Input&sortField=fdr) |
| --- | --- | --- | --- | --- | --- | --- | --- |
| [oxidoreductase activity, acting on a sulfur group of donors](http://amigo.geneontology.org/amigo/term/GO:0016667) | [55](http://www.pantherdb.org/tools/gxIdsList.do?acc=GO:0016667&reflist=1) | [3](http://www.pantherdb.org/tools/gxIdsList.do?acc=GO:0016667&list=Client%20Text%20Box%20Input&organism=Homo%20sapiens) | .06 | 53.48 | + | 2.71E-05 | 4.57E-02 |
| 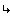[oxidoreductase activity](http://amigo.geneontology.org/amigo/term/GO:0016491) | [736](http://www.pantherdb.org/tools/gxIdsList.do?acc=GO:0016491&reflist=1) | [8](http://www.pantherdb.org/tools/gxIdsList.do?acc=GO:0016491&list=Client%20Text%20Box%20Input&organism=Homo%20sapiens) | .75 | 10.66 | + | 3.72E-07 | 1.88E-03 |
| [small molecule binding](http://amigo.geneontology.org/amigo/term/GO:0036094) | [2587](http://www.pantherdb.org/tools/gxIdsList.do?acc=GO:0036094&reflist=1) | [11](http://www.pantherdb.org/tools/gxIdsList.do?acc=GO:0036094&list=Client%20Text%20Box%20Input&organism=Homo%20sapiens) | 2.64 | 4.17 | + | 1.31E-05 | 3.31E-02 |

**Supplementary Table S2C**. Cellular Component gene ontology terms for the 19 DEPs in PPIP5K2-knockdown HCF cells with TGFβ1 treatment and CMS status as cofactors (FDR ≤0.05).

| [GO cellular component complete](http://www.pantherdb.org/tools/compareToRefList.jsp?sortOrder=1&sortList=categories) | Reference [#](http://www.pantherdb.org/tools/compareToRefList.jsp?sortOrder=2&sortList=Homo%20sapiens) | Observed [#](http://www.pantherdb.org/tools/compareToRefList.jsp?sortOrder=2&sortList=Client%20Text%20Box%20Input&sortField=num) | [expected](http://www.pantherdb.org/tools/compareToRefList.jsp?sortOrder=2&sortList=Client%20Text%20Box%20Input&sortField=exp) # | [Fold Enrichment](http://www.pantherdb.org/tools/compareToRefList.jsp?sortOrder=2&sortList=Client%20Text%20Box%20Input&sortField=foldEnrich) | [+/-](http://www.pantherdb.org/tools/compareToRefList.jsp?sortOrder=1&sortList=Client%20Text%20Box%20Input&sortField=rep) | [raw P value](http://www.pantherdb.org/tools/compareToRefList.jsp?sortOrder=1&sortList=Client%20Text%20Box%20Input&sortField=pval) | [FDR](http://www.pantherdb.org/tools/compareToRefList.jsp?sortOrder=2&sortList=Client%20Text%20Box%20Input&sortField=fdr) |
| --- | --- | --- | --- | --- | --- | --- | --- |
| [nucleosome](http://amigo.geneontology.org/amigo/term/GO:0000786) | [99](http://www.pantherdb.org/tools/gxIdsList.do?acc=GO:0000786&reflist=1) | [3](http://www.pantherdb.org/tools/gxIdsList.do?acc=GO:0000786&list=Client%20Text%20Box%20Input&organism=Homo%20sapiens) | .10 | 29.71 | + | 1.47E-04 | 3.23E-02 |
| [extracellular exosome](http://amigo.geneontology.org/amigo/term/GO:0070062) | [2101](http://www.pantherdb.org/tools/gxIdsList.do?acc=GO:0070062&reflist=1) | [15](http://www.pantherdb.org/tools/gxIdsList.do?acc=GO:0070062&list=Client%20Text%20Box%20Input&organism=Homo%20sapiens) | 2.14 | 7.00 | + | 4.22E-11 | 8.35E-08 |
| 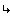[extracellular vesicle](http://amigo.geneontology.org/amigo/term/GO:1903561) | [2124](http://www.pantherdb.org/tools/gxIdsList.do?acc=GO:1903561&reflist=1) | [15](http://www.pantherdb.org/tools/gxIdsList.do?acc=GO:1903561&list=Client%20Text%20Box%20Input&organism=Homo%20sapiens) | 2.17 | 6.92 | + | 4.93E-11 | 4.88E-08 |
| 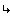[extracellular membrane-bounded organelle](http://amigo.geneontology.org/amigo/term/GO:0065010) | [2125](http://www.pantherdb.org/tools/gxIdsList.do?acc=GO:0065010&reflist=1) | [15](http://www.pantherdb.org/tools/gxIdsList.do?acc=GO:0065010&list=Client%20Text%20Box%20Input&organism=Homo%20sapiens) | 2.17 | 6.92 | + | 4.96E-11 | 2.46E-08 |
| 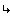[membrane-bounded organelle](http://amigo.geneontology.org/amigo/term/GO:0043227) | [13240](http://www.pantherdb.org/tools/gxIdsList.do?acc=GO:0043227&reflist=1) | [21](http://www.pantherdb.org/tools/gxIdsList.do?acc=GO:0043227&list=Client%20Text%20Box%20Input&organism=Homo%20sapiens) | 13.50 | 1.56 | + | 1.23E-04 | 3.04E-02 |
| 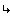[extracellular organelle](http://amigo.geneontology.org/amigo/term/GO:0043230) | [2125](http://www.pantherdb.org/tools/gxIdsList.do?acc=GO:0043230&reflist=1) | [15](http://www.pantherdb.org/tools/gxIdsList.do?acc=GO:0043230&list=Client%20Text%20Box%20Input&organism=Homo%20sapiens) | 2.17 | 6.92 | + | 4.96E-11 | 3.28E-08 |
| 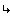[extracellular region](http://amigo.geneontology.org/amigo/term/GO:0005576) | [4292](http://www.pantherdb.org/tools/gxIdsList.do?acc=GO:0005576&reflist=1) | [16](http://www.pantherdb.org/tools/gxIdsList.do?acc=GO:0005576&list=Client%20Text%20Box%20Input&organism=Homo%20sapiens) | 4.38 | 3.65 | + | 8.89E-08 | 2.93E-05 |
| 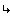[vesicle](http://amigo.geneontology.org/amigo/term/GO:0031982) | [3998](http://www.pantherdb.org/tools/gxIdsList.do?acc=GO:0031982&reflist=1) | [15](http://www.pantherdb.org/tools/gxIdsList.do?acc=GO:0031982&list=Client%20Text%20Box%20Input&organism=Homo%20sapiens) | 4.08 | 3.68 | + | 3.50E-07 | 9.90E-05 |
| 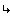[extracellular space](http://amigo.geneontology.org/amigo/term/GO:0005615) | [3358](http://www.pantherdb.org/tools/gxIdsList.do?acc=GO:0005615&reflist=1) | [16](http://www.pantherdb.org/tools/gxIdsList.do?acc=GO:0005615&list=Client%20Text%20Box%20Input&organism=Homo%20sapiens) | 3.43 | 4.67 | + | 2.29E-09 | 9.06E-07 |

**Supplementary Table S3**. (A) PANTHER (B) Reactome pathways for 19 significant differentially expressed proteins in HCF-PPIP5K2-shRNA1 with TGFβ1 treatment and CMS status as cofactors (FDR ≤ 0.05).

1. PANTHER Pathways

| [PANTHER Pathways](http://www.pantherdb.org/tools/compareToRefList.jsp?sortOrder=1&sortList=categories) | Reference [#](http://www.pantherdb.org/tools/compareToRefList.jsp?sortOrder=2&sortList=Homo%20sapiens) | Observed [#](http://www.pantherdb.org/tools/compareToRefList.jsp?sortOrder=2&sortList=Client%20Text%20Box%20Input&sortField=num) | [expected](http://www.pantherdb.org/tools/compareToRefList.jsp?sortOrder=2&sortList=Client%20Text%20Box%20Input&sortField=exp) # | [Fold Enrichment](http://www.pantherdb.org/tools/compareToRefList.jsp?sortOrder=2&sortList=Client%20Text%20Box%20Input&sortField=foldEnrich) | [+/-](http://www.pantherdb.org/tools/compareToRefList.jsp?sortOrder=1&sortList=Client%20Text%20Box%20Input&sortField=rep) | [raw P value](http://www.pantherdb.org/tools/compareToRefList.jsp?sortOrder=1&sortList=Client%20Text%20Box%20Input&sortField=pval) | [FDR](http://www.pantherdb.org/tools/compareToRefList.jsp?sortOrder=2&sortList=Client%20Text%20Box%20Input&sortField=fdr) |
| --- | --- | --- | --- | --- | --- | --- | --- |
| [Glycolysis](javascript:openDiagramWindow('/pathway/pathwayDiagram.jsp?color=1&catsInfo=true&catAccession=P00024%27);) | [20](http://www.pantherdb.org/tools/gxIdsList.do?acc=P00024&reflist=1) | [4](http://www.pantherdb.org/tools/gxIdsList.do?acc=P00024&list=Client%20Text%20Box%20Input&organism=Homo%20sapiens) | .02 | > 100 | + | 8.35E-09 | 1.34E-06 |

1. Reactome pathways

| [Reactome pathways](http://www.pantherdb.org/tools/compareToRefList.jsp?sortOrder=1&sortList=categories) | Reference [#](http://www.pantherdb.org/tools/compareToRefList.jsp?sortOrder=2&sortList=Homo%20sapiens) | Observed [#](http://www.pantherdb.org/tools/compareToRefList.jsp?sortOrder=2&sortList=Client%20Text%20Box%20Input&sortField=num) | [Expected](http://www.pantherdb.org/tools/compareToRefList.jsp?sortOrder=2&sortList=Client%20Text%20Box%20Input&sortField=exp) # | [Fold Enrichment](http://www.pantherdb.org/tools/compareToRefList.jsp?sortOrder=2&sortList=Client%20Text%20Box%20Input&sortField=foldEnrich) | [+/-](http://www.pantherdb.org/tools/compareToRefList.jsp?sortOrder=1&sortList=Client%20Text%20Box%20Input&sortField=rep) | [raw P value](http://www.pantherdb.org/tools/compareToRefList.jsp?sortOrder=1&sortList=Client%20Text%20Box%20Input&sortField=pval) | [FDR](http://www.pantherdb.org/tools/compareToRefList.jsp?sortOrder=2&sortList=Client%20Text%20Box%20Input&sortField=fdr) |
| --- | --- | --- | --- | --- | --- | --- | --- |
| [Gluconeogenesis](http://www.reactome.org/PathwayBrowser/#/R-HSA-70263) | [33](http://www.pantherdb.org/tools/gxIdsList.do?acc=R-HSA-70263&reflist=1) | [3](http://www.pantherdb.org/tools/gxIdsList.do?acc=R-HSA-70263&list=Client%20Text%20Box%20Input&organism=Homo%20sapiens) | .03 | 89.13 | + | 6.37E-06 | 1.59E-02 |
| 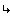[Glucose metabolism](http://www.reactome.org/PathwayBrowser/#/R-HSA-70326) | [86](http://www.pantherdb.org/tools/gxIdsList.do?acc=R-HSA-70326&reflist=1) | [3](http://www.pantherdb.org/tools/gxIdsList.do?acc=R-HSA-70326&list=Client%20Text%20Box%20Input&organism=Homo%20sapiens) | .09 | 34.20 | + | 9.79E-05 | 4.88E-02 |
| [Interleukin-12 signaling](http://www.reactome.org/PathwayBrowser/#/R-HSA-9020591) | [46](http://www.pantherdb.org/tools/gxIdsList.do?acc=R-HSA-9020591&reflist=1) | [3](http://www.pantherdb.org/tools/gxIdsList.do?acc=R-HSA-9020591&list=Client%20Text%20Box%20Input&organism=Homo%20sapiens) | .05 | 63.94 | + | 1.63E-05 | 2.03E-02 |
| 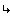[Interleukin-12 family signaling](http://www.reactome.org/PathwayBrowser/#/R-HSA-447115) | [56](http://www.pantherdb.org/tools/gxIdsList.do?acc=R-HSA-447115&reflist=1) | [3](http://www.pantherdb.org/tools/gxIdsList.do?acc=R-HSA-447115&list=Client%20Text%20Box%20Input&organism=Homo%20sapiens) | .06 | 52.52 | + | 2.86E-05 | 2.37E-02 |
| [Glycolysis](http://www.reactome.org/PathwayBrowser/#/R-HSA-70171) | [67](http://www.pantherdb.org/tools/gxIdsList.do?acc=R-HSA-70171&reflist=1) | [3](http://www.pantherdb.org/tools/gxIdsList.do?acc=R-HSA-70171&list=Client%20Text%20Box%20Input&organism=Homo%20sapiens) | .07 | 43.90 | + | 4.78E-05 | 2.98E-02 |

**SUPPLEMENTARY FIGURE S1**

**
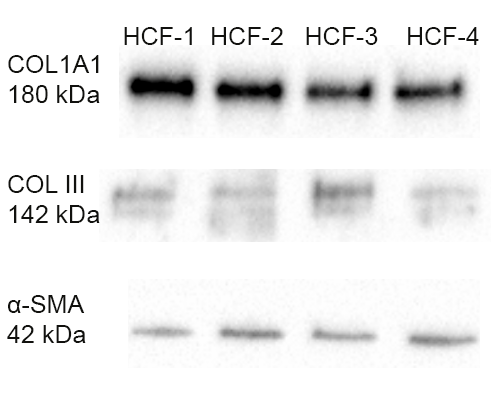
**

**Supplementary Figure S1**. Validation of protein expression of α-smooth muscle actin (αSMA), COL1A1, and COL3A1 from the four primary human corneal stromal cell donors using antibody-based western blots with separate western blots against each marker protein. We used rabbit anti-αSMA antibody (Abcam, catalog # ab32575, with 1:3000 dilution, Waltham, MA, USA), rabbit anti-COL1A1 antibody (Invitrogen, catalog # PA5-29569, with 1:1000 dilution, Carlsbad, CA, USA), and goat anti-COL3A1 antibody (SouthernBioTech, catalog # 1330-01, with 1:500 dilution, Homewood, AL, USA). The secondary antibody was peroxidase-conjugated anti-rabbit IgG secondary antibody (ThermoFisher Scientific, Catalog# 31460, Waltham, MA). For the western blots with αSMA and COL1A1, total protein loading was 5μg per sample while 100μg was used for the western blot with COL3A1. Membranes were exposed to Clarity enhanced chemiluminescence (ECL) reagent (Bio-Rad) and visualized using a ChemiDoc MP (Bio-Rad). Detection and quantification of band intensities was conducted using Image Lab 5.2.1 software (Bio-Rad).

**SUPPLEMENTARY FIGURE S2**

**
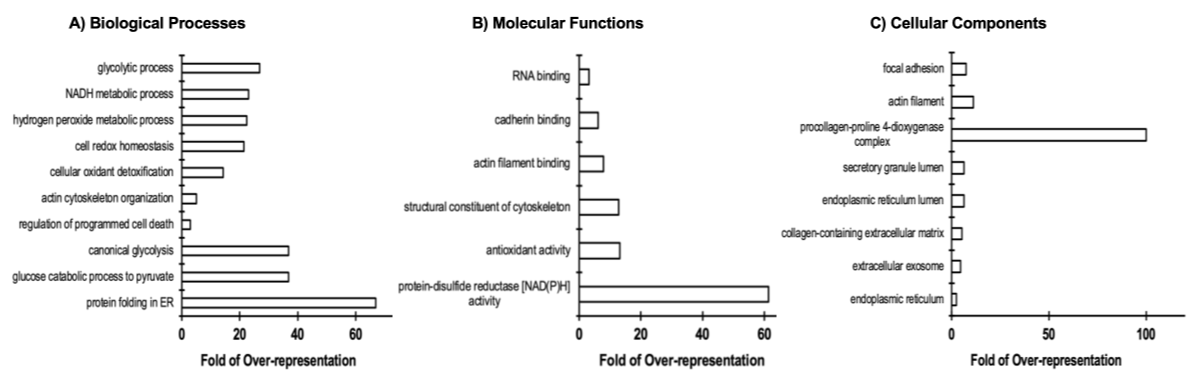
**

**Supplementary Figure S2**. Gene ontology terms showing over-represented groups of proteins involved in (a) biological processes, (b) molecular functions and (c) cellular components for the 110 significant differentially expressed proteins in PPIP5K2-knockdown HCF cells with TGFβ1 treatment and CMS status as cofactors, compared to those HCFs without PPIP5K2 knockdown (FDR ≤ 0.05).

**SUPPLEMENTARY FIGURE S3**


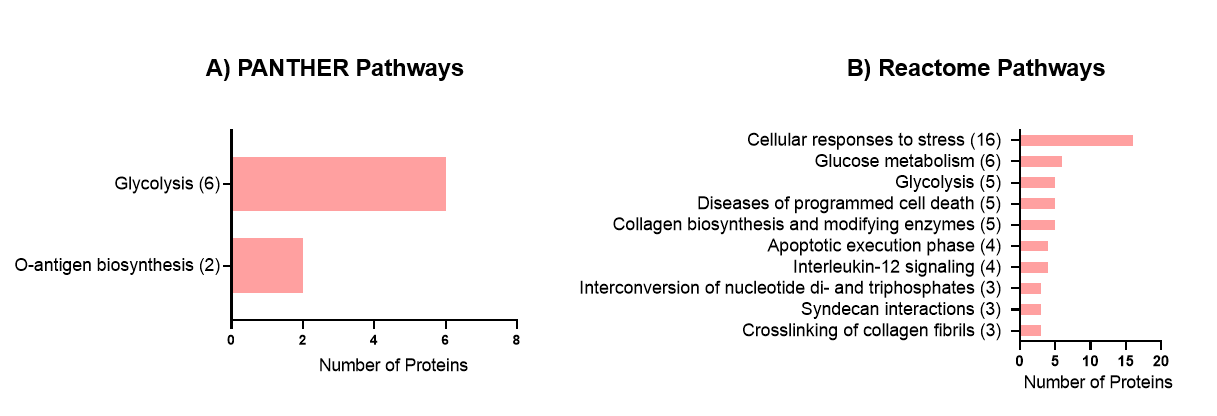


**Supplementary Figure S3**. (A) PANTHER (B) Reactome pathways for the 110 significant differentially expressed proteins in PPIP5K2-knockdown HCF cells with TGFβ1 treatment and CMS status as cofactors, compared to HCFs with scrambled controls (FDR ≤ 0.05). Numbers in brackets are the number of proteins involved in each process.

**SUPPLEMENTARY FIGURE S4**


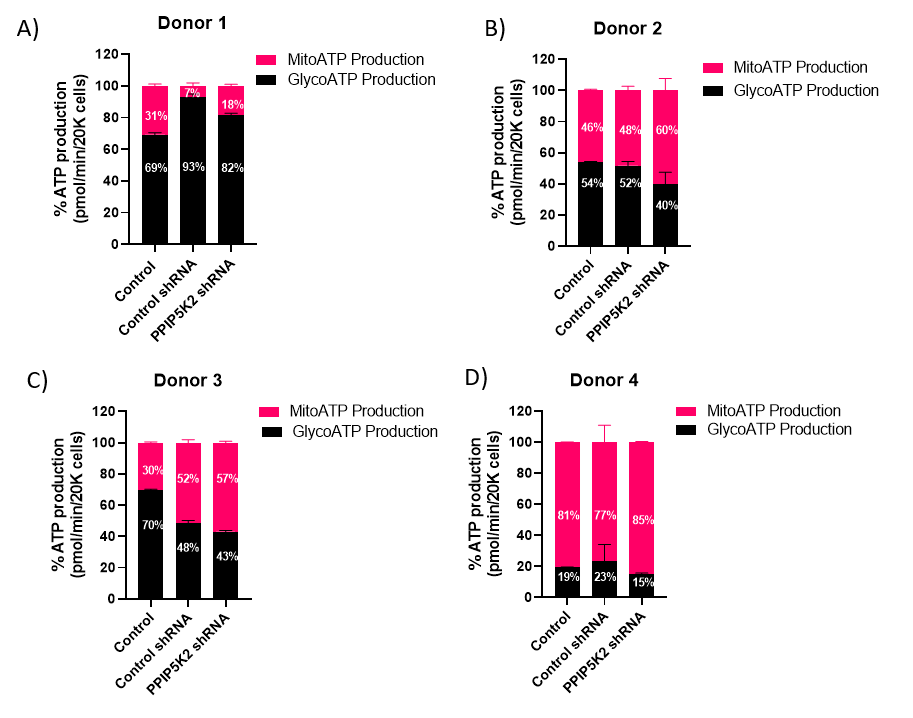


Supplementary Figure S4: ATP Profiles of human corneal stromal fibroblasts with or without PPIP5K2 knockdown from 4 different donors.

(A) Real-time ATP rate assay in primary human corneal stromal fibroblasts in donor 1 with control (no transduction), control shRNA, and shRNA against *PPIP5K2*. Approximately 93% and 7% of ATP was generated from glycolysis and oxidative phosphorylation, respectively in the corneal stromal fibroblasts. A knockdown of *PPIP5K2* slightly reduced ATP production from glycolysis to favor oxidative phosphorylation. (B) ATP profile in donor 2, approximately 52% and 48% of ATP was generated from glycolysis and oxidative phosphorylation, respectively in the corneal stromal fibroblasts. A knockdown of *PPIP5K2* slightly reduced ATP production from glycolysis to favor oxidative phosphorylation. (C) ATP profile in donor 3, approximately 48% and 52% of ATP was generated from glycolysis and oxidative phosphorylation, respectively in the corneal stromal fibroblasts. A knockdown of *PPIP5K2* slightly reduced ATP production from glycolysis to favor oxidative phosphorylation. (D) ATP profile in donor 4, Majority of ATP was generated from oxidative phosphorylation in the corneal stromal fibroblasts. A knockdown of *PPIP5K2* further increased ATP production from oxidative phosphorylation.

**SUPPLEMENTARY FIGURE S5**


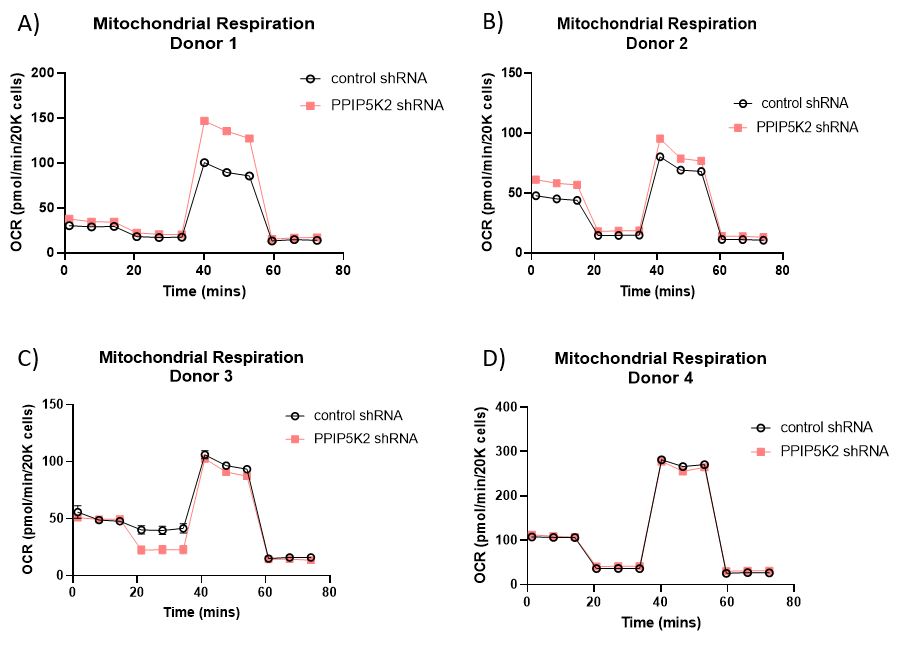


Supplementary Figure S5: Variability in mitochondrial function profiles from 4 donor HCF cells.

Mito Stress test assay in primary human corneal stromal fibroblasts in donors 1 (A), 2 (B), 3 (C), and 4 (D) with HCF cells with PPIP5K2 knockdown or scrambled controls. Each assay for each donor was completed with eight technical replicates. Each data point represents an OCR measurement.

**SUPPLEMENTARY FIGURE S6**


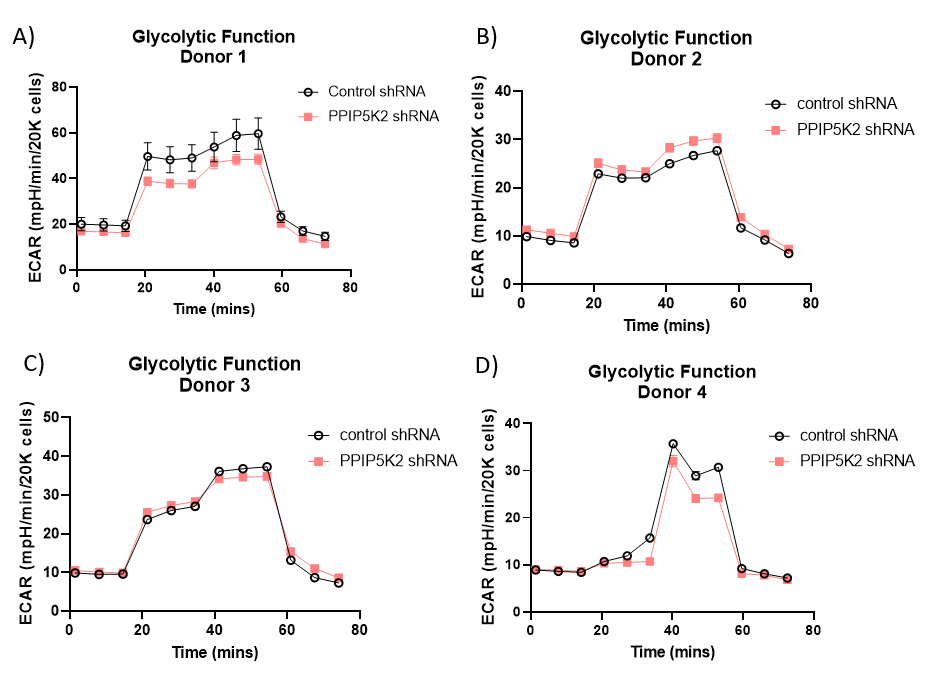


Supplementary Figure S6: Variability in glycolytic function profiles from 4 donor HCF cells.

(A-D) Glycolysis stress test assay in primary human corneal stromal fibroblasts in donors 1, 2, 3 and 4 with HCF cells with PPIP5K2 knockdown or scrambled controls. Each data point represents an ECAR measurement with 8 technical replicates.
